# Supplementary material for: A revamped rat reference genome improves the discovery of genetic diversity in laboratory rats
Source: Cell Genom. 2024 Mar 26;4(4):100527. doi: 10.1016/j.xgen.2024.100527 (PMC11019364; doi:10.1016/j.xgen.2024.100527)
Supplement: Document S1. Figures S1–S22 [file mmc1.pdf]

Cell Genomics, Volume *4*

Supplemental information

A revamped rat reference genome improves the discovery of genetic diversity in laboratory rats

Tristan V. de Jong, Yanchao Pan, Pasi Rastas, Daniel Munro, Monika Tutaj, Huda Akil, Chris Benner, Denghui Chen, Apurva S. Chitre, William Chow, Vincenza Colonna, Clifton L. Dalgard, Wendy M. Demos, Peter A. Doris, Erik Garrison, Aron M. Geurts, Hakan M. Gunturkun, Victor Guryev, Thibaut Hourlier, Kerstin Howe, Jun Huang, Ted Kalbfleisch, Panjun Kim, Ling Li, Spencer Mahaffey, Fergal J. Martin, Pejman Mohammadi, Ayse Bilge Ozel, Oksana Polesskaya, Michal Pravenec, Pjotr Prins, Jonathan Sebat, Jennifer R. Smith, Leah C. Solberg Woods, Boris Tabakoff, Alan Tracey, Marcela Uliano-Silva, Flavia Villani, Hongyang Wang, Burt M. Sharp, Francesca Telese, Zhihua Jiang, Laura Saba, Xusheng Wang, Terence D. Murphy, Abraham A. Palmer, Anne E. Kwitek, Melinda R. Dwinell, Robert W. Williams, Jun Z. Li, and Hao Chen

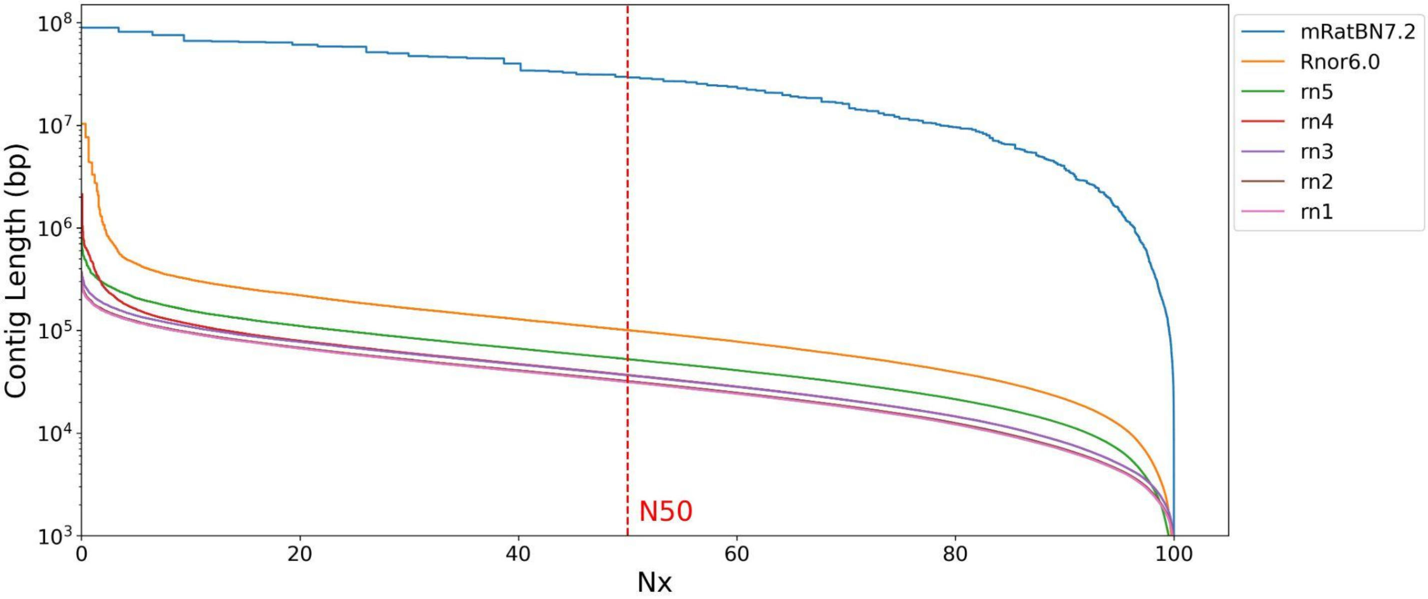

Figure S1 (related to Figure 1). The Contig Nx plot for mRatBN7.2 and 6 prior rat reference genomes. The red dashed line indicates the contig N50 values. Small improvements in contig continuity are observed for the previous updates, with the most significant improvement (~290X) coming from mRatBN7.2

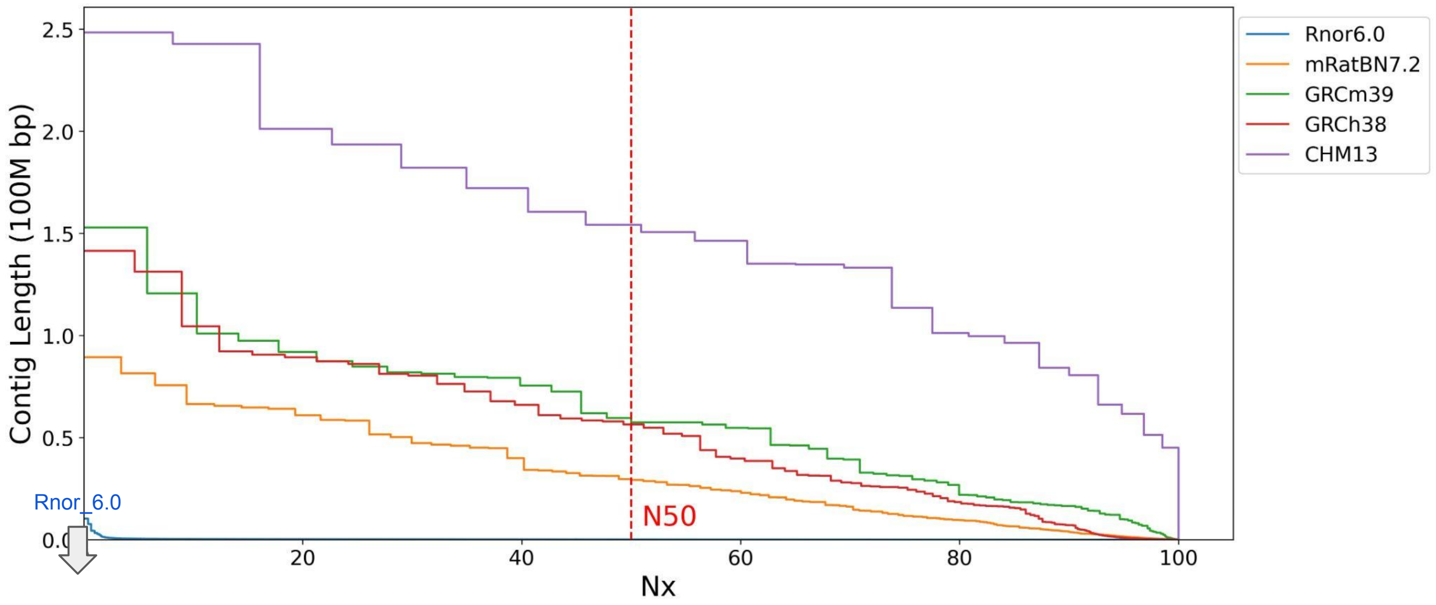

Figure S2 (related to Figure 1). The Contig Nx plot for the Rat, Mouse, and Human reference genomes. The red dashed line indicates the contig N50 values. The Nx curve for Rnor6.0 is very low and partially overlapping with the X axis. The CHM13, being the first truly gapless human genome, is in its own tier in terms of assembly continuity. The GRCh38 released in 2014 and the GRCm39 released in 2020 have similar continuity. Although mRatBN7.2 is still lagging behind in continuity compared to the current human and mouse reference genomes, it represent a very significant improvement over the current rat reference genome Rnor_6.0 (Orange vs. Cyan line).

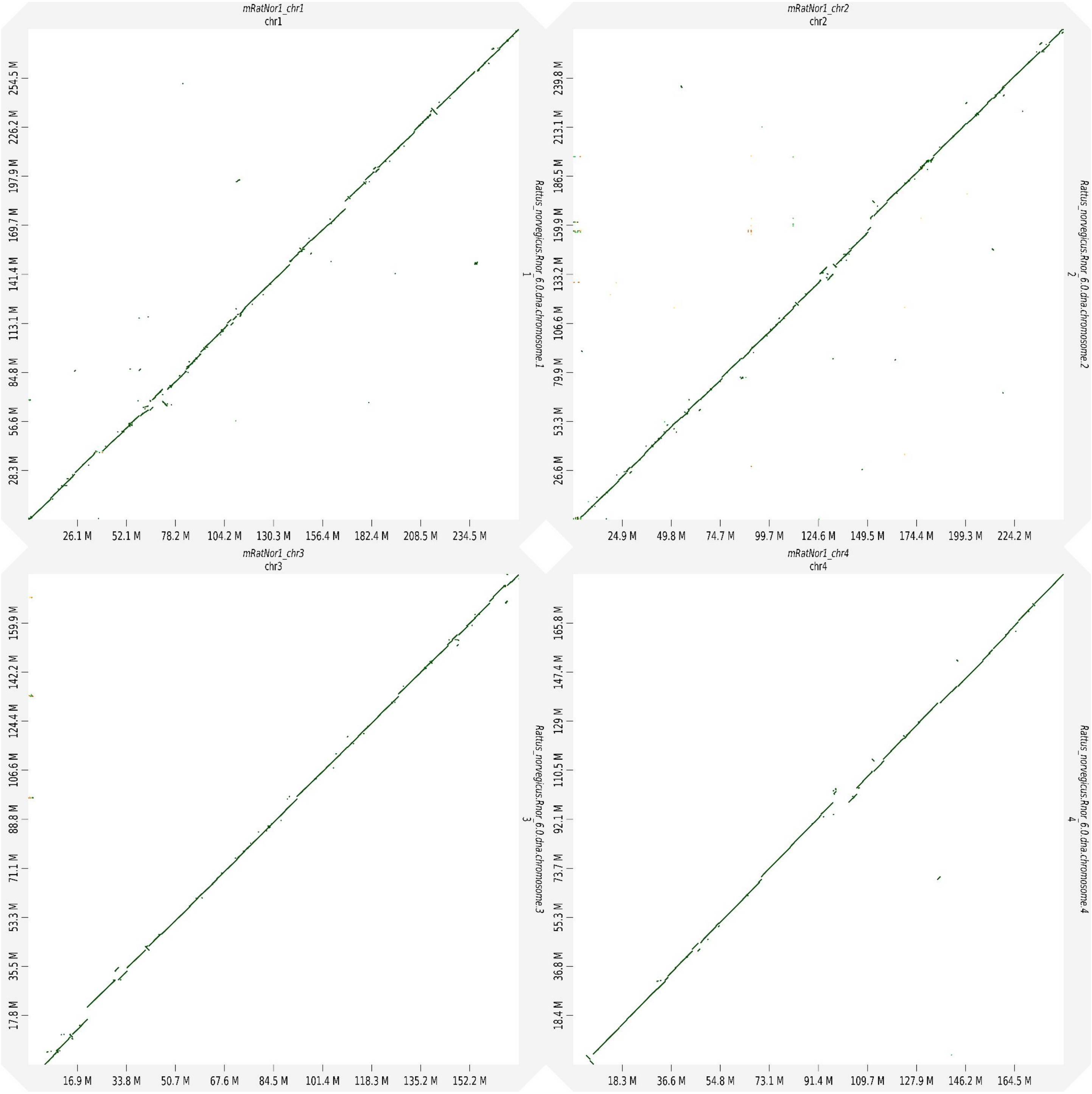

Figure S3 (related to Figure 1). Chromosomal dot plots between Rnor_6.0 and mRatBN7.2. mRatBN7.2 is on the x-axis and Rnor_6.0 is on the y-axis. mRatNor1 is the initial name of the assembly released by the Darwin Tree of Life/Vertebrate Genome Project. chr1-chr4 are shown here. To see the complete set, see the Key Resources Table "Chromosomal dot plots between Rnor_6.0 and mRatBN7.2" https://doi.org/10.5281/zenodo.10515796

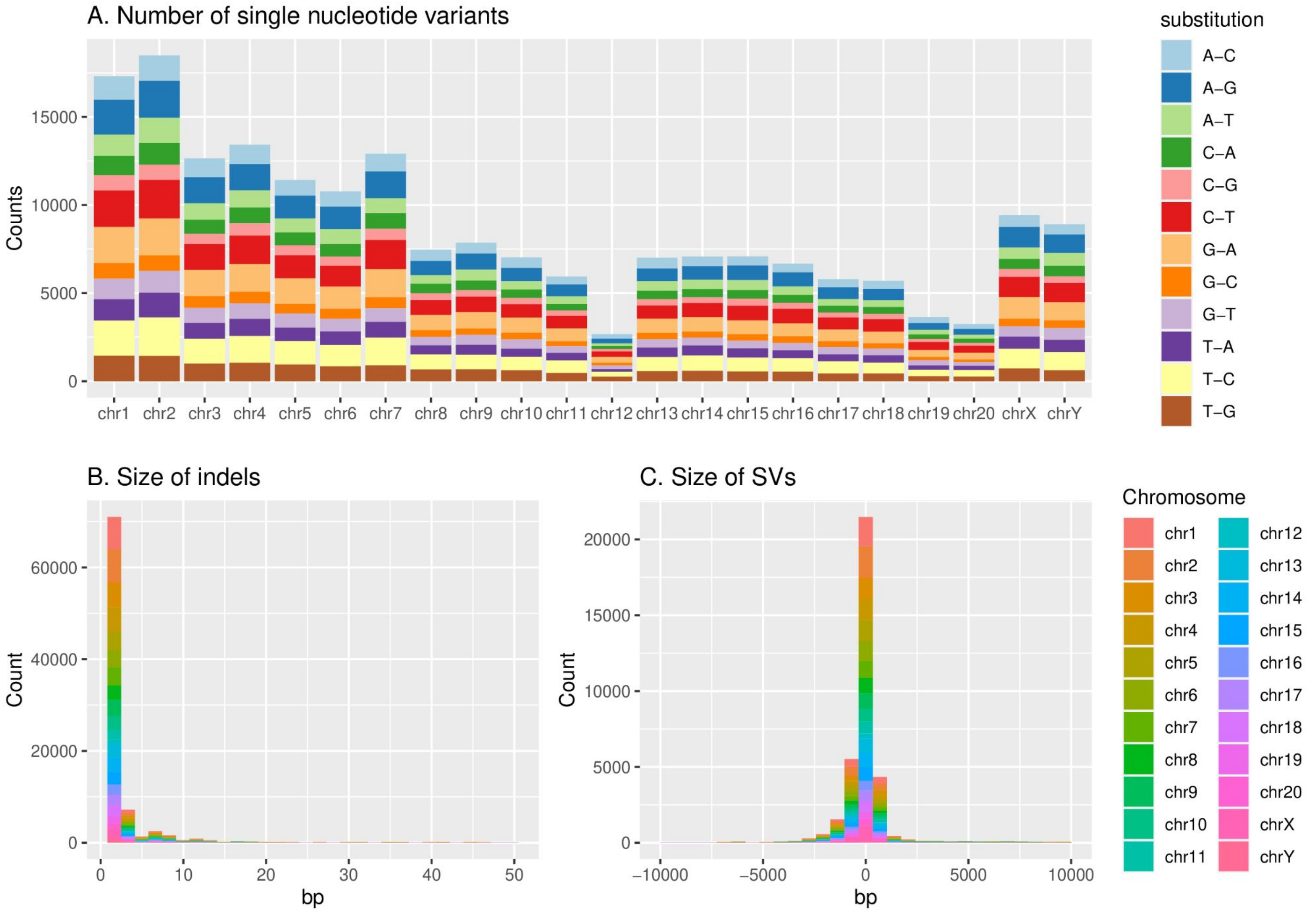

Figure S4 (related to Figure 1). SNP and structural variants between Rnor_6.0 and mRatBN7.2. A) The substitution frequency per chromosome for Rnor_6.0 and mRatBN7.2. **B)** The size of indels between Rnor 6.0 and mRatBN7.2. **C)** The size of SVs between Rnor6.0 and mRatBN7.2.

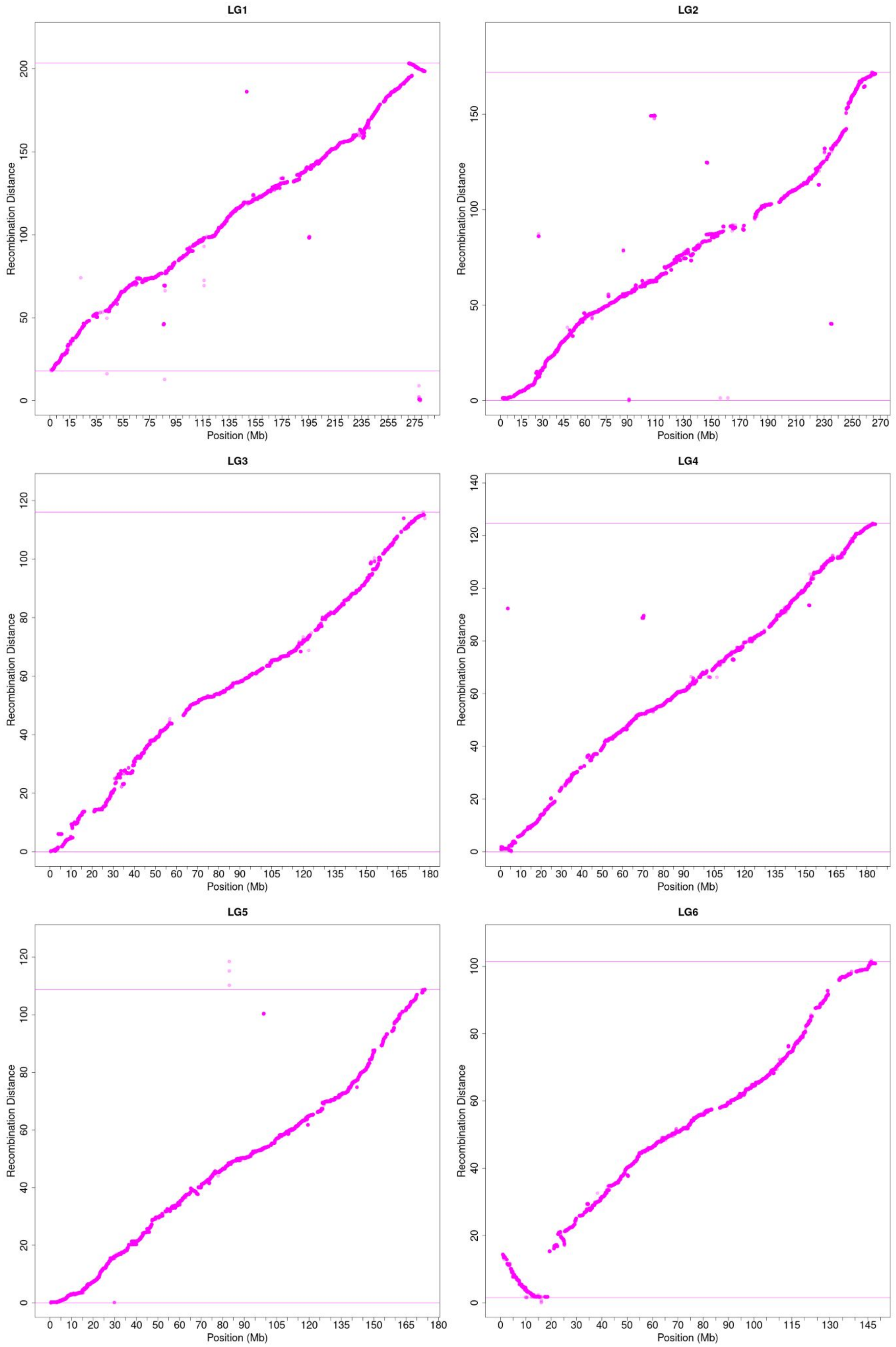

Figure S5 (related to Figure 1). The order of genetic markers and the distances from a rat genetic map compared to their locations in Rnor_6.0. Note the large inversion at proximal Chr 6. chr1-chr6 are shown here. To see the complete set, see the Key Resources Table "The order of genetic markers and the distances from a rat genetic map compared to their locations in Rnor_6.0" https://doi.org/10.5281/zenodo.10520087

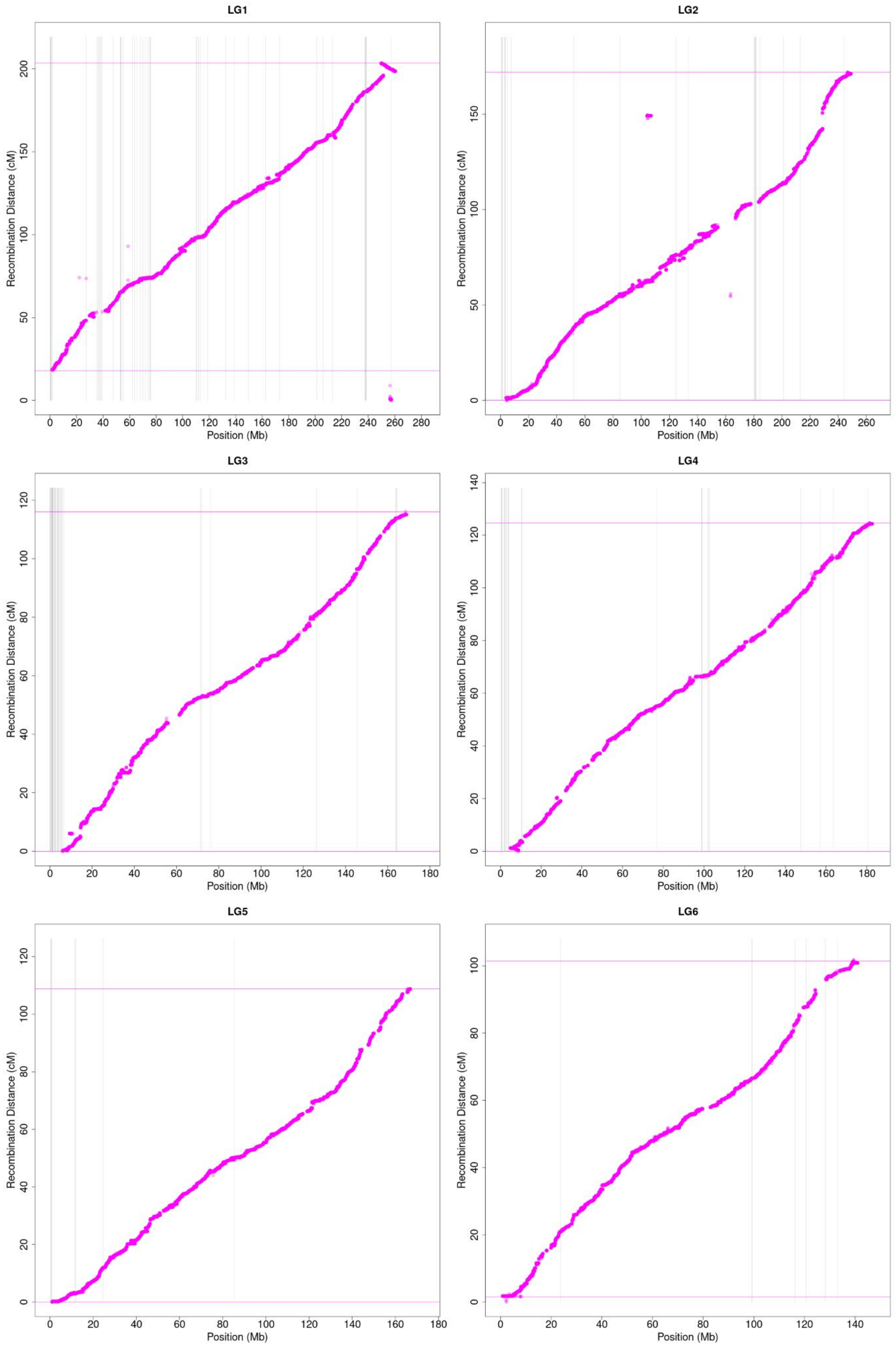

Figure S6 (related to Figure 1). The order of genetic markers and the distances from a rat genetic map compared to their locations in mRatBN7.2 chr1-chr6 are shown here. To see the complete set, see the Key Resources Table "The order of genetic markers and the distances from a rat genetic map compared to their locations in mRatBN7.2" https://doi.org/10.5281/zenodo.10520119

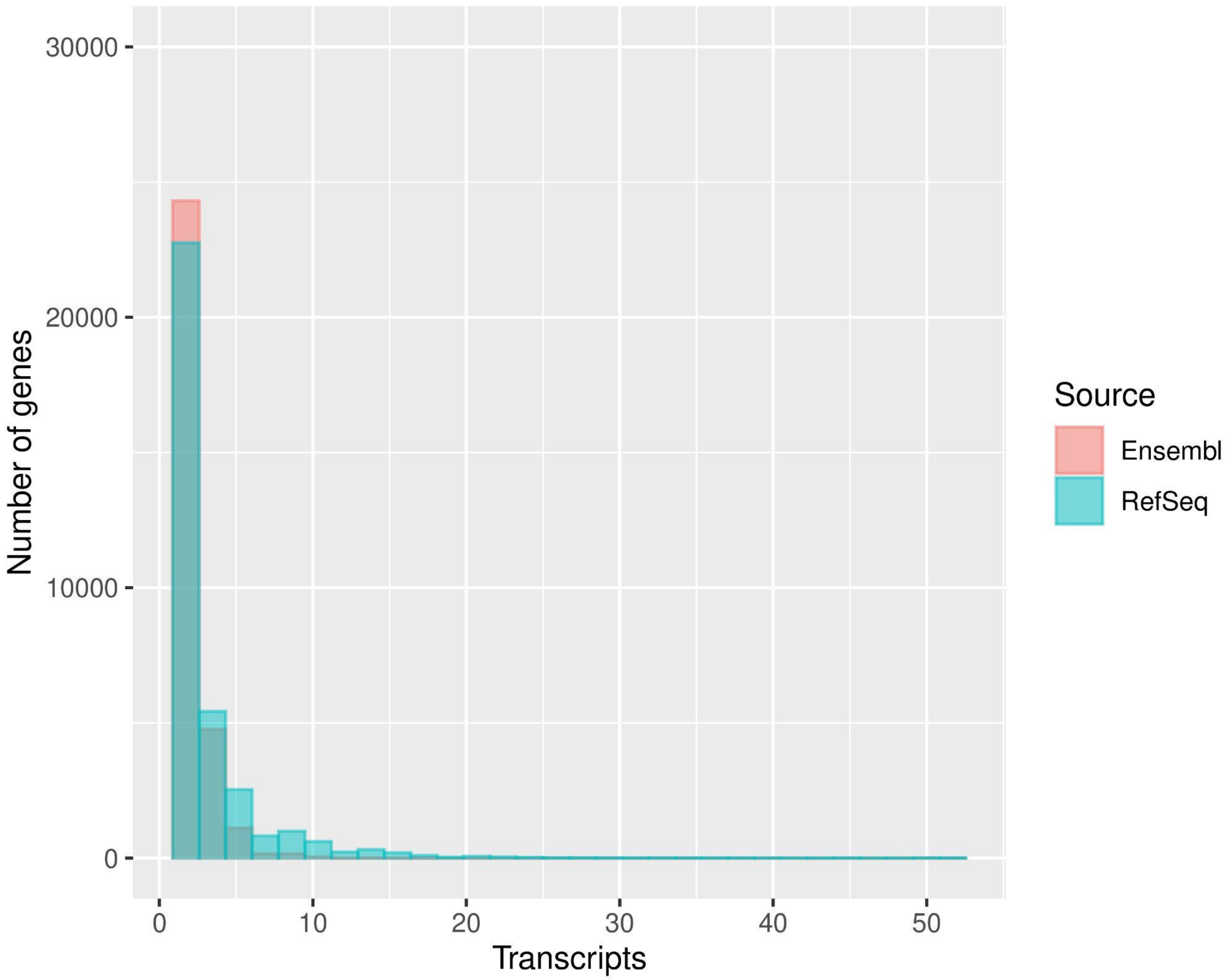

Figure S7 (related to Figure 1). The number of transcripts annotated by Ensembl and RefSeq. Refseq annotated a greater number of genes with multiple transcripts than Ensembl. The average number of transcripts per gene was 2.9 for RefSeq and 1.8 for Ensembl.

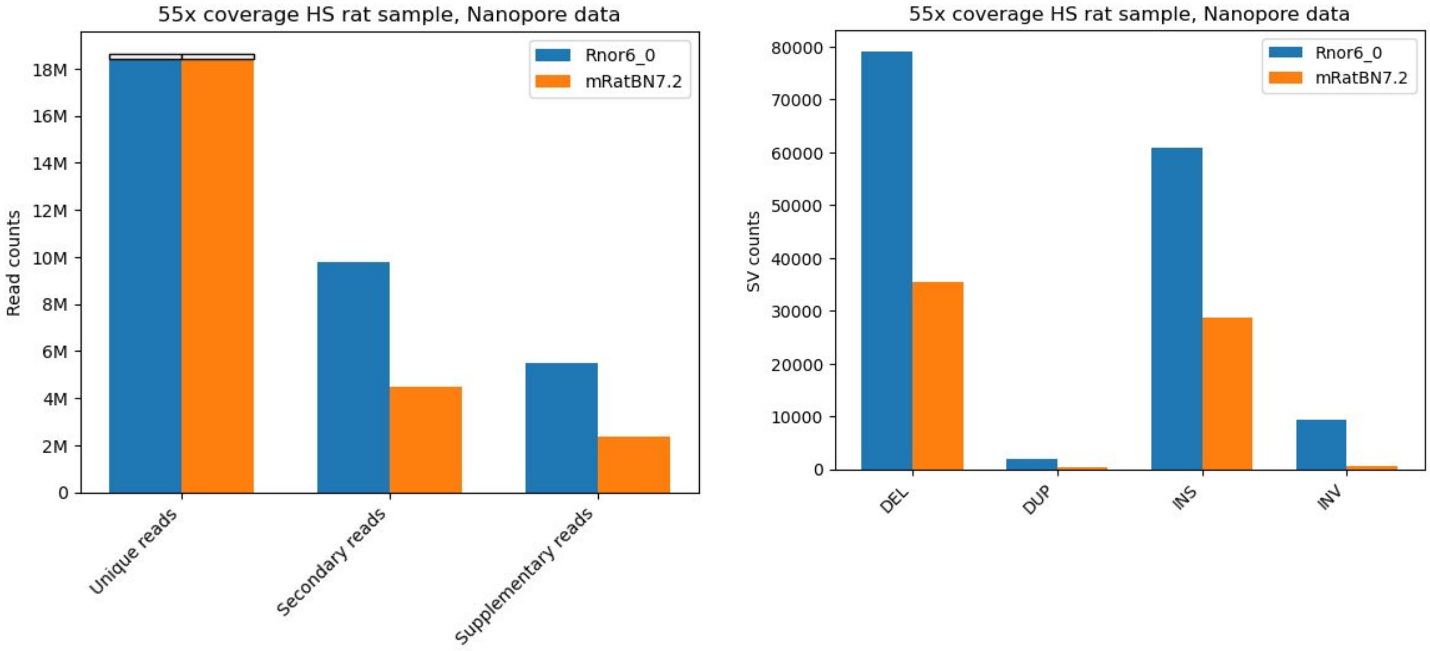

Figure S8 (related to Figure 2). Mapping results of nanopore data of one HS rat with 55x coverage. Using mRatBN7.2 as the reference decreased the number of structural variants compared to using Rnor_6.0 as the reference. In the context of our other findings, this is likely due to the improved quality of the reference genome.

A

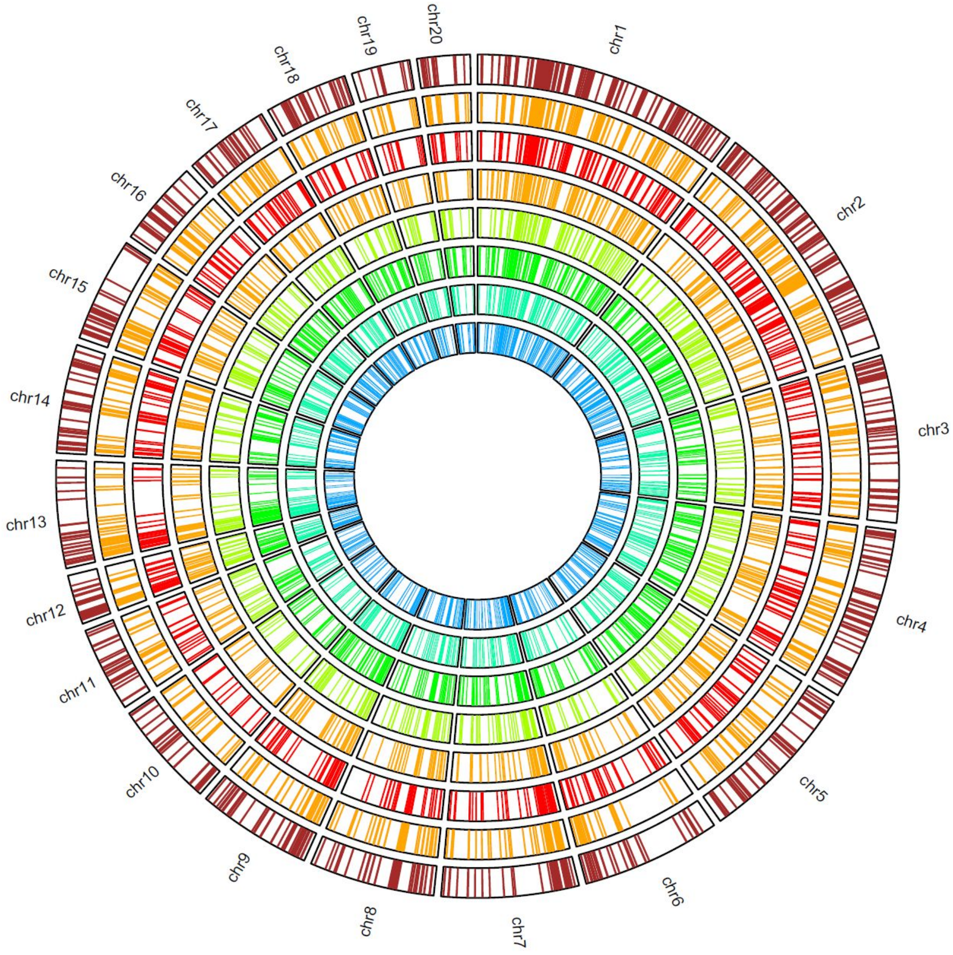

B

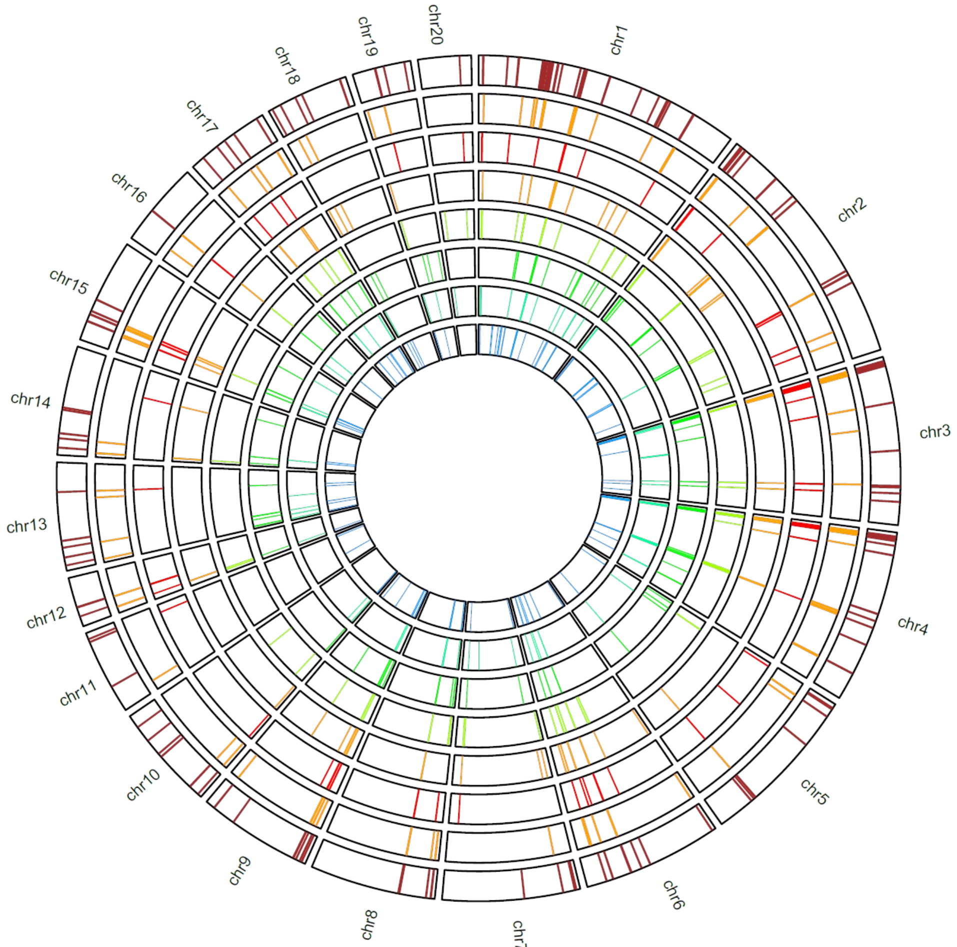

Figure S9 (related to Figure 2). Locations of large deletions from multiple linked-read samples. A) Rnor_6.0 B) mRatBN7.2. From outer circle to inner circle are the following strains: BN, SHR/Olalpcv, BXH10, BXH8, BXH2, HXB17, HXB2, and HXB21.

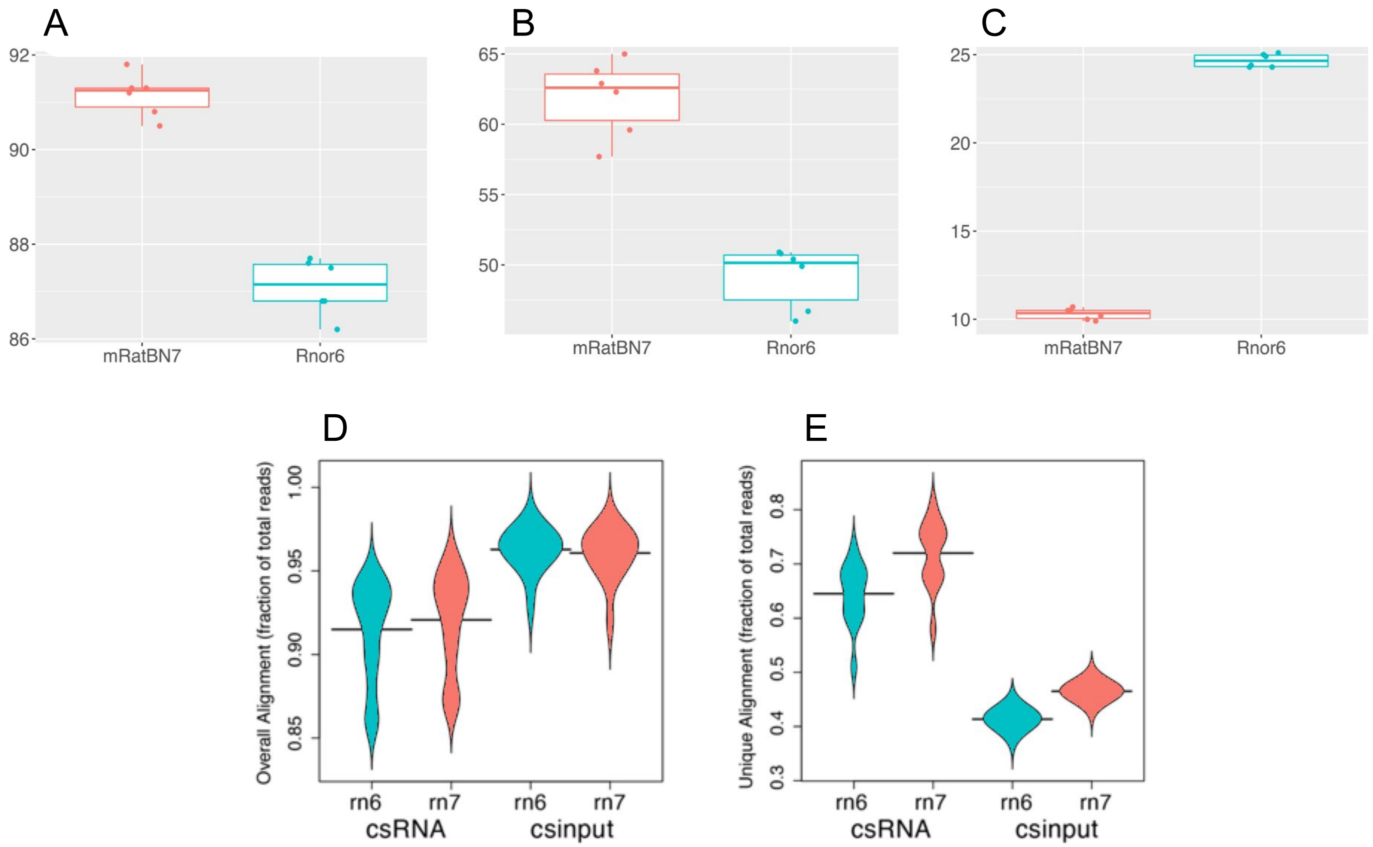

Figure S10 (related to Figure 2). Mapping metrics of single nuclei RNA-seq and small capped RNA-seq data generated using rat brain tissues. Percentage of snRNA-seq reads mapped to A) the genome, B) the transcriptome, and C) an intergenic region of the genome for mRatBN7.2 and Rnor_6.0. D) Overall mapping rates of csRNA-seq data to two reference genomes. E) Unique alignment of csRNA-seq data to two reference genomes.

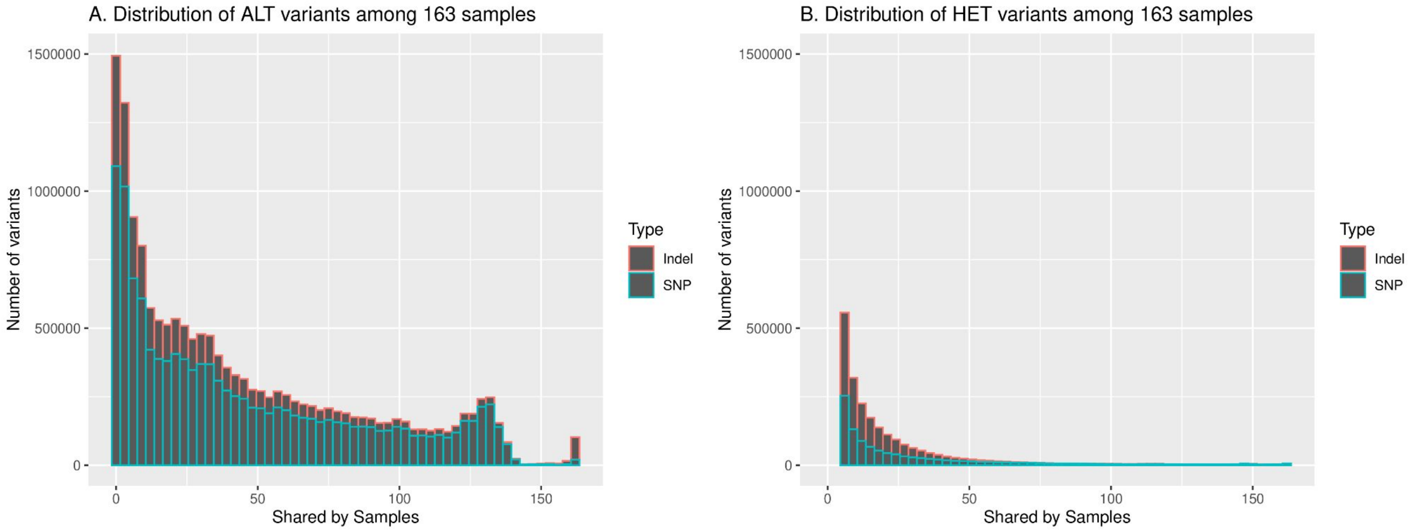

Figure S11 (related to Figure 6). Distribution of the total number of variants shared by 1 or more samples across all 163 samples for A) Homozygous alternative SNPs/Indels. The lack of variants shared by approximately 140-155 samples and the distinct peak after 155 samples indicate variants shared by more than 156 samples are likely caused by errors in the reference genome. B) Heterozygous SNPs/Indels. Similar pattern as A can be observed.

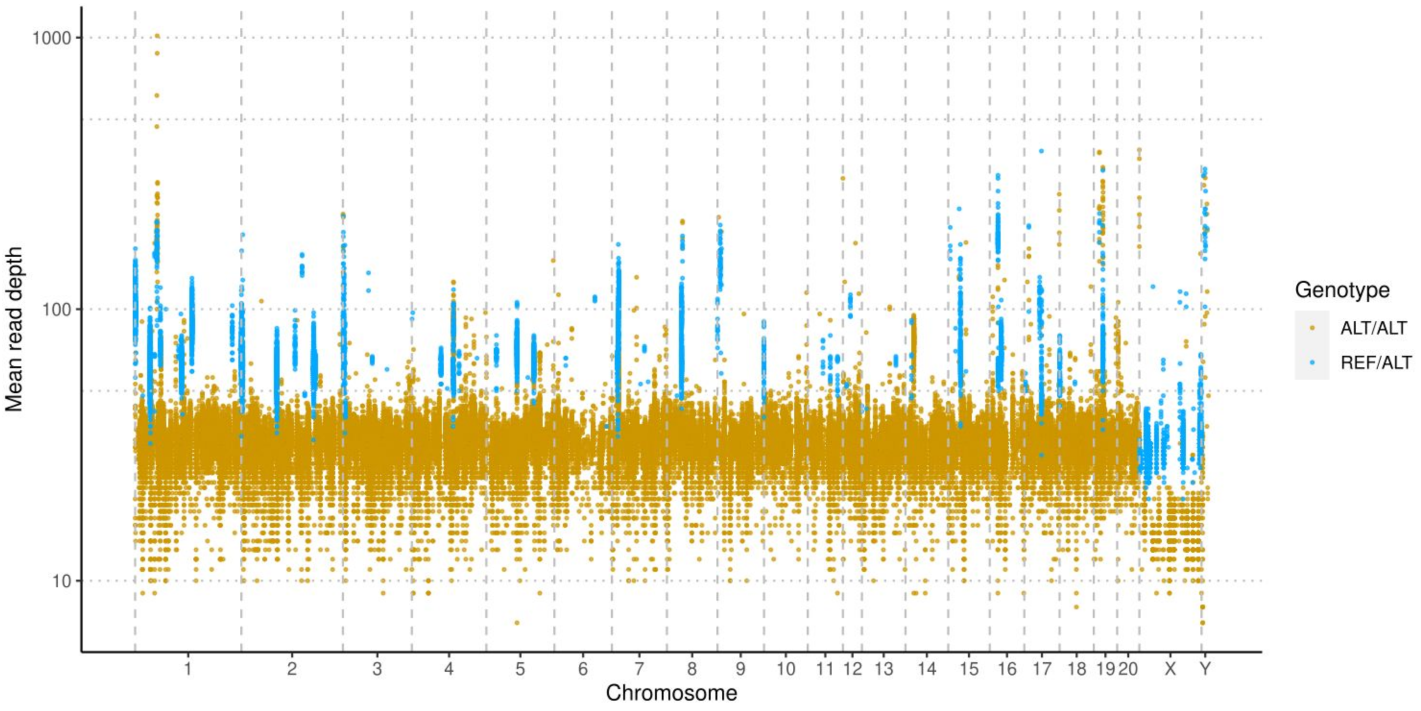

Figure S12 (related to Figure 6). SNPs and indels indicate remaining errors in mRatBN7.2. Base-level errors are indicated by homozygous variants that are shared by the majority (i.e. more than 153 out of 163) of samples, including all seven BN/NHsdMcwi rats, one of them is part of the data used to assemble mRatBN7.2. Variants that are heterozygous for the majority of the samples are clustered in a few regions and have significantly higher read-depth. This suggests that they originated from collapsed repeats in mRatBN7.2.

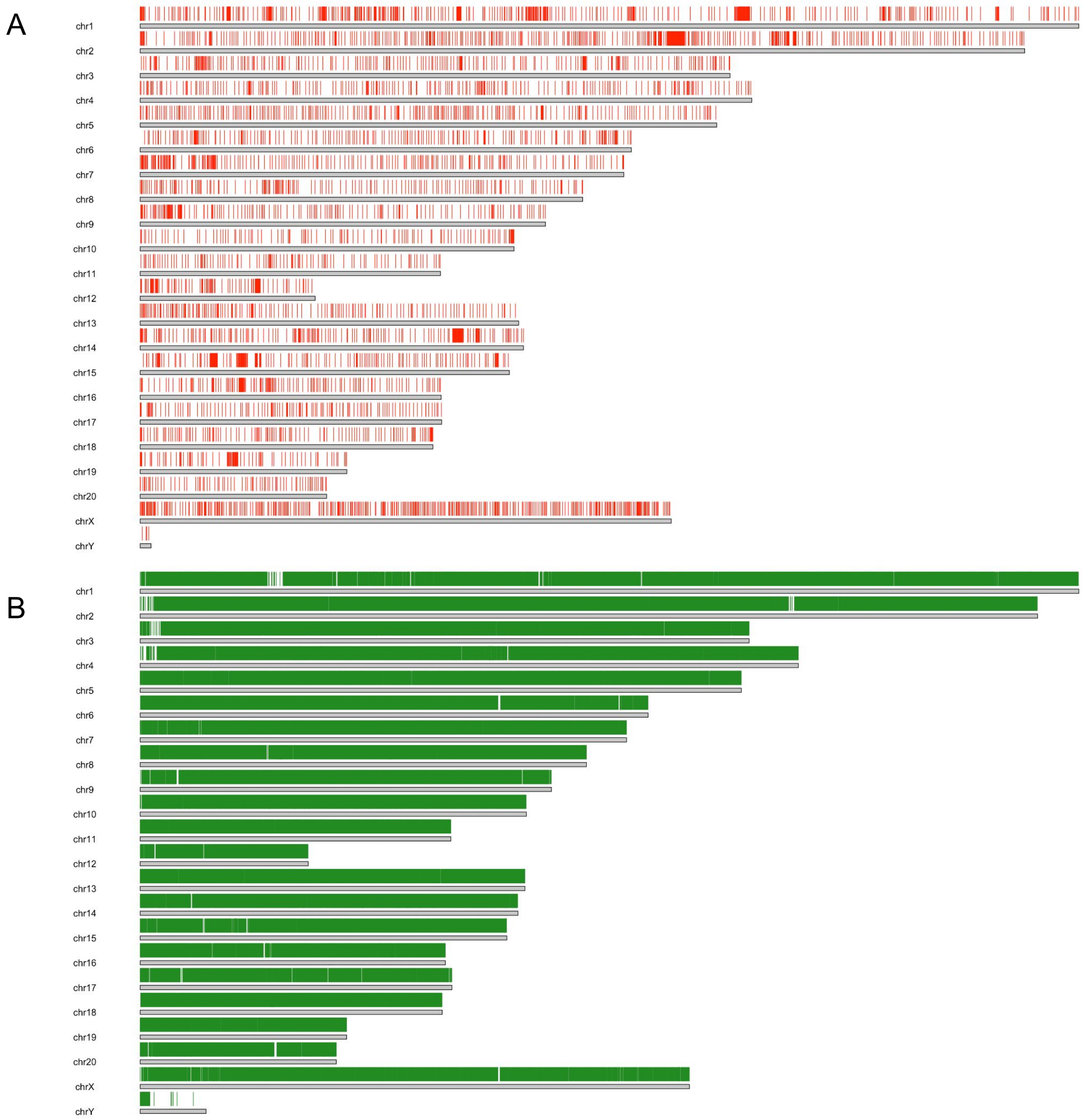

Figure S13 (related to Figure 4). Simulated liftover analysis: unliftable and lifted site distribution. An evenly 1000 bp spaced bed file covering Rnor_6.0 is generated and then lifted to mRatBN7.2. Out of the 2,782,023 sites, 92.04% are liftable, 7.96% are not liftable. A) The distribution of the unliftable sites are plotted on Rnor_6.0. B) The distribution of the lifted sites are plotted on mRatBN7.2. The data are subsampled by 50 for better visualization.

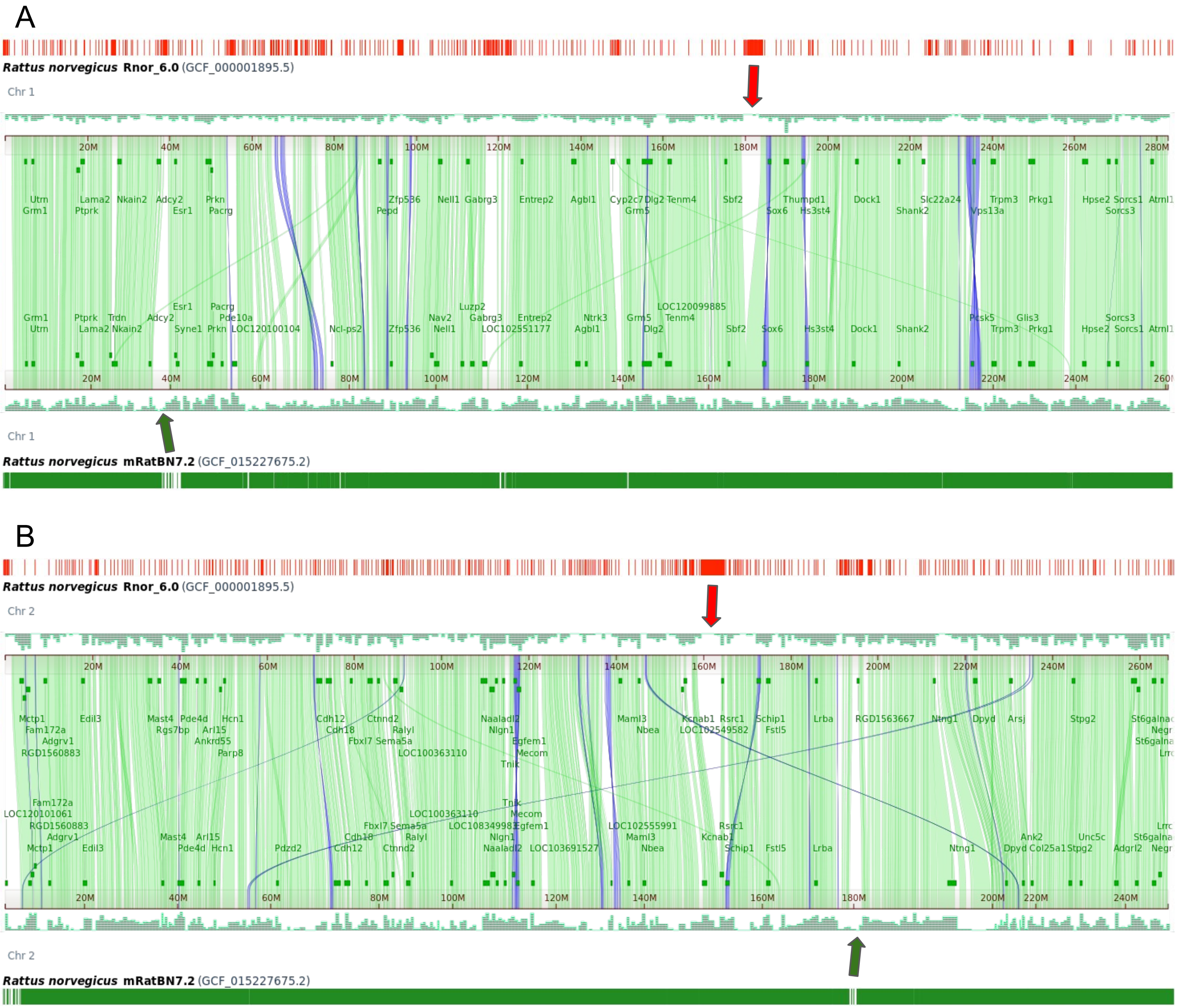

Figure S14 (related to Figure 4). Simulated liftover analysis and comparative genome view. Top red track is the unliftable sites distributed on the Rnor_6.0. Bottom green track is the lifted sites distributed on the mRatBN7.2. The middle is the NCBI comparative genome viewer between chr1 of Rnor_6.0 and mRatBN7.2. The red arrow highlights a region that's not liftable in Rnor_6.0. According to the comparative genome view, the region has no corresponding region in mRatBN7.2 (not just chr1). The green arrow highlights a regiono in mRatBN7.2 that has no lifted sites from Rnor_6.0. According to the comparative genome view, this region has matching region in Rnor_6.0. A) Chromosome 1. B) Chromosome 2.

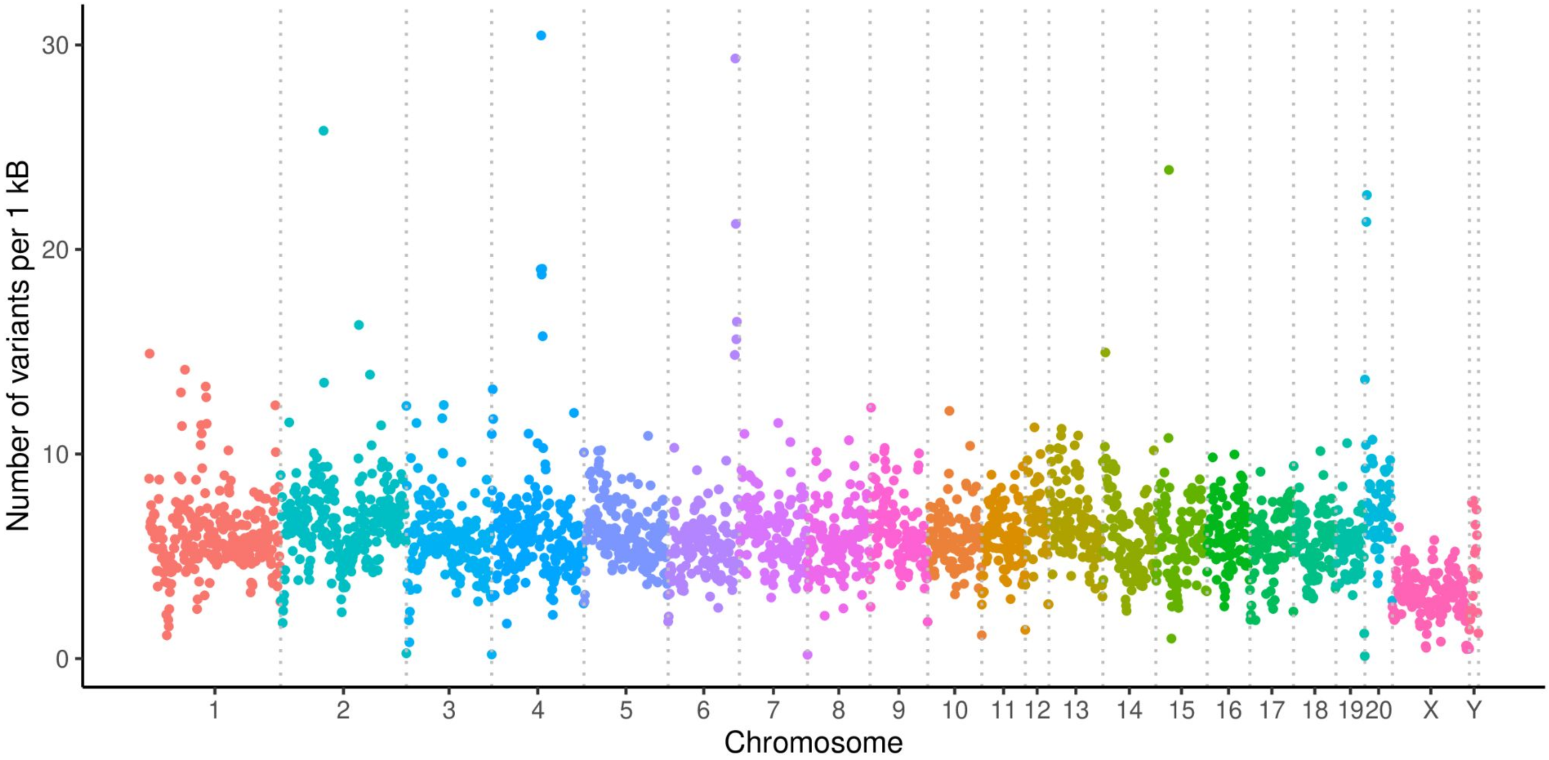

Figure S15 (related to Figure 5). Density of variants across the genome in a collection of 163 rat WGS samples. The mean variant density was 5.96 ± 2.20/Kb (mean ± SD). The highest variant density of 30.5/Kb was found on Chr 4 at 98 Mb.

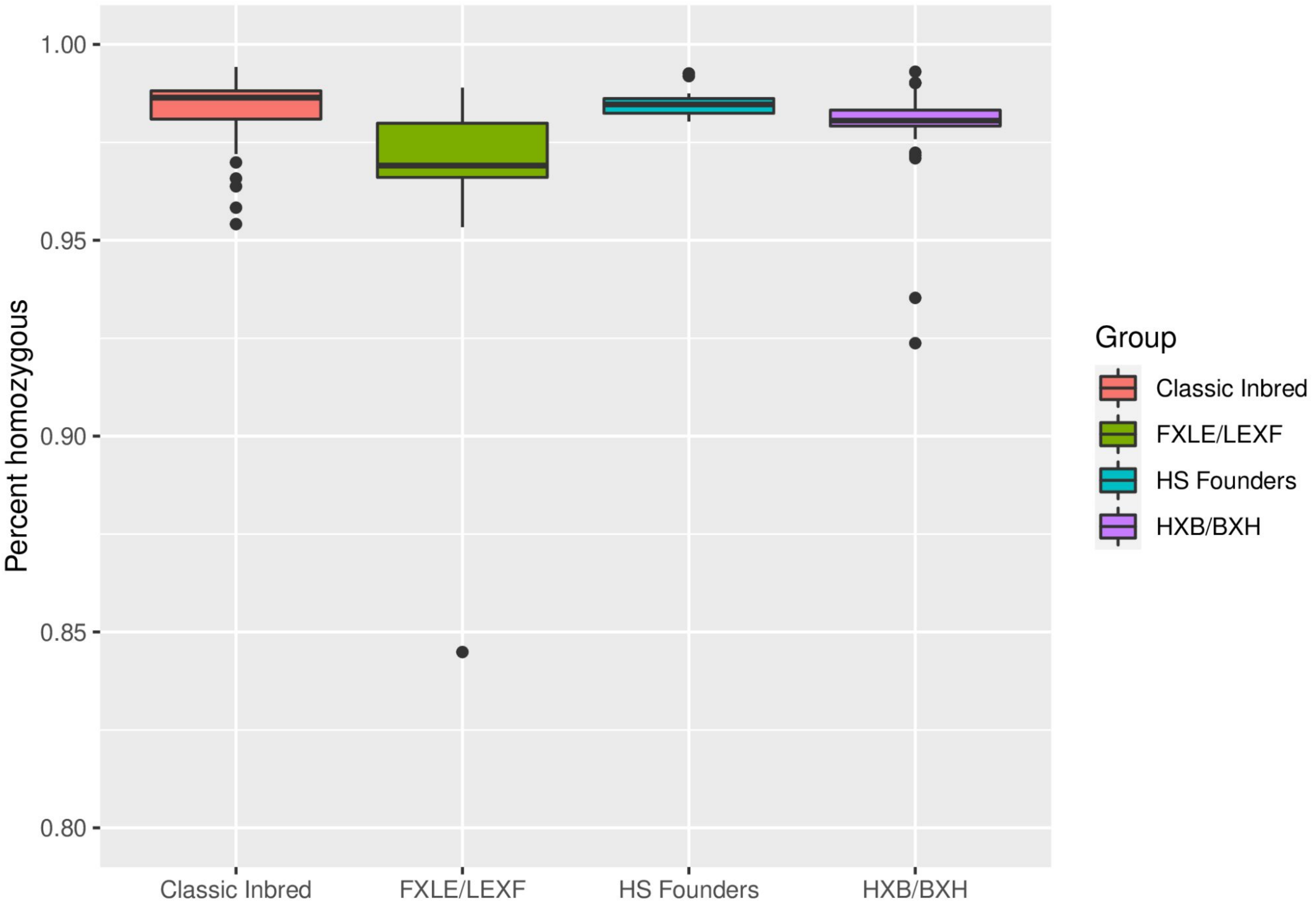

Figure S16 (related to Figure 5). Homozygosity of inbred strains. Variants in most sample were homozygous, confirming the inbred nature of most strains. A few exceptions were noted. For example, 15.6% of the variants from FXLE24 were heterozygous. Additionally, BXH2, which we sequenced two samples, has ~7.7% heterozygous variants.

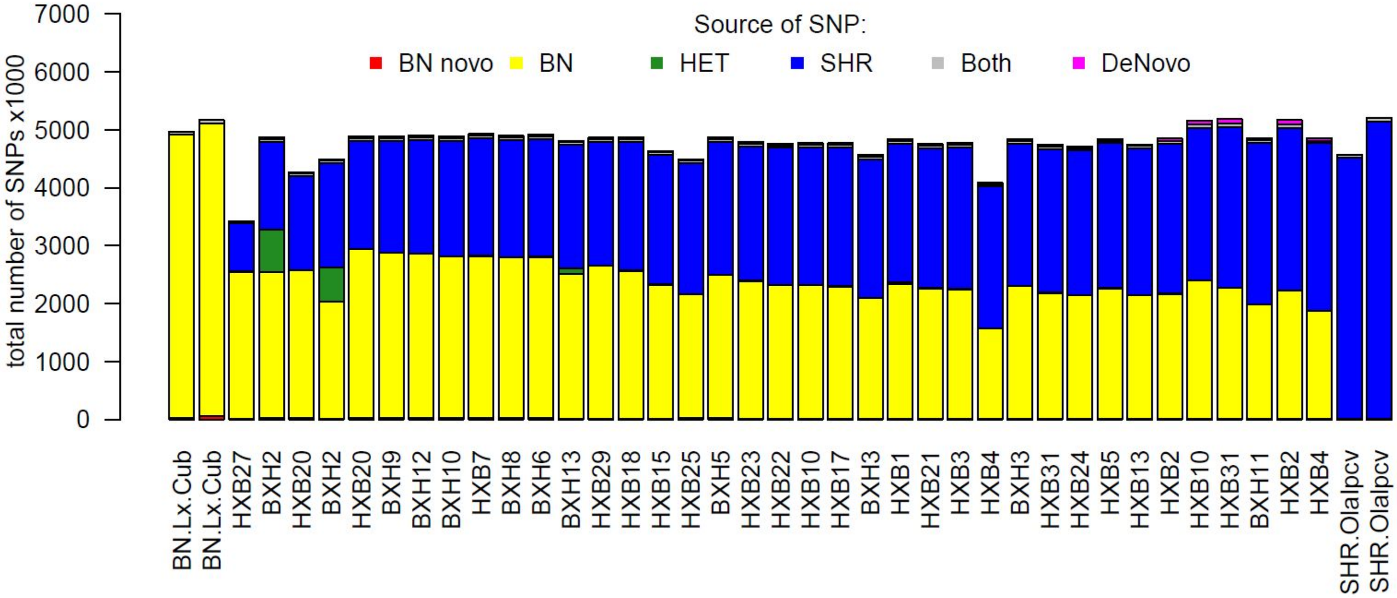

Figure S17 (related to Figure 5). The majority of the variants in HXB/BXH RI panel are inherited from the parental strains. De Novo Mutations on BN.Lx (Red) Mutations originating from BN (Yellow), Heterozygous (Green), Mutations originating from SHR (Blue), SNPs found on both BN and SHR (Grey), De Novo SNPs from neither parental strains (Pink).

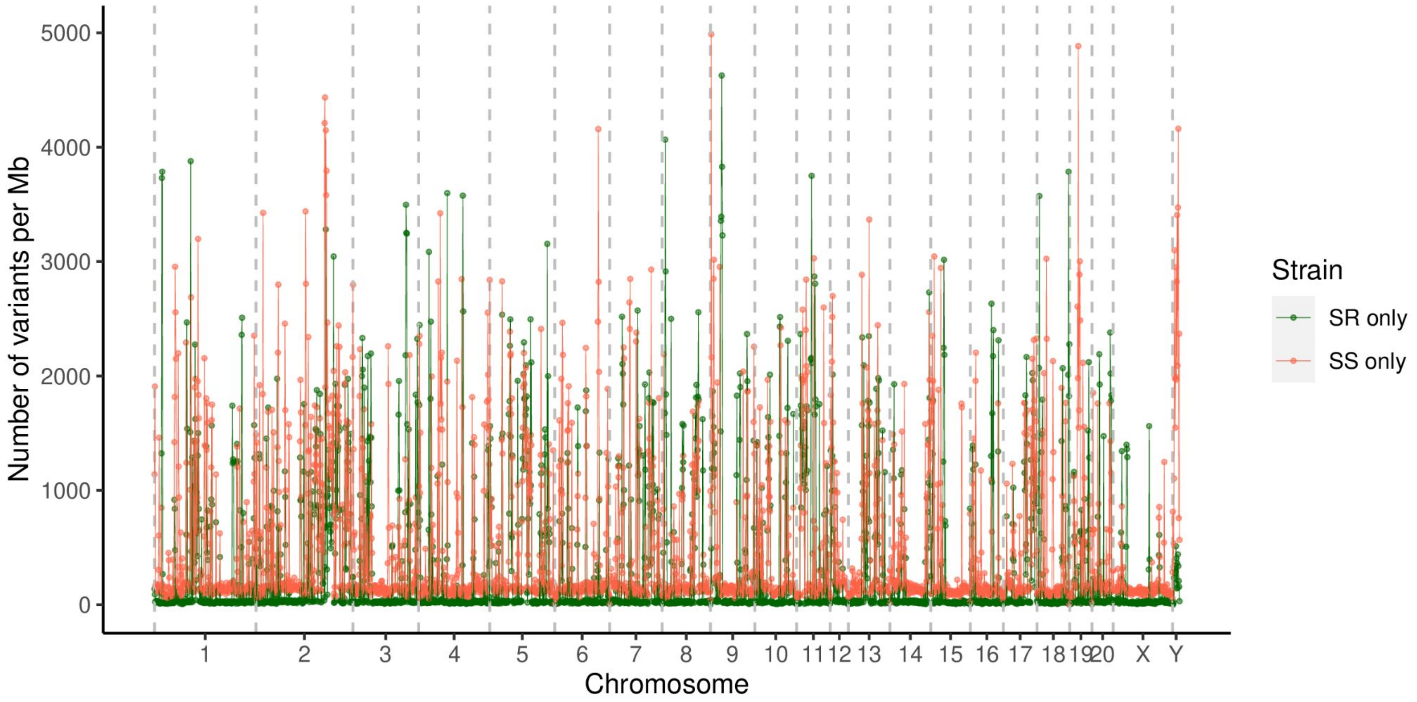

Figure S18 (related to Figure 5). Distribution of variants different between SS and SR rats. Total number of variants unique to either SS or SR rats across the genome. The SS strain contains more unique variants than SR.

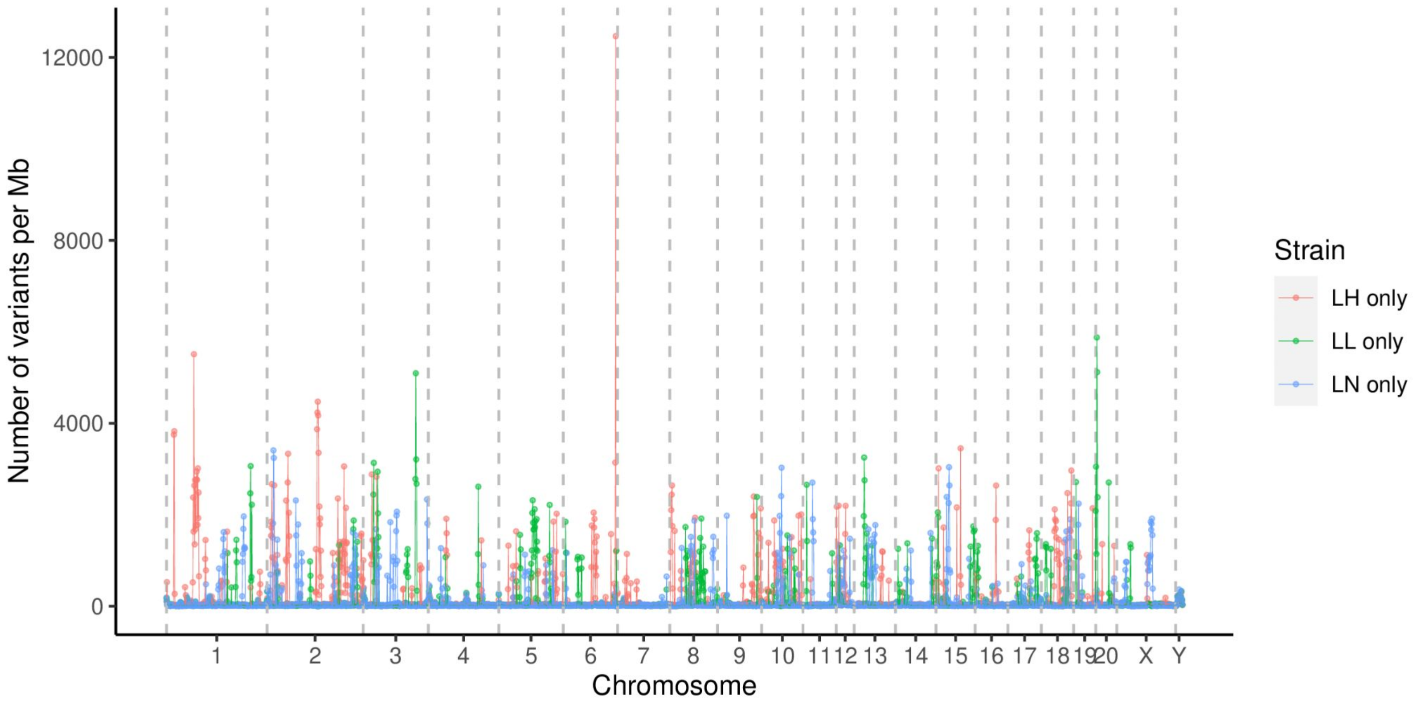

Figure S19 (related to Figure 5). Distribution of variants unique to LL/LH/LN rats. Strain-specific variants tend to cluster by strain.

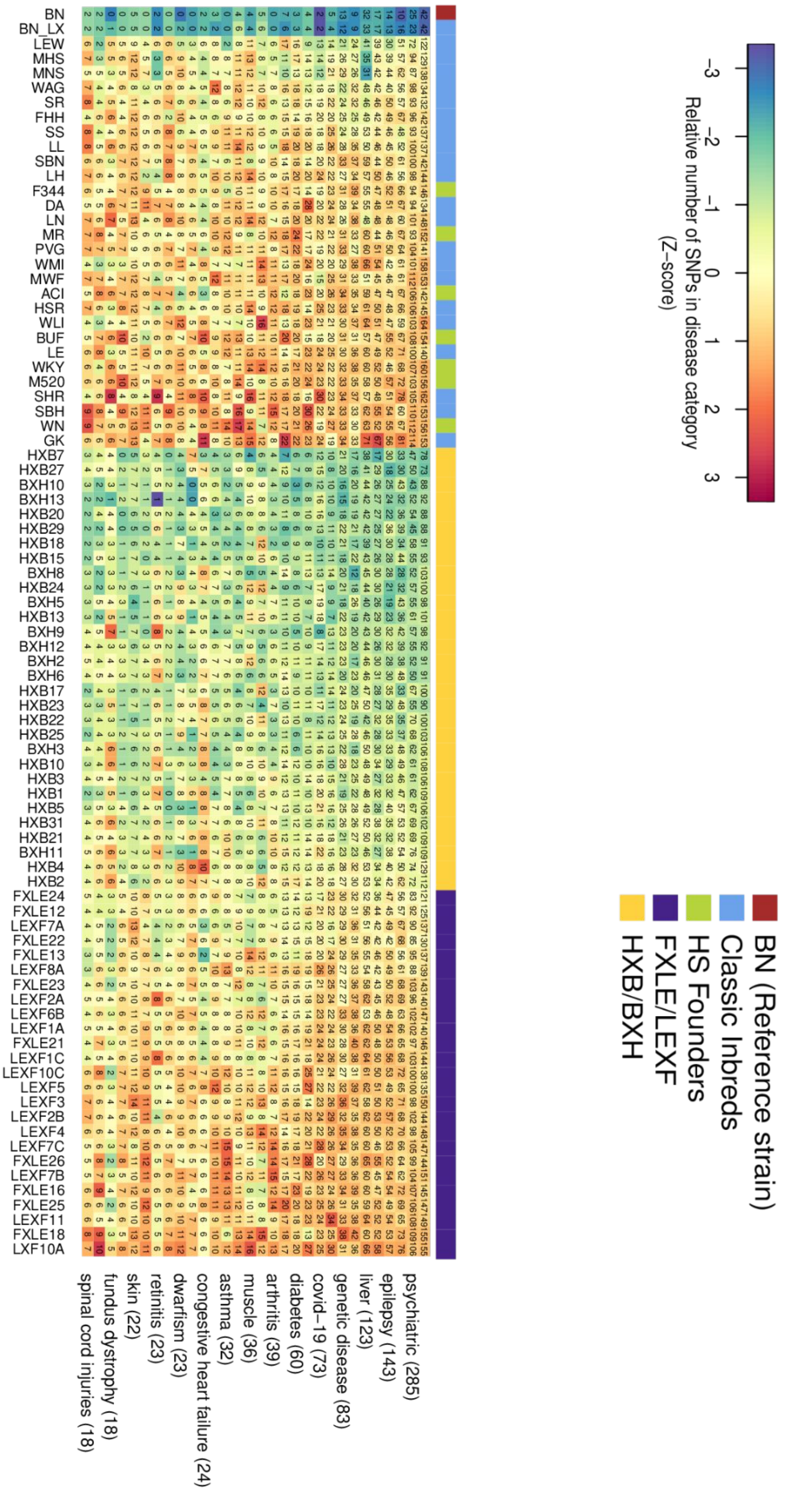

Figure S20 (related to Figure 5). Overview of disease ontology annotation of variants in RatCollection. Heat maps shows relative number of SNPs in proximity of genes associated per disease category within the reference, classic inbreds, HS progenitors and recombinant inbred panels.

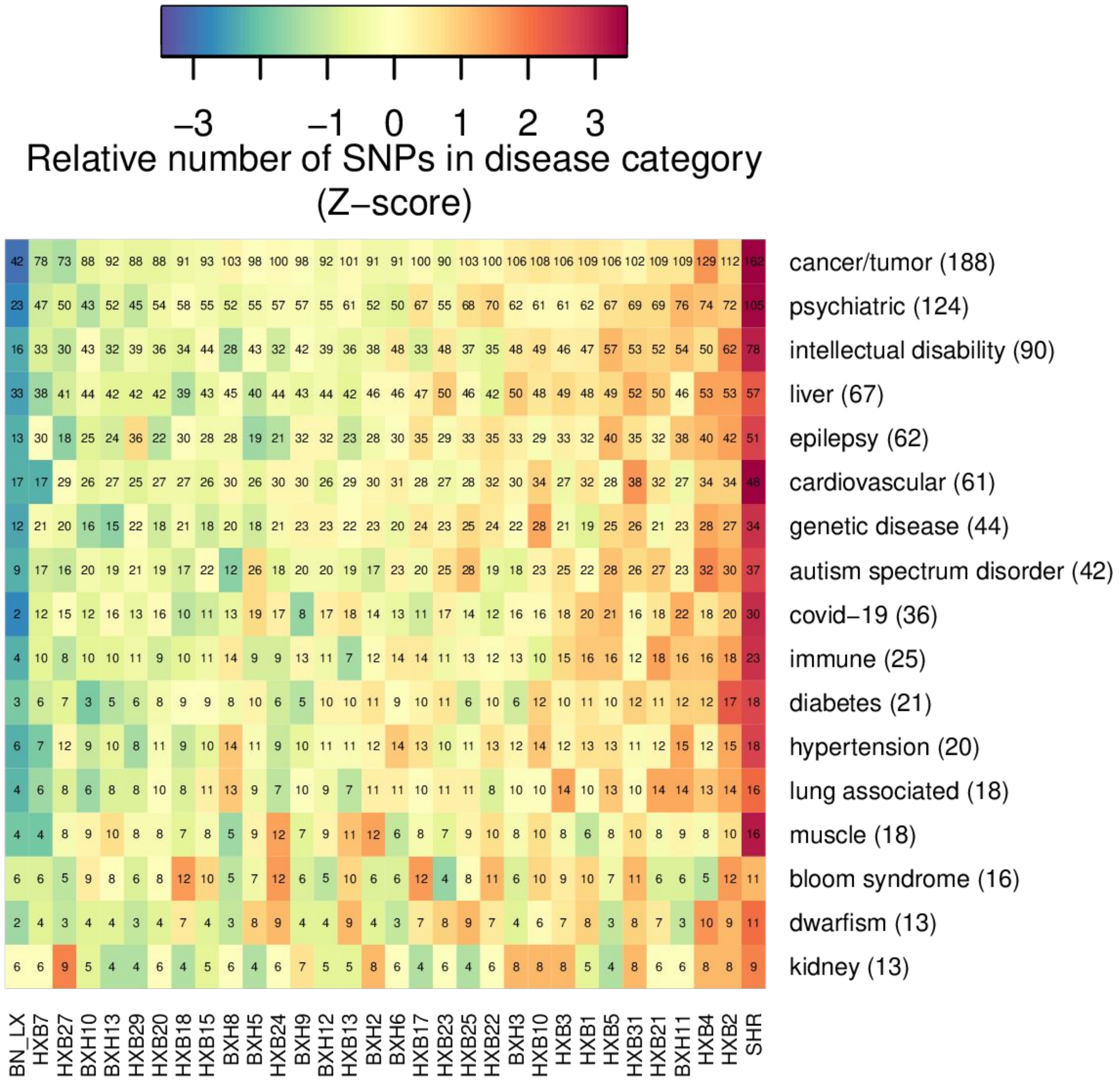

Figure S21 (related to Figure 5). Disease ontology of genetic variants found in the HXB/BXH RI panel. Numbers within the grid show the absolute number of SNPs with high impact on genes within the disease annotation. Numbers following the disease name shows the total number of annotated genes with at least 1 high impact variant within the panel.

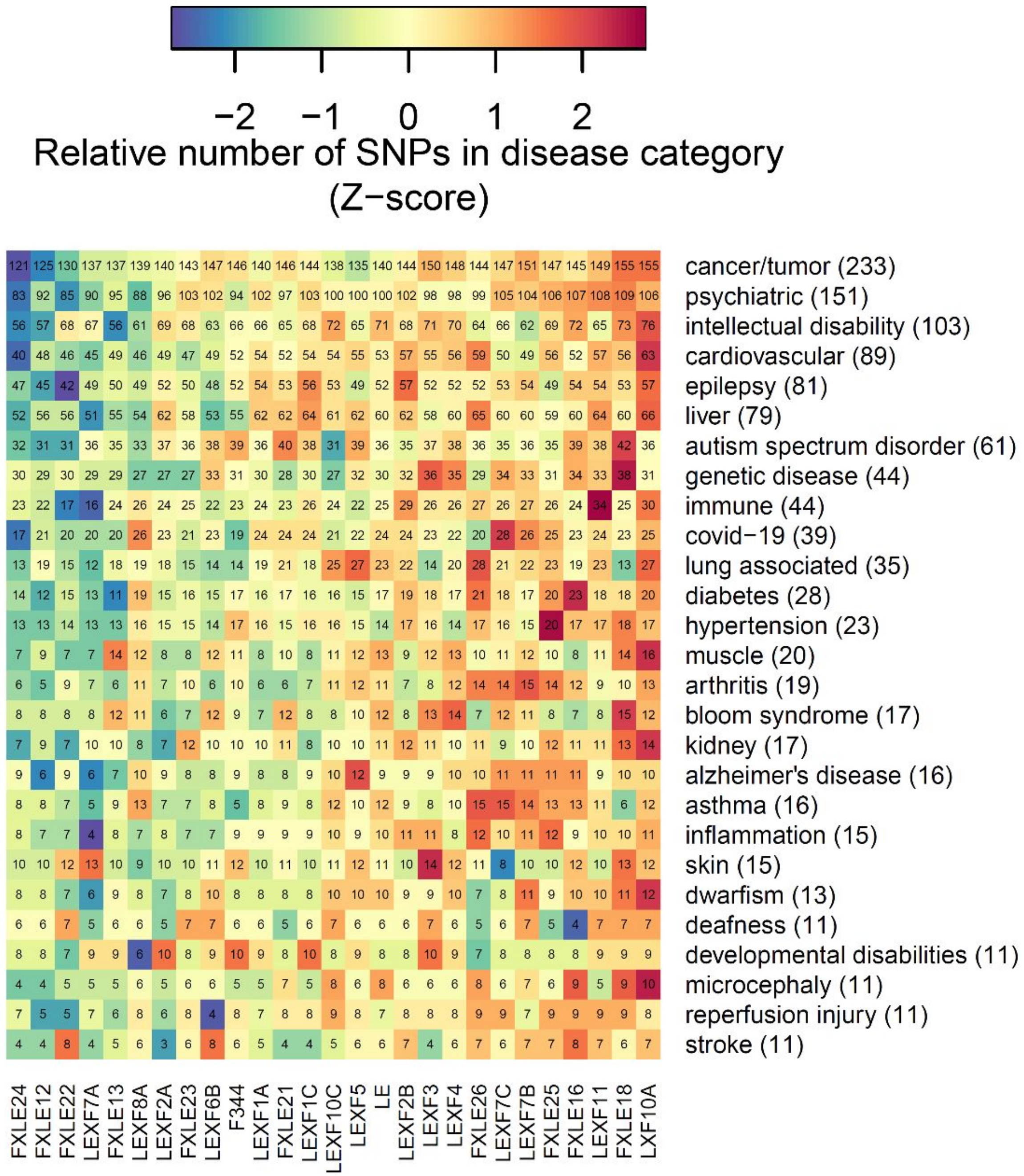

Figure S22 (related to Figure 5). Disease ontology annotation of variants in the LEXF/FXLE RI panel. Numbers within the grid show the absolute number of SNPs with high impact on genes within the disease annotation. Numbers following the disease name shows the total number of annotated genes with at least 1 high impact variant within the panel.

Supplemental references in Excel files

[1] L. Wu, W. Shi, J. Long, X. Guo, K. Michailidou, J. Beesley, MK. Bolla, XO. Shu, Y. Lu, Q. Cai, F. Al-Ejeh, E. Rozali, Q. Wang, J. Dennis, B. Li, C. Zeng, H. Feng, A. Gusev, RT. Barfield, IL. Andrulis, H. Anton-Culver, V. Arndt, KJ. Aronson, PL. Auer, M. Barrdahl, C. Baynes, MW. Beckmann, J. Benitez, M. Bermisheva, C. Blomqvist, NV. Bogdanova, SE. Bojesen, H. Brauch, H. Brenner, L. Brinton, P. Broberg, SY. Brucker, B. Burwinkel, T. Caldés, F. Canzian, BD. Carter, JE. Castelao, J. Chang-Claude, X. Chen, TD. Cheng, H. Christiansen, CL. Clarke, M. Collée, S. Cornelissen, FJ. Couch, D. Cox, A. Cox, SS. Cross, JM. Cunningham, K. Czene, MB. Daly, P. Devilee, KF. Doheny, T. Dörk, I. Dos-Santos-Silva, M. Dumont, M. Dwek, DM. Eccles, U. Eilber, AH. Eliassen, C. Engel, M. Eriksson, L. Fachal, PA. Fasching, J. Figueroa, D. Flesch-Janys, O. Fletcher, H. Flyger, L. Fritschi, M. Gabrielson, M. Gago-Dominguez, SM. Gapstur, M. García-Closas, MM. Gaudet, M. Ghoussaini, GG. Giles, MS. Goldberg, DE. Goldgar, A. González-Neira, P. Guénel, E. Hahnen, CA. Haiman, N. Håkansson, P. Hall, E. Hallberg, U. Hamann, P. Harrington, A. Hein, B. Hicks, P. Hillemanns, A. Hollestelle, RN. Hoover, JL. Hopper, G. Huang, K. Humphreys, DJ. Hunter, A. Jakubowska, W. Janni, EM. John, N. Johnson, K. Jones, ME. Jones, A. Jung, R. Kaaks, MJ. Kerin, E. Khusnutdinova, VM. Kosma, VN. Kristensen, D. Lambrechts, L. Le Marchand, J. Li, S. Lindström, J. Lissowska, WY. Lo, S. Loibl, J. Lubinski, C. Luccarini, MP. Lux, RJ. MacInnis, T. Maishman, IM. Kostovska, A. Mannermaa, JE. Manson, S. Margolin, D. Mavroudis, H. Meijers-Heijboer, A. Meindl, U. Menon, J. Meyer, AM. Mulligan, SL. Neuhausen, H. Nevanlinna, P. Neven, SF. Nielsen, BG. Nordestgaard, OI. Olopade, JE. Olson, H. Olsson, P. Peterlongo, J. Peto, D. Plaseska-Karanfilska, R. Prentice, N. Presneau, K. Pylkäs, B. Rack, P. Radice, N. Rahman, G. Rennert, HS. Rennert, V. Rhenius, A. Romero, J. Romm, A. Rudolph, E. Saloustros, DP. Sandler, EJ. Sawyer, MK. Schmidt, RK. Schmutzler, A. Schneeweiss, RJ. Scott, CG. Scott, S. Seal, M. Shah, MJ. Shrubsole, A. Smeets, MC. Southey, JJ. Spinelli, J. Stone, H. Surowy, AJ. Swerdlow, RM. Tamimi, W. Tapper, JA. Taylor, MB. Terry, DC. Tessier, A. Thomas, K. Thöne, RAEM. Tollenaar, D. Torres, T. Truong, M. Untch, C. Vachon, D. Van Den Berg, D. Vincent, Q. Waisfisz, CR. Weinberg, C. Wendt, AS. Whittemore, H. Wildiers, WC. Willett, R. Winqvist, A. Wolk, L. Xia, XR. Yang, A. Ziogas, E. Ziv, AM. Dunning, PDP. Pharoah, J. Simard, RL. Milne, SL. Edwards, P. Kraft, DF. Easton, G. Chenevix-Trench, and W. Zheng. A transcriptome-wide association study of 229,000 women identifies new candidate susceptibility genes for breast cancer. Nat Genet, 50(7):968–978, 2018. PMCID: PMC6314198, PMID: 29915430.

[2] PA. Northcott, Y. Nakahara, X. Wu, L. Feuk, DW. Ellison, S. Croul, S. Mack, PN. Kongkham, J. Peacock, A. Dubuc, YS. Ra, K. Zilberberg, J. McLeod, SW. Scherer, J. Sunil Rao, CG. Eberhart, W. Grajkowska, Y. Gillespie, B. Lach, R. Grundy, IF. Pollack, RL. Hamilton, T. Van Meter, CG. Carlotti, F. Boop, D. Bigner, RJ. Gilbertson, JT. Rutka, and MD. Taylor. Multiple recurrent genetic events converge on control of histone lysine methylation in medulloblastoma. Nat Genet, 41(4):465–472, 2009. PMCID: PMC4454371, PMID: 19270706.

[3] H. Guo, MH. Duyzend, BP. Coe, C. Baker, K. Hoekzema, J. Gerdts, TN. Turner, MC. Zody, JS. Beighley, SC. Murali, BJ. Nelson, MJ. Bamshad, DA. Nickerson, RA. Bernier, and EE. Eichler. Genome sequencing identifies multiple deleterious variants in autism

patients with more severe phenotypes. Genet Med, 21(7):1611–1620, 2019. PMCID: PMC6546556, PMID: 30504930.

[4] EA. Stahl, G. Breen, AJ. Forstner, A. McQuillin, S. Ripke, V. Trubetskoy, M. Mattheisen, Y. Wang, JRI. Coleman, HA. Gaspar, CA. de Leeuw, S. Steinberg, JMW. Pavlides, M. Trzaskowski, EM. Byrne, TH. Pers, PA. Holmans, AL. Richards, L. Abbott, E. Agerbo, H. Akil, D. Albani, N. Alliey-Rodriguez, TD. Als, A. Anjorin, V. Antilla, S. Awasthi, JA. Badner, M. Bækvad-Hansen, JD. Barchas, N. Bass, M. Bauer, R. Belliveau, SE. Bergen, CB. Pedersen, E. Bøen, MP. Boks, J. Boocock, M. Budde, W. Bunney, M. Burmeister, J. Bybjerg-Grauholm, W. Byerley, M. Casas, F. Cerrato, P. Cervantes, K. Chambert, AW. Charney, D. Chen, C. Churchhouse, TK. Clarke, W. Coryell, DW. Craig, C. Cruceanu, D. Curtis, PM. Czerski, AM. Dale, S. de Jong, F. Degenhardt, J. Del-Favero, JR. DePaulo, S. Djurovic, AL. Dobbyn, A. Dumont, T. Elvsåshagen, V. Escott-Price, CC. Fan, SB. Fischer, M. Flickinger, TM. Foroud, L. Forty, J. Frank, C. Fraser, NB. Freimer, L. Frisén, K. Gade, D. Gage, J. Garnham, C. Giambartolomei, MG. Pedersen, J. Goldstein, SD. Gordon, K. Gordon-Smith, EK. Green, MJ. Green, TA. Greenwood, J. Grove, W. Guan, J. Guzman-Parra, ML. Hamshere, M. Hautzinger, U. Heilbronner, S. Herms, M. Hipolito, P. Hoffmann, D. Holland, L. Huckins, S. Jamain, JS. Johnson, A. Juréus, R. Kandaswamy, R. Karlsson, JL. Kennedy, S. Kittel-Schneider, JA. Knowles, M. Kogevinas, AC. Koller, R. Kupka, C. Lavebratt, J. Lawrence, WB. Lawson, M. Leber, PH. Lee, SE. Levy, JZ. Li, C. Liu, S. Lucae, A. Maaser, DJ. MacIntyre, PB. Mahon, W. Maier, L. Martinsson, S. McCarroll, P. McGuffin, MG. McInnis, JD. McKay, H. Medeiros, SE. Medland, F. Meng, L. Milani, GW. Montgomery, DW. Morris, TW. Mühleisen, N. Mullins, H. Nguyen, CM. Nievergelt, AN. Adolfsson, EA. Nwulia, C. O'Donovan, LMO. Loohuis, APS. Ori, L. Oruc, U. Ösby, RH. Perlis, A. Perry, A. Pfennig, JB. Potash, SM. Purcell, EJ. Regeer, A. Reif, CS. Reinbold, JP. Rice, F. Rivas, M. Rivera, P. Roussos, DM. Ruderfer, E. Ryu, C. Sánchez-Mora, AF. Schatzberg, WA. Scheftner, NJ. Schork, C. Shannon Weickert, T. Shehktman, PD. Shilling, E. Sigurdsson, C. Slaney, OB. Smeland, JL. Sobell, C. Søholm Hansen, AT. Spijker, D. St Clair, M. Steffens, JS. Strauss, F. Streit, J. Strohmaier, S. Szelinger, RC. Thompson, TE. Thorgeirsson, J. Treutlein, H. Vedder, W. Wang, SJ. Watson, TW. Weickert, SH. Witt, S. Xi, W. Xu, AH. Young, P. Zandi, P. Zhang, S. Zöllner, R. Adolfsson, I. Agartz, M. Alda, L. Backlund, BT. Baune, F. Bellivier, WH. Berrettini, JM. Biernacka, DHR. Blackwood, M. Boehnke, AD. Børglum, A. Corvin, N. Craddock, MJ. Daly, U. Dannlowski, T. Esko, B. Etain, M. Frye, JM. Fullerton, ES. Gershon, M. Gill, F. Goes, M. Grigoroiu-Serbanescu, J. Hauser, DM. Hougaard, CM. Hultman, I. Jones, LA. Jones, RS. Kahn, G. Kirov, M. Landén, M. Leboyer, CM. Lewis, QS. Li, J. Lissowska, NG. Martin, F. Mayoral, SL. McElroy, AM. McIntosh, FJ. McMahon, I. Melle, A. Metspalu, PB. Mitchell, G. Morken, O. Mors, PB. Mortensen, B. Müller-Myhsok, RM. Myers, BM. Neale, V. Nimgaonkar, M. Nordentoft, MM. Nöthen, MC. O'Donovan, KJ. Oedegaard, MJ. Owen, SA. Paciga, C. Pato, MT. Pato, D. Posthuma, JA. Ramos-Quiroga, M. Ribasés, M. Rietschel, GA. Rouleau, M. Schalling, PR. Schofield, TG. Schulze, A. Serretti, JW. Smoller, H. Stefansson, K. Stefansson, E. Stordal, PF. Sullivan, G. Turecki, AE. Vaaler, E. Vieta, JB. Vincent, T. Werge, JI. Nurnberger, NR. Wray, A. Di Florio, HJ. Edenberg, S. Cichon, RA. Ophoff, LJ. Scott, OA. Andreassen, J. Kelsoe, and P. Sklar. Genome-wide association study identifies 30 loci associated with bipolar disorder. Nat Genet, 51(5):793–803, 2019. PMCID: PMC6956732, PMID: 31043756.

[5] S. Richards, N. Aziz, S. Bale, D. Bick, S. Das, J. Gastier-Foster, WW. Grody, M. Hegde, E. Lyon, E. Spector, K. Voelkerding, and HL. Rehm. Standards and guidelines for the

interpretation of sequence variants: a joint consensus recommendation of the american college of medical genetics and genomics and the association for molecular pathology. Genet Med, 17(5):405–424, 2015. PMCID: PMC4544753, PMID: 25741868.

[6] K. Downes, K. Megy, D. Duarte, M. Vries, J. Gebhart, S. Hofer, O. Shamardina, SVV. Deevi, J. Stephens, R. Mapeta, S. Tuna, N. Al Hasso, MW. Besser, N. Cooper, L. Daugherty, N. Gleadall, D. Greene, M. Haimel, H. Martin, S. Papadia, S. Revel-Vilk, S. Sivapalaratnam, E. Symington, W. Thomas, C. Thys, A. Tolios, CJ. Penkett, WH. Ouwehand, S. Abbs, MA. Laffan, E. Turro, I. Simeoni, AD. Mumford, YMC. Henskens, I. Pabinger, K. Gomez, and K. Freson. Diagnostic high-throughput sequencing of 2396 patients with bleeding, thrombotic, and platelet disorders. Blood, 134(23):2082–2091, 2019. PMCID: PMC6993014, PMID: 31064749.

[7] ER. Riggs, EF. Andersen, AM. Cherry, S. Kantarci, H. Kearney, A. Patel, G. Raca, DI. Ritter, ST. South, EC. Thorland, D. Pineda-Alvarez, S. Aradhya, and CL. Martin. Technical standards for the interpretation and reporting of constitutional copy-number variants: a joint consensus recommendation of the american college of medical genetics and genomics (acmg) and the clinical genome resource (clingen). Genet Med, 22(2):245–257, 2020. PMCID: PMC7313390, PMID: 31690835.

[8] K. Nykamp, M. Anderson, M. Powers, J. Garcia, B. Herrera, YY. Ho, Y. Kobayashi, N. Patil, J. Thusberg, M. Westbrook, and S. Topper. Sherloc: a comprehensive refinement of the acmg-amp variant classification criteria. Genet Med, 19(10):1105–1117, 2017. PMCID: PMC5632818, PMID: 28492532.

[9] HM. Kearney, EC. Thorland, KK. Brown, F. Quintero-Rivera, and ST. South. American college of medical genetics standards and guidelines for interpretation and reporting of postnatal constitutional copy number variants. Genet Med, 13(7):680–685, 2011. PMID: 21681106.

[10] M. Assoum, C. Philippe, B. Isidor, L. Perrin, P. Makrythanasis, N. Sondheimer, C. Paris, J. Douglas, G. Lesca, S. Antonarakis, H. Hamamy, T. Jouan, Y. Duffourd, S. Auvin, A. Saunier, A. Begtrup, C. Nowak, N. Chatron, D. Ville, K. Mireskandari, P. Milani, P. Jonveaux, G. Lemeur, M. Milh, M. Amamoto, M. Kato, M. Nakashima, N. Miyake, N. Matsumoto, A. Masri, C. Thauvin-Robinet, JB. Rivière, L. Faivre, and J. Thevenon. Autosomal-recessive mutations in ap3b2, adaptor-related protein complex 3 beta 2 subunit, cause an early-onset epileptic encephalopathy with optic atrophy. Am J Hum Genet, 99(6):1368–1376, 2016. PMCID: PMC5142104, PMID: 27889060.

[11] Y. Xiong, Y. Liu, L. Cao, D. Wang, M. Guo, A. Jiang, D. Guo, W. Hu, J. Yang, Z. Tang, H. Wu, Y. Lin, M. Zhang, Q. Zhang, M. Shi, Y. Liu, Y. Zhou, K. Lan, and Y. Chen. Transcriptomic characteristics of bronchoalveolar lavage fluid and peripheral blood mononuclear cells in covid-19 patients. Emerg Microbes Infect, 9(1):761–770, 2020. PMCID: PMC7170362, PMID: 32228226.

[12] CA. Moore, CA. Parkin, Y. Bidet, and PW. Ingham. A role for the myoblast city homologues dock1 and dock5 and the adaptor proteins crk and crk-like in zebrafish myoblast fusion. Development, 134(17):3145–3153, 2007. PMID: 17670792.

[13] JF. Côté and K. Vuori. Gef what? dock180 and related proteins help rac to polarize cells in

new ways. Trends Cell Biol, 17(8):383–393, 2007. PMCID: PMC2887429, PMID: 17765544.

[14] KV. Pajcini, JH. Pomerantz, O. Alkan, R. Doyonnas, and HM. Blau. Myoblasts and macrophages share molecular components that contribute to cell-cell fusion. J Cell Biol, 180(5):1005–1019, 2008. PMCID: PMC2265408, PMID: 18332221.

[15] S. Kumari, V. Borroni, A. Chaudhry, B. Chanda, R. Massol, S. Mayor, and FJ. Barrantes. Nicotinic acetylcholine receptor is internalized via a rac-dependent, dynamin-independent endocytic pathway. J Cell Biol, 181(7): 1179–1193, 2008. PMCID: PMC2442195, PMID: 18591431.

[16] M. Laurin, N. Fradet, A. Blangy, A. Hall, K. Vuori, and JF. Côté. The atypical rac activator dock180 (dock1) regulates myoblast fusion in vivo. Proc Natl Acad Sci U S A, 105(40):15446–15451, 2008. PMCID: PMC2563090, PMID: 18820033.

[17] F. Sanematsu, M. Hirashima, M. Laurin, R. Takii, A. Nishikimi, K. Kitajima, G. Ding, M. Noda, Y. Murata, Y. Tanaka, S. Masuko, T. Suda, C. Meno, JF. Côté, T. Nagasawa, and Y. Fukui. Dock180 is a rac activator that regulates cardiovascular development by acting downstream of cxcr4. Circ Res, 107(9):1102–1105, 2010. PMCID: PMC4640903, PMID: 20829512.

[18] G. Gadea and A. Blangy. Dock-family exchange factors in cell migration and disease. Eur J Cell Biol, 93(10-12): 466–477, 2014. PMID: 25022758.

[19] PT. Hallock, S. Chin, S. Blais, TA. Neubert, and DJ. Glass. Sorbs1 and -2 interact with crkl and are required for acetylcholine receptor cluster formation. Mol Cell Biol, 36(2):262–270, 2016. PMCID: PMC4719301, PMID: 26527617.

[20] V. Ubba, UK. Soni, S. Chadchan, VK. Maurya, V. Kumar, R. Maurya, H. Chaturvedi, R. Singh, A. Dwivedi, and RK. Jha. Rhog-dock1-rac1 signaling axis is perturbed in dhea-induced polycystic ovary in rat model. Reprod Sci, 24(5):738–752, 2017. PMID: 27662902.

[21] A. Entwistle, RJ. Zalin, AE. Warner, and S. Bevan. A role for acetylcholine receptors in the fusion of chick myoblasts. J Cell Biol, 106(5):1703–1712, 1988. PMCID: PMC2115056, PMID: 3372592.

[22] T. Drgon, PW. Zhang, C. Johnson, D. Walther, J. Hess, M. Nino, and GR. Uhl. Genome wide association for addiction: replicated results and comparisons of two analytic approaches. PLoS One, 5(1):e8832, 2010. PMCID: PMC2809089, PMID: 20098672.

[23] O. Baspinar, M. Kilinc, A. Balat, MA. Celkan, and Y. Coskun. Long tortuous aorta in a child with larsen syndrome. Can J Cardiol, 21(3):299–301, 2005. PMID: 15776121.

[24] M. Renard, T. Holm, R. Veith, BL. Callewaert, LC. Adès, O. Baspinar, A. Pickart, M. Dasouki, J. Hoyer, A. Rauch, P. Trapane, MG. Earing, PJ. Coucke, LY. Sakai, HC. Dietz, AM. De Paepe, and BL. Loeys. Altered tgfbeta signaling and cardiovascular manifestations in patients with autosomal recessive cutis laxa type i caused by fibulin-4 deficiency. Eur J Hum Genet, 18(8):895–901, 2010. PMCID: PMC2987390, PMID: 20389311.

[25] V. Mathieu, C. Pirker, WM. Schmidt, S. Spiegl-Kreinecker, D. Lötsch, P. Heffeter, B. Hegedus, M. Grusch, R. Kiss, and W. Berger. Aggressiveness of human melanoma xenograft models is promoted by aneuploidy-driven gene expression deregulation. Oncotarget, 3(4):399–413, 2012. PMCID: PMC3380575, PMID: 22535842.

[26] SK. Lee, MS. Park, and MJ. Nam. Aspirin has antitumor effects via expression of calpain gene in cervical cancer cells. J Oncol, 2008:285374, 2008. PMCID: PMC2648633, PMID: 19266085.

[27] DB. Hoffmann, SK. Williams, J. Bojcevski, A. Müller, C. Stadelmann, V. Naidoo, BA. Bahr, R. Diem, and R. Fairless. Calcium influx and calpain activation mediate preclinical retinal neurodegeneration in autoimmune optic neuritis. J Neuropathol Exp Neurol, 72(8):745–757, 2013. PMID: 23860028.

[28] A. Wessmann, T. Goedde, A. Fischer, P. Wohlsein, H. Hamann, O. Distl, and A. Tipold. Hereditary ataxia in the jack russell terrier–clinical and genetic investigations. J Vet Intern Med, 18(4):515–521, 2004. PMID: 15320590.

[29] M. Cachin and M. Vandevelde. Congenital tremor with spongy degeneration of the central nervous system in two puppies. J Vet Intern Med, 5(2):87–90, 1991. PMID: 2061870.

[30] K. Simpson, S. Eminaga, and GB. Cherubini. Hereditary ataxia in jack russell terriers in the uk. Vet Rec, 170 (21):548, 2012. PMID: 22634896.

[31] A. Vanhaesebrouck, R. Franklin, L. Van Ham, and S. Bhatti. Hereditary ataxia, myokymia and neuromyotonia in jack russell terriers. Vet Rec, 171(5):131–132, 2012. PMID: 22872628.

[32] OP. Forman, L. De Risio, and CS. Mellersh. Missense mutation in capn1 is associated with spinocerebellar ataxia in the parson russell terrier dog breed. PLoS One, 8(5):e64627, 2013. PMCID: PMC3669408, PMID: 23741357.

[33] T. Palmer. Hereditary ataxia in jack russell terriers in the uk. Vet Rec, 174(10):258, 2014. PMID: 24736825.

[34] GB. Cherubini. Hereditary ataxia in jack russell terriers in the uk. Vet Rec, 174(10):258, 2014. PMID: 24736826.

[35] Z. Gan-Or, N. Bouslam, N. Birouk, A. Lissouba, DB. Chambers, J. Vérièpe, A. Androschuk, SB. Laurent, D. Rochefort, D. Spiegelman, A. Dionne-Laporte, A. Szuto, M. Liao, DA. Figlewicz, A. Bouhouche, A. Benomar, M. Yahyaoui, R. Ouazzani, G. Yoon, N. Dupré, O. Suchowersky, FV. Bolduc, JA. Parker, PA. Dion, P. Drapeau, GA. Rouleau, and B. Ouled Amar Bencheikh. Mutations in capn1 cause autosomal-recessive hereditary spastic paraplegia. Am J Hum Genet, 98(5):1038–1046, 2016. PMCID: PMC4863665, PMID: 27153400.

[36] Z. Gan-Or, N. Bouslam, N. Birouk, A. Lissouba, DB. Chambers, J. Vérièpe, A. Androschuk, SB. Laurent, D. Rochefort, D. Spiegelman, A. Dionne-Laporte, A. Szuto, M. Liao, DA. Figlewicz, A. Bouhouche, A. Benomar, M. Yahyaoui, R. Ouazzani, G. Yoon, N. Dupré, O. Suchowersky, FV. Bolduc, JA. Parker, PA. Dion, P. Drapeau, GA. Rouleau, and B. Ouled Amar Bencheikh. Mutations in capn1 cause autosomal-recessive hereditary

spastic paraplegia. Am J Hum Genet, 98(6):1271, 2016. PMCID: PMC4908182, PMID: 27259058.

[37] Y. Wang, J. Hersheson, D. Lopez, M. Hammer, Y. Liu, KH. Lee, V. Pinto, J. Seinfeld, S. Wiethoff, J. Sun, R. Amouri, F. Hentati, N. Baudry, J. Tran, AB. Singleton, M. Coutelier, A. Brice, G. Stevanin, A. Durr, X. Bi, H. Houlden, and M. Baudry. Defects in the capn1 gene result in alterations in cerebellar development and cerebellar ataxia in mice and humans. Cell Rep, 16(1):79–91, 2016. PMCID: PMC4927383, PMID: 27320912.

[38] TW. Lewis and CS. Mellersh. Changes in mutation frequency of eight mendelian inherited disorders in eight pedigree dog populations following introduction of a commercial dna test. PLoS One, 14(1):e0209864, 2019. PMCID: PMC6334900, PMID: 30650096.

[39] WJ. Hartley and AC. Palmer. Ataxia in jack russell terriers. Acta Neuropathol, 26(1):71–74, 1973. PMID: 4747697.

[40] N. Weinhold, A. Jacobsen, N. Schultz, C. Sander, and W. Lee. Genome-wide analysis of noncoding regulatory mutations in cancer. Nat Genet, 46(11):1160–1165, 2014. PMCID: PMC4217527, PMID: 25261935.

[41] E. Castellsagué, S. González, M. Nadal, O. Campos, E. Guinó, M. Urioste, I. Blanco, T. Frebourg, and G. Capellá. Detection of apc gene deletions using quantitative multiplex pcr of short fluorescent fragments. Clin Chem, 54(7):1132–1140, 2008. PMID: 18487285.

[42] M. De Rosa, M. Galatola, S. Borriello, F. Duraturo, S. Masone, and P. Izzo. Implication of adenomatous polyposis coli and mutyh mutations in familial colorectal polyposis. Dis Colon Rectum, 52(2):268–274, 2009. PMID: 19279422.

[43] A. Rohlin, Y. Engwall, K. Fritzell, K. Göransson, A. Bergsten, Z. Einbeigi, M. Nilbert, P. Karlsson, J. Björk, and M. Nordling. Inactivation of promoter 1b of apc causes partial gene silencing: evidence for a significant role of the promoter in regulation and causative of familial adenomatous polyposis. Oncogene, 30(50):4977–4989, 2011. PMCID: PMC3240859, PMID: 21643010.

[44] E. Turro, WJ. Astle, K. Megy, S. Gräf, D. Greene, O. Shamardina, HL. Allen, A. Sanchis-Juan, M. Frontini, C. Thys, J. Stephens, R. Mapeta, OS. Burren, K. Downes, M. Haimel, S. Tuna, SVV. Deevi, TJ. Aitman, DL. Bennett, P. Calleja, K. Carss, MJ. Caulfield, PF. Chinnery, PH. Dixon, DP. Gale, R. James, A. Koziell, MA. Laffan, AP. Levine, ER. Maher, HS. Markus, J. Morales, NW. Morrell, AD. Mumford, E. Ormondroyd, S. Rankin, A. Rendon, S. Richardson, I. Roberts, NBA. Roy, MA. Saleem, KGC. Smith, H. Stark, RYY. Tan, AC. Themistocleous, AJ. Thrasher, H. Watkins, AR. Webster, MR. Wilkins, C. Williamson, J. Whitworth, S. Humphray, DR. Bentley, N. Kingston, N. Walker, JR. Bradley, S. Ashford, CJ. Penkett, K. Freson, KE. Stirrups, FL. Raymond, and WH. Ouwehand. Whole-genome sequencing of patients with rare diseases in a national health system. Nature, 583(7814):96–9102, 2020. PMCID: PMC7610553, PMID: 32581362.

[45] J. Zhang, K. Wang, J. Zhang, SS. Liu, L. Dai, and JY. Zhang. Using proteomic approach to identify tumor-associated proteins as biomarkers in human esophageal squamous cell

carcinoma. J Proteome Res, 10 (6):2863–2872, 2011. PMCID: PMC3119842, PMID: 21517111.

[46] N. Loukili, N. Rosenblatt-Velin, J. Li, S. Clerc, P. Pacher, F. Feihl, B. Waeber, and L. Liaudet. Peroxynitrite induces hmgb1 release by cardiac cells in vitro and hmgb1 upregulation in the infarcted myocardium in vivo. Cardiovasc Res, 89(3):586–594, 2011. PMCID: PMC3028979, PMID: 21113057.

[47] SJ. Schomisch, DG. Murdock, N. Hedayati, JL. Carino, EJ. Lesnefsky, and BL. Cmolik. Cardioplegia prevents ischemia-induced transcriptional alterations of cytoprotective genes in rat hearts: a dna microarray study. J Thorac Cardiovasc Surg, 130(4):1151, 2005. PMID: 16214533.

[48] T. Nishida, M. Tsubota, Y. Kawaishi, H. Yamanishi, N. Kamitani, F. Sekiguchi, H. Ishikura, K. Liu, M. Nishibori, and A. Kawabata. Involvement of high mobility group box 1 in the development and maintenance of chemotherapy-induced peripheral neuropathy in rats. Toxicology, 365:48–58, 2016. PMID: 27474498.

[49] SY. Lin, YY. Wang, WY. Chen, SL. Liao, ST. Chou, CP. Yang, and CJ. Chen. Hepatoprotective activities of rosmarinic acid against extrahepatic cholestasis in rats. Food Chem Toxicol, 108(Pt A):214–223, 2017. PMID: 28789951.

[50] D. Tang, R. Kang, W. Xiao, H. Zhang, MT. Lotze, H. Wang, and X. Xiao. Quercetin prevents lps-induced high-mobility group box 1 release and proinflammatory function. Am J Respir Cell Mol Biol, 41(6):651–660, 2009. PMCID: PMC2784404, PMID: 19265175.

[51] ML. Xu, XJ. Yu, JQ. Zhao, Y. Du, WJ. Xia, Q. Su, MM. Du, Q. Yang, J. Qi, Y. Li, SW. Zhou, GQ. Zhu, HB. Li, and YM. Kang. Calcitriol ameliorated autonomic dysfunction and hypertension by down-regulating inflammation and oxidative stress in the paraventricular nucleus of shr. Toxicol Appl Pharmacol, 394:114950, 2020. PMID: 32147540.

[52] M. Gao, Z. Hu, Y. Zheng, Y. Zeng, X. Shen, D. Zhong, and F. He. Peroxisome proliferator-activated receptor γ agonist troglitazone inhibits high mobility group box 1 expression in endothelial cells via suppressing transcriptional activity of nuclear factor κb and activator protein 1. Shock, 36(3):228–234, 2011. PMID: 21617575.

[53] TH. Kim, SK. Ku, and JS. Bae. Inhibitory effects of kaempferol-3-o-sophoroside on hmgb1-mediated proinflammatory responses. Food Chem Toxicol, 50(3-4):1118–1123, 2012. PMID: 22178603.

[54] EJ. Yang, W. Lee, SK. Ku, KS. Song, and JS. Bae. Anti-inflammatory activities of oleanolic acid on hmgb1 activated huvecs. Food Chem Toxicol, 50(5):1288–1294, 2012. PMID: 22386814.

[55] W. Lee, SK. Ku, JW. Bae, and JS. Bae. Inhibitory effects of lycopene on hmgb1-mediated pro-inflammatory responses in both cellular and animal models. Food Chem Toxicol, 50(6):1826–1833, 2012. PMID: 22429818.

[56] W. Lee, SK. Ku, TH. Kim, and JS. Bae. Emodin-6-o-β-d-glucoside inhibits hmgb1-induced inflammatory responses in vitro and in vivo. Food Chem Toxicol, 52:97–9104, 2013.

PMID: 23146691.

[57] M. Zhao, Y. Feng, J. Xiao, J. Liang, Y. Yin, and D. Chen. Sodium tanshinone iia sulfonate prevents hypoxic trophoblast-induced endothelial cell dysfunction via targeting hmgb1 release. J Biochem Mol Toxicol, 31(7), 2017. PMID: 28294475.

[58] DJ. Antoine, DP. Williams, A. Kipar, RE. Jenkins, SL. Regan, JG. Sathish, NR. Kitteringham, and BK. Park. High-mobility group box-1 protein and keratin-18, circulating serum proteins informative of acetaminophen-induced necrosis and apoptosis in vivo. Toxicol Sci, 112(2):521–531, 2009. PMID: 19783637.

[59] W. Zhang, M. Zhang, Z. Wang, Y. Cheng, H. Liu, Z. Zhou, B. Han, B. Chen, H. Yao, and J. Chao. Neogambogic acid prevents silica-induced fibrosis via inhibition of high-mobility group box 1 and mcp-1-induced protein 1. Toxicol Appl Pharmacol, 309:129–140, 2016. PMID: 27616297.

[60] YM. Allette, MR. Due, SM. Wilson, P. Feldman, MS. Ripsch, R. Khanna, and FA. White. Identification of a functional interaction of hmgb1 with receptor for advanced glycation end-products in a model of neuropathic pain. Brain Behav Immun, 42:169–177, 2014. PMCID: PMC4560334, PMID: 25014009.

[61] I. Kushima, B. Aleksic, M. Nakatochi, T. Shimamura, T. Okada, Y. Uno, M. Morikawa, K. Ishizuka, T. Shiino, H. Kimura, Y. Arioka, A. Yoshimi, Y. Takasaki, Y. Yu, Y. Nakamura, M. Yamamoto, T. Iidaka, S. Iritani, T. Inada, N. Ogawa, E. Shishido, Y. Torii, N. Kawano, Y. Omura, T. Yoshikawa, T. Uchiyama, T. Yamamoto, M. Ikeda, R. Hashimoto, H. Yamamori, Y. Yasuda, T. Someya, Y. Watanabe, J. Egawa, A. Nunokawa, M. Itokawa, M. Arai, M. Miyashita, A. Kobori, M. Suzuki, T. Takahashi, M. Usami, M. Kodaira, K. Watanabe, T. Sasaki, H. Kuwabara, M. Tochigi, F. Nishimura, H. Yamasue, Y. Eriguchi, S. Benner, M. Kojima, W. Yassin, T. Munesue, S. Yokoyama, R. Kimura, Y. Funabiki, H. Kosaka, M. Ishitobi, T. Ohmori, S. Numata, T. Yoshikawa, T. Toyota, K. Yamakawa, T. Suzuki, Y. Inoue, K. Nakaoka, YI. Goto, M. Inagaki, N. Hashimoto, I. Kusumi, S. Son, T. Murai, T. Ikegame, N. Okada, K. Kasai, S. Kunimoto, D. Mori, N. Iwata, and N. Ozaki. Comparative analyses of copy-number variation in autism spectrum disorder and schizophrenia reveal etiological overlap and biological insights. Cell Rep, 24(11):2838–2856, 2018. PMID: 30208311.

[62] IM. Campbell, B. Yuan, C. Robberecht, R. Pfundt, P. Szafranski, ME. McEntagart, SC. Nagamani, A. Erez, M. Bartnik, B. Wiśniowiecka-Kowalnik, KS. Plunkett, AN. Pursley, SH. Kang, W. Bi, SR. Lalani, CA. Bacino, M. Vast, K. Marks, M. Patton, P. Olofsson, A. Patel, JA. Veltman, SW. Cheung, CA. Shaw, LE. Vissers, JR. Vermeesch, JR. Lupski, and P. Stankiewicz. Parental somatic mosaicism is underrecognized and influences recurrence risk of genomic disorders. Am J Hum Genet, 95(2):173–182, 2014. PMCID: PMC4129404, PMID: 25087610.

[63] EA. Otto, TW. Hurd, R. Airik, M. Chaki, W. Zhou, C. Stoetzel, SB. Patil, S. Levy, AK. Ghosh, CA. Murga-Zamalloa, J. van Reeuwijk, SJ. Letteboer, L. Sang, RH. Giles, Q. Liu, KL. Coene, A. Estrada-Cuzcano, RW. Collin, HM. McLaughlin, S. Held, JM. Kasanuki, G. Ramaswami, J. Conte, I. Lopez, J. Washburn, J. Macdonald, J. Hu, Y. Yamashita, ER. Maher, LM. Guay-Woodford, HP. Neumann, N. Obermüller, RK. Koenekoop, C. Bergmann, X. Bei, RA. Lewis, N. Katsanis, V. Lopes, DS. Williams, RH.

Lyons, CV. Dang, DA. Brito, MB. Dias, X. Zhang, JD. Cavalcoli, G. Nürnberg, P. Nürnberg, EA. Pierce, PK. Jackson, C. Antignac, S. Saunier, R. Roepman, H. Dollfus, H. Khanna, and F. Hildebrandt. Candidate exome capture identifies mutation of sdccag8 as the cause of a retinal-renal ciliopathy. Nat Genet, 42(10):840–850, 2010. PMCID: PMC2947620, PMID: 20835237.

[64] MA. Enoch, Z. Zhou, M. Kimura, DC. Mash, Q. Yuan, and D. Goldman. Gabaergic gene expression in postmortem hippocampus from alcoholics and cocaine addicts; corresponding findings in alcohol-naïve p and np rats. PLoS One, 7(1):e29369, 2012. PMCID: PMC3258238, PMID: 22253714.

[65] CI. Dixon, TW. Rosahl, and DN. Stephens. Targeted deletion of the gabra2 gene encoding alpha2-subunits of gaba(a) receptors facilitates performance of a conditioned emotional response, and abolishes anxiolytic effects of benzodiazepines and barbiturates. Pharmacol Biochem Behav, 90(1):1–8, 2008. PMID: 18313124.

[66] SH. Fatemi, TJ. Reutiman, TD. Folsom, and PD. Thuras. Gaba(a) receptor downregulation in brains of subjects with autism. J Autism Dev Disord, 39(2):223–230, 2009. PMCID: PMC2697059, PMID: 18821008.

[67] MA. Enoch, CA. Hodgkinson, Q. Yuan, PH. Shen, D. Goldman, and A. Roy. The influence of gabra2, childhood trauma, and their interaction on alcohol, heroin, and cocaine dependence. Biol Psychiatry, 67(1):20–27, 2010. PMCID: PMC2964936, PMID: 19833324.

[68] CI. Dixon, HV. Morris, G. Breen, S. Desrivieres, S. Jugurnauth, RC. Steiner, H. Vallada, C. Guindalini, R. Laranjeira, G. Messas, TW. Rosahl, JR. Atack, DR. Peden, D. Belelli, JJ. Lambert, SL. King, G. Schumann, and DN. Stephens. Cocaine effects on mouse incentive-learning and human addiction are linked to alpha2 subunit-containing gabaa receptors. Proc Natl Acad Sci U S A, 107(5):2289–2294, 2010. PMCID: PMC2836671, PMID: 20133874.

[69] N. Orenstein, H. Goldberg-Stern, R. Straussberg, L. Bazak, M. Weisz Hubshman, N. Kropach, O. Gilad, O. Scheuerman, Y. Dory, D. Kraus, S. Tzur, N. Magal, Y. Kilim, V. Shkalim Zemer, and L. Basel-Salmon. A de novo gabra2 missense mutation in severe early-onset epileptic encephalopathy with a choreiform movement disorder. Eur J Paediatr Neurol, 22(3):516–524, 2018. PMID: 29422393.

[70] M. Argos, MG. Kibriya, F. Parvez, F. Jasmine, M. Rakibuz-Zaman, and H. Ahsan. Gene expression profiles in peripheral lymphocytes by arsenic exposure and skin lesion status in a bangladeshi population. Cancer Epidemiol Biomarkers Prev, 15(7):1367–1375, 2006. PMID: 16835338.

[71] BJ. Andersen, BA. Rosa, J. Kupritz, A. Meite, T. Serge, MI. Hertz, K. Curtis, CL. King, M. Mitreva, PU. Fischer, and GJ. Weil. Systems analysis-based assessment of post-treatment adverse events in lymphatic filariasis. PLoS Negl Trop Dis, 13(9):e0007697, 2019. PMCID: PMC6762072, PMID: 31557154.

[72] W. D'Souza, S. Pradhan, and D. Saranath. Multiple single nucleotide polymorphism analysis and association of specific genotypes in fhit, samd4a, and ankrd17 in indian

patients with oral cancer. Head Neck, 39(8): 1586–1595, 2017. PMID: 28580594.

[73] RA. Spanjaard, KM. Whren, C. Graves, and J. Bhawan. Tumor necrosis factor receptor superfamily member troy is a novel melanoma biomarker and potential therapeutic target. Int J Cancer, 120(6):1304–1310, 2007. PMID: 17187358.

[74] JX. Bei, Y. Li, WH. Jia, BJ. Feng, G. Zhou, LZ. Chen, QS. Feng, HQ. Low, H. Zhang, F. He, ES. Tai, T. Kang, ET. Liu, J. Liu, and YX. Zeng. A genome-wide association study of nasopharyngeal carcinoma identifies three new susceptibility loci. Nat Genet, 42(7):599–603, 2010. PMID: 20512145.

[75] M. Iyoda, KL. Hudkins, TA. Wietecha, MC. Banas, S. Guo, G. Liu, L. Wang, J. Kowalewska, and CE. Alpers. All-trans-retinoic acid aggravates cryoglobulin-associated membranoproliferative glomerulonephritis in mice. Nephrol Dial Transplant, 22(12):3451–3461, 2007. PMID: 17686814.

[76] DG. Torgerson, EJ. Ampleford, GY. Chiu, WJ. Gauderman, CR. Gignoux, PE. Graves, BE. Himes, AM. Levin, RA. Mathias, DB. Hancock, JW. Baurley, C. Eng, DA. Stern, JC. Celedón, N. Rafaels, D. Capurso, DV. Conti, LA. Roth, M. Soto-Quiros, A. Togias, X. Li, RA. Myers, I. Romieu, DJ. Van Den Berg, D. Hu, NN. Hansel, RD. Hernandez, E. Israel, MT. Salam, J. Galanter, PC. Avila, L. Avila, JR. Rodriquez-Santana, R. Chapela, W. Rodriguez-Cintron, GB. Diette, NF. Adkinson, RA. Abel, KD. Ross, M. Shi, MU. Faruque, GM. Dunston, HR. Watson, VJ. Mantese, SC. Ezurum, L. Liang, I. Ruczinski, JG. Ford, S. Huntsman, KF. Chung, H. Vora, X. Li, WJ. Calhoun, M. Castro, JJ. Sienra-Monge, B. del Rio-Navarro, KA. Deichmann, A. Heinzmann, SE. Wenzel, WW. Busse, JE. Gern, RF. Lemanske, TH. Beaty, ER. Bleecker, BA. Raby, DA. Meyers, SJ. London, FD. Gilliland, EG. Burchard, FD. Martinez, ST. Weiss, LK. Williams, KC. Barnes, C. Ober, and DL. Nicolae. Meta-analysis of genome-wide association studies of asthma in ethnically diverse north american populations. Nat Genet, 43(9):887–892, 2011. PMCID: PMC3445408, PMID: 21804549.

[77] M. Li, P. Hener, Z. Zhang, S. Kato, D. Metzger, and P. Chambon. Topical vitamin d3 and low-calcemic analogs induce thymic stromal lymphopoietin in mouse keratinocytes and trigger an atopic dermatitis. Proc Natl Acad Sci U S A, 103(31):11736–11741, 2006. PMCID: PMC1544239, PMID: 16880407.

[78] Z. Zhu, MH. Oh, J. Yu, YJ. Liu, and T. Zheng. The role of tslp in il-13-induced atopic march. Sci Rep, 1:23, 2011. PMCID: PMC3251897, PMID: 22355542.

[79] CS. Hau, N. Kanda, and S. Watanabe. Suppressive effects of antimycotics on thymic stromal lymphopoietin production in human keratinocytes. J Dermatol Sci, 71(3):174–183, 2013. PMID: 23688403.

[80] T. Hidaka, E. Ogawa, EH. Kobayashi, T. Suzuki, R. Funayama, T. Nagashima, T. Fujimura, S. Aiba, K. Nakayama, R. Okuyama, and M. Yamamoto. The aryl hydrocarbon receptor ahr links atopic dermatitis and air pollution via induction of the neurotrophic factor artemin. Nat Immunol, 18(1):64–73, 2017. PMID: 27869817.

[81] B. Chen, W. Wei, L. Ma, B. Yang, RM. Gill, MS. Chua, AJ. Butte, and S. So. Computational discovery of niclosamide ethanolamine, a repurposed drug candidate that reduces

growth of hepatocellular carcinoma cells in vitro and in mice by inhibiting cell division cycle 37 signaling. Gastroenterology, 152(8):2022–2036, 2017. PMCID: PMC5447464, PMID: 28284560.

[82] ME. Rothenberg, JM. Spergel, JD. Sherrill, K. Annaiah, LJ. Martin, A. Cianferoni, L. Gober, C. Kim, J. Glessner, E. Frackelton, K. Thomas, C. Blanchard, C. Liacouras, R. Verma, S. Aceves, MH. Collins, T. Brown-Whitehorn, PE. Putnam, JP. Franciosi, RM. Chiavacci, SF. Grant, JP. Abonia, PM. Sleiman, and H. Hakonarson. Common variants at 5q22 associate with pediatric eosinophilic esophagitis. Nat Genet, 42(4):289–291, 2010. PMCID: PMC3740732, PMID: 20208534.

[83] Y. Wang, Y. Le, W. Zhao, Y. Lin, Y. Wu, C. Yu, J. Xiong, F. Zou, H. Dong, S. Cai, and H. Zhao. Short thymic stromal lymphopoietin attenuates toluene diisocyanate-induced airway inflammation and inhibits high mobility group box 1-receptor for advanced glycation end products and long thymic stromal lymphopoietin expression. Toxicol Sci, 157(2):276–290, 2017. PMID: 28329851.

[84] DF. Gudbjartsson, US. Bjornsdottir, E. Halapi, A. Helgadottir, P. Sulem, GM. Jonsdottir, G. Thorleifsson, H. Helgadottir, V. Steinthorsdottir, H. Stefansson, C. Williams, J. Hui, J. Beilby, NM. Warrington, A. James, LJ. Palmer, GH. Koppelman, A. Heinzmann, M. Krueger, HM. Boezen, A. Wheatley, J. Altmuller, HD. Shin, ST. Uh, HS. Cheong, B. Jonsdottir, D. Gislason, CS. Park, LM. Rasmussen, C. Porsbjerg, JW. Hansen, V. Backer, T. Werge, C. Janson, UB. Jönsson, MC. Ng, J. Chan, WY. So, R. Ma, SH. Shah, CB. Granger, AA. Quyyumi, AI. Levey, V. Vaccarino, MP. Reilly, DJ. Rader, MJ. Williams, AM. van Rij, GT. Jones, E. Trabetti, G. Malerba, PF. Pignatti, A. Boner, L. Pescollderungg, D. Girelli, O. Olivieri, N. Martinelli, BR. Ludviksson, D. Ludviksdottir, GI. Eyjolfsson, D. Arnar, G. Thorgeirsson, K. Deichmann, PJ. Thompson, M. Wjst, IP. Hall, DS. Postma, T. Gislason, J. Gulcher, A. Kong, I. Jonsdottir, U. Thorsteinsdottir, and K. Stefansson. Sequence variants affecting eosinophil numbers associate with asthma and myocardial infarction. Nat Genet, 41(3):342–347, 2009. PMID: 19198610.

[85] X. Shen, H. Ying, Y. Qiu, JS. Park, R. Shyam, ZL. Chi, T. Iwata, and BY. Yue. Processing of optineurin in neuronal cells. J Biol Chem, 286(5):3618–3629, 2011. PMCID: PMC3030366, PMID: 21059646.

[86] S. Monemi, G. Spaeth, A. DaSilva, S. Popinchalk, E. Ilitchev, J. Liebmann, R. Ritch, E. Héon, RP. Crick, A. Child, and M. Sarfarazi. Identification of a novel adult-onset primary open-angle glaucoma (poag) gene on 5q22.1. Hum Mol Genet, 14(6):725–733, 2005. PMID: 15677485.

[87] F. Pasutto, CY. Mardin, K. Michels-Rautenstrauss, BH. Weber, H. Sticht, G. Chavarria-Soley, B. Rautenstrauss, F. Kruse, and A. Reis. Profiling of wdr36 missense variants in german patients with glaucoma. Invest Ophthalmol Vis Sci, 49(1):270–274, 2008. PMID: 18172102.

[88] TK. Footz, JL. Johnson, S. Dubois, N. Boivin, V. Raymond, and MA. Walter. Glaucoma-associated wdr36 variants encode functional defects in a yeast model system. Hum Mol Genet, 18(7):1276–1287, 2009. PMID: 19150991.

[89] JS. Berg, M. Adams, N. Nassar, C. Bizon, K. Lee, CP. Schmitt, KC. Wilhelmsen, and JP.

Evans. An informatics approach to analyzing the incidentalome. Genet Med, 15(1):36–44, 2013. PMCID: PMC3538953, PMID: 22995991.

[90] Y. Freudenberg-Hua, J. Freudenberg, V. Vacic, A. Abhyankar, AK. Emde, D. Ben-Avraham, N. Barzilai, D. Oschwald, E. Christen, J. Koppel, B. Greenwald, RB. Darnell, S. Germer, G. Atzmon, and P. Davies. Disease variants in genomes of 44 centenarians. Mol Genet Genomic Med, 2(5):438–450, 2014. PMCID: PMC4190879, PMID: 25333069.

[91] J. Meletiadis, S. Chanock, and TJ. Walsh. Defining targets for investigating the pharmacogenomics of adverse drug reactions to antifungal agents. Pharmacogenomics, 9(5):561–584, 2008. PMID: 18466103.

[92] MD. AbdulHameed, GJ. Tawa, K. Kumar, DL. Ippolito, JA. Lewis, JD. Stallings, and A. Wallqvist. Systems level analysis and identification of pathways and networks associated with liver fibrosis. PLoS One, 9(11):e112193, 2014. PMCID: PMC4224449, PMID: 25380136.

[93] J. Rae, D. Noack, PG. Heyworth, BA. Ellis, JT. Curnutte, and AR. Cross. Molecular analysis of 9 new families with chronic granulomatous disease caused by mutations in cyba, the gene encoding p22(phox). Blood, 96(3): 1106–1112, 2000. PMID: 10910929.

[94] D. Roos, DB. Kuhns, A. Maddalena, J. Bustamante, C. Kannengiesser, M. de Boer, K. van Leeuwen, MY. Köker, B. Wolach, J. Roesler, HL. Malech, SM. Holland, JI. Gallin, and MJ. Stasia. Hematologically important mutations: the autosomal recessive forms of chronic granulomatous disease (second update). Blood Cells Mol Dis, 44(4):291–299, 2010. PMCID: PMC4568122, PMID: 20167518.

[95] MP. Adam, J. Feldman, GM. Mirzaa, RA. Pagon, SE. Wallace, LJH. Bean, KW. Gripp, A. Amemiya, JW. Leiding, and SM. Holland. 1993. PMCID: PMC2846862, PMID: 22876374.

[96] CF. Yang, FJ. Tsai, SP. Lin, CC. Lee, and JY. Wu. A novel in-frame deletion mutation (c106-111del) identified in a taiwan chinese patient with type iva mucopolysaccharidosis. Hum Mutat, 18(3):254, 2001. PMID: 11524742.

[97] S. Tomatsu, AM. Montaño, T. Nishioka, MA. Gutierrez, OM. Peña, GG. Tranda Firescu, P. Lopez, S. Yamaguchi, A. Noguchi, and T. Orii. Mutation and polymorphism spectrum of the galns gene in mucopolysaccharidosis iva (morquio a). Hum Mutat, 26(6):500–512, 2005. PMID: 16287098.

[98] Z. Wang, W. Zhang, Y. Wang, Y. Meng, L. Su, H. Shi, and S. Huang. Mucopolysaccharidosis iva mutations in chinese patients: 16 novel mutations. J Hum Genet, 55(8):534–540, 2010. PMID: 20574428.

[99] LM. Pollard, JR. Jones, and TC. Wood. Molecular characterization of 355 mucopolysaccharidosis patients reveals 104 novel mutations. J Inherit Metab Dis, 36(2):179–187, 2013. PMID: 22976768.

[100] VC. Dũng, S. Tomatsu, AM. Montaño, G. Gottesman, MB. Bober, W. Mackenzie, M. Maeda, GA. Mitchell, Y. Suzuki, and T. Orii. Mucopolysaccharidosis iva: correlation between genotype, phenotype and keratan sulfate levels. Mol Genet Metab, 110(1-

2):129–138, 2013. PMCID: PMC3779837, PMID: 23876334.

[101] D. He, Y. Huang, Z. Ou, H. Sheng, S. Li, X. Zhao, R. Li, J. Zheng, and L. Liu. Molecular genetic assay of mucopolysaccharidosis iva in south china. Gene, 532(1):46–52, 2013. PMID: 24035930.

[102] J. Ye, HL. Lei, HW. Zhang, WJ. Qiu, LS. Han, Y. Wang, XY. Li, and XF. Gu. [analysis of galns gene mutation in thirty-eight chinese patients with mucopolysaccharidosis type iva]. Zhonghua Er Ke Za Zhi, 51(6):414–419, 2013. PMID: 24120057.

[103] A. Morrone, KL. Tylee, M. Al-Sayed, AC. Brusius-Facchin, A. Caciotti, HJ. Church, MJ. Coll, K. Davidson, MJ. Fietz, L. Gort, M. Hegde, F. Kubaski, L. Lacerda, F. Laranjeira, S. Leistner-Segal, S. Mooney, S. Pajares, L. Pollard, I. Ribeiro, RY. Wang, and N. Miller. Molecular testing of 163 patients with morquio a (mucopolysaccharidosis iva) identifies 39 novel galns mutations. Mol Genet Metab, 112(2):160–170, 2014. PMCID: PMC4203673, PMID: 24726177.

[104] A. Morrone, A. Caciotti, R. Atwood, K. Davidson, C. Du, P. Francis-Lyon, P. Harmatz, M. Mealiffe, S. Mooney, TR. Oron, A. Ryles, KA. Zawadzki, and N. Miller. Morquio a syndrome-associated mutations: a review of alterations in the galns gene and a new locus-specific database. Hum Mutat, 35(11):1271–1279, 2014. PMCID: PMC4238747, PMID: 25137622.

[105] AM. Bidchol, A. Dalal, H. Shah, S. S, S. Nampoothiri, M. Kabra, N. Gupta, S. Danda, K. Gowrishankar, SR. Phadke, S. Kapoor, M. Kamate, IC. Verma, RD. Puri, VH. Sankar, AR. Devi, SJ. Patil, P. Ranganath, SJ. Jain, M. Agarwal, A. Singh, P. Mishra, PM. Tamhankar, PM. Gopinath, HA. Nagarajaram, K. Satyamoorthy, and KM. Girisha. Galns mutations in indian patients with mucopolysaccharidosis iva. Am J Med Genet A, (11): 2793–2801, 2014. PMID: 25252036.

[106] A. Caciotti, R. Tonin, M. Rigoldi, L. Ferri, S. Catarzi, C. Cavicchi, E. Procopio, MA. Donati, A. Ficcadenti, A. Fiumara, R. Barone, L. Garavelli, MD. Rocco, M. Filocamo, D. Antuzzi, M. Scarpa, SD. Mooney, B. Li, A. Skouma, S. Bianca, D. Concolino, R. Casalone, E. Monti, M. Pantaleo, S. Giglio, R. Guerrini, R. Parini, and A. Morrone. Optimizing the molecular diagnosis of galns: novel methods to define and characterize morquio-a syndrome-associated mutations. Hum Mutat, 36(3):357–368, 2015. PMID: 25545067.

[107] J. Xie, J. Pan, D. Guo, W. Pan, R. Li, C. Guo, M. Du, W. Jiang, and Y. Guo. Mutation analysis and pathogenicity identification of mucopolysaccharidosis type iva in 8 south china families. Gene, 686:261–269, 2019. PMID: 30458289.

[108] HY. Leong, NA. Abdul Azize, HB. Chew, WT. Keng, MK. Thong, MKN. Mohd Khalid, LC. Hung, N. Mohamed Zainudin, A. Ramlee, MA. Md Haniffa, Y. Yakob, and LH. Ngu. Clinical, biochemical and genetic profiles of patients with mucopolysaccharidosis type iva (morquio a syndrome) in malaysia: the first national natural history cohort study. Orphanet J Rare Dis, 14(1):143, 2019. PMCID: PMC6570902, PMID: 31200731.

[109] YH. Chien, NC. Lee, PW. Chen, HY. Yeh, MH. Gelb, PC. Chiu, SY. Chu, CH. Lee, AR. Lee, and WL. Hwu. Newborn screening for morquio disease and other lysosomal storage diseases: results from the 8-plex assay for 70,000 newborns. Orphanet J Rare

Dis, 15(1):38, 2020. PMCID: PMC6998831, PMID: 32014045.

[110] A. Zanetti, F. D'Avanzo, M. AlSayed, AC. Brusius-Facchin, YH. Chien, R. Giugliani, E. Izzo, DC. Kasper, HY. Lin, SP. Lin, L. Pollard, A. Singh, R. Tonin, T. Wood, A. Morrone, and R. Tomanin. Molecular basis of mucopolysaccharidosis iva (morquio a syndrome): A review and classification of galns gene variants and reporting of 68 novel variants. Hum Mutat, 42(11):1384–1398, 2021. PMCID: PMC9291100, PMID: 34387910.

[111] T. Ogawa, S. Tomatsu, S. Fukuda, A. Yamagishi, GM. Rezvi, K. Sukegawa, N. Kondo, Y. Suzuki, N. Shimozawa, and T. Orü. Mucopolysaccharidosis iva: screening and identification of mutations of the n-acetylgalactosamine-6-sulfate sulfatase gene. Hum Mol Genet, 4(3):341–349, 1995. PMID: 7795586.

[112] J. Mateu, M. Alzamora, M. Franco, and MJ. Buisán. Ifosfamide extravasation. Ann Pharmacother, 28(11): 1243–1244, 1994. PMID: 7849337.

[113] JD. Smith, AV. Hing, CM. Clarke, NM. Johnson, FA. Perez, SS. Park, JA. Horst, B. Mecham, L. Maves, DA. Nickerson, and ML. Cunningham. Exome sequencing identifies a recurrent de novo zswim6 mutation associated with acromelic frontonasal dysostosis. Am J Hum Genet, 95(2):235–240, 2014. PMCID: PMC4129399, PMID: 25105228.

[114] SR. Twigg, LB. Ousager, KA. Miller, Y. Zhou, SC. Elalaoui, A. Sefiani, GS. Bak, H. Hove, LK. Hansen, CR. Fagerberg, M. Tajir, and AO. Wilkie. Acromelic frontonasal dysostosis and zswim6 mutation: phenotypic spectrum and mosaicism. Clin Genet, 90(3):270–275, 2016. PMCID: PMC5025718, PMID: 26706854.

[115] J. Zhang, FE. Frerman, and JJ. Kim. Structure of electron transfer flavoprotein-ubiquinone oxidoreductase and electron transfer to the mitochondrial ubiquinone pool. Proc Natl Acad Sci U S A, 103(44):16212–16217, 2006. PMCID: PMC1637562, PMID: 17050691.

[116] SI. Goodman, RJ. Binard, MR. Woontner, and FE. Frerman. Glutaric acidemia type ii: gene structure and mutations of the electron transfer flavoprotein:ubiquinone oxidoreductase (etf:qo) gene. Mol Genet Metab, 77 (1-2):86–90, 2002. PMID: 12359134.

[117] RK. Olsen, M. Pourfarzam, AA. Morris, RC. Dias, I. Knudsen, BS. Andresen, N. Gregersen, and SE. Olpin. Lipid-storage myopathy and respiratory insufficiency due to etfqo mutations in a patient with late-onset multiple acyl-coa dehydrogenation deficiency. J Inherit Metab Dis, 27(5):671–678, 2004. PMID: 15669683.

[118] N. Cornelius, FE. Frerman, TJ. Corydon, J. Palmfeldt, P. Bross, N. Gregersen, and RK. Olsen. Molecular mechanisms of riboflavin responsiveness in patients with etf-qo variations and multiple acyl-coa dehydrogenation deficiency. Hum Mol Genet, 21(15):3435–3448, 2012. PMID: 22611163.

[119] MP. Marrades, P. González-Muniesa, D. Arteta, JA. Martínez, and MJ. Moreno-Aliaga. Orchestrated downregulation of genes involved in oxidative metabolic pathways in obese vs. lean high-fat young male consumers. J Physiol Biochem, 67(1):15–26, 2011. PMID: 20882379.

[120] EB. Kaminsky, V. Kaul, J. Paschall, DM. Church, B. Bunke, D. Kunig, D. Moreno-De-Luca, A. Moreno-De-Luca, JG. Mulle, ST. Warren, G. Richard, JG. Compton, AE. Fuller, TJ.

Gliem, S. Huang, MN. Collinson, SJ. Beal, T. Ackley, DL. Pickering, DM. Golden, E. Aston, H. Whitby, S. Shetty, MR. Rossi, MK. Rudd, ST. South, AR. Brothman, WG. Sanger, RK. Iyer, JA. Crolla, EC. Thorland, S. Aradhya, DH. Ledbetter, and CL. Martin. An evidence-based approach to establish the functional and clinical significance of copy number variants in intellectual and developmental disabilities. Genet Med, 13(9):777–784, 2011. PMCID: PMC3661946, PMID: 21844811.

[121] D. Moreno-De-Luca, SJ. Sanders, AJ. Willsey, JG. Mulle, JK. Lowe, DH. Geschwind, MW. State, CL. Martin, and DH. Ledbetter. Using large clinical data sets to infer pathogenicity for rare copy number variants in autism cohorts. Mol Psychiatry, 18(10):1090–1095, 2013. PMCID: PMC3720840, PMID: 23044707.

[122] VM. Leppa, SN. Kravitz, CL. Martin, J. Andrieux, C. Le Caignec, D. Martin-Coignard, C. DyBuncio, SJ. Sanders, JK. Lowe, RM. Cantor, and DH. Geschwind. Rare inherited and de novo cnvs reveal complex contributions to asd risk in multiplex families. Am J Hum Genet, 99(3):540–554, 2016. PMCID: PMC5011063, PMID: 27569545.

[123] MR. Bekheirnia, N. Bekheirnia, MN. Bainbridge, S. Gu, ZH. Coban Akdemir, T. Gambin, NK. Janzen, SN. Jhangiani, DM. Muzny, M. Michael, ED. Brewer, E. Elenberg, AS. Kale, AA. Riley, SJ. Swartz, DA. Scott, Y. Yang, PR. Srivaths, SE. Wenderfer, J. Bodurtha, CD. Applegate, M. Velinov, A. Myers, L. Borovik, WJ. Craigen, NA. Hanchard, JA. Rosenfeld, RA. Lewis, ET. Gonzales, RA. Gibbs, JW. Belmont, DR. Roth, C. Eng, MC. Braun, JR. Lupski, and DJ. Lamb. Whole-exome sequencing in the molecular diagnosis of individuals with congenital anomalies of the kidney and urinary tract and identification of a new causative gene. Genet Med, 19 (4):412–420, 2017. PMCID: PMC5362362, PMID: 27657687.

[124] CR. Marshall, A. Noor, JB. Vincent, AC. Lionel, L. Feuk, J. Skaug, M. Shago, R. Moessner, D. Pinto, Y. Ren, B. Thiruvahindrapduram, A. Fiebig, S. Schreiber, J. Friedman, CE. Ketelaars, YJ. Vos, C. Ficicioglu, S. Kirkpatrick, R. Nicolson, L. Sloman, A. Summers, CA. Gibbons, A. Teebi, D. Chitayat, R. Weksberg, A. Thompson, C. Vardy, V. Crosbie, S. Luscombe, R. Baatjes, L. Zwaigenbaum, W. Roberts, B. Fernandez, P. Szatmari, and SW. Scherer. Structural variation of chromosomes in autism spectrum disorder. Am J Hum Genet, 82(2):477–488, 2008. PMCID: PMC2426913, PMID: 18252227.

[125] A. Noor, A. Whibley, CR. Marshall, PJ. Gianakopoulos, A. Piton, AR. Carson, M. Orlic-Milacic, AC. Lionel, D. Sato, D. Pinto, I. Drmic, C. Noakes, L. Senman, X. Zhang, R. Mo, J. Gauthier, J. Crosbie, AT. Pagnamenta, J. Munson, AM. Estes, A. Fiebig, A. Franke, S. Schreiber, AF. Stewart, R. Roberts, R. McPherson, SJ. Guter, EH. Cook, G. Dawson, GD. Schellenberg, A. Battaglia, E. Maestrini, L. Jeng, T. Hutchison, E. Rajcan-Separovic, AE. Chudley, SM. Lewis, X. Liu, JJ. Holden, B. Fernandez, L. Zwaigenbaum, SE. Bryson, W. Roberts, P. Szatmari, L. Gallagher, MR. Stratton, J. Gecz, AF. Brady, CE. Schwartz, RJ. Schachar, AP. Monaco, GA. Rouleau, CC. Hui, F. Lucy Raymond, SW. Scherer, and JB. Vincent. Disruption at the ptchd1 locus on xp22.11 in autism spectrum disorder and intellectual disability. Sci Transl Med, 2(49):49ra68, 2010. PMCID: PMC2987731, PMID: 20844286.

[126] MT. Carter, SM. Nikkel, BA. Fernandez, CR. Marshall, A. Noor, AC. Lionel, A. Prasad, D. Pinto, AM. Joseph-George, C. Noakes, C. Fairbrother-Davies, W. Roberts, J. Vincent,

R. Weksberg, and SW. Scherer. Hemizygous deletions on chromosome 1p21.3 involving the dpyd gene in individuals with autism spectrum disorder. Clin Genet, 80(5):435–443, 2011. PMID: 21114665.

[127] AB. Van Kuilenburg, P. Vreken, NG. Abeling, HD. Bakker, R. Meinsma, H. Van Lenthe, RA. De Abreu, JA. Smeitink, H. Kayserili, MY. Apak, E. Christensen, I. Holopainen, K. Pulkki, D. Riva, G. Botteon, E. Holme, M. Tulinius, WJ. Kleijer, FA. Beemer, M. Duran, KE. Niezen-Koning, GP. Smit, C. Jakobs, LM. Smit, and AH. Van Gennip. Genotype and phenotype in patients with dihydropyrimidine dehydrogenase deficiency. Hum Genet, 104(1):1–9, 1999. PMID: 10071185.

[128] P. Jézéquel, MP. Joalland, G. Milano, D. Lanoë, G. Ricolleau, E. Marie-Christine, and R. Deporte-Fety. Common dpyd mutation associated with 5-fluorouracil toxicity detected by pcr-mediated site-directed mutagenesis. Clin Chem, 46(2):309–310, 2000. PMID: 10657402.

[129] ES. Collie-Duguid, MC. Etienne, G. Milano, and HL. McLeod. Known variant dpyd alleles do not explain dpd deficiency in cancer patients. Pharmacogenetics, 10(3):217–223, 2000. PMID: 10803677.

[130] AB. van Kuilenburg, J. Haasjes, DJ. Richel, L. Zoetekouw, H. Van Lenthe, RA. De Abreu, JG. Maring, P. Vreken, and AH. van Gennip. Clinical implications of dihydropyrimidine dehydrogenase (dpd) deficiency in patients with severe 5-fluorouracil-associated toxicity: identification of new mutations in the dpd gene. Clin Cancer Res, 6 (12):4705–4712, 2000. PMID: 11156223.

[131] AB. van Kuilenburg, EW. Muller, J. Haasjes, R. Meinsma, L. Zoetekouw, HR. Waterham, F. Baas, DJ. Richel, and AH. van Gennip. Lethal outcome of a patient with a complete dihydropyrimidine dehydrogenase (dpd) deficiency after administration of 5-fluorouracil: frequency of the common ivs14+1g>a mutation causing dpd deficiency. Clin Cancer Res, 7(5):1149–1153, 2001. PMID: 11350878.

[132] M. Raida, W. Schwabe, P. Häusler, AB. Van Kuilenburg, AH. Van Gennip, D. Behnke, and K. Höffken. Prevalence of a common point mutation in the dihydropyrimidine dehydrogenase (dpd) gene within the 5'-splice donor site of intron 14 in patients with severe 5-fluorouracil (5-fu)- related toxicity compared with controls. Clin Cancer Res, 7(9):2832–2839, 2001. PMID: 11555601.

[133] MR. Johnson, K. Wang, and RB. Diasio. Profound dihydropyrimidine dehydrogenase deficiency resulting from a novel compound heterozygote genotype. Clin Cancer Res, 8(3):768–774, 2002. PMID: 11895907.

[134] JG. Maring, AB. van Kuilenburg, J. Haasjes, H. Piersma, HJ. Groen, DR. Uges, AH. Van Gennip, and EG. De Vries. Reduced 5-fu clearance in a patient with low dpd activity due to heterozygosity for a mutant allele of the dpyd gene. Br J Cancer, 86(7):1028–1033, 2002. PMCID: PMC2364178, PMID: 11953843.

[135] AB. van Kuilenburg, D. Dobritzsch, R. Meinsma, J. Haasjes, HR. Waterham, MJ. Nowaczyk, GD. Maropoulos, G. Hein, H. Kalhoff, JM. Kirk, H. Baaske, A. Aukett, JA. Duley, KP. Ward, Y. Lindqvist, and AH. van Gennip. Novel disease-causing mutations in

the dihydropyrimidine dehydrogenase gene interpreted by analysis of the three-dimensional protein structure. Biochem J, 364(Pt 1):157–163, 2002. PMCID: PMC1222557, PMID: 11988088.

[136] AB. Van Kuilenburg, R. Meinsma, L. Zoetekouw, and AH. Van Gennip. Increased risk of grade iv neutropenia after administration of 5-fluorouracil due to a dihydropyrimidine dehydrogenase deficiency: high prevalence of the ivs14+1g>a mutation. Int J Cancer, 101(3):253–258, 2002. PMID: 12209976.

[137] AB. Van Kuilenburg, R. Meinsma, L. Zoetekouw, and AH. Van Gennip. High prevalence of the ivs14 + 1g>a mutation in the dihydropyrimidine dehydrogenase gene of patients with severe 5-fluorouracil-associated toxicity. Pharmacogenetics, 12(7):555–558, 2002. PMID: 12360106.

[138] H. Ezzeldin, MR. Johnson, Y. Okamoto, and R. Diasio. Denaturing high performance liquid chromatography analysis of the dpyd gene in patients with lethal 5-fluorouracil toxicity. Clin Cancer Res, 9(8):3021–3028, 2003. PMID: 12912951.

[139] N. Salgueiro, I. Veiga, M. Fragoso, O. Sousa, N. Costa, ML. Pellon, E. Sanches, JG. dos Santos, MR. Teixeira, and S. Castedo. Mutations in exon 14 of dihydropyrimidine dehydrogenase and 5-fluorouracil toxicity in portuguese colorectal cancer patients. Genet Med, 6(2):102–107, 2004. PMID: 15017333.

[140] M. Steiner, M. Seule, B. Steiner, I. Bauer, M. Freund, CH. Köhne, and P. Schuff-Werner. 5-fluorouracil/irinotecan induced lethal toxicity as a result of a combined pharmacogenetic syndrome: report of a case. J Clin Pathol, 58 (5):553–555, 2005. PMCID: PMC1770668, PMID: 15858133.

[141] NA. Al-Sanna'a, AB. Van Kuilenburg, TM. Atrak, MA. Abdul-Jabbar, and AH. Van Gennip. Dihydropyrimidine dehydrogenase deficiency presenting at birth. J Inherit Metab Dis, 28(5):793–796, 2005. PMID: 16151913.

[142] HH. Ezzeldin, AM. Lee, LK. Mattison, and RB. Diasio. Methylation of the dpyd promoter: an alternative mechanism for dihydropyrimidine dehydrogenase deficiency in cancer patients. Clin Cancer Res, 11(24 Pt 1): 8699–8705, 2005. PMID: 16361556.

[143] R. Largillier, MC. Etienne-Grimaldi, JL. Formento, J. Ciccolini, JF. Nebbia, A. Ginot, M. Francoual, N. Renée, JM. Ferrero, C. Foa, M. Namer, B. Lacarelle, and G. Milano. Pharmacogenetics of capecitabine in advanced breast cancer patients. Clin Cancer Res, 12(18):5496–5502, 2006. PMID: 17000685.

[144] M. Boisdron-Celle, G. Remaud, S. Traore, AL. Poirier, L. Gamelin, A. Morel, and E. Gamelin. 5-fluorouracil-related severe toxicity: a comparison of different methods for the pretherapeutic detection of dihydropyrimidine dehydrogenase deficiency. Cancer Lett, 249(2):271–282, 2007. PMID: 17064846.

[145] A. Morel, M. Boisdron-Celle, L. Fey, P. Soulie, MC. Craipeau, S. Traore, and E. Gamelin. Clinical relevance of different dihydropyrimidine dehydrogenase gene single nucleotide polymorphisms on 5-fluorouracil tolerance. Mol Cancer Ther, 5(11):2895–2904, 2006. PMID: 17121937.

[146] MW. Saif, H. Ezzeldin, K. Vance, S. Sellers, and RB. Diasio. Dpyd*2a mutation: the most common mutation associated with dpd deficiency. Cancer Chemother Pharmacol, 60(4):503–507, 2007. PMID: 17165084.

[147] J. Salgado, N. Zabalegui, C. Gil, I. Monreal, J. Rodríguez, and J. García-Foncillas. Polymorphisms in the thymidylate synthase and dihydropyrimidine dehydrogenase genes predict response and toxicity to capecitabine-raltitrexed in colorectal cancer. Oncol Rep, 17(2):325–328, 2007. PMID: 17203168.

[148] N. Magné, MC. Etienne-Grimaldi, L. Cals, N. Renée, JL. Formento, M. Francoual, and G. Milano. Dihydropyrimidine dehydrogenase activity and the ivs14+1g>a mutation in patients developing 5fu-related toxicity. Br J Clin Pharmacol, 64(2):237–240, 2007. PMCID: PMC2000632, PMID: 17335544.

[149] O. Capitain, M. Boisdron-Celle, AL. Poirier, S. Abadie-Lacourtoisie, A. Morel, and E. Gamelin. The influence of fluorouracil outcome parameters on tolerance and efficacy in patients with advanced colorectal cancer. Pharmacogenomics J, 8(4):256–267, 2008. PMID: 17700593.

[150] M. Schwab, UM. Zanger, C. Marx, E. Schaeffeler, K. Klein, J. Dippon, R. Kerb, J. Blievernicht, J. Fischer, U. Hofmann, C. Bokemeyer, and M. Eichelbaum. Role of genetic and nongenetic factors for fluorouracil treatment-related severe toxicity: a prospective clinical trial by the german 5-fu toxicity study group. J Clin Oncol, 26(13):2131–2138, 2008. PMID: 18299612.

[151] V. Sulzyc-Bielicka, A. Bińczak-Kuleta, W. Pioch, J. Kładny, K. Gziut, D. Bielicki, and A. Ciechanowicz. 5-fluorouracil toxicity-attributable ivs14 + 1g > a mutation of the dihydropyrimidine dehydrogenase gene in polish colorectal cancer patients. Pharmacol Rep, 60(2):238–242, 2008. PMID: 18443386.

[152] AB. van Kuilenburg, JG. Maring, A. Schalhorn, C. Terborg, H. Schmalenberg, D. Behnke, W. Schwabe, K. Jabschinsky, and P. Hausler. Pharmacokinetics of 5-fluorouracil in patients heterozygous for the ivs14+1g > a mutation in the dihydropyrimidine dehydrogenase gene. Nucleosides Nucleotides Nucleic Acids, 27(6):692–698, 2008. PMID: 18600527.

[153] E. Gross, B. Busse, M. Riemenschneider, S. Neubauer, K. Seck, HG. Klein, M. Kiechle, F. Lordick, and A. Meindl. Strong association of a common dihydropyrimidine dehydrogenase gene polymorphism with fluoropyrimidine-related toxicity in cancer patients. PLoS One, 3(12):e4003, 2008. PMCID: PMC2602733, PMID: 19104657.

[154] Z. Kleibl, J. Fidlerova, P. Kleiblova, S. Kormunda, M. Bilek, K. Bouskova, J. Sevcik, and J. Novotny. Influence of dihydropyrimidine dehydrogenase gene (dpyd) coding sequence variants on the development of fluoropyrimidine-related toxicity in patients with high-grade toxicity and patients with excellent tolerance of fluoropyrimidine-based chemotherapy. Neoplasma, 56(4):303–316, 2009. PMID: 19473056.

[155] A. Loganayagam, M. Arenas-Hernandez, L. Fairbanks, P. Ross, JD. Sanderson, and AM. Marinaki. The contribution of deleterious dpyd gene sequence variants to fluoropyrimidine toxicity in british cancer patients. Cancer Chemother Pharmacol,

65(2):403–406, 2010. PMID: 19795123.

[156] MS. Braun, SD. Richman, L. Thompson, CL. Daly, AM. Meade, JW. Adlard, JM. Allan, MK. Parmar, P. Quirke, and MT. Seymour. Association of molecular markers with toxicity outcomes in a randomized trial of chemotherapy for advanced colorectal cancer: the focus trial. J Clin Oncol, 27(33):5519–5528, 2009. PMID: 19858398.

[157] V. Boige, J. Mendiboure, JP. Pignon, MA. Loriot, M. Castaing, M. Barrois, D. Malka, DA. Trégouët, O. Bouché, D. Le Corre, I. Miran, C. Mulot, M. Ducreux, P. Beaune, and P. Laurent-Puig. Pharmacogenetic assessment of toxicity and outcome in patients with metastatic colorectal cancer treated with lv5fu2, folfox, and folfiri: Ffcd 2000-05. J Clin Oncol, 28(15):2556–2564, 2010. PMID: 20385995.

[158] T. Cerić, N. Obralić, L. Kapur-Pojskić, D. Macić, S. Beslija, A. Pasić, and S. Cerić. Investigation of ivs14 + 1g > a polymorphism of dpyd gene in a group of bosnian patients treated with 5-fluorouracil and capecitabine. Bosn J Basic Med Sci, 10(2):133–139, 2010. PMCID: PMC5509399, PMID: 20507294.

[159] HL. McLeod, DJ. Sargent, S. Marsh, EM. Green, CR. King, CS. Fuchs, RK. Ramanathan, SK. Williamson, BP. Findlay, SN. Thibodeau, A. Grothey, RF. Morton, and RM. Goldberg. Pharmacogenetic predictors of adverse events and response to chemotherapy in metastatic colorectal cancer: results from north american gastrointestinal intergroup trial n9741. J Clin Oncol, 28(20):3227–3233, 2010. PMCID: PMC2903324, PMID: 20530282.

[160] AB. van Kuilenburg, J. Meijer, AN. Mul, R. Meinsma, V. Schmid, D. Dobritzsch, RC. Hennekam, MM. Mannens, M. Kiechle, MC. Etienne-Grimaldi, HJ. Klümpen, JG. Maring, VA. Derleyn, E. Maartense, G. Milano, R. Vijzelaar, and E. Gross. Intragenic deletions and a deep intronic mutation affecting pre-mrna splicing in the dihydropyrimidine dehydrogenase gene as novel mechanisms causing 5-fluorouracil toxicity. Hum Genet, 128 (5):529–538, 2010. PMCID: PMC2955237, PMID: 20803296.

[161] J. Savva-Bordalo, J. Ramalho-Carvalho, M. Pinheiro, VL. Costa, A. Rodrigues, PC. Dias, I. Veiga, M. Machado, MR. Teixeira, R. Henrique, and C. Jerónimo. Promoter methylation and large intragenic rearrangements of dpyd are not implicated in severe toxicity to 5-fluorouracil-based chemotherapy in gastrointestinal cancer patients. BMC Cancer, 10:470, 2010. PMCID: PMC2940808, PMID: 20809970.

[162] MH. Kristensen, PL. Pedersen, GV. Melsen, J. Ellehauge, and J. Mejer. Variants in the dihydropyrimidine dehydrogenase, methylenetetrahydrofolate reductase and thymidylate synthase genes predict early toxicity of 5-fluorouracil in colorectal cancer patients. J Int Med Res, 38(3):870–883, 2010. PMID: 20819423.

[163] P. Cellier, B. Leduc, L. Martin, B. Vié, C. Chevelle, V. Vendrely, A. Salemkour, C. Carrie, G. Calais, P. Burtin, L. Campion, M. Boisdron-Celle, A. Morel, V. Berger, and E. Gamelin. Phase ii study of preoperative radiation plus concurrent daily tegafur-uracil (uft) with leucovorin for locally advanced rectal cancer. BMC Cancer, 11:98, 2011. PMCID: PMC3070684, PMID: 21410976.

[164] MJ. Deenen, J. Tol, AM. Burylo, VD. Doodeman, A. de Boer, A. Vincent, HJ. Guchelaar,

PH. Smits, JH. Beijnen, CJ. Punt, JH. Schellens, and A. Cats. Relationship between single nucleotide polymorphisms and haplotypes in dpyd and toxicity and efficacy of capecitabine in advanced colorectal cancer. Clin Cancer Res, 17(10): 3455–3468, 2011. PMID: 21498394.

[165] CA. Galván, OC. Elbarcha, EJ. Fernández, DM. Beltramo, and NW. Soria. Genetic profiling of gstp1, dpyd, fcgr2a, fcgr3a and ccnd1 genes in an argentinian population. Clin Biochem, 44(13):1058–1061, 2011. PMID: 21723269.

[166] U. Amstutz, TK. Froehlich, and CR. Largiadèr. Dihydropyrimidine dehydrogenase gene as a major predictor of severe 5-fluorouracil toxicity. Pharmacogenomics, 12(9):1321–1336, 2011. PMID: 21919607.

[167] AB. van Kuilenburg, P. Häusler, A. Schalhorn, MW. Tanck, JH. Proost, C. Terborg, D. Behnke, W. Schwabe, K. Jabschinsky, and JG. Maring. Evaluation of 5-fluorouracil pharmacokinetics in cancer patients with a c.1905+1g>a mutation in dpyd by means of a bayesian limited sampling strategy. Clin Pharmacokinet, 51(3): 163–174, 2012. PMID: 22339448.

[168] M. Whirl-Carrillo, EM. McDonagh, JM. Hebert, L. Gong, K. Sangkuhl, CF. Thorn, RB. Altman, and TE. Klein. Pharmacogenomics knowledge for personalized medicine. Clin Pharmacol Ther, 92(4):414–417, 2012. PMCID: PMC3660037, PMID: 22992668.

[169] SM. Offer, NJ. Wegner, C. Fossum, K. Wang, and RB. Diasio. Phenotypic profiling of dpyd variations relevant to 5-fluorouracil sensitivity using real-time cellular analysis and in vitro measurement of enzyme activity. Cancer Res, 73(6):1958–1968, 2013. PMCID: PMC3602211, PMID: 23328581.

[170] E. Borràs, E. Dotor, A. Arcusa, MJ. Gamundi, I. Hernan, M. de Sousa Dias, B. Mañé, JA. Agúndez, M. Blanca, and M. Carballo. High-resolution melting analysis of the common c.1905+1g>a mutation causing dihydropyrimidine dehydrogenase deficiency and lethal 5-fluorouracil toxicity. Front Genet, 3:312, 2012. PMCID: PMC3547229, PMID: 23335937.

[171] D. Dhawan, H. Panchal, S. Shukla, and H. Padh. Genetic variability & chemotoxicity of 5-fluorouracil & cisplatin in head & neck cancer patients: a preliminary study. Indian J Med Res, 137(1):125–129, 2013. PMCID: PMC3657875, PMID: 23481061.

[172] E. Magnani, E. Farnetti, D. Nicoli, B. Casali, L. Savoldi, C. Focaccetti, C. Boni, A. Albini, and M. Banzi. Fluoropyrimidine toxicity in patients with dihydropyrimidine dehydrogenase splice site variant: the need for further revision of dose and schedule. Intern Emerg Med, 8(5):417–423, 2013. PMID: 23585145.

[173] MW. Saif. Dihydropyrimidine dehydrogenase gene (dpyd) polymorphism among caucasian and non-caucasian patients with 5-fu- and capecitabine-related toxicity using full sequencing of dpyd. Cancer Genomics Proteomics, 10(2):89–92, 2013. PMID: 23603345.

[174] A. Loganayagam, M. Arenas Hernandez, A. Corrigan, L. Fairbanks, CM. Lewis, P. Harper, N. Maisey, P. Ross, JD. Sanderson, and AM. Marinaki. Pharmacogenetic variants in the dpyd, tyms, cda and mthfr genes are clinically significant predictors of fluoropyrimidine

toxicity. Br J Cancer, 108(12):2505–2515, 2013. PMCID: PMC3694243, PMID: 23736036.

[175] S. Terrazzino, S. Cargnin, M. Del Re, R. Danesi, PL. Canonico, and AA. Genazzani. Dpyd ivs14+1g>a and 2846a>t genotyping for the prediction of severe fluoropyrimidine-related toxicity: a meta-analysis. Pharmacogenomics, 14(11):1255–1272, 2013. PMID: 23930673.

[176] BA. Jennings, YK. Loke, J. Skinner, M. Keane, GS. Chu, R. Turner, D. Epurescu, A. Barrett, and G. Willis. Evaluating predictive pharmacogenetic signatures of adverse events in colorectal cancer patients treated with fluoropyrimidines. PLoS One, 8(10):e78053, 2013. PMCID: PMC3805522, PMID: 24167597.

[177] D. Rosmarin, C. Palles, D. Church, E. Domingo, A. Jones, E. Johnstone, H. Wang, S. Love, P. Julier, C. Scudder, G. Nicholson, A. Gonzalez-Neira, M. Martin, D. Sargent, E. Green, H. McLeod, UM. Zanger, M. Schwab, M. Braun, M. Seymour, L. Thompson, B. Lacas, V. Boige, N. Ribelles, S. Afzal, H. Enghusen, SA. Jensen, MC. Etienne-Grimaldi, G. Milano, M. Wadelius, B. Glimelius, H. Garmo, M. Gusella, T. Lecomte, P. Laurent-Puig, E. Martinez-Balibrea, R. Sharma, J. Garcia-Foncillas, Z. Kleibl, A. Morel, JP. Pignon, R. Midgley, D. Kerr, and I. Tomlinson. Genetic markers of toxicity from capecitabine and other fluorouracil-based regimens: investigation in the quasar2 study, systematic review, and meta-analysis. J Clin Oncol, 32(10):1031–1039, 2014. PMCID: PMC4879695, PMID: 24590654.

[178] D. Rosmarin, C. Palles, A. Pagnamenta, K. Kaur, G. Pita, M. Martin, E. Domingo, A. Jones, K. Howarth, L. Freeman-Mills, E. Johnstone, H. Wang, S. Love, C. Scudder, P. Julier, C. Fernández-Rozadilla, C. Ruiz-Ponte, A. Carracedo, S. Castellvi-Bel, A. Castells, A. Gonzalez-Neira, J. Taylor, R. Kerr, D. Kerr, and I. Tomlinson. A candidate gene study of capecitabine-related toxicity in colorectal cancer identifies new toxicity variants at dpyd and a putative role for enosf1 rather than tyms. Gut, 64(1):111–120, 2015. PMCID: PMC4283622, PMID: 24647007.

[179] SM. Offer, CC. Fossum, NJ. Wegner, AJ. Stuflesser, GL. Butterfield, and RB. Diasio. Comparative functional analysis of dpyd variants of potential clinical relevance to dihydropyrimidine dehydrogenase activity. Cancer Res, 74(9):2545–2554, 2014. PMCID: PMC4012613, PMID: 24648345.

[180] B. Suarez Martinez-Falero and R. Gillmore. A rare cause of susceptibility to neutropenic sepsis in a patient with metastatic pancreas cancer. BMJ Case Rep, 2014, 2014. PMCID: PMC3987224, PMID: 24700034.

[181] X. Cai, JM. Fang, P. Xue, WF. Song, J. Hu, HL. Gu, HY. Yang, and LW. Wang. The role of ivs14+1 g > a genotype detection in the dihydropyrimidine dehydrogenase gene and pharmacokinetic monitoring of 5-fluorouracil in the individualized adjustment of 5-fluorouracil for patients with local advanced and metastatic colorectal cancer: a preliminary report. Eur Rev Med Pharmacol Sci, 18(8):1247–1258, 2014. PMID: 24817302.

[182] TK. Froehlich, U. Amstutz, S. Aebi, M. Joerger, and CR. Largiadèr. Clinical importance of risk variants in the dihydropyrimidine dehydrogenase gene for the prediction of early-

onset fluoropyrimidine toxicity. Int J Cancer, 136(3):730–739, 2015. PMID: 24923815.

[183] AM. Lee, Q. Shi, E. Pavey, SR. Alberts, DJ. Sargent, FA. Sinicrope, JL. Berenberg, RM. Goldberg, and RB. Diasio. Dpyd variants as predictors of 5-fluorouracil toxicity in adjuvant colon cancer treatment (ncctg n0147). J Natl Cancer Inst, 106(12), 2014. PMCID: PMC4271081, PMID: 25381393.

[184] J. Sistonen, B. Büchel, TK. Froehlich, D. Kummer, S. Fontana, M. Joerger, AB. van Kuilenburg, and CR. Largiadèr. Predicting 5-fluorouracil toxicity: Dpd genotype and 5,6-dihydrouracil:uracil ratio. Pharmacogenomics, 15(13):1653–1666, 2014. PMID: 25410891.

[185] X. Zhu, S. Petrovski, P. Xie, EK. Ruzzo, YF. Lu, KM. McSweeney, B. Ben-Zeev, A. Nissenkorn, Y. Anikster, D. Oz-Levi, RS. Dhindsa, Y. Hitomi, K. Schoch, RC. Spillmann, G. Heimer, D. Marek-Yagel, M. Tzadok, Y. Han, G. Worley, J. Goldstein, YH. Jiang, D. Lancet, E. Pras, V. Shashi, D. McHale, AC. Need, and DB. Goldstein. Whole-exome sequencing in undiagnosed genetic diseases: interpreting 119 trios. Genet Med, 17(10): 774–781, 2015. PMCID: PMC4791490, PMID: 25590979.

[186] M. Joerger, AD. Huitema, H. Boot, A. Cats, VD. Doodeman, PH. Smits, L. Vainchtein, H. Rosing, I. Meijerman, M. Zueger, D. Meulendijks, TD. Cerny, JH. Beijnen, and JH. Schellens. Germline tyms genotype is highly predictive in patients with metastatic gastrointestinal malignancies receiving capecitabine-based chemotherapy. Cancer Chemother Pharmacol, 75(4):763–772, 2015. PMID: 25677447.

[187] G. Toffoli, L. Giodini, A. Buonadonna, M. Berretta, A. De Paoli, S. Scalone, G. Miolo, E. Mini, S. Nobili, S. Lonardi, N. Pella, G. Lo Re, M. Montico, R. Roncato, E. Dreussi, S. Gagno, and E. Cecchin. Clinical validity of a dpyd-based pharmacogenetic test to predict severe toxicity to fluoropyrimidines. Int J Cancer, 137(12): 2971–2980, 2015. PMID: 26099996.

[188] G. Gentile, A. Botticelli, L. Lionetto, F. Mazzuca, M. Simmaco, P. Marchetti, and M. Borro. Genotype-phenotype correlations in 5-fluorouracil metabolism: a candidate dpyd haplotype to improve toxicity prediction. Pharmacogenomics J, 16(4):320–325, 2016. PMID: 26216193.

[189] F. Thomas, I. Hennebelle, C. Delmas, I. Lochon, C. Dhelens, C. Garnier Tixidre, A. Bonadona, N. Penel, A. Goncalves, JP. Delord, C. Toulas, and E. Chatelut. Genotyping of a family with a novel deleterious dpyd mutation supports the pretherapeutic screening of dpd deficiency with dihydrouracil/uracil ratio. Clin Pharmacol Ther, 99(2):235–242, 2016. PMID: 26265035.

[190] I. Karbassi, GA. Maston, A. Love, C. DiVincenzo, CD. Braastad, CD. Elzinga, AR. Bright, D. Previte, K. Zhang, CM. Rowland, M. McCarthy, JL. Lapierre, F. Dubois, KA. Medeiros, SD. Batish, J. Jones, K. Liaquat, CA. Hoffman, M. Jaremko, Z. Wang, W. Sun, A. Buller-Burckle, CM. Strom, SB. Keiles, and JJ. Higgins. A standardized dna variant scoring system for pathogenicity assessments in mendelian disorders. Hum Mutat, 37 (1):127–134, 2016. PMCID: PMC4737317, PMID: 26467025.

[191] D. Meulendijks, LM. Henricks, GS. Sonke, MJ. Deenen, TK. Froehlich, U. Amstutz, CR.

Largiadèr, BA. Jennings, AM. Marinaki, JD. Sanderson, Z. Kleibl, P. Kleiblova, M. Schwab, UM. Zanger, C. Palles, I. Tomlinson, E. Gross, AB. van Kuilenburg, CJ. Punt, M. Koopman, JH. Beijnen, A. Cats, and JH. Schellens. Clinical relevance of dpyd variants c.1679t>g, c.1236g>a/hapb3, and c.1601g>a as predictors of severe fluoropyrimidine-associated toxicity: a systematic review and meta-analysis of individual patient data. Lancet Oncol, 16(16):1639–1650, 2015. PMID: 26603945.

[192] V. Boige, M. Vincent, P. Alexandre, S. Tejpar, S. Landolfi, K. Le Malicot, R. Greil, PJ. Cuyle, M. Yilmaz, R. Faroux, A. Matzdorff, R. Salazar, C. Lepage, J. Taieb, and P. Laurent-Puig. Dpyd genotyping to predict adverse events following treatment with fluorouracil-based adjuvant chemotherapy in patients with stage iii colon cancer: A secondary analysis of the petacc-8 randomized clinical trial. JAMA Oncol, 2(5):655–662, 2016. PMID: 26794347.

[193] ABPV. Kuilenburg, J. Meijer, MWT. Tanck, D. Dobritzsch, L. Zoetekouw, LL. Dekkers, J. Roelofsen, R. Meinsma, M. Wymenga, W. Kulik, B. Büchel, RCM. Hennekam, and CR. Largiadèr. Phenotypic and clinical implications of variants in the dihydropyrimidine dehydrogenase gene. Biochim Biophys Acta, 1862(4):754–762, 2016. PMID: 26804652.

[194] XQ. Zhao, WJ. Cao, HP. Yang, XW. Yang, P. Tang, L. Sun, and X. Gao. Dpyd gene polymorphisms are associated with risk and chemotherapy prognosis in pediatric patients with acute lymphoblastic leukemia. Tumour Biol, 37(8):10393–10402, 2016. PMID: 26846104.

[195] F. Mazzuca, M. Borro, A. Botticelli, E. Mazzotti, L. Marchetti, G. Gentile, M. La Torre, L. Lionetto, M. Simmaco, and P. Marchetti. Pre-treatment evaluation of 5-fluorouracil degradation rate: association of poor and ultra-rapid metabolism with severe toxicity in a colorectal cancer patients cohort. Oncotarget, 7(15):20612–20620, 2016. PMCID: PMC4991479, PMID: 26967565.

[196] G. Milano. Highlight on dpyd gene polymorphisms and treatment by capecitabine (.). Scand J Clin Lab Invest Suppl, 245:30–33, 2016. PMID: 27454530.

[197] M. Borro, A. Botticelli, F. Mazzuca, EC. Onesti, G. Gentile, A. Romiti, B. Cerbelli, E. Mazzotti, L. Marchetti, L. Lionetto, M. Simmaco, and P. Marchetti. Pre-treatment assay of 5-fluorouracil degradation rate (5-fudr) to improve prediction of 5-fluorouracil toxicity in gastro-esophageal cancer. Oncotarget, 8(8):14050–14057, 2017. PMCID: PMC5355161, PMID: 27738344.

[198] M. Roberto, A. Romiti, A. Botticelli, F. Mazzuca, L. Lionetto, G. Gentile, I. Paris, R. Falcone, M. Bassanelli, FR. Di Pietro, CE. Onesti, E. Anselmi, S. Macrini, M. Simmaco, and P. Marchetti. Evaluation of 5-fluorouracil degradation rate and pharmacogenetic profiling to predict toxicity following adjuvant capecitabine. Eur J Clin Pharmacol, 73(2):157–164, 2017. PMID: 27864592.

[199] Q. Nie, S. Shrestha, EE. Tapper, CS. Trogstad-Isaacson, KJ. Bouchonville, AM. Lee, R. Wu, CR. Jerde, Z. Wang, PA. Kubica, SM. Offer, and RB. Diasio. Quantitative contribution of rs75017182 to dihydropyrimidine dehydrogenase mrna splicing and enzyme activity. Clin Pharmacol Ther, 102(4):662–670, 2017. PMCID: PMC6138243, PMID: 28295243.

[200] MC. Etienne-Grimaldi, JC. Boyer, C. Beroud, L. Mbatchi, A. van Kuilenburg, C. Bobin-Dubigeon, F. Thomas, E. Chatelut, JL. Merlin, F. Pinguet, C. Ferrand, J. Meijer, A. Evrard, L. Llorca, G. Romieu, P. Follana, T. Bachelot, L. Chaigneau, X. Pivot, V. Dieras, R. Largillier, M. Mousseau, A. Goncalves, H. Roché, J. Bonneterre, V. Servent, N. Dohollou, Y. Château, E. Chamorey, JP. Desvignes, D. Salgado, JM. Ferrero, and G. Milano. New advances in dpyd genotype and risk of severe toxicity under capecitabine. PLoS One, 12(5):e0175998, 2017. PMCID: PMC5421769, PMID: 28481884.

[201] LM. Henricks, EJM. Siemerink, H. Rosing, J. Meijer, SMI. Goorden, AM. Polstra, L. Zoetekouw, A. Cats, JHM. Schellens, and ABP. van Kuilenburg. Capecitabine-based treatment of a patient with a novel dpyd genotype and complete dihydropyrimidine dehydrogenase deficiency. Int J Cancer, 142(2):424–430, 2018. PMID: 28929491.

[202] A. Ruzzo, F. Graziano, F. Galli, F. Galli, E. Rulli, S. Lonardi, M. Ronzoni, B. Massidda, V. Zagonel, N. Pella, C. Mucciarini, R. Labianca, MT. Ionta, I. Bagaloni, E. Veltri, P. Sozzi, S. Barni, V. Ricci, L. Foltran, M. Nicolini, E. Biondi, A. Bramati, D. Turci, S. Lazzarelli, C. Verusio, F. Bergamo, A. Sobrero, L. Frontini, M. Menghi, and M. Magnani. Dihydropyrimidine dehydrogenase pharmacogenetics for predicting fluoropyrimidine-related toxicity in the randomised, phase iii adjuvant tosca trial in high-risk colon cancer patients. Br J Cancer, 117(9): 1269–1277, 2017. PMCID: PMC5709672, PMID: 29065426.

[203] NA. Nahid, MNH. Apu, MR. Islam, S. Shabnaz, SM. Chowdhury, MU. Ahmed, Z. Nahar, MS. Islam, MS. Islam, and A. Hasnat. Dpyd*2a and mthfr c677t predict toxicity and efficacy, respectively, in patients on chemotherapy with 5-fluorouracil for colorectal cancer. Cancer Chemother Pharmacol, 81(1):119–129, 2018. PMID: 29134491.

[204] U. Amstutz, LM. Henricks, SM. Offer, J. Barbarino, JHM. Schellens, JJ. Swen, TE. Klein, HL. McLeod, KE. Caudle, RB. Diasio, and M. Schwab. Clinical pharmacogenetics implementation consortium (cpic) guideline for dihydropyrimidine dehydrogenase genotype and fluoropyrimidine dosing: 2017 update. Clin Pharmacol Ther, 103(2):210–216, 2018. PMCID: PMC5760397, PMID: 29152729.

[205] LM. Henricks, CATC. Lunenburg, FM. de Man, D. Meulendijks, GWJ. Frederix, E. Kienhuis, GJ. Creemers, A. Baars, VO. Dezentjé, ALT. Imholz, FJF. Jeurissen, JEA. Portielje, RLH. Jansen, P. Hamberg, AJ. Ten Tije, HJ. Droogendijk, M. Koopman, P. Nieboer, MHW. van de Poel, CMPW. Mandigers, H. Rosing, JH. Beijnen, EV. Werkhoven, ABP. van Kuilenburg, RHN. van Schaik, RHJ. Mathijssen, JJ. Swen, H. Gelderblom, A. Cats, HJ. Guchelaar, and JHM. Schellens. Dpyd genotype-guided dose individualisation of fluoropyrimidine therapy in patients with cancer: a prospective safety analysis. Lancet Oncol, 19(11):1459–1467, 2018. PMID: 30348537.

[206] R. Meinsma, P. Fernandez-Salguero, AB. Van Kuilenburg, AH. Van Gennip, and FJ. Gonzalez. Human polymorphism in drug metabolism: mutation in the dihydropyrimidine dehydrogenase gene results in exon skipping and thymine uracilurea. DNA Cell Biol, 14(1):1–6, 1995. PMID: 7832988.

[207] AH. van Gennip, NG. Abeling, AE. Stroomer, H. van Lenthe, and HD. Bakker. Clinical and biochemical findings in six patients with pyrimidine degradation defects. J Inherit Metab Dis, 17(1):130–132, 1994. PMID: 8051923.

[208] X. Wei, HL. McLeod, J. McMurrough, FJ. Gonzalez, and P. Fernandez-Salguero. Molecular basis of the human dihydropyrimidine dehydrogenase deficiency and 5-fluorouracil toxicity. J Clin Invest, 98(3):610–615, 1996. PMCID: PMC507468, PMID: 8698850.

[209] P. Vreken, AB. Van Kuilenburg, R. Meinsma, GP. Smit, HD. Bakker, RA. De Abreu, and AH. van Gennip. A point mutation in an invariant splice donor site leads to exon skipping in two unrelated dutch patients with dihydropyrimidine dehydrogenase deficiency. J Inherit Metab Dis, 19(5):645–654, 1996. PMID: 8892022.

[210] I. Holopainen, K. Pulkki, OJ. Heinonen, K. Näntö-Salonen, L. Haataja, J. Greter, E. Holme, AB. van Kuilenburg, P. Vreken, and AH. van Gennip. Partial epilepsy in a girl with a symptom-free sister: first two finnish patients with dihydropyrimidine dehydrogenase deficiency. J Inherit Metab Dis, 20(5):719–720, 1997. PMID: 9323575.

[211] P. Vreken, AB. Van Kuilenburg, R. Meinsma, and AH. van Gennip. Dihydropyrimidine dehydrogenase (dpd) deficiency: identification and expression of missense mutations c29r, r886h and r235w. Hum Genet, 101(3): 333–338, 1997. PMID: 9439663.

[212] AB. Van Kuilenburg, P. Vreken, LV. Beex, R. Meinsma, H. Van Lenthe, RA. De Abreu, and AH. van Gennip. Heterozygosity for a point mutation in an invariant splice donor site of dihydropyrimidine dehydrogenase and severe 5-fluorouracil related toxicity. Eur J Cancer, 33(13):2258–2264, 1997. PMID: 9470816.

[213] P. Vreken, AB. van Kuilenburg, R. Meinsma, FA. Beemer, M. Duran, and AH. van Gennip. Dihydropyrimidine dehydrogenase deficiency: a novel mutation and expression of missense mutations in e. coli. J Inherit Metab Dis, 21(3):276–279, 1998. PMID: 9686374.

[214] J. Lindberg, IG. Mills, D. Klevebring, W. Liu, M. Neiman, J. Xu, P. Wikström, P. Wiklund, F. Wiklund, L. Egevad, and H. Grönberg. The mitochondrial and autosomal mutation landscapes of prostate cancer. Eur Urol, 63(4): 702–708, 2013. PMID: 23265383.

[215] K. Miyake, S. Imura, T. Yoshizumi, T. Ikemoto, Y. Morine, and M. Shimada. Role of thymidine phosphorylase and orotate phosphoribosyltransferase mrna expression and its ratio to dihydropyrimidine dehydrogenase in the prognosis and clinicopathological features of patients with pancreatic cancer. Int J Clin Oncol, 12(2):111–119, 2007. PMID: 17443278.

[216] Y. Fukui, T. Oka, S. Nagayama, PV. Danenberg, KD. Danenberg, and M. Fukushima. Thymidylate synthase, dihydropyrimidine dehydrogenase, orotate phosphoribosyltransferase mrna and protein expression levels in solid tumors in large scale population analysis. Int J Mol Med, 22(6):709–716, 2008. PMID: 19020767.

[217] T. Takahashi, H. Yoshida, Y. Mamada, N. Taniai, Y. Mizuguchi, T. Shimizu, D. Kakinuma, Y. Ishikawa, K. Akimaru, Y. Sugisaki, and T. Tajiri. Profiling of fluorouracil-related genes by microdissection technique in hepatocellular carcinoma. Hepatogastroenterology, 54(78):1612–1616, 2007. PMID: 18019677.

[218] R. Napieralski, K. Ott, M. Kremer, K. Specht, H. Vogelsang, K. Becker, M. Müller, F. Lordick, U. Fink, J. Rüdiger Siewert, H. Höfler, and G. Keller. Combined gadd45a and

thymidine phosphorylase expression levels predict response and survival of neoadjuvant-treated gastric cancer patients. Clin Cancer Res, 11(8): 3025–3031, 2005. PMID: 15837757.

[219] M. Oeda, K. Yoshida, Y. Sanada, Y. Wada, T. Suzuki, H. Mizuiri, K. Konishi, H. Shigematsu, K. Tanabe, and M. Fukushima. The expression profiles of orotate phosphoribosyltransferase and dihydropyrimidine dehydrogenase in gastric cancer and their clinical significance. Oncol Rep, 16(6):1165–1172, 2006. PMID: 17089033.

[220] H. Makino, H. Uetake, K. Danenberg, PV. Danenberg, and K. Sugihara. Efficacy of laser capture microdissection plus rt-pcr technique in analyzing gene expression levels in human gastric cancer and colon cancer. BMC Cancer, 8:210, 2008. PMCID: PMC2533342, PMID: 18652704.

[221] D. Vallböhmer, DY. Yang, H. Kuramochi, D. Shimizu, KD. Danenberg, J. Lindebjerg, JN. Nielsen, A. Jakobsen, and PV. Danenberg. Dpd is a molecular determinant of capecitabine efficacy in colorectal cancer. Int J Oncol, 31(2):413–418, 2007. PMID: 17611699.

[222] H. Nagano, W. Ichikawa, M. Simizu, Y. Shirota, and Z. Nihei. [thymidylate synthase and dihydropyrimidine dehydrogenase gene expressions in colorectal cancer using the danenberg tumor profile method]. Gan To Kagaku Ryoho, 31(6):889–892, 2004. PMID: 15222106.

[223] EA. Kidd, J. Yu, X. Li, WD. Shannon, MA. Watson, and HL. McLeod. Variance in the expression of 5-fluorouracil pathway genes in colorectal cancer. Clin Cancer Res, 11(7):2612–2619, 2005. PMID: 15814641.

[224] F. Amatori, A. Di Paolo, M. Del Tacca, G. Fontanini, F. Vannozzi, L. Boldrini, G. Bocci, M. Lastella, and R. Danesi. Thymidylate synthase, dihydropyrimidine dehydrogenase and thymidine phosphorylase expression in colorectal cancer and normal mucosa in patients. Pharmacogenet Genomics, 16(11):809–816, 2006. PMID: 17047489.

[225] DA. Franco and HS. Greenberg. 5-fu multifocal inflammatory leukoencephalopathy and dihydropyrimidine dehydrogenase deficiency. Neurology, 56(1):110–112, 2001. PMID: 11148247.

[226] C. Schmidt, U. Hofmann, D. Kohlmüller, T. Mürdter, UM. Zanger, M. Schwab, and GF. Hoffmann. Comprehensive analysis of pyrimidine metabolism in 450 children with unspecific neurological symptoms using high-pressure liquid chromatography-electrospray ionization tandem mass spectrometry. J Inherit Metab Dis, 28 (6):1109–1122, 2005. PMID: 16435204.

[227] AB. van Kuilenburg, JW. Baars, R. Meinsma, and AH. van Gennip. Lethal 5-fluorouracil toxicity associated with a novel mutation in the dihydropyrimidine dehydrogenase gene. Ann Oncol, 14(2):341–342, 2003. PMID: 12562666.

[228] A. Broyl, SL. Corthals, JL. Jongen, B. van der Holt, R. Kuiper, Y. de Knegt, M. van Duin, L. el Jarari, U. Bertsch, HM. Lokhorst, BG. Durie, H. Goldschmidt, and P. Sonneveld. Mechanisms of peripheral neuropathy associated with bortezomib and vincristine in patients with newly diagnosed multiple myeloma: a prospective analysis of data from the

hovon-65/gmmg-hd4 trial. Lancet Oncol, 11(11):1057–1065, 2010. PMID: 20864405.

[229] B. Xu, JL. Roos, P. Dexheimer, B. Boone, B. Plummer, S. Levy, JA. Gogos, and M. Karayiorgou. Exome sequencing supports a de novo mutational paradigm for schizophrenia. Nat Genet, 43(9):864–868, 2011. PMCID: PMC3196550, PMID: 21822266.

[230] B. Xu, I. Ionita-Laza, JL. Roos, B. Boone, S. Woodrick, Y. Sun, S. Levy, JA. Gogos, and M. Karayiorgou. De novo gene mutations highlight patterns of genetic and neural complexity in schizophrenia. Nat Genet, 44(12): 1365–1369, 2012. PMCID: PMC3556813, PMID: 23042115.

[231] RB. Diasio, TL. Beavers, and JT. Carpenter. Familial deficiency of dihydropyrimidine dehydrogenase. biochemical basis for familial pyrimidinemia and severe 5-fluorouracil-induced toxicity. J Clin Invest, 81(1): 47–51, 1988. PMCID: PMC442471, PMID: 3335642.

[232] AB. van Kuilenburg, R. Meinsma, and AH. van Gennip. Pyrimidine degradation defects and severe 5-fluorouracil toxicity. Nucleosides Nucleotides Nucleic Acids, 23(8-9):1371–1375, 2004. PMID: 15571261.

[233] S. Podliesna, J. Delanne, L. Miller, DJ. Tester, M. Uzunyan, S. Yano, M. Klerk, BC. Cannon, A. Khongphatthanayothin, G. Laurent, G. Bertaux, S. Falcon-Eicher, S. Wu, HY. Yen, H. Gao, AAM. Wilde, L. Faivre, MJ. Ackerman, EM. Lodder, and CR. Bezzina. Supraventricular tachycardias, conduction disease, and cardiomyopathy in 3 families with the same rare variant in tnni3k (p.glu768lys). Heart Rhythm, 16(1): 98–9105, 2019. PMID: 30010057.

[234] S. Hoshimoto, CT. Kuo, KK. Chong, TL. Takeshima, Y. Takei, MW. Li, SK. Huang, MS. Sim, DL. Morton, and DS. Hoon. Aim1 and line-1 epigenetic aberrations in tumor and serum relate to melanoma progression and disease outcome. J Invest Dermatol, 132(6):1689–1697, 2012. PMCID: PMC3352986, PMID: 22402438.

[235] ME. Talkowski, SV. Mullegama, JA. Rosenfeld, BW. van Bon, Y. Shen, EA. Repnikova, J. Gastier-Foster, DL. Thrush, S. Kathiresan, DM. Ruderfer, C. Chiang, C. Hanscom, C. Ernst, AM. Lindgren, CC. Morton, Y. An, C. Astbury, LA. Brueton, KD. Lichtenbelt, LC. Ades, M. Fichera, C. Romano, JW. Innis, CA. Williams, D. Bartholomew, MI. Van Allen, A. Parikh, L. Zhang, BL. Wu, RE. Pyatt, S. Schwartz, LG. Shaffer, BB. de Vries, JF. Gusella, and SH. Elsea. Assessment of 2q23.1 microdeletion syndrome implicates mbd5 as a single causal locus of intellectual disability, epilepsy, and autism spectrum disorder. Am J Hum Genet, 89(4):551–563, 2011. PMCID: PMC3188839, PMID: 21981781.

[236] SV. Mullegama, JA. Rosenfeld, C. Orellana, BW. van Bon, S. Halbach, EA. Repnikova, L. Brick, C. Li, L. Dupuis, M. Rosello, S. Aradhya, DJ. Stavropoulos, K. Manickam, E. Mitchell, JC. Hodge, ME. Talkowski, JF. Gusella, K. Keller, J. Zonana, S. Schwartz, RE. Pyatt, DJ. Waggoner, LG. Shaffer, AE. Lin, BB. de Vries, R. Mendoza-Londono, and SH. Elsea. Reciprocal deletion and duplication at 2q23.1 indicates a role for mbd5 in autism spectrum disorder. Eur J Hum Genet, 22(1):57–63, 2014. PMCID: PMC3865402, PMID: 23632792.

[237] CS. Richards, S. Bale, DB. Bellissimo, S. Das, WW. Grody, MR. Hegde, E. Lyon, and BE. Ward. Acmg recommendations for standards for interpretation and reporting of sequence variations: Revisions 2007. Genet Med, 10(4):294–300, 2008. PMID: 18414213.

[238] JMM. Howson, W. Zhao, DR. Barnes, WK. Ho, R. Young, DS. Paul, LL. Waite, DF. Freitag, EB. Fauman, EL. Salfati, BB. Sun, JD. Eicher, AD. Johnson, WHH. Sheu, SF. Nielsen, WY. Lin, P. Surendran, A. Malarstig, JB. Wilk, A. Tybjærg-Hansen, KL. Rasmussen, PR. Kamstrup, P. Deloukas, J. Erdmann, S. Kathiresan, NJ. Samani, H. Schunkert, H. Watkins, R. Do, DJ. Rader, JA. Johnson, SL. Hazen, AA. Quyyumi, JA. Spertus, CJ. Pepine, N. Franceschini, A. Justice, AP. Reiner, S. Buyske, LA. Hindorff, CL. Carty, KE. North, C. Kooperberg, E. Boerwinkle, K. Young, M. Graff, U. Peters, D. Absher, CA. Hsiung, WJ. Lee, KD. Taylor, YH. Chen, IT. Lee, X. Guo, RH. Chung, YJ. Hung, JI. Rotter, JJ. Juang, T. Quertermous, TD. Wang, A. Rasheed, P. Frossard, DS. Alam, AAS. Majumder, E. Di Angelantonio, R. Chowdhury, YI. Chen, BG. Nordestgaard, TL. Assimes, J. Danesh, AS. Butterworth, and D. Saleheen. Fifteen new risk loci for coronary artery disease highlight arterial-wall-specific mechanisms. Nat Genet, 49(7):1113–1119, 2017. PMCID: PMC5555387, PMID: 28530674.

[239] SK. Ku, EJ. Yang, KS. Song, and JS. Bae. Rosmarinic acid down-regulates endothelial protein c receptor shedding in vitro and in vivo. Food Chem Toxicol, 59:311–315, 2013. PMID: 23774263.

[240] L. Shan, M. Yu, and EG. Snyderwine. Global gene expression profiling of chemically induced rat mammary gland carcinomas and adenomas. Toxicol Pathol, 33(7):768–775, 2005. PMID: 16316942.

[241] SJ. Hsiao, MA. Karajannis, D. Diolaiti, MM. Mansukhani, JG. Bender, AL. Kung, and JH. Garvin. A novel, potentially targetable tmem106b-braf fusion in pleomorphic xanthoastrocytoma. Cold Spring Harb Mol Case Stud, 3(2):a001396, 2017. PMCID: PMC5334470, PMID: 28299358.

[242] D. Pehlivan, CR. Beck, Y. Okamoto, T. Harel, ZH. Akdemir, SN. Jhangiani, MA. Withers, MT. Goksungur, CM. Carvalho, D. Czesnik, C. Gonzaga-Jauregui, W. Wiszniewski, DM. Muzny, RA. Gibbs, B. Rautenstrauss, MW. Sereda, and JR. Lupski. The role of combined snv and cnv burden in patients with distal symmetric polyneuropathy. Genet Med, 18(5):443–451, 2016. PMCID: PMC5322766, PMID: 26378787.

[243] R. Altara, FA. Zouein, RD. Brandão, SN. Bajestani, A. Cataliotti, and GW. Booz. In silico analysis of differential gene expression in three common rat models of diastolic dysfunction. Front Cardiovasc Med, 5:11, 2018. PMCID: PMC5850854, PMID: 29556499.

[244] J. Wang, PR. Ahimaz, S. Hashemifar, J. Khlevner, JA. Picoraro, W. Middlesworth, MM. Elfiky, J. Que, Y. Shen, and WK. Chung. Novel candidate genes in esophageal atresia/tracheoesophageal fistula identified by exome sequencing. Eur J Hum Genet, 29(1):122–130, 2021. PMCID: PMC7852873, PMID: 32641753.

[245] Y. Yu, K. Schleich, B. Yue, S. Ji, P. Lohneis, K. Kemper, MR. Silvis, N. Qutob, E. van Rooijen, M. Werner-Klein, L. Li, D. Dhawan, S. Meierjohann, M. Reimann, A. Elkahloun,

S. Treitschke, B. Dörken, C. Speck, FA. Mallette, LI. Zon, SL. Holmen, DS. Peeper, Y. Samuels, CA. Schmitt, and S. Lee. Targeting the senescence-overriding cooperative activity of structurally unrelated h3k9 demethylases in melanoma. Cancer Cell, 33(2):322–336, 2018. PMCID: PMC5977991, PMID: 29438700.

[246] AP. Trifa, C. Bănescu, AS. Bojan, CM. Voina, S;. Popa, S. Vis, an, AD. Ciubean, F. Tripon, D. Dima, VM. Popov, SC. Vesa, M. Andreescu, T. Török-Vistai, RG. Mihăilă, N. Berbec, I. Macarie, A. Coliţă, M. Iordache, AC. Cătană, MF. Farcas, , C. Tomuleasa, K. Vasile, C. Truică, A. Todincă, L. Pop-Muntean, R. Manolache, H. Bumbea, AM. Vlădăreanu, M. Gaman, CM. Ciufu, and RA. Popp. Mecom, hbs1l-myb, thrb-rarb, jak2, and tert polymorphisms defining the genetic predisposition to myeloproliferative neoplasms: A study on 939 patients. Am J Hematol, 93(1):100–106, 2018. PMID: 29047144.

[247] D. Pietra, A. Brisci, E. Rumi, S. Boggi, C. Elena, A. Pietrelli, R. Bordoni, M. Ferrari, F. Passamonti, G. De Bellis, L. Cremonesi, and M. Cazzola. Deep sequencing reveals double mutations in cis of mpl exon 10 in myeloproliferative neoplasms. Haematologica, 96(4):607–611, 2011. PMCID: PMC3069239, PMID: 21228032.

[248] Y. Pikman, BH. Lee, T. Mercher, E. McDowell, BL. Ebert, M. Gozo, A. Cuker, G. Wernig, S. Moore, I. Galinsky, DJ. DeAngelo, JJ. Clark, SJ. Lee, TR. Golub, M. Wadleigh, DG. Gilliland, and RL. Levine. Mplw515l is a novel somatic activating mutation in myelofibrosis with myeloid metaplasia. PLoS Med, 3(7):e270, 2006. PMCID: PMC1502153, PMID: 16834459.

[249] AD. Pardanani, RL. Levine, T. Lasho, Y. Pikman, RA. Mesa, M. Wadleigh, DP. Steensma, MA. Elliott, AP. Wolanskyj, WJ. Hogan, RF. McClure, MR. Litzow, DG. Gilliland, and A. Tefferi. Mpl515 mutations in myeloproliferative and other myeloid disorders: a study of 1182 patients. Blood, 108(10):3472–3476, 2006. PMID: 16868251.

[250] J. Kota, N. Caceres, and SN. Constantinescu. Aberrant signal transduction pathways in myeloproliferative neoplasms. Leukemia, 22(10):1828–1840, 2008. PMID: 18769448.

[251] AC. Glembotsky, L. Korin, PR. Lev, CD. Chazarreta, RF. Marta, FC. Molinas, and PG. Heller. Screening for mpl mutations in essential thrombocythemia and primary myelofibrosis: normal mpl expression and absence of constitutive stat3 and stat5 activation in mplw515l-positive platelets. Eur J Haematol, 84(5):398–405, 2010. PMID: 20113333.

[252] W. Ma, X. Zhang, X. Wang, Z. Zhang, CH. Yeh, J. Uyeji, and M. Albitar. Mpl mutation profile in jak2 mutation-negative patients with myeloproliferative disorders. Diagn Mol Pathol, 20(1):34–39, 2011. PMID: 21326037.

[253] Z. Wu, X. Zhang, X. Xu, Y. Chen, T. Hu, Z. Kang, S. Li, H. Wang, W. Liu, X. Ma, and M. Guan. The mutation profile of jak2 and calr in chinese han patients with philadelphia chromosome-negative myeloproliferative neoplasms. J Hematol Oncol, 7:48, 2014. PMCID: PMC4223390, PMID: 25023898.

[254] J. Yu, Z. Huang, and JY. Fan. [akt is a therapeutic target in myeloproliferative neoplasms]. Zhongguo Shi Yan Xue Ye Xue Za Zhi, 25(4):1105–1112, 2017. PMID:

28823277.

[255] J. Xie, X. Chen, F. Gao, R. Hou, T. Tian, Y. Zhang, L. Fan, J. Hu, G. Zhu, W. Yang, and H. Wang. Two activating mutations of mpl in triple-negative myeloproliferative neoplasms. Cancer Med, 8(11):5254–5263, 2019. PMCID: PMC6718619, PMID: 31294534.

[256] X. Qin, Y. Guo, H. Du, Y. Zhong, J. Zhang, X. Li, H. Yu, Z. Zhang, Z. Jia, and Z. Li. Comparative analysis for glycopatterns and complex-type n-glycans of glycoprotein in sera from chronic hepatitis b- and c-infected patients. Front Physiol, 8:596, 2017. PMCID: PMC5566988, PMID: 28871230.

[257] AR. Moliterno, DM. Williams, LI. Gutierrez-Alamillo, R. Salvatori, RG. Ingersoll, and JL. Spivak. Mpl baltimore: a thrombopoietin receptor polymorphism associated with thrombocytosis. Proc Natl Acad Sci U S A, 101(31): 11444–11447, 2004. PMCID: PMC509220, PMID: 15269348.

[258] DL. Bodian, JN. McCutcheon, P. Kothiyal, KC. Huddleston, RK. Iyer, JG. Vockley, and JE. Niederhuber. Germline variation in cancer-susceptibility genes in a healthy, ancestrally diverse cohort: implications for individual genome sequencing. PLoS One, 9(4):e94554, 2014. PMCID: PMC3984285, PMID: 24728327.

[259] DS. Kim, AA. Burt, JE. Ranchalis, B. Wilmot, JD. Smith, KE. Patterson, BP. Coe, YK. Li, MJ. Bamshad, M. Nikolas, EE. Eichler, JM. Swanson, JT. Nigg, DA. Nickerson, and GP. Jarvik. Sequencing of sporadic attention-deficit hyperactivity disorder (adhd) identifies novel and potentially pathogenic de novo variants and excludes overlap with genes associated with autism spectrum disorder. Am J Med Genet B Neuropsychiatr Genet, 174(4):381–389, 2017. PMCID: PMC5467442, PMID: 28332277.

[260] MS. Leduc, Z. Niu, W. Bi, W. Zhu, I. Miloslavskaya, T. Chiang, H. Streff, JR. Seavitt, SA. Murray, C. Eng, A. Chan, Y. Yang, and SR. Lalani. Cript exonic deletion and a novel missense mutation in a female with short stature, dysmorphic features, microcephaly, and pigmentary abnormalities. Am J Med Genet A, 170(8): 2206–2211, 2016. PMCID: PMC5725961, PMID: 27250922.

[261] Y. Ciribilli, P. Singh, A. Inga, and J. Borlak. c-myc targeted regulators of cell metabolism in a transgenic mouse model of papillary lung adenocarcinoma. Oncotarget, 7(40):65514–65539, 2016. PMCID: PMC5323172, PMID: 27602772.

[262] K. Warnatz, U. Salzer, M. Rizzi, B. Fischer, S. Gutenberger, J. Böhm, AK. Kienzler, Q. Pan-Hammarström, L. Hammarström, M. Rakhmanov, M. Schlesier, B. Grimbacher, HH. Peter, and H. Eibel. B-cell activating factor receptor deficiency is associated with an adult-onset antibody deficiency syndrome in humans. Proc Natl Acad Sci U S A, 106(33):13945–13950, 2009. PMCID: PMC2722504, PMID: 19666484.

[263] F. Vetrini, S. McKee, JA. Rosenfeld, M. Suri, AM. Lewis, KM. Nugent, E. Roeder, RO. Littlejohn, S. Holder, W. Zhu, JT. Alaimo, B. Graham, JM. Harris, JB. Gibson, M. Pastore, KL. McBride, M. Komara, L. Al-Gazali, A. Al Shamsi, EA. Fanning, KJ. Wierenga, DA. Scott, Z. Ben-Neriah, V. Meiner, H. Cassuto, O. Elpeleg, JL. Holder, LC. Burrage, LH. Seaver, L. Van Maldergem, S. Mahida, JS. Soul, M. Marlatt, L. Matyakhina, J. Vogt, JA. Gold, SM. Park, V. Varghese, AK. Lampe, A. Kumar, M. Lees, M. Holder-

Espinasse, V. McConnell, B. Bernhard, E. Blair, V. Harrison, DM. Muzny, RA. Gibbs, SH. Elsea, JE. Posey, W. Bi, S. Lalani, F. Xia, Y. Yang, CM. Eng, JR. Lupski, and P. Liu. De novo and inherited tcf20 pathogenic variants are associated with intellectual disability, dysmorphic features, hypotonia, and neurological impairments with similarities to smith-magenis syndrome. Genome Med, 11(1):12, 2019. PMCID: PMC6393995, PMID: 30819258.

[264] Y. Huang, L. Yang, J. Wang, F. Yang, Y. Xiao, R. Xia, X. Yuan, and M. Yan. Twelve novel atm mutations identified in chinese ataxia telangiectasia patients. Neuromolecular Med, 15(3):536–540, 2013. PMCID: PMC3732755, PMID: 23807571.

[265] MJ. Podralska, A. Stembalska, R. Ślezak, ˛ A. Lewandowicz-Uszyńska, B. Pietrucha, S. Kołtan, J. Wigowska-Sowińska, J. Pilch, M. Mosor, I. Ziółkowska-Suchanek, A. Dzikiewicz-Krawczyk, and R. Słomski. Ten new atm alterations in polish patients with ataxia-telangiectasia. Mol Genet Genomic Med, 2(6):504–511, 2014. PMCID: PMC4303220, PMID: 25614872.

[266] SR. McWhinney, RT. Pilarski, SR. Forrester, MC. Schneider, MM. Sarquis, EP. Dias, and C. Eng. Large germline deletions of mitochondrial complex ii subunits sdhb and sdhd in hereditary paraganglioma. J Clin Endocrinol Metab, 89(11):5694–5699, 2004. PMID: 15531530.

[267] JA. Wambach, GM. Stettner, TB. Haack, K. Writzl, A. Škofljanec, A. Maver, F. Munell, S. Ossowski, M. Bosio, DJ. Wegner, M. Shinawi, D. Baldridge, B. Alhaddad, TM. Strom, DK. Grange, E. Wilichowski, R. Troxell, J. Collins, BB. Warner, RE. Schmidt, A. Pestronk, FS. Cole, and R. Steinfeld. Survival among children with "lethal" congenital contracture syndrome 11 caused by novel mutations in the gliomedin gene (gldn). Hum Mutat, 38(11):1477–1484, 2017. PMCID: PMC5638693, PMID: 28726266.

[268] M. Pergande, S. Motameny, O. Özdemir, M. Kreutzer, H. Wang, HS. Daimagüler, K. Becker, M. Karakaya, H. Ehrhardt, N. Elcioglu, S. Ostojic, CM. Chao, A. Kawalia, O. Duman, A. Koy, A. Hahn, J. Reimann, K. Schoner, A. Schänzer, JH. Westhoff, EMC. Schwaibold, M. Cossee, M. Imbert-Bouteille, H. von Pein, G. Haliloglu, H. Topaloglu, J. Altmüller, P. Nürnberg, H. Thiele, R. Heller, and S. Cirak. The genomic and clinical landscape of fetal akinesia. Genet Med, 22(3):511–523, 2020. PMID: 31680123.

[269] AI. den Hollander, RK. Koenekoop, MD. Mohamed, HH. Arts, K. Boldt, KV. Towns, T. Sedmak, M. Beer, K. Nagel-Wolfrum, M. McKibbin, S. Dharmaraj, I. Lopez, L. Ivings, GA. Williams, K. Springell, CG. Woods, H. Jafri, Y. Rashid, TM. Strom, B. van der Zwaag, I. Gosens, FF. Kersten, E. van Wijk, JA. Veltman, MN. Zonneveld, SE. van Beersum, IH. Maumenee, U. Wolfrum, ME. Cheetham, M. Ueffing, FP. Cremers, CF. Inglehearn, and R. Roepman. Mutations in lca5, encoding the ciliary protein lebercilin, cause leber congenital amaurosis. Nat Genet, 39(7):889–895, 2007. PMID: 17546029.

[270] DS. Mackay, AD. Borman, R. Sui, LI. van den Born, EL. Berson, LA. Ocaka, AE. Davidson, JR. Heckenlively, K. Branham, H. Ren, I. Lopez, M. Maria, M. Azam, A. Henkes, E. Blokland, R. Qamar, AR. Webster, FPM. Cremers, AT. Moore, RK. Koenekoop, S. Andreasson, E. de Baere, J. Bennett, GJ. Chader, W. Berger, I. Golovleva, J. Greenberg, AI. den Hollander, CCW. Klaver, BJ. Klevering, B. Lorenz, MN. Preising, R. Ramsear, L. Roberts, R. Roepman, K. Rohrschneider, and B. Wissinger.

Screening of a large cohort of leber congenital amaurosis and retinitis pigmentosa patients identifies novel lca5 mutations and new genotype-phenotype correlations. Hum Mutat, 34(11):1537–1546, 2013. PMCID: PMC4337959, PMID: 23946133.

[271] KJ. Carss, G. Arno, M. Erwood, J. Stephens, A. Sanchis-Juan, S. Hull, K. Megy, D. Grozeva, E. Dewhurst, S. Malka, V. Plagnol, C. Penkett, K. Stirrups, R. Rizzo, G. Wright, D. Josifova, M. Bitner-Glindzicz, RH. Scott, E. Clement, L. Allen, R. Armstrong, AF. Brady, J. Carmichael, M. Chitre, RHH. Henderson, J. Hurst, RE. MacLaren, E. Murphy, J. Paterson, E. Rosser, DA. Thompson, E. Wakeling, WH. Ouwehand, M. Michaelides, AT. Moore, AR. Webster, and FL. Raymond. Comprehensive rare variant analysis via whole-genome sequencing to determine the molecular pathology of inherited retinal disease. Am J Hum Genet, 100(1):75–90, 2017. PMCID: PMC5223092, PMID: 28041643.

[272] SG. Jacobson, TS. Aleman, AV. Cideciyan, A. Sumaroka, SB. Schwartz, EA. Windsor, M. Swider, W. Herrera, and EM. Stone. Leber congenital amaurosis caused by lebercilin (lca5) mutation: retained photoreceptors adjacent to retinal disorganization. Mol Vis, 15:1098–1106, 2009. PMCID: PMC2690955, PMID: 19503738.

[273] MP. Adam, J. Feldman, GM. Mirzaa, RA. Pagon, SE. Wallace, LJH. Bean, KW. Gripp, A. Amemiya, RG. Weleber, PJ. Francis, KM. Trzupek, and C. Beattie. 1993. PMCID: PMC3647373, PMID: 20301475.

[274] K. Boldt, DA. Mans, J. Won, J. van Reeuwijk, A. Vogt, N. Kinkl, SJ. Letteboer, WL. Hicks, RE. Hurd, JK. Naggert, Y. Texier, AI. den Hollander, RK. Koenekoop, J. Bennett, FP. Cremers, CJ. Gloeckner, PM. Nishina, R. Roepman, and M. Ueffing. Disruption of intraflagellar protein transport in photoreceptor cilia causes leber congenital amaurosis in humans and mice. J Clin Invest, 121(6):2169–2180, 2011. PMCID: PMC3104757, PMID: 21606596.

[275] KD. Farwell, L. Shahmirzadi, D. El-Khechen, Z. Powis, EC. Chao, B. Tippin Davis, RM. Baxter, W. Zeng, C. Mroske, MC. Parra, SK. Gandomi, I. Lu, X. Li, H. Lu, HM. Lu, D. Salvador, D. Ruble, M. Lao, S. Fischbach, J. Wen, S. Lee, A. Elliott, CL. Dunlop, and S. Tang. Enhanced utility of family-centered diagnostic exome sequencing with inheritance model-based analysis: results from 500 unselected families with undiagnosed genetic conditions. Genet Med, 17(7):578–586, 2015. PMID: 25356970.

[276] N. Akizu, V. Cantagrel, MS. Zaki, L. Al-Gazali, X. Wang, RO. Rosti, E. Dikoglu, AB. Gelot, B. Rosti, KK. Vaux, EM. Scott, JL. Silhavy, J. Schroth, B. Copeland, AE. Schaffer, PL. Gordts, JD. Esko, MD. Buschman, SJ. Field, G. Napolitano, GM. Abdel-Salam, RK. Ozgul, MS. Sagıroglu, M. Azam, S. Ismail, M. Aglan, L. Selim, IG. Mahmoud, S. Abdel-Hadi, AE. Badawy, AA. Sadek, F. Mojahedi, H. Kayserili, A. Masri, L. Bastaki, S. Temtamy, U. Müller, I. Desguerre, JL. Casanova, A. Dursun, M. Gunel, SB. Gabriel, P. de Lonlay, and JG. Gleeson. Biallelic mutations in snx14 cause a syndromic form of cerebellar atrophy and lysosome-autophagosome dysfunction. Nat Genet, 47(5):528–534, 2015. PMCID: PMC4414867, PMID: 25848753.

[277] AS. Ho, K. Kannan, DM. Roy, LG. Morris, I. Ganly, N. Katabi, D. Ramaswami, LA. Walsh, S. Eng, JT. Huse, J. Zhang, I. Dolgalev, K. Huberman, A. Heguy, A. Viale, M. Drobnjak, MA. Leversha, CE. Rice, B. Singh, NG. Iyer, CR. Leemans, E. Bloemena, RL. Ferris,

RR. Seethala, BE. Gross, Y. Liang, R. Sinha, L. Peng, BJ. Raphael, S. Turcan, Y. Gong, N. Schultz, S. Kim, S. Chiosea, JP. Shah, C. Sander, W. Lee, and TA. Chan. The mutational landscape of adenoid cystic carcinoma. Nat Genet, 45(7):791–798, 2013. PMCID: PMC3708595, PMID: 23685749.

[278] K. Kataoka, Y. Nagata, A. Kitanaka, Y. Shiraishi, T. Shimamura, J. Yasunaga, Y. Totoki, K. Chiba, A. Sato-Otsubo, G. Nagae, R. Ishii, S. Muto, S. Kotani, Y. Watatani, J. Takeda, M. Sanada, H. Tanaka, H. Suzuki, Y. Sato, Y. Shiozawa, T. Yoshizato, K. Yoshida, H. Makishima, M. Iwanaga, G. Ma, K. Nosaka, M. Hishizawa, H. Itonaga, Y. Imaizumi, W. Munakata, H. Ogasawara, T. Sato, K. Sasai, K. Muramoto, M. Penova, T. Kawaguchi, H. Nakamura, N. Hama, K. Shide, Y. Kubuki, T. Hidaka, T. Kameda, T. Nakamaki, K. Ishiyama, S. Miyawaki, SS. Yoon, K. Tobinai, Y. Miyazaki, A. Takaori-Kondo, F. Matsuda, K. Takeuchi, O. Nureki, H. Aburatani, T. Watanabe, T. Shibata, M. Matsuoka, S. Miyano, K. Shimoda, and S. Ogawa. Integrated molecular analysis of adult t cell leukemia/lymphoma. Nat Genet, 47(11):1304–1315, 2015. PMID: 26437031.

[279] JD. Campbell, A. Alexandrov, J. Kim, J. Wala, AH. Berger, CS. Pedamallu, SA. Shukla, G. Guo, AN. Brooks, BA. Murray, M. Imielinski, X. Hu, S. Ling, R. Akbani, M. Rosenberg, C. Cibulskis, A. Ramachandran, EA. Collisson, DJ. Kwiatkowski, MS. Lawrence, JN. Weinstein, RG. Verhaak, CJ. Wu, PS. Hammerman, AD. Cherniack, G. Getz, MN. Artyomov, R. Schreiber, R. Govindan, and M. Meyerson. Distinct patterns of somatic genome alterations in lung adenocarcinomas and squamous cell carcinomas. Nat Genet, 48(6):607–616, 2016. PMCID: PMC4884143, PMID: 27158780.

[280] RN. Doan, ET. Lim, S. De Rubeis, C. Betancur, DJ. Cutler, AG. Chiocchetti, LM. Overman, A. Soucy, S. Goetze, CM. Freitag, MJ. Daly, CA. Walsh, JD. Buxbaum, and TW. Yu. Recessive gene disruptions in autism spectrum disorder. Nat Genet, 51(7):1092–1098, 2019. PMCID: PMC6629034, PMID: 31209396.

[281] K. Niizuma, H. Endo, C. Nito, DJ. Myer, and PH. Chan. Potential role of puma in delayed death of hippocampal ca1 neurons after transient global cerebral ischemia. Stroke, 40(2):618–625, 2009. PMCID: PMC2631621, PMID: 19095966.

[282] SP. Garrison, JR. Jeffers, C. Yang, JA. Nilsson, MA. Hall, JE. Rehg, W. Yue, J. Yu, L. Zhang, M. Onciu, JT. Sample, JL. Cleveland, and GP. Zambetti. Selection against puma gene expression in myc-driven b-cell lymphomagenesis. Mol Cell Biol, 28(17):5391–5402, 2008. PMCID: PMC2519737, PMID: 18573879.

[283] H. Wang, H. Qian, J. Yu, X. Zhang, L. Zhang, M. Fu, X. Liang, Q. Zhan, and C. Lin. Administration of puma adenovirus increases the sensitivity of esophageal cancer cells to anticancer drugs. Cancer Biol Ther, 5(4): 380–385, 2006. PMID: 16481741.

[284] SY. Shi, CT. Luk, SA. Schroer, MJ. Kim, DW. Dodington, T. Sivasubramaniyam, L. Lin, EP. Cai, SY. Lu, KU. Wagner, RP. Bazinet, and M. Woo. Janus kinase 2 (jak2) dissociates hepatosteatosis from hepatocellular carcinoma in mice. J Biol Chem, 292(9):3789–3799, 2017. PMCID: PMC5339761, PMID: 28100771.

[285] A. Driss, S. Noguchi, R. Amouri, M. Kefi, T. Sasaki, K. Sugie, S. Souilem, YK. Hayashi, N. Shimizu, S. Minoshima, J. Kudoh, F. Hentati, and I. Nishino. Fukutin-related protein gene mutated in the original kindred limb-girdle md 2i. Neurology, 60(8):1341–1344,

2003. PMID: 12707439.

[286] S. Quijano-Roy, I. Martí-Carrera, S. Makri, M. Mayer, S. Maugenre, P. Richard, C. Berard, L. Viollet, B. Leheup, P. Guicheney, JM. Pinard, B. Estournet, and RY. Carlier. Brain mri abnormalities in muscular dystrophy due to fkrp mutations. Brain Dev, 28(4):232–242, 2006. PMID: 16368217.

[287] XQ. Rosales, SJ. Moser, T. Tran, B. McCarthy, N. Dunn, P. Habib, OP. Simonetti, JR. Mendell, and SV. Raman. Cardiovascular magnetic resonance of cardiomyopathy in limb girdle muscular dystrophy 2b and 2i. J Cardiovasc Magn Reson, 13(1):39, 2011. PMCID: PMC3170213, PMID: 21816046.

[288] P. Hafner, U. Bonati, A. Fischmann, J. Schneider, S. Frank, DJ. Morris-Rosendahl, A. Dumea, K. Heinimann, and D. Fischer. Skeletal muscle mri of the lower limbs in congenital muscular dystrophy patients with novel pomt1 and pomt2 mutations. Neuromuscul Disord, 24(4):321–324, 2014. PMID: 24556424.

[289] A. Abulí, M. Boada, B. Rodríguez-Santiago, B. Coroleu, A. Veiga, L. Armengol, PN. Barri, LA. Pérez-Jurado, and X. Estivill. Ngs-based assay for the identification of individuals carrying recessive genetic mutations in reproductive medicine. Hum Mutat, 37(6):516–523, 2016. PMID: 26990548.

[290] M. Brockington, Y. Yuva, P. Prandini, SC. Brown, S. Torelli, MA. Benson, R. Herrmann, LV. Anderson, R. Bashir, JM. Burgunder, S. Fallet, N. Romero, M. Fardeau, V. Straub, G. Storey, C. Pollitt, I. Richard, CA. Sewry, K. Bushby, T. Voit, DJ. Blake, and F. Muntoni. Mutations in the fukutin-related protein gene (fkrp) identify limb girdle muscular dystrophy 2i as a milder allelic variant of congenital muscular dystrophy mdc1c. Hum Mol Genet, 10(25):2851–2859, 2001. PMID: 11741828.

[291] E. Mercuri, M. Brockington, V. Straub, S. Quijano-Roy, Y. Yuva, R. Herrmann, SC. Brown, S. Torelli, V. Dubowitz, DJ. Blake, NB. Romero, B. Estournet, CA. Sewry, P. Guicheney, T. Voit, and F. Muntoni. Phenotypic spectrum associated with mutations in the fukutin-related protein gene. Ann Neurol, 53(4):537–542, 2003. PMID: 12666124.

[292] F. de Paula, N. Vieira, A. Starling, LU. Yamamoto, B. Lima, R. de Cássia Pavanello, M. Vainzof, V. Nigro, and M. Zatz. Asymptomatic carriers for homozygous novel mutations in the fkrp gene: the other end of the spectrum. Eur J Hum Genet, 11(12):923–930, 2003. PMID: 14647208.

[293] MC. Walter, JA. Petersen, R. Stucka, D. Fischer, R. Schröder, M. Vorgerd, A. Schroers, H. Schreiber, CO. Hanemann, U. Knirsch, A. Rosenbohm, A. Huebner, N. Barisic, R. Horvath, S. Komoly, P. Reilich, W. Müller-Felber, D. Pongratz, JS. Müller, EA. Auerswald, and H. Lochmüller. Fkrp (826c>a) frequently causes limb-girdle muscular dystrophy in german patients. J Med Genet, 41(4):e50, 2004. PMCID: PMC1735747, PMID: 15060126.

[294] CT. Esapa, RA. McIlhinney, and DJ. Blake. Fukutin-related protein mutations that cause congenital muscular dystrophy result in er-retention of the mutant protein in cultured cells. Hum Mol Genet, 14(2):295–305, 2005. PMID: 15574464.

[295] P. Frosk, CR. Greenberg, AA. Tennese, R. Lamont, E. Nylen, C. Hirst, D. Frappier, NM.

Roslin, M. Zaik, K. Bushby, V. Straub, M. Zatz, F. de Paula, K. Morgan, TM. Fujiwara, and K. Wrogemann. The most common mutation in fkrp causing limb girdle muscular dystrophy type 2i (lgmd2i) may have occurred only once and is present in hutterites and other populations. Hum Mutat, 25(1):38–44, 2005. PMID: 15580560.

[296] T. Müller, M. Krasnianski, R. Witthaut, M. Deschauer, and S. Zierz. Dilated cardiomyopathy may be an early sign of the c826a fukutin-related protein mutation. Neuromuscul Disord, 15(5):372–376, 2005. PMID: 15833432.

[297] M. Schwartz, JM. Hertz, ML. Sveen, and J. Vissing. Lgmd2i presenting with a characteristic duchenne or becker muscular dystrophy phenotype. Neurology, 64(9):1635–1637, 2005. PMID: 15883334.

[298] P. Frosk, MR. Del Bigio, K. Wrogemann, and CR. Greenberg. Hutterite brothers both affected with two forms of limb girdle muscular dystrophy: Lgmd2h and lgmd2i. Eur J Hum Genet, 13(8):978–982, 2005. PMID: 15886712.

[299] ML. Sveen, M. Schwartz, and J. Vissing. High prevalence and phenotype-genotype correlations of limb girdle muscular dystrophy type 2i in denmark. Ann Neurol, 59(5):808–815, 2006. PMID: 16634037.

[300] C. Gaul, M. Deschauer, C. Tempelmann, S. Vielhaber, HU. Klein, HJ. Heinze, S. Zierz, and F. Grothues. Cardiac involvement in limb-girdle muscular dystrophy 2i : conventional cardiac diagnostic and cardiovascular magnetic resonance. J Neurol, 253(10):1317–1322, 2006. PMID: 16786213.

[301] E. Keramaris-Vrantsis, PJ. Lu, T. Doran, A. Zillmer, J. Ashar, CT. Esapa, MA. Benson, DJ. Blake, J. Rosenfeld, and QL. Lu. Fukutin-related protein localizes to the golgi apparatus and mutations lead to mislocalization in muscle in vivo. Muscle Nerve, 36(4):455–465, 2007. PMID: 17554798.

[302] A. D'Amico, S. Petrini, F. Parisi, A. Tessa, P. Francalanci, G. Grutter, FM. Santorelli, and E. Bertini. Heart transplantation in a child with lgmd2i presenting as isolated dilated cardiomyopathy. Neuromuscul Disord, 18(2): 153–155, 2008. PMID: 18060779.

[303] P. Reilich, JA. Petersen, S. Vielhaber, C. Mawrin, C. Schneider-Gold, C. Sommer, K. Reiners, M. Deschauer, D. Pongratz, H. Lochmüller, and MC. Walter. Lgmd 2i due to the common mutation 826c>a in the fkrp gene presenting as myopathy with vacuoles and paired-helical filaments. Acta Myol, 25(2):73–76, 2006. PMID: 18593008.

[304] K. Wahbi, C. Meune, el H. Hamouda, T. Stojkovic, P. Laforêt, HM. Bécane, B. Eymard, and D. Duboc. Cardiac assessment of limb-girdle muscular dystrophy 2i patients: an echography, holter ecg and magnetic resonance imaging study. Neuromuscul Disord, 18(8):650–655, 2008. PMID: 18639457.

[305] F. Hanisch, D. Grimm, S. Zierz, and M. Deschauer. Frequency of the fkrp mutation c.826c>a in isolated hyperckemia and in limb girdle muscular dystrophy type 2 in german patients. J Neurol, 257(2):300–301, 2010. PMID: 19820980.

[306] RR. Bennett, HE. Schneider, E. Estrella, S. Burgess, AS. Cheng, C. Barrett, V. Lip, PS. Lai, Y. Shen, BL. Wu, BT. Darras, AH. Beggs, and LM. Kunkel. Automated dna mutation

detection using universal conditions direct sequencing: application to ten muscular dystrophy genes. BMC Genet, 10:66, 2009. PMCID: PMC2781300, PMID: 19835634.

[307] PJ. Lu, A. Zillmer, X. Wu, H. Lochmuller, J. Vachris, D. Blake, YM. Chan, and QL. Lu. Mutations alter secretion of fukutin-related protein. Biochim Biophys Acta, 1802(2):253–258, 2010. PMID: 19900540.

[308] E. Stensland, S. Lindal, C. Jonsrud, T. Torbergsen, LA. Bindoff, M. Rasmussen, A. Dahl, F. Thyssen, and O. Nilssen. Prevalence, mutation spectrum and phenotypic variability in norwegian patients with limb girdle muscular dystrophy 2i. Neuromuscul Disord, 21(1):41–46, 2011. PMID: 20961759.

[309] KD. Mathews, CM. Stephan, K. Laubenthal, TL. Winder, DE. Michele, SA. Moore, and KP. Campbell. Myoglobinuria and muscle pain are common in patients with limb-girdle muscular dystrophy 2i. Neurology, 76 (2):194–195, 2011. PMCID: PMC3030231, PMID: 21220724.

[310] CJ. Bell, DL. Dinwiddie, NA. Miller, SL. Hateley, EE. Ganusova, J. Mudge, RJ. Langley, L. Zhang, CC. Lee, FD. Schilkey, V. Sheth, JE. Woodward, HE. Peckham, GP. Schroth, RW. Kim, and SF. Kingsmore. Carrier testing for severe childhood recessive diseases by next-generation sequencing. Sci Transl Med, 3(65):65ra4, 2011. PMCID: PMC3740116, PMID: 21228398.

[311] D. Renard, C. Fernandez, C. Bouchet-Seraphin, and P. Labauge. Cortical heterotopia in lgmd2i. Neuromuscul Disord, 22(5):443–444, 2012. PMID: 22264518.

[312] JX. Chong, R. Ouwenga, RL. Anderson, DJ. Waggoner, and C. Ober. A population-based study of autosomal-recessive disease-causing mutations in a founder population. Am J Hum Genet, 91(4):608–620, 2012. PMCID: PMC3484657, PMID: 22981120.

[313] KG. Hollingsworth, TA. Willis, MG. Bates, BJ. Dixon, H. Lochmüller, K. Bushby, J. Bourke, GA. MacGowan, and V. Straub. Subepicardial dysfunction leads to global left ventricular systolic impairment in patients with limb girdle muscular dystrophy 2i. Eur J Heart Fail, 15(9):986–994, 2013. PMID: 23576288.

[314] A. Blaeser, E. Keramaris, YM. Chan, S. Sparks, D. Cowley, X. Xiao, and QL. Lu. Mouse models of fukutin-related protein mutations show a wide range of disease phenotypes. Hum Genet, 132(8):923–934, 2013. PMID: 23591631.

[315] H. Duzkale, J. Shen, H. McLaughlin, A. Alfares, MA. Kelly, TJ. Pugh, BH. Funke, HL. Rehm, and MS. Lebo. A systematic approach to assessing the clinical significance of genetic variants. Clin Genet, 84(5):453–463, 2013. PMCID: PMC3995020, PMID: 24033266.

[316] M. Rasmussen, D. Scheie, N. Breivik, M. Mork, and S. Lindal. Clinical and muscle biopsy findings in norwegian paediatric patients with limb girdle muscular dystrophy 2i. Acta Paediatr, 103(5):553–558, 2014. PMID: 24447024.

[317] C. Qiao, CH. Wang, C. Zhao, P. Lu, H. Awano, B. Xiao, J. Li, Z. Yuan, Y. Dai, CB. Martin, J. Li, Q. Lu, and X. Xiao. Muscle and heart function restoration in a limb girdle muscular dystrophy 2i (lgmd2i) mouse model by systemic fkrp gene delivery. Mol Ther,

22(11):1890–1899, 2014. PMCID: PMC4429733, PMID: 25048216.

[318] LV. Schottlaender, A. Petzold, N. Wood, and H. Houlden. Diagnostic clues and manifesting carriers in fukutin-related protein (fkrp) limb-girdle muscular dystrophy. J Neurol Sci, 348(1-2):266–268, 2015. PMID: 25560911.

[319] J. Svahn, N. Streichenberger, O. Benveniste, R. Menassa, L. Michel, H. Fayolle, and P. Petiot. Significant response to immune therapies in a case of subacute necrotizing myopathy and fkrp mutations. Neuromuscul Disord, 25(11):865–868, 2015. PMID: 26363967.

[320] TO. Krag and J. Vissing. A new mouse model of limb-girdle muscular dystrophy type 2i homozygous for the common l276i mutation mimicking the mild phenotype in humans. J Neuropathol Exp Neurol, 74(12): 1137–1146, 2015. PMID: 26574668.

[321] SM. Yap, M. Farrell, J. Cryan, and S. Smyth. An irish case of limb-girdle muscular dystrophy 2i with structural eye involvement. Muscle Nerve, 54(3):509–510, 2016. PMID: 26833294.

[322] L. Ten Dam, WS. Frankhuizen, WHJP. Linssen, CS. Straathof, EH. Niks, K. Faber, A. Fock, JB. Kuks, E. Brusse, R. de Coo, N. Voermans, A. Verrips, JE. Hoogendijk, L. van der Pol, D. Westra, M. de Visser, AJ. van der Kooi, and I. Ginjaar. Autosomal recessive limb-girdle and miyoshi muscular dystrophies in the netherlands: The clinical and molecular spectrum of 244 patients. Clin Genet, 96(2):126–133, 2019. PMID: 30919934.

[323] M. Brockington, DJ. Blake, P. Prandini, SC. Brown, S. Torelli, MA. Benson, CP. Ponting, B. Estournet, NB. Romero, E. Mercuri, T. Voit, CA. Sewry, P. Guicheney, and F. Muntoni. Mutations in the fukutin-related protein gene (fkrp) cause a form of congenital muscular dystrophy with secondary laminin alpha2 deficiency and abnormal glycosylation of alpha-dystroglycan. Am J Hum Genet, 69(6):1198–1209, 2001. PMCID: PMC1235559, PMID: 11592034.

[324] CT. Esapa, MA. Benson, JE. Schröder, E. Martin-Rendon, M. Brockington, SC. Brown, F. Muntoni, S. Kröger, and DJ. Blake. Functional requirements for fukutin-related protein in the golgi apparatus. Hum Mol Genet, 11 (26):3319–3331, 2002. PMID: 12471058.

[325] D. Atac, S. Koller, JVM. Hanson, S. Feil, A. Tiwari, A. Bahr, L. Baehr, I. Magyar, R. Kottke, C. Gerth-Kahlert, and W. Berger. Atonal homolog 7 (atoh7) loss-of-function mutations in predominant bilateral optic nerve hypoplasia. Hum Mol Genet, 29(1):132–148, 2020. PMID: 31696227.

[326] C. Chen, X. Fu, D. Zhang, Y. Li, Y. Xie, Y. Li, and Y. Huang. Varied pathways of stage ia lung adenocarcinomas discovered by integrated gene expression analysis. Int J Biol Sci, 7(5):551–566, 2011. PMCID: PMC3088877, PMID: 21552421.

[327] NA. Bersinger, DM. Wunder, MH. Birkhäuser, and MD. Mueller. Gene expression in cultured endometrium from women with different outcomes following ivf. Mol Hum Reprod, 14(8):475–484, 2008. PMID: 18539642.

[328] LR. Shiow, DW. Roadcap, K. Paris, SR. Watson, IL. Grigorova, T. Lebet, J. An, Y. Xu, CN. Jenne, N. Föger, RU. Sorensen, CC. Goodnow, JE. Bear, JM. Puck, and JG. Cyster.

The actin regulator coronin 1a is mutant in a thymic egress-deficient mouse strain and in a patient with severe combined immunodeficiency. Nat Immunol, 9 (11):1307–1315, 2008. PMCID: PMC2672406, PMID: 18836449.

[329] A. Stray-Pedersen, E. Jouanguy, A. Crequer, AA. Bertuch, BS. Brown, SN. Jhangiani, DM. Muzny, T. Gambin, H. Sorte, G. Sasa, D. Metry, J. Campbell, MM. Sockrider, MK. Dishop, DM. Scollard, RA. Gibbs, EM. Mace, JS. Orange, JR. Lupski, JL. Casanova, and LM. Noroski. Compound heterozygous coro1a mutations in siblings with a mucocutaneous-immunodeficiency syndrome of epidermodysplasia verruciformis-hpv, molluscum contagiosum and granulomatous tuberculoid leprosy. J Clin Immunol, 34(7):871–890, 2014. PMCID: PMC4386834, PMID: 25073507.

[330] LA. Weiss, Y. Shen, JM. Korn, DE. Arking, DT. Miller, R. Fossdal, E. Saemundsen, H. Stefansson, MA. Ferreira, T. Green, OS. Platt, DM. Ruderfer, CA. Walsh, D. Altshuler, A. Chakravarti, RE. Tanzi, K. Stefansson, SL. Santangelo, JF. Gusella, P. Sklar, BL. Wu, and MJ. Daly. Association between microdeletion and microduplication at 16p11.2 and autism. N Engl J Med, 358(7):667–675, 2008. PMID: 18184952.

[331] DP. Moreira, K. Griesi-Oliveira, AL. Bossolani-Martins, NC. Lourenço, VN. Takahashi, KM. da Rocha, ES. Moreira, E. Vadasz, JG. Meira, D. Bertola, E. O'Halloran, TR. Magalhães, AC. Fett-Conte, and MR. Passos-Bueno. Investigation of 15q11-q13, 16p11.2 and 22q13 cnvs in autism spectrum disorder brazilian individuals with and without epilepsy. PLoS One, 9(9):e107705, 2014. PMCID: PMC4177849, PMID: 25255310.

[332] V. Krishnan and SL. Zeichner. Host cell gene expression during human immunodeficiency virus type 1 latency and reactivation and effects of targeting genes that are differentially expressed in viral latency. J Virol, 78(17): 9458–9473, 2004. PMCID: PMC506933, PMID: 15308739.

[333] DB. Sparrow, A. McInerney-Leo, ZS. Gucev, B. Gardiner, M. Marshall, PJ. Leo, DL. Chapman, V. Tasic, A. Shishko, MA. Brown, EL. Duncan, and SL. Dunwoodie. Autosomal dominant spondylocostal dysostosis is caused by mutation in tbx6. Hum Mol Genet, 22(8):1625–1631, 2013. PMID: 23335591.

[334] JR. Lupski, C. Gonzaga-Jauregui, Y. Yang, MN. Bainbridge, S. Jhangiani, CJ. Buhay, CL. Kovar, M. Wang, AC. Hawes, JG. Reid, C. Eng, DM. Muzny, and RA. Gibbs. Exome sequencing resolves apparent incidental findings and reveals further complexity of sh3tc2 variant alleles causing charcot-marie-tooth neuropathy. Genome Med, 5(6):57, 2013. PMCID: PMC3706849, PMID: 23806086.

[335] Y. Yang, DM. Muzny, JG. Reid, MN. Bainbridge, A. Willis, PA. Ward, A. Braxton, J. Beuten, F. Xia, Z. Niu, M. Hardison, R. Person, MR. Bekheirnia, MS. Leduc, A. Kirby, P. Pham, J. Scull, M. Wang, Y. Ding, SE. Plon, JR. Lupski, AL. Beaudet, RA. Gibbs, and CM. Eng. Clinical whole-exome sequencing for the diagnosis of mendelian disorders. N Engl J Med, 369(16):1502–1511, 2013. PMCID: PMC4211433, PMID: 24088041.

[336] N. Wu, X. Ming, J. Xiao, Z. Wu, X. Chen, M. Shinawi, Y. Shen, G. Yu, J. Liu, H. Xie, ZS. Gucev, S. Liu, N. Yang, H. Al-Kateb, J. Chen, J. Zhang, N. Hauser, T. Zhang, V. Tasic, P. Liu, X. Su, X. Pan, C. Liu, L. Wang, J. Shen, J. Shen, Y. Chen, T. Zhang, J. Zhang,

KW. Choy, J. Wang, Q. Wang, S. Li, W. Zhou, J. Guo, Y. Wang, C. Zhang, H. Zhao, Y. An, Y. Zhao, J. Wang, Z. Liu, Y. Zuo, Y. Tian, X. Weng, VR. Sutton, H. Wang, Y. Ming, S. Kulkarni, TP. Zhong, PF. Giampietro, SL. Dunwoodie, SW. Cheung, X. Zhang, L. Jin, JR. Lupski, G. Qiu, and F. Zhang. Tbx6 null variants and a common hypomorphic allele in congenital scoliosis. N Engl J Med, 372(4):341–350, 2015. PMCID: PMC4326244, PMID: 25564734.

[337] K. Wang, ST. Yuen, J. Xu, SP. Lee, HH. Yan, ST. Shi, HC. Siu, S. Deng, KM. Chu, S. Law, KH. Chan, AS. Chan, WY. Tsui, SL. Ho, AK. Chan, JL. Man, V. Foglizzo, MK. Ng, AS. Chan, YP. Ching, GH. Cheng, T. Xie, J. Fernandez, VS. Li, H. Clevers, PA. Rejto, M. Mao, and SY. Leung. Whole-genome sequencing and comprehensive molecular profiling identify new driver mutations in gastric cancer. Nat Genet, 46(6):573–582, 2014. PMID: 24816253.

[338] SL. Van Driest, O. Gakh, SR. Ommen, G. Isaya, and MJ. Ackerman. Molecular and functional characterization of a human frataxin mutation found in hypertrophic cardiomyopathy. Mol Genet Metab, 85(4):280–285, 2005. PMID: 15936968.

[339] ML. Jauslin, T. Meier, RA. Smith, and MP. Murphy. Mitochondria-targeted antioxidants protect friedreich ataxia fibroblasts from endogenous oxidative stress more effectively than untargeted antioxidants. FASEB J, 17(13): 1972–1974, 2003. PMID: 12923074.

[340] M. Pandolfo. Frataxin deficiency and mitochondrial dysfunction. Mitochondrion, 2(1-2):87–93, 2002. PMID: 16120311.

[341] RA. Schoenfeld, E. Napoli, A. Wong, S. Zhan, L. Reutenauer, D. Morin, AR. Buckpitt, F. Taroni, B. Lonnerdal, M. Ristow, H. Puccio, and GA. Cortopassi. Frataxin deficiency alters heme pathway transcripts and decreases mitochondrial heme metabolites in mammalian cells. Hum Mol Genet, 14(24):3787–3799, 2005. PMID: 16239244.

[342] KZ. Bencze, KC. Kondapalli, JD. Cook, S. McMahon, C. Millán-Pacheco, N. Pastor, and TL. Stemmler. The structure and function of frataxin. Crit Rev Biochem Mol Biol, 41(5):269–291, 2006. PMCID: PMC2859089, PMID: 16911956.

[343] RB. Wilson. Iron dysregulation in friedreich ataxia. Semin Pediatr Neurol, 13(3):166–175, 2006. PMID: 17101455.

[344] M. Rai, E. Soragni, CJ. Chou, G. Barnes, S. Jones, JR. Rusche, JM. Gottesfeld, and M. Pandolfo. Two new pimelic diphenylamide hdac inhibitors induce sustained frataxin upregulation in cells from friedreich's ataxia patients and in a mouse model. PLoS One, 5(1):e8825, 2010. PMCID: PMC2809102, PMID: 20098685.

[345] K. Kemp, E. Mallam, K. Hares, J. Witherick, N. Scolding, and A. Wilkins. Mesenchymal stem cells restore frataxin expression and increase hydrogen peroxide scavenging enzymes in friedreich ataxia fibroblasts. PLoS One, 6(10):e26098, 2011. PMCID: PMC3189234, PMID: 22016819.

[346] L. Li, L. Voullaire, C. Sandi, MA. Pook, PA. Ioannou, MB. Delatycki, and JP. Sarsero. Pharmacological screening using an fxn-egfp cellular genomic reporter assay for the therapy of friedreich ataxia. PLoS One, 8(2):e55940, 2013. PMCID: PMC3572186, PMID: 23418481.

[347] Y. Shen, MZ. McMackin, Y. Shan, A. Raetz, S. David, and G. Cortopassi. Frataxin deficiency promotes excess microglial dna damage and inflammation that is rescued by pj34. PLoS One, 11(3):e0151026, 2016. PMCID: PMC4783034, PMID: 26954031.

[348] L. Guo, Q. Wang, L. Weng, LA. Hauser, CJ. Strawser, AG. Rocha, A. Dancis, C. Mesaros, DR. Lynch, and IA. Blair. Liquid chromatography-high resolution mass spectrometry analysis of platelet frataxin as a protein biomarker for the rare disease friedreich's ataxia. Anal Chem, 90(3):2216–2223, 2018. PMCID: PMC5817373, PMID: 29272104.

[349] L. Guo, Q. Wang, L. Weng, LA. Hauser, CJ. Strawser, C. Mesaros, DR. Lynch, and IA. Blair. Characterization of a new n-terminally acetylated extra-mitochondrial isoform of frataxin in human erythrocytes. Sci Rep, 8(1): 17043, 2018. PMCID: PMC6242848, PMID: 30451920.

[350] Y. Gui, G. Guo, Y. Huang, X. Hu, A. Tang, S. Gao, R. Wu, C. Chen, X. Li, L. Zhou, M. He, Z. Li, X. Sun, W. Jia, J. Chen, S. Yang, F. Zhou, X. Zhao, S. Wan, R. Ye, C. Liang, Z. Liu, P. Huang, C. Liu, H. Jiang, Y. Wang, H. Zheng, L. Sun, X. Liu, Z. Jiang, D. Feng, J. Chen, S. Wu, J. Zou, Z. Zhang, R. Yang, J. Zhao, C. Xu, W. Yin, Z. Guan, J. Ye, H. Zhang, J. Li, K. Kristiansen, ML. Nickerson, D. Theodorescu, Y. Li, X. Zhang, S. Li, J. Wang, H. Yang, J. Wang, and Z. Cai. Frequent mutations of chromatin remodeling genes in transitional cell carcinoma of the bladder. Nat Genet, 43(9):875–878, 2011. PMCID: PMC5373841, PMID: 21822268.

[351] Z. Zelenko, L. Aghajanova, JC. Irwin, and LC. Giudice. Nuclear receptor, coregulator signaling, and chromatin remodeling pathways suggest involvement of the epigenome in the steroid hormone response of endometrium and abnormalities in endometriosis. Reprod Sci, 19(2):152–162, 2012. PMCID: PMC3343132, PMID: 22138541.

[352] DM. Park, J. Li, H. Okamoto, O. Akeju, SH. Kim, I. Lubensky, A. Vortmeyer, J. Dambrosia, RJ. Weil, EH. Oldfield, JK. Park, and Z. Zhuang. N-cor pathway targeting induces glioblastoma derived cancer stem cell differentiation. Cell Cycle, 6(4):467–470, 2007. PMID: 17312396.

[353] J. Armenia, SAM. Wankowicz, D. Liu, J. Gao, R. Kundra, E. Reznik, WK. Chatila, D. Chakravarty, GC. Han, I. Coleman, B. Montgomery, C. Pritchard, C. Morrissey, CE. Barbieri, H. Beltran, A. Sboner, Z. Zafeiriou, S. Miranda, CM. Bielski, AV. Penson, C. Tolonen, FW. Huang, D. Robinson, YM. Wu, R. Lonigro, LA. Garraway, F. Demichelis, PW. Kantoff, ME. Taplin, W. Abida, BS. Taylor, HI. Scher, PS. Nelson, JS. de Bono, MA. Rubin, CL. Sawyers, AM. Chinnaiyan, N. Schultz, and EM. Van Allen. The long tail of oncogenic drivers in prostate cancer. Nat Genet, 50(5):645–651, 2018. PMCID: PMC6107367, PMID: 29610475.

[354] S. Chen, W. Lu, MF. Yueh, E. Rettenmeier, M. Liu, M. Paszek, J. Auwerx, RT. Yu, RM. Evans, K. Wang, M. Karin, and RH. Tukey. Intestinal ncor1, a regulator of epithelial cell maturation, controls neonatal hyperbilirubinemia. Proc Natl Acad Sci U S A, 114(8):E1432–E1440, 2017. PMCID: PMC5338369, PMID: 28167773.

[355] A. Fujimoto, M. Furuta, Y. Totoki, T. Tsunoda, M. Kato, Y. Shiraishi, H. Tanaka, H. Taniguchi, Y. Kawakami, M. Ueno, K. Gotoh, S. Ariizumi, CP. Wardell, S. Hayami, T. Nakamura, H. Aikata, K. Arihiro, KA. Boroevich, T. Abe, K. Nakano, K. Maejima, A.

Sasaki-Oku, A. Ohsawa, T. Shibuya, H. Nakamura, N. Hama, F. Hosoda, Y. Arai, S. Ohashi, T. Urushidate, G. Nagae, S. Yamamoto, H. Ueda, K. Tatsuno, H. Ojima, N. Hiraoka, T. Okusaka, M. Kubo, S. Marubashi, T. Yamada, S. Hirano, M. Yamamoto, H. Ohdan, K. Shimada, O. Ishikawa, H. Yamaue, K. Chayama, S. Miyano, H. Aburatani, T. Shibata, and H. Nakagawa. Whole-genome mutational landscape and characterization of noncoding and structural mutations in liver cancer. Nat Genet, 48(5): 500–509, 2016. PMID: 27064257.

[356] ZH. Zhang, H. Yamashita, T. Toyama, Y. Yamamoto, T. Kawasoe, M. Ibusuki, S. Tomita, H. Sugiura, S. Kobayashi, Y. Fujii, and H. Iwase. Nuclear corepressor 1 expression predicts response to first-line endocrine therapy for breast cancer patients on relapse. Chin Med J (Engl), 122(15):1764–1768, 2009. PMID: 19781322.

[357] P. Szafranski, GK. Von Allmen, BH. Graham, AA. Wilfong, SH. Kang, JA. Ferreira, SJ. Upton, JB. Moeschler, W. Bi, JA. Rosenfeld, LG. Shaffer, S. Wai Cheung, P. Stankiewicz, and SR. Lalani. 6q22.1 microdeletion and susceptibility to pediatric epilepsy. Eur J Hum Genet, 23(2):173–179, 2015. PMCID: PMC4297903, PMID: 24824130.

[358] S. Hanein, I. Perrault, S. Gerber, G. Tanguy, F. Barbet, D. Ducroq, P. Calvas, H. Dollfus, C. Hamel, T. Lopponen, F. Munier, L. Santos, S. Shalev, D. Zafeiriou, JL. Dufier, A. Munnich, JM. Rozet, and J. Kaplan. Leber congenital amaurosis: comprehensive survey of the genetic heterogeneity, refinement of the clinical definition, and genotype-phenotype correlations as a strategy for molecular diagnosis. Hum Mutat, 23(4):306–317, 2004. PMID: 15024725.

[359] JC. Booij, RJ. Florijn, JB. ten Brink, W. Loves, F. Meire, MJ. van Schooneveld, PT. de Jong, and AA. Bergen. Identification of mutations in the aipl1, crb1, gucy2d, rpe65, and rpgrip1 genes in patients with juvenile retinitis pigmentosa. J Med Genet, 42(11):e67, 2005. PMCID: PMC1735944, PMID: 16272259.

[360] BH. Anderson, PR. Kasher, J. Mayer, M. Szynkiewicz, EM. Jenkinson, SS. Bhaskar, JE. Urquhart, SB. Daly, JE. Dickerson, J. O'Sullivan, EO. Leibundgut, J. Muter, GM. Abdel-Salem, R. Babul-Hirji, P. Baxter, A. Berger, L. Bonafé, JE. Brunstom-Hernandez, JA. Buckard, D. Chitayat, WK. Chong, DM. Cordelli, P. Ferreira, J. Fluss, EH. Forrest, E. Franzoni, C. Garone, SR. Hammans, G. Houge, I. Hughes, S. Jacquemont, PY. Jeannet, RJ. Jefferson, R. Kumar, G. Kutschke, S. Lundberg, CM. Lourenço, R. Mehta, S. Naidu, KK. Nischal, L. Nunes, K. Ounap, M. Philippart, P. Prabhakar, SR. Risen, R. Schiffmann, C. Soh, JB. Stephenson, H. Stewart, J. Stone, JL. Tolmie, MS. van der Knaap, JP. Vieira, CN. Vilain, EL. Wakeling, V. Wermenbol, A. Whitney, SC. Lovell, S. Meyer, JH. Livingston, GM. Baerlocher, GC. Black, GI. Rice, and YJ. Crow. Mutations in ctc1, encoding conserved telomere maintenance component 1, cause coats plus. Nat Genet, 44(3):338–342, 2012. PMID: 22267198.

[361] A. Polvi, T. Linnankivi, T. Kivelä, R. Herva, JP. Keating, O. Mäkitie, D. Pareyson, L. Vainionpää, J. Lahtinen, I. Hovatta, H. Pihko, and AE. Lehesjoki. Mutations in ctc1, encoding the cts telomere maintenance complex component 1, cause cerebroretinal microangiopathy with calcifications and cysts. Am J Hum Genet, 90(3): 540–549, 2012. PMCID: PMC3309194, PMID: 22387016.

[362] MW. Ruijs, S. Verhoef, MA. Rookus, R. Pruntel, AH. van der Hout, FB. Hogervorst, I. Kluijt, RH. Sijmons, CM. Aalfs, A. Wagner, MG. Ausems, N. Hoogerbrugge, CJ. van Asperen, EB. Gomez Garcia, H. Meijers-Heijboer, LP. Ten Kate, FH. Menko, and LJ. van 't Veer. Tp53 germline mutation testing in 180 families suspected of li-fraumeni syndrome: mutation detection rate and relative frequency of cancers in different familial phenotypes. J Med Genet, 47(6):421–428, 2010. PMID: 20522432.

[363] A. Shlien, B. Baskin, MI. Achatz, DJ. Stavropoulos, KE. Nichols, L. Hudgins, CF. Morel, MP. Adam, N. Zhukova, L. Rotin, A. Novokmet, H. Druker, M. Shago, PN. Ray, P. Hainaut, and D. Malkin. A common molecular mechanism underlies two phenotypically distinct 17p13.1 microdeletion syndromes. Am J Hum Genet, 87(5): 631–642, 2010. PMCID: PMC2978979, PMID: 21056402.

[364] Y. Zerdoumi, J. Aury-Landas, C. Bonaïti-Pellié, C. Derambure, R. Sesboüé, M. Renaux-Petel, T. Frebourg, G. Bougeard, and JM. Flaman. Drastic effect of germline tp53 missense mutations in li-fraumeni patients. Hum Mutat, 34(3):453–461, 2013. PMID: 23172776.

[365] LR. Susswein, ML. Marshall, R. Nusbaum, KJ. Vogel Postula, SM. Weissman, L. Yackowski, EM. Vaccari, J. Bissonnette, JK. Booker, ML. Cremona, F. Gibellini, PD. Murphy, DE. Pineda-Alvarez, GD. Pollevick, Z. Xu, G. Richard, S. Bale, RT. Klein, KS. Hruska, and WK. Chung. Pathogenic and likely pathogenic variant prevalence among the first 10,000 patients referred for next-generation cancer panel testing. Genet Med, 18(8): 823–832, 2016. PMCID: PMC4985612, PMID: 26681312.

[366] M. Weigell-Weber, S. Fokstuen, B. Török, G. Niemeyer, A. Schinzel, and M. Hergersberg. Codons 837 and 838 in the retinal guanylate cyclase gene on chromosome 17p: hot spots for mutations in autosomal dominant cone-rod dystrophy? Arch Ophthalmol, 118(2):300, 2000. PMID: 10676808.

[367] SE. Wilkie, RJ. Newbold, E. Deery, CE. Walker, I. Stinton, V. Ramamurthy, JB. Hurley, SS. Bhattacharya, MJ. Warren, and DM. Hunt. Functional characterization of missense mutations at codon 838 in retinal guanylate cyclase correlates with disease severity in patients with autosomal dominant cone-rod dystrophy. Hum Mol Genet, 9(20):3065–3073, 2000. PMID: 11115851.

[368] AM. Payne, AG. Morris, SM. Downes, S. Johnson, AC. Bird, AT. Moore, SS. Bhattacharya, and DM. Hunt. Clustering and frequency of mutations in the retinal guanylate cyclase (gucy2d) gene in patients with dominant cone-rod dystrophies. J Med Genet, 38(9):611–614, 2001. PMCID: PMC1734946, PMID: 11565546.

[369] N. Udar, S. Yelchits, M. Chalukya, V. Yellore, S. Nusinowitz, R. Silva-Garcia, T. Vrabec, I. Hussles Maumenee, L. Donoso, and KW. Small. Identification of gucy2d gene mutations in cord5 families and evidence of incomplete penetrance. Hum Mutat, 21(2):170–171, 2003. PMID: 12552567.

[370] D. Zobor, E. Zrenner, B. Wissinger, S. Kohl, and H. Jägle. Gucy2d- or guca1a-related autosomal dominant cone-rod dystrophy: is there a phenotypic difference? Retina, 34(8):1576–1587, 2014. PMID: 24875811.

[371] F. Jiang, K. Xu, X. Zhang, Y. Xie, F. Bai, and Y. Li. Gucy2d mutations in a chinese cohort with autosomal dominant cone or cone-rod dystrophies. Doc Ophthalmol, 131(2):105–114, 2015. PMID: 26298565.

[372] C. Jespersgaard, M. Fang, M. Bertelsen, X. Dang, H. Jensen, Y. Chen, N. Bech, L. Dai, T. Rosenberg, J. Zhang, LB. Møller, Z. Tümer, K. Brøndum-Nielsen, and K. Grønskov. Molecular genetic analysis using targeted ngs analysis of 677 individuals with retinal dystrophy. Sci Rep, 9(1):1219, 2019. PMCID: PMC6362094, PMID: 30718709.

[373] J. Maggi, S. Koller, L. Bähr, S. Feil, F. Kivrak Pfiffner, JVM. Hanson, A. Maspoli, C. Gerth-Kahlert, and W. Berger. Long-range pcr-based ngs applications to diagnose mendelian retinal diseases. Int J Mol Sci, 22(4), 2021. PMCID: PMC7913364, PMID: 33546218.

[374] G. Dodt, DG. Kim, SA. Reimann, BE. Reuber, K. McCabe, SJ. Gould, and SJ. Mihalik. L-pipecolic acid oxidase, a human enzyme essential for the degradation of l-pipecolic acid, is most similar to the monomeric sarcosine oxidases. Biochem J, (Pt 3):487–494, 2000. PMCID: PMC1220782, PMID: 10642506.

[375] JE. Posey, JA. Rosenfeld, RA. James, M. Bainbridge, Z. Niu, X. Wang, S. Dhar, W. Wiszniewski, ZH. Akdemir, T. Gambin, F. Xia, RE. Person, M. Walkiewicz, CA. Shaw, VR. Sutton, AL. Beaudet, D. Muzny, CM. Eng, Y. Yang, RA. Gibbs, JR. Lupski, E. Boerwinkle, and SE. Plon. Molecular diagnostic experience of whole-exome sequencing in adult patients. Genet Med, 18(7):678–685, 2016. PMCID: PMC4892996, PMID: 26633545.

[376] XD. Hao, P. Chen, YY. Zhang, SX. Li, WY. Shi, and H. Gao. De novo mutations of tuba3d are associated with keratoconus. Sci Rep, 7(1):13570, 2017. PMCID: PMC5648796, PMID: 29051577.

[377] CL. Galligan, E. Baig, V. Bykerk, EC. Keystone, and EN. Fish. Distinctive gene expression signatures in rheumatoid arthritis synovial tissue fibroblast cells: correlates with disease activity. Genes Immun, 8(6): 480–491, 2007. PMID: 17568789.

[378] W. Zhang, K. Murao, X. Zhang, K. Matsumoto, S. Diah, M. Okada, K. Miyake, N. Kawai, Z. Fei, and T. Tamiya. Resveratrol represses ykl-40 expression in human glioma u87 cells. BMC Cancer, 10:593, 2010. PMCID: PMC2988030, PMID: 21029458.

[379] BM. Ku, YK. Lee, J. Ryu, JY. Jeong, J. Choi, KM. Eun, HY. Shin, DG. Kim, EM. Hwang, JC. Yoo, JY. Park, GS. Roh, HJ. Kim, GJ. Cho, WS. Choi, SH. Paek, and SS. Kang. Chi3l1 (ykl-40) is expressed in human gliomas and regulates the invasion, growth and survival of glioma cells. Int J Cancer, 128(6):1316–1326, 2011. PMID: 20506295.

[380] C. Ruiz-Romero, V. Calamia, J. Mateos, V. Carreira, M. Martínez-Gomariz, M. Fernández, and FJ. Blanco. Mitochondrial dysregulation of osteoarthritic human articular chondrocytes analyzed by proteomics: a decrease in mitochondrial superoxide dismutase points to a redox imbalance. Mol Cell Proteomics, 8(1):172–189, 2009. PMCID: PMC2713027, PMID: 18784066.

[381] RR. Reams, D. Agrawal, MB. Davis, S. Yoder, FT. Odedina, N. Kumar, JM. Higginbotham, T. Akinremi, S. Suther, and KF. Soliman. Microarray comparison of prostate tumor gene expression in african-american and caucasian american males: a pilot project study.

Infect Agent Cancer, (Suppl 1):S3, 2009. PMCID: PMC2638462, PMID: 19208208.

[382] H. Tummala, AD. Dokal, A. Walne, A. Ellison, S. Cardoso, S. Amirthasigamanipillai, M. Kirwan, I. Browne, JK. Sidhu, V. Rajeeve, A. Rio-Machin, AA. Seraihi, AS. Duncombe, M. Jenner, OP. Smith, H. Enright, A. Norton, T. Aksu, NY. Özbek, N. Pontikos, P. Cutillas, I. Dokal, and T. Vulliamy. Genome instability is a consequence of transcription deficiency in patients with bone marrow failure harboring biallelic ercc6l2 variants. Proc Natl Acad Sci U S A, 115(30):7777–7782, 2018. PMCID: PMC6064997, PMID: 29987015.

[383] E. Dardiotis, E. Karampinis, V. Siokas, AM. Aloizou, D. Rikos, S. Ralli, D. Papadimitriou, DP. Bogdanos, and GM. Hadjigeorgiou. Ercc6l2 rs591486 polymorphism and risk for amyotrophic lateral sclerosis in greek population. Neurol Sci, 40(6):1237–1244, 2019. PMID: 30879219.

[384] CC. Sun, Q. Zhou, W. Hu, SJ. Li, F. Zhang, ZL. Chen, G. Li, ZY. Bi, YY. Bi, FY. Gong, T. Bo, ZP. Yuan, WD. Hu, BT. Zhan, Q. Zhang, QZ. Tang, and DJ. Li. Transcriptional e2f1/2/5/8 as potential targets and transcriptional e2f3/6/7 as new biomarkers for the prognosis of human lung carcinoma. Aging (Albany NY), 10(5):973–987, 2018. PMCID: PMC5990399, PMID: 29754146.

[385] W. Cai, DS. Casey, and JC. Dekkers. Selection response and genetic parameters for residual feed intake in yorkshire swine. J Anim Sci, 86(2):287–298, 2008. PMID: 17998435.

[386] AG. Ozuna, RR. Rowland, JC. Nietfeld, MA. Kerrigan, JC. Dekkers, and CR. Wyatt. Preliminary findings of a previously unrecognized porcine primary immunodeficiency disorder. Vet Pathol, 50(1):144–146, 2013. PMID: 22903400.

[387] MT. Basel, S. Balivada, AP. Beck, MA. Kerrigan, MM. Pyle, JC. Dekkers, CR. Wyatt, RR. Rowland, DE. Anderson, SH. Bossmann, and DL. Troyer. Human xenografts are not rejected in a naturally occurring immunodeficient porcine line: a human tumor model in pigs. Biores Open Access, 1(2):63–68, 2012. PMCID: PMC3559234, PMID: 23514746.

[388] CL. Ewen, AG. Cino-Ozuna, H. He, MA. Kerrigan, JC. Dekkers, CK. Tuggle, RR. Rowland, and CR. Wyatt. Analysis of blood leukocytes in a naturally occurring immunodeficiency of pigs shows the defect is localized to b and t cells. Vet Immunol Immunopathol, 162(3-4):174–179, 2014. PMID: 25454085.

[389] EH. Waide, JC. Dekkers, JW. Ross, RR. Rowland, CR. Wyatt, CL. Ewen, AB. Evans, DM. Thekkoot, NJ. Boddicker, NV. Serão, NM. Ellinwood, and CK. Tuggle. Not all scid pigs are created equally: Two independent mutations in the artemis gene cause scid in pigs. J Immunol, 195(7):3171–3179, 2015. PMCID: PMC5621739, PMID: 26320255.

[390] F. Connell, K. Kalidas, P. Ostergaard, G. Brice, T. Homfray, L. Roberts, DJ. Bunyan, S. Mitton, S. Mansour, P. Mortimer, and S. Jeffery. Linkage and sequence analysis indicate that ccbe1 is mutated in recessively inherited generalised lymphatic dysplasia. Hum Genet, 127(2):231–241, 2010. PMID: 19911200.

[391] M. Alders, BM. Hogan, E. Gjini, F. Salehi, L. Al-Gazali, EA. Hennekam, EE. Holmberg, MM. Mannens, MF. Mulder, GJ. Offerhaus, TE. Prescott, EJ. Schroor, JB. Verheij, M.

Witte, PJ. Zwijnenburg, M. Vikkula, S. Schulte-Merker, and RC. Hennekam. Mutations in ccbe1 cause generalized lymph vessel dysplasia in humans. Nat Genet, 41(12):1272–1274, 2009. PMID: 19935664.

[392] S. Yadav, S. Mukhopadhyay, M. Anbalagan, and N. Makridakis. Somatic mutations in catalytic core of polk reported in prostate cancer alter translesion dna synthesis. Hum Mutat, 36(9):873–880, 2015. PMCID: PMC4537374, PMID: 26046662.

[393] AC. Bostian, L. Maddukuri, MR. Reed, T. Savenka, JH. Hartman, L. Davis, DL. Pouncey, GP. Miller, and RL. Eoff. Kynurenine signaling increases dna polymerase kappa expression and promotes genomic instability in glioblastoma cells. Chem Res Toxicol, 29(1):101–108, 2016. PMCID: PMC4718841, PMID: 26651356.

[394] M. Cai, S. Dai, W. Chen, C. Xia, L. Lu, S. Dai, J. Qi, M. Wang, M. Wang, L. Zhou, F. Lei, T. Zuo, H. Zeng, and X. Zhao. Environmental factors, seven gwas-identified susceptibility loci, and risk of gastric cancer and its precursors in a chinese population. Cancer Med, 6(3):708–720, 2017. PMCID: PMC5345626, PMID: 28220687.

[395] Y. Kinoshita, K. Takasu, T. Yuri, K. Yoshizawa, N. Uehara, A. Kimura, H. Miki, A. Tsubura, and N. Shikata. Two cases of malignant peritoneal mesothelioma without asbestos exposure: cytologic and immunohistochemical features. Ann Diagn Pathol, 17(1):99–9103, 2013. PMID: 22784439.

[396] H. Helgason, T. Rafnar, HS. Olafsdottir, JG. Jonasson, A. Sigurdsson, SN. Stacey, A. Jonasdottir, L. Tryggvadottir, K. Alexiusdottir, A. Haraldsson, L. le Roux, J. Gudmundsson, H. Johannsdottir, A. Oddsson, A. Gylfason, OT. Magnusson, G. Masson, T. Jonsson, H. Skuladottir, DF. Gudbjartsson, U. Thorsteinsdottir, P. Sulem, and K. Stefansson. Loss-of-function variants in atm confer risk of gastric cancer. Nat Genet, 47(8): 906–910, 2015. PMID: 26098866.

[397] A. Kirby, A. Gnirke, DB. Jaffe, V. Barešová, N. Pochet, B. Blumenstiel, C. Ye, D. Aird, C. Stevens, JT. Robinson, MN. Cabili, I. Gat-Viks, E. Kelliher, R. Daza, M. DeFelice, H. Hůlková, J. Sovová, P. Vylet'al, C. Antignac, M. Guttman, RE. Handsaker, D. Perrin, S. Steelman, S. Sigurdsson, SJ. Scheinman, C. Sougnez, K. Cibulskis, M. Parkin, T. Green, E. Rossin, MC. Zody, RJ. Xavier, MR. Pollak, SL. Alper, K. Lindblad-Toh, S. Gabriel, PS. Hart, A. Regev, C. Nusbaum, S. Kmoch, AJ. Bleyer, ES. Lander, and MJ. Daly. Mutations causing medullary cystic kidney disease type 1 lie in a large vntr in muc1 missed by massively parallel sequencing. Nat Genet, 45 (3):299–303, 2013. PMCID: PMC3901305, PMID: 23396133.

[398] J. Creaney, A. Segal, G. Sterrett, MA. Platten, E. Baker, AR. Murch, AK. Nowak, BW. Robinson, and MJ. Millward. Overexpression and altered glycosylation of muc1 in malignant mesothelioma. Br J Cancer, 98(9): 1562–1569, 2008. PMCID: PMC2391110, PMID: 18454162.

[399] X. Xu, L. Bai, W. Chen, MT. Padilla, Y. Liu, KC. Kim, SA. Belinsky, and Y. Lin. Muc1 contributes to bpde-induced human bronchial epithelial cell transformation through facilitating egfr activation. PLoS One, 7(3):e33846, 2012. PMCID: PMC3310874, PMID: 22457794.

[400] SP. Treon, JA. Mollick, M. Urashima, G. Teoh, D. Chauhan, A. Ogata, N. Raje, JH. Hilgers, L. Nadler, AR. Belch, LM. Pilarski, and KC. Anderson. Muc-1 core protein is expressed on multiple myeloma cells and is induced by dexamethasone. Blood, 93(4):1287–1298, 1999. PMID: 9949172.

[401] CH. Takimoto, ZH. Lu, R. Zhang, MD. Liang, LV. Larson, LR. Cantilena, JL. Grem, CJ. Allegra, RB. Diasio, and E. Chu. Severe neurotoxicity following 5-fluorouracil-based chemotherapy in a patient with dihydropyrimidine dehydrogenase deficiency. Clin Cancer Res, 2(3):477–481, 1996. PMID: 9816193.

[402] CW. Woods, MT. McClain, M. Chen, AK. Zaas, BP. Nicholson, J. Varkey, T. Veldman, SF. Kingsmore, Y. Huang, R. Lambkin-Williams, AG. Gilbert, AO. Hero, E. Ramsburg, S. Glickman, JE. Lucas, L. Carin, and GS. Ginsburg. A host transcriptional signature for presymptomatic detection of infection in humans exposed to influenza h1n1 or h3n2. PLoS One, 8(1):e52198, 2013. PMCID: PMC3541408, PMID: 23326326.

[403] KA. Aldinger, AE. Timms, Z. Thomson, GM. Mirzaa, JT. Bennett, AB. Rosenberg, CM. Roco, M. Hirano, F. Abidi, P. Haldipur, CV. Cheng, S. Collins, K. Park, J. Zeiger, LM. Overmann, FS. Alkuraya, LG. Biesecker, SR. Braddock, S. Cathey, MT. Cho, BHY. Chung, DB. Everman, YA. Zarate, JR. Jones, CE. Schwartz, A. Goldstein, RJ. Hopkin, ID. Krantz, RL. Ladda, KA. Leppig, BC. McGillivray, S. Sell, K. Wusik, JG. Gleeson, DA. Nickerson, MJ. Bamshad, D. Gerrelli, SN. Lisgo, G. Seelig, GE. Ishak, AJ. Barkovich, CJ. Curry, IA. Glass, KJ. Millen, D. Doherty, and WB. Dobyns. Redefining the etiologic landscape of cerebellar malformations. Am J Hum Genet, 105(3):606–615, 2019. PMCID: PMC6731369, PMID: 31474318.

[404] C. Nava, F. Lamari, D. Héron, C. Mignot, A. Rastetter, B. Keren, D. Cohen, A. Faudet, D. Bouteiller, M. Gilleron, A. Jacquette, S. Whalen, A. Afenjar, D. Périsse, C. Laurent, C. Dupuits, C. Gautier, M. Gérard, G. Huguet, S. Caillet, B. Leheup, M. Leboyer, C. Gillberg, R. Delorme, T. Bourgeron, A. Brice, and C. Depienne. Analysis of the chromosome x exome in patients with autism spectrum disorders identified novel candidate genes, including tmlhe. Transl Psychiatry, 2(10):e179, 2012. PMCID: PMC3565810, PMID: 23092983.

[405] YJ. Yang, L. Hu, YP. Xia, CY. Jiang, C. Miao, CQ. Yang, M. Yuan, and L. Wang. Resveratrol suppresses glial activation and alleviates trigeminal neuralgia via activation of ampk. J Neuroinflammation, 13(1):84, 2016. PMCID: PMC4837542, PMID: 27093858.

[406] M. Tong, M. Dong, and SM. de la Monte. Brain insulin-like growth factor and neurotrophin resistance in parkinson’s disease and dementia with lewy bodies: potential role of manganese neurotoxicity. J Alzheimers Dis, 16(3):585–599, 2009. PMCID: PMC2852260, PMID: 19276553.

[407] T. Chen, W. Wang, JR. Li, HZ. Xu, YC. Peng, LF. Fan, F. Yan, C. Gu, L. Wang, and G. Chen. Parp inhibition attenuates early brain injury through nf-κb/mmp-9 pathway in a rat model of subarachnoid hemorrhage. Brain Res, 1644:32–38, 2016. PMID: 27157545.

[408] SS. Wang, Y. Lu, N. Rothman, AM. Abdou, JR. Cerhan, A. De Roos, S. Davis, RK. Severson, W. Cozen, SJ. Chanock, L. Bernstein, LM. Morton, and P. Hartge. Variation

in effects of non-hodgkin lymphoma risk factors according to the human leukocyte antigen (hla)-drb1*01:01 allele and ancestral haplotype 8.1. PLoS One, 6 (11):e26949, 2011. PMCID: PMC3212525, PMID: 22096508.

[409] SH. Kim, YM. Ye, NS. Palikhe, JE. Kim, and HS. Park. Genetic and ethnic risk factors associated with drug hypersensitivity. Curr Opin Allergy Clin Immunol, 10(4):280–290, 2010. PMID: 20485159.

[410] M. Marchini, R. Antonioli, A. Lleò, M. Barili, M. Caronni, L. Origgi, M. Vanoli, and R. Scorza. Hla class ii antigens associated with lupus nephritis in italian sle patients. Hum Immunol, 64(4):462–468, 2003. PMID: 12651073.

[411] W. Wen, W. Su, H. Tang, W. Le, X. Zhang, Y. Zheng, X. Liu, L. Xie, J. Li, J. Ye, L. Dong, X. Cui, Y. Miao, D. Wang, J. Dong, C. Xiao, W. Chen, and H. Wang. Immune cell profiling of covid-19 patients in the recovery stage by single-cell sequencing. Cell Discov, 6:31, 2020. PMCID: PMC7197635, PMID: 32377375.

[412] JR. Bill, DG. Mack, MT. Falta, LA. Maier, AK. Sullivan, FG. Joslin, AK. Martin, BM. Freed, BL. Kotzin, and AP. Fontenot. Beryllium presentation to cd4+ t cells is dependent on a single amino acid residue of the mhc class ii beta-chain. J Immunol, 175(10):7029–7037, 2005. PMID: 16272364.

[413] H. Sato, P. Spagnolo, L. Silveira, KI. Welsh, RM. du Bois, LS. Newman, and LA. Maier. Btnl2 allele associations with chronic beryllium disease in hla-dpb1*glu69-negative individuals. Tissue Antigens, 70(6):480–486, 2007. PMID: 17927685.

[414] I. Gunnarsson, B. Ringertz, J. Bratt, and B. Sundelin. Hla-drb1*0301 and dqa1*0501 in ra. Ann Rheum Dis, 60 (7):727, 2001. PMCID: PMC1753742, PMID: 11436868.

[415] N. Zhu, F. Luo, Q. Chen, N. Li, H. Xiong, Y. Feng, Z. Yang, and W. Hou. Influence of hla-drb alleles on haemorrhagic fever with renal syndrome in a chinese han population in hubei province, china. Eur J Clin Microbiol Infect Dis, 34(1):187–195, 2015. PMID: 25169964.

[416] MD. Rossman, B. Thompson, M. Frederick, M. Maliarik, MC. Iannuzzi, BA. Rybicki, JP. Pandey, LS. Newman, E. Magira, B. Beznik-Cizman, and D. Monos. Hla-drb1*1101: a significant risk factor for sarcoidosis in blacks and whites. Am J Hum Genet, 73(4):720–735, 2003. PMCID: PMC1180597, PMID: 14508706.

[417] F. Speerstra, P. Reekers, LB. van de Putte, JP. Vandenbroucke, JJ. Rasker, and DJ. de Rooij. Hla-dr antigens and proteinuria induced by aurothioglucose and d-penicillamine in patients with rheumatoid arthritis. J Rheumatol, 10(6):948–953, 1983. PMID: 6420562.

[418] AA. Al-Eisa, MZ. Haider, and BS. Srivasta. Hla-drb1 alleles in kuwaiti children with idiopathic nephrotic syndrome. Pediatr Nephrol, 15(1-2):79–81, 2000. PMID: 11095018.

[419] K. Saito. [analysis of a genetic factor of metal allergy–polymorphism of hla-dr, -dq gene]. Kokubyo Gakkai Zasshi, 63(1):53–69, 1996. PMID: 8725357.

[420] RP. Warren, JD. Odell, WL. Warren, RA. Burger, A. Maciulis, WW. Daniels, and AR. Torres. Strong association of the third hypervariable region of hla-dr beta 1 with autism.

J Neuroimmunol, 67(2):97–9102, 1996. PMID: 8765331.

[421] SJ. Dunstan, NT. Hue, B. Han, Z. Li, TT. Tram, KS. Sim, CM. Parry, NT. Chinh, H. Vinh, NP. Lan, NT. Thieu, PV. Vinh, S. Koirala, S. Dongol, A. Arjyal, A. Karkey, O. Shilpakar, C. Dolecek, JN. Foo, le T. Phuong, MN. Lanh, T. Do, T. Aung, DN. Hon, YY. Teo, ML. Hibberd, KL. Anders, Y. Okada, S. Raychaudhuri, CP. Simmons, S. Baker, PI. de Bakker, B. Basnyat, TT. Hien, JJ. Farrar, and CC. Khor. Variation at hla-drb1 is associated with resistance to enteric fever. Nat Genet, 46(12):1333–1336, 2014. PMCID: PMC5099079, PMID: 25383971.

[422] M. Grosser, T. Luther, M. Fuessel, J. Bickhardt, V. Magdolen, and G. Baretton. Clinical course of sarcoidosis in dependence on hla-drb1 allele frequencies, inflammatory markers, and the presence of m. tuberculosis dna fragments. Sarcoidosis Vasc Diffuse Lung Dis, 22(1):66–74, 2005. PMID: 15881283.

[423] SH. Kim, JH. Choi, KW. Lee, SH. Kim, ES. Shin, HB. Oh, CH. Suh, DH. Nahm, and HS. Park. The human leucocyte antigen-drb1*1302-dqb1*0609-dpb1*0201 haplotype may be a strong genetic marker for aspirin-induced urticaria. Clin Exp Allergy, 35(3):339–344, 2005. PMID: 15784113.

[424] LOF. Cangussu, R. Teixeira, EF. Campos, GF. Rampim, SA. Mingoti, OA. Martins-Filho, and M. Gerbase-DeLima. Hla class ii alleles and chronic hepatitis c virus infection. Scand J Immunol, 74(3): 282–287, 2011. PMID: 21535077.

[425] N. Kaushansky, M. Eisenstein, S. Boura-Halfon, BE. Hansen, CH. Nielsen, R. Milo, G. Zeilig, H. Lassmann, DM. Altmann, and A. Ben-Nun. Role of a novel human leukocyte antigen-dqa1*01:02;drb1*15:01 mixed isotype heterodimer in the pathogenesis of "humanized" multiple sclerosis-like disease. J Biol Chem, 290(24): 15260–15278, 2015. PMCID: PMC4463466, PMID: 25911099.

[426] SH. Kim, HB. Oh, KW. Lee, ES. Shin, CW. Kim, CS. Hong, DH. Nahm, and HS. Park. Hla drb1*15-dpb1*05 haplotype: a susceptible gene marker for isocyanate-induced occupational asthma? Allergy, 61(7):891–894, 2006. PMID: 16792590.

[427] N. Keicho, S. Itoyama, K. Kashiwase, NC. Phi, HT. Long, LD. Ha, VV. Ban, BK. Hoa, NT. Hang, M. Hijikata, S. Sakurada, M. Satake, K. Tokunaga, T. Sasazuki, and T. Quy. Association of human leukocyte antigen class ii alleles with severe acute respiratory syndrome in the vietnamese population. Hum Immunol, 70(7):527–531, 2009. PMCID: PMC7132661, PMID: 19445991.

[428] GA. Heap, MN. Weedon, CM. Bewshea, A. Singh, M. Chen, JB. Satchwell, JP. Vivian, K. So, PC. Dubois, JM. Andrews, V. Annese, P. Bampton, M. Barnardo, S. Bell, A. Cole, SJ. Connor, T. Creed, FR. Cummings, M. D'Amato, TK. Daneshmend, RN. Fedorak, TH. Florin, DR. Gaya, E. Greig, J. Halfvarson, A. Hart, PM. Irving, G. Jones, A. Karban, IC. Lawrance, JC. Lee, C. Lees, R. Lev-Tzion, JO. Lindsay, J. Mansfield, J. Mawdsley, Z. Mazhar, M. Parkes, K. Parnell, TR. Orchard, G. Radford-Smith, RK. Russell, D. Reffitt, J. Satsangi, MS. Silverberg, GC. Sturniolo, M. Tremelling, EV. Tsianos, DA. van Heel, A. Walsh, G. Watermeyer, RK. Weersma, S. Zeissig, J. Rossjohn, AL. Holden, and T. Ahmad. Hla-dqa1-hla-drb1 variants confer susceptibility to pancreatitis induced by thiopurine immunosuppressants. Nat Genet, 46(10):1131–1134, 2014. PMCID:

PMC6379053, PMID: 25217962.

[429] SJ. Glatt, IP. Everall, WS. Kremen, J. Corbeil, R. Sásik, N. Khanlou, M. Han, CC. Liew, and MT. Tsuang. Comparative gene expression analysis of blood and brain provides concurrent validation of selenbp1 up-regulation in schizophrenia. Proc Natl Acad Sci U S A, 102(43):15533–15538, 2005. PMCID: PMC1266138, PMID: 16223876.

[430] H. Mahdi, BA. Fisher, H. Källberg, D. Plant, V. Malmström, J. Rönnelid, P. Charles, B. Ding, L. Alfredsson, L. Padyukov, DP. Symmons, PJ. Venables, L. Klareskog, and K. Lundberg. Specific interaction between genotype, smoking and autoimmunity to citrullinated alpha-enolase in the etiology of rheumatoid arthritis. Nat Genet, 41(12):1319–1324, 2009. PMID: 19898480.

[431] EA. Stahl, S. Raychaudhuri, EF. Remmers, G. Xie, S. Eyre, BP. Thomson, Y. Li, FA. Kurreeman, A. Zhernakova, A. Hinks, C. Guiducci, R. Chen, L. Alfredsson, CI. Amos, KG. Ardlie, A. Barton, J. Bowes, E. Brouwer, NP. Burtt, JJ. Catanese, J. Coblyn, MJ. Coenen, KH. Costenbader, LA. Criswell, JB. Crusius, J. Cui, PI. de Bakker, PL. De Jager, B. Ding, P. Emery, E. Flynn, P. Harrison, LJ. Hocking, TW. Huizinga, DL. Kastner, X. Ke, AT. Lee, X. Liu, P. Martin, AW. Morgan, L. Padyukov, MD. Posthumus, TR. Radstake, DM. Reid, M. Seielstad, MF. Seldin, NA. Shadick, S. Steer, PP. Tak, W. Thomson, AH. van der Helm-van Mil, IE. van der Horst-Bruinsma, CE. van der Schoot, PL. van Riel, ME. Weinblatt, AG. Wilson, GJ. Wolbink, BP. Wordsworth, C. Wijmenga, EW. Karlson, RE. Toes, N. de Vries, AB. Begovich, J. Worthington, KA. Siminovitch, PK. Gregersen, L. Klareskog, and RM. Plenge. Genome-wide association study meta-analysis identifies seven new rheumatoid arthritis risk loci. Nat Genet, 42(6):508–514, 2010. PMCID: PMC4243840, PMID: 20453842.

[432] S. Raychaudhuri, C. Sandor, EA. Stahl, J. Freudenberg, HS. Lee, X. Jia, L. Alfredsson, L. Padyukov, L. Klareskog, J. Worthington, KA. Siminovitch, SC. Bae, RM. Plenge, PK. Gregersen, and PI. de Bakker. Five amino acids in three hla proteins explain most of the association between mhc and seropositive rheumatoid arthritis. Nat Genet, 44(3):291–296, 2012. PMCID: PMC3288335, PMID: 22286218.

[433] SV. Ramagopalan, UC. Meier, M. Conacher, GC. Ebers, G. Giovannoni, DH. Crawford, and KA. McAulay. Role of the hla system in the association between multiple sclerosis and infectious mononucleosis. Arch Neurol, 68 (4):469–472, 2011. PMID: 21482926.

[434] P. Goyette, G. Boucher, D. Mallon, E. Ellinghaus, L. Jostins, H. Huang, S. Ripke, ES. Gusareva, V. Annese, SL. Hauser, JR. Oksenberg, I. Thomsen, S. Leslie, MJ. Daly, K. Van Steen, RH. Duerr, JC. Barrett, DP. McGovern, LP. Schumm, JA. Traherne, MN. Carrington, V. Kosmoliaptsis, TH. Karlsen, A. Franke, and JD. Rioux. High-density mapping of the mhc identifies a shared role for hla-drb1*01:03 in inflammatory bowel diseases and heterozygous advantage in ulcerative colitis. Nat Genet, 47(2):172–179, 2015. PMCID: PMC4310771, PMID: 25559196.

[435] H. Hor, Z. Kutalik, Y. Dauvilliers, A. Valsesia, GJ. Lammers, CE. Donjacour, A. Iranzo, J. Santamaria, R. Peraita Adrados, JL. Vicario, S. Overeem, I. Arnulf, I. Theodorou, P. Jennum, S. Knudsen, C. Bassetti, J. Mathis, M. Lecendreux, G. Mayer, P. Geisler, A. Benetó, B. Petit, C. Pfister, JV. Bürki, G. Didelot, M. Billiard, G. Ercilla, W. Verduijn, FH. Claas, P. Vollenweider, G. Waeber, DM. Waterworth, V. Mooser, R. Heinzer, JS.

Beckmann, S. Bergmann, and M. Tafti. Genome-wide association study identifies new hla class ii haplotypes strongly protective against narcolepsy. Nat Genet, 42(9):786–789, 2010. PMID: 20711174.

[436] C. Lesseur, B. Diergaarde, AF. Olshan, V. Wünsch-Filho, AR. Ness, G. Liu, M. Lacko, J. Eluf-Neto, S. Franceschi, P. Lagiou, GJ. Macfarlane, L. Richiardi, S. Boccia, J. Polesel, K. Kjaerheim, D. Zaridze, M. Johansson, AM. Menezes, MP. Curado, M. Robinson, W. Ahrens, C. Canova, A. Znaor, X. Castellsagué, DI. Conway, I. Holcátová, D. Mates, M. Vilensky, CM. Healy, N. Szeszenia-Dabrowska, E. Fabiánová, J. Lissowska, JR. Grandis, MC. Weissler, EH. Tajara, FD. Nunes, MB. de Carvalho, S. Thomas, RJ. Hung, WH. Peters, R. Herrero, G. Cadoni, HB. Bueno-de Mesquita, A. Steffen, A. Agudo, O. Shangina, X. Xiao, V. Gaborieau, A. Chabrier, D. Anantharaman, P. Boffetta, CI. Amos, JD. McKay, and P. Brennan. Genome-wide association analyses identify new susceptibility loci for oral cavity and pharyngeal cancer. Nat Genet, 48(12):1544–1550, 2016. PMCID: PMC5131845, PMID: 27749845.

[437] MI. Lucena, M. Molokhia, Y. Shen, TJ. Urban, GP. Aithal, RJ. Andrade, CP. Day, F. Ruiz-Cabello, PT. Donaldson, C. Stephens, M. Pirmohamed, M. Romero-Gomez, JM. Navarro, RJ. Fontana, M. Miller, M. Groome, E. Bondon-Guitton, A. Conforti, BH. Stricker, A. Carvajal, L. Ibanez, QY. Yue, M. Eichelbaum, A. Floratos, I. Pe'er, MJ. Daly, DB. Goldstein, JF. Dillon, MR. Nelson, PB. Watkins, and AK. Daly. Susceptibility to amoxicillin-clavulanate-induced liver injury is influenced by multiple hla class i and ii alleles. Gastroenterology, 141(1):338–347, 2011. PMCID: PMC3129430, PMID: 21570397.

[438] M. Fakiola, A. Strange, HJ. Cordell, EN. Miller, M. Pirinen, Z. Su, A. Mishra, S. Mehrotra, GR. Monteiro, G. Band, C. Bellenguez, S. Dronov, S. Edkins, C. Freeman, E. Giannoulatou, E. Gray, SE. Hunt, HG. Lacerda, C. Langford, R. Pearson, NN. Pontes, M. Rai, SP. Singh, L. Smith, O. Sousa, D. Vukcevic, E. Bramon, MA. Brown, JP. Casas, A. Corvin, A. Duncanson, J. Jankowski, HS. Markus, CG. Mathew, CN. Palmer, R. Plomin, A. Rautanen, SJ. Sawcer, RC. Trembath, AC. Viswanathan, NW. Wood, ME. Wilson, P. Deloukas, L. Peltonen, F. Christiansen, C. Witt, SM. Jeronimo, S. Sundar, CC. Spencer, JM. Blackwell, and P. Donnelly. Common variants in the hla-drb1-hla-dqa1 hla class ii region are associated with susceptibility to visceral leishmaniasis. Nat Genet, 45(2):208–213, 2013. PMCID: PMC3664012, PMID: 23291585.

[439] X. Hu, AJ. Deutsch, TL. Lenz, S. Onengut-Gumuscu, B. Han, WM. Chen, JM. Howson, JA. Todd, PI. de Bakker, SS. Rich, and S. Raychaudhuri. Additive and interaction effects at three amino acid positions in hla-dq and hla-dr molecules drive type 1 diabetes risk. Nat Genet, 47(8):898–905, 2015. PMCID: PMC4930791, PMID: 26168013.

[440] S. Okada, ML. Kamb, JP. Pandey, RM. Philen, LA. Love, and FW. Miller. Immunogenetic risk and protective factors for the development of l-tryptophan-associated eosinophilia-myalgia syndrome and associated symptoms. Arthritis Rheum, 61(10):1305–1311, 2009. PMCID: PMC2761987, PMID: 19790128.

[441] SH. Kim and HS. Park. Genetic markers for differentiating aspirin-hypersensitivity. Yonsei Med J, 47(1):15–21, 2006. PMCID: PMC2687575, PMID: 16502481.

[442] CE. Mapp, B. Beghè, A. Balboni, G. Zamorani, M. Padoan, L. Jovine, OR. Baricordi, and

LM. Fabbri. Association between hla genes and susceptibility to toluene diisocyanate-induced asthma. Clin Exp Allergy, 30 (5):651–656, 2000. PMID: 10792356.

[443] K. Kiryluk, Y. Li, F. Scolari, S. Sanna-Cherchi, M. Choi, M. Verbitsky, D. Fasel, S. Lata, S. Prakash, S. Shapiro, C. Fischman, HJ. Snyder, G. Appel, C. Izzi, BF. Viola, N. Dallera, L. Del Vecchio, C. Barlassina, E. Salvi, FE. Bertinetto, A. Amoroso, S. Savoldi, M. Rocchietti, A. Amore, L. Peruzzi, R. Coppo, M. Salvadori, P. Ravani, R. Magistroni, GM. Ghiggeri, G. Caridi, M. Bodria, F. Lugani, L. Allegri, M. Delsante, M. Maiorana, A. Magnano, G. Frasca, E. Boer, G. Boscutti, C. Ponticelli, R. Mignani, C. Marcantoni, D. Di Landro, D. Santoro, A. Pani, R. Polci, S. Feriozzi, S. Chicca, M. Galliani, M. Gigante, L. Gesualdo, P. Zamboli, GG. Battaglia, M. Garozzo, D. Maixnerová, V. Tesar, F. Eitner, T. Rauen, J. Floege, T. Kovacs, J. Nagy, K. Mucha, L. Paczek, ˛ M. Zaniew, M. Mizerska-Wasiak, M. Roszkowska-Blaim, K. Pawlaczyk, D. Gale, J. Barratt, L. Thibaudin, F. Berthoux, G. Canaud, A. Boland, M. Metzger, U. Panzer, H. Suzuki, S. Goto, I. Narita, Y. Caliskan, J. Xie, P. Hou, N. Chen, H. Zhang, RJ. Wyatt, J. Novak, BA. Julian, J. Feehally, B. Stengel, D. Cusi, RP. Lifton, and AG. Gharavi. Discovery of new risk loci for iga nephropathy implicates genes involved in immunity against intestinal pathogens. Nat Genet, 46(11):1187–1196, 2014. PMCID: PMC4213311, PMID: 25305756.

[444] TR. Radstake, O. Gorlova, B. Rueda, JE. Martin, BZ. Alizadeh, R. Palomino-Morales, MJ. Coenen, MC. Vonk, AE. Voskuyl, AJ. Schuerwegh, JC. Broen, PL. van Riel, R. van 't Slot, A. Italiaander, RA. Ophoff, G. Riemekasten, N. Hunzelmann, CP. Simeon, N. Ortego-Centeno, MA. González-Gay, MF. González-Escribano, P. Airo, J. van Laar, A. Herrick, J. Worthington, R. Hesselstrand, V. Smith, F. de Keyser, F. Houssiau, MM. Chee, R. Madhok, P. Shiels, R. Westhovens, A. Kreuter, H. Kiener, E. de Baere, T. Witte, L. Padykov, L. Klareskog, L. Beretta, R. Scorza, BA. Lie, AM. Hoffmann-Vold, P. Carreira, J. Varga, M. Hinchcliff, PK. Gregersen, AT. Lee, J. Ying, Y. Han, SF. Weng, CI. Amos, FM. Wigley, L. Hummers, JL. Nelson, SK. Agarwal, S. Assassi, P. Gourh, FK. Tan, BP. Koeleman, FC. Arnett, J. Martin, and MD. Mayes. Genome-wide association study of systemic sclerosis identifies cd247 as a new susceptibility locus. Nat Genet, 42(5): 426–429, 2010. PMCID: PMC2861917, PMID: 20383147.

[445] I. Gockel, J. Becker, MM. Wouters, S. Niebisch, HR. Gockel, T. Hess, D. Ramonet, J. Zimmermann, AG. Vigo, G. Trynka, AR. de León, JP. de la Serna, E. Urcelay, V. Kumar, L. Franke, HJ. Westra, D. Drescher, W. Kneist, JU. Marquardt, PR. Galle, M. Mattheisen, V. Annese, A. Latiano, U. Fumagalli, L. Laghi, R. Cuomo, G. Sarnelli, M. Müller, AJ. Eckardt, J. Tack, P. Hoffmann, S. Herms, E. Mangold, S. Heilmann, R. Kiesslich, BH. von Rahden, HD. Allescher, HG. Schulz, C. Wijmenga, MT. Heneka, H. Lang, KP. Hopfner, MM. Nöthen, GE. Boeckxstaens, PI. de Bakker, M. Knapp, and J. Schumacher. Common variants in the hla-dq region confer susceptibility to idiopathic achalasia. Nat Genet, 46(8):901–904, 2014. PMID: 24997987.

[446] M. Hussong, ST. Börno, M. Kerick, A. Wunderlich, A. Franz, H. Sültmann, B. Timmermann, H. Lehrach, M. Hirsch-Kauffmann, and MR. Schweiger. The bromodomain protein brd4 regulates the keap1/nrf2-dependent oxidative stress response. Cell Death Dis, 5(4):e1195, 2014. PMCID: PMC4001311, PMID: 24763052.

[447] F. Marbach, CF. Rustad, A. Riess, D. Ðukić, TC. Hsieh, I. Jobani, T. Prescott, A. Bevot, F. Erger, G. Houge, M. Redfors, J. Altmueller, T. Stokowy, C. Gilissen, C. Kubisch, E. Scarano, L. Mazzanti, T. Fiskerstrand, PM. Krawitz, D. Lessel, and C. Netzer. The

discovery of a lemd2-associated nuclear envelopathy with early progeroid appearance suggests advanced applications for ai-driven facial phenotyping. Am J Hum Genet, 104 (4):749–757, 2019. PMCID: PMC6451726, PMID: 30905398.

[448] M. Christensen, M. Duno, AM. Lund, F. Skovby, and E. Christensen. Xanthurenic aciduria due to a mutation in kynu encoding kynureninase. J Inherit Metab Dis, 30(2):248–255, 2007. PMID: 17334708.

[449] DG. Walker, J. Link, LF. Lue, JE. Dalsing-Hernandez, and BE. Boyes. Gene expression changes by amyloid beta peptide-stimulated human postmortem brain microglia identify activation of multiple inflammatory processes. J Leukoc Biol, 79(3):596–610, 2006. PMID: 16365156.

[450] N. Ehmke, K. Cusmano-Ozog, R. Koenig, M. Holtgrewe, B. Nur, E. Mihci, H. Babcock, C. Gonzaga-Jauregui, JD. Overton, J. Xiao, AF. Martinez, M. Muenke, A. Balzer, J. Jochim, N. El Choubassi, B. Fischer-Zirnsak, C. Huber, U. Kornak, SH. Elsea, V. Cormier-Daire, and CR. Ferreira. Biallelic variants in kynu cause a multisystemic syndrome with hand hyperphalangism. Bone, 133:115219, 2020. PMCID: PMC10521254, PMID: 31923704.

[451] RW. Collin, K. Nikopoulos, M. Dona, C. Gilissen, A. Hoischen, FN. Boonstra, JA. Poulter, H. Kondo, W. Berger, C. Toomes, T. Tahira, LR. Mohn, EA. Blokland, L. Hetterschijt, M. Ali, JM. Groothuismink, L. Duijkers, CF. Inglehearn, L. Sollfrank, TM. Strom, E. Uchio, CE. van Nouhuys, H. Kremer, JA. Veltman, E. van Wijk, and FP. Cremers. Znf408 is mutated in familial exudative vitreoretinopathy and is crucial for the development of zebrafish retinal vasculature. Proc Natl Acad Sci U S A, 110(24):9856–9861, 2013. PMCID: PMC3683717, PMID: 23716654.

[452] I. Habibi, A. Chebil, F. Kort, DF. Schorderet, and L. El Matri. Exome sequencing confirms znf408 mutations as a cause of familial retinitis pigmentosa. Ophthalmic Genet, 38(5):494–497, 2017. PMID: 28095122.

[453] A. Avila-Fernandez, R. Perez-Carro, M. Corton, MI. Lopez-Molina, L. Campello, A. Garanto, L. Fernandez-Sanchez, L. Duijkers, MA. Lopez-Martinez, R. Riveiro-Alvarez, LR. Da Silva, R. Sanchez-Alcudia, E. Martin-Garrido, N. Reyes, F. Garcia-Garcia, J. Dopazo, B. Garcia-Sandoval, RW. Collin, N. Cuenca, and C. Ayuso. Whole-exome sequencing reveals znf408 as a new gene associated with autosomal recessive retinitis pigmentosa with vitreal alterations. Hum Mol Genet, 24(14):4037–4048, 2015. PMID: 25882705.

[454] H. Najmabadi, H. Hu, M. Garshasbi, T. Zemojtel, SS. Abedini, W. Chen, M. Hosseini, F. Behjati, S. Haas, P. Jamali, A. Zecha, M. Mohseni, L. Püttmann, LN. Vahid, C. Jensen, LA. Moheb, M. Bienek, F. Larti, I. Mueller, R. Weissmann, H. Darvish, K. Wrogemann, V. Hadavi, B. Lipkowitz, S. Esmaeeli-Nieh, D. Wieczorek, R. Kariminejad, SG. Firouzabadi, M. Cohen, Z. Fattahi, I. Rost, F. Mojahedi, C. Hertzberg, A. Dehghan, A. Rajab, MJ. Banavandi, J. Hoffer, M. Falah, L. Musante, V. Kalscheuer, R. Ullmann, AW. Kuss, A. Tzschach, K. Kahrizi, and HH. Ropers. Deep sequencing reveals 50 novel genes for recessive cognitive disorders. Nature, 478(7367):57–63, 2011. PMID: 21937992.

[455] RS. Thomas, TM. O'Connell, L. Pluta, RD. Wolfinger, L. Yang, and TJ. Page. A

comparison of transcriptomic and metabonomic technologies for identifying biomarkers predictive of two-year rodent cancer bioassays. Toxicol Sci, 96(1):40–46, 2007. PMID: 17114358.

[456] S. Nicole, A. Chaouch, T. Torbergsen, S. Bauché, E. de Bruyckere, MJ. Fontenille, MA. Horn, M. van Ghelue, S. Løseth, Y. Issop, D. Cox, JS. Müller, T. Evangelista, E. Stålberg, C. Ioos, A. Barois, G. Brochier, D. Sternberg, E. Fournier, D. Hantaï, A. Abicht, M. Dusl, SH. Laval, H. Griffin, B. Eymard, and H. Lochmüller. Agrin mutations lead to a congenital myasthenic syndrome with distal muscle weakness and atrophy. Brain, 137(Pt 9): 2429–2443, 2014. PMID: 24951643.

[457] JC. Zenteno, B. Buentello-Volante, MA. Quiroz-González, and MA. Quiroz-Reyes. Compound heterozygosity for a novel and a recurrent mfrp gene mutation in a family with the nanophthalmos-retinitis pigmentosa complex. Mol Vis, 15:1794–1798, 2009. PMCID: PMC2742641, PMID: 19753314.

[458] MB. Consugar, D. Navarro-Gomez, EM. Place, KM. Bujakowska, ME. Sousa, ZD. Fonseca-Kelly, DG. Taub, M. Janessian, DY. Wang, ED. Au, KB. Sims, DA. Sweetser, AB. Fulton, Q. Liu, JL. Wiggs, X. Gai, and EA. Pierce. Panel-based genetic diagnostic testing for inherited eye diseases is highly accurate and reproducible, and more sensitive for variant detection, than exome sequencing. Genet Med, 17(4):253–261, 2015. PMCID: PMC4572572, PMID: 25412400.

[459] Y. Xu, L. Guan, X. Xiao, J. Zhang, S. Li, H. Jiang, X. Jia, Y. Yin, X. Guo, Z. Yang, and Q. Zhang. Identification of mfrp mutations in chinese families with high hyperopia. Optom Vis Sci, 93(1):19–26, 2016. PMID: 26583794.

[460] G. Velez, SH. Tsang, YT. Tsai, CW. Hsu, A. Gore, AH. Abdelhakim, M. Mahajan, RH. Silverman, JR. Sparrow, AG. Bassuk, and VB. Mahajan. Gene therapy restores mfrp and corrects axial eye length. Sci Rep, 7(1):16151, 2017. PMCID: PMC5701072, PMID: 29170418.

[461] D. Matías-Pérez, LA. García-Montaño, M. Cruz-Aguilar, IA. García-Montalvo, J. Nava-Valdéz, T. Barragán-Arevalo, C. Villanueva-Mendoza, CE. Villarroel, C. Guadarrama-Vallejo, RV. la Cruz, O. Chacón-Camacho, and JC. Zenteno. Identification of novel pathogenic variants and novel gene-phenotype correlations in mexican subjects with microphthalmia and/or anophthalmia by next-generation sequencing. J Hum Genet, 63(11):1169–1180, 2018. PMID: 30181649.

[462] R. Ayyagari, MN. Mandal, AJ. Karoukis, L. Chen, NC. McLaren, M. Lichter, DT. Wong, PF. Hitchcock, RC. Caruso, SE. Moroi, IH. Maumenee, and PA. Sieving. Late-onset macular degeneration and long anterior lens zonules result from a ctrp5 gene mutation. Invest Ophthalmol Vis Sci, 46(9):3363–3371, 2005. PMID: 16123441.

[463] OH. Sundin, GS. Leppert, ED. Silva, JM. Yang, S. Dharmaraj, IH. Maumenee, LC. Santos, CF. Parsa, EI. Traboulsi, KW. Broman, C. Dibernardo, JS. Sunness, J. Toy, and EM. Weinberg. Extreme hyperopia is the result of null mutations in mfrp, which encodes a frizzled-related protein. Proc Natl Acad Sci U S A, 102(27): 9553–9558, 2005. PMCID: PMC1172243, PMID: 15976030.

[464] T. Yamamoto, MA. Mencarelli, C. Di Marco, M. Mucciolo, M. Vascotto, P. Balestri, M. Gérard, M. Mathieu-Dramard, J. Andrieux, M. Breuning, MJ. Hoffer, CA. Ruivenkamp, S. Shimada, N. Sangu, K. Shimojima, R. Umezu, H. Kawame, M. Matsuo, K. Saito, A. Renieri, and F. Mari. Overlapping microdeletions involving 15q22.2 narrow the critical region for intellectual disability to narg2 and rora. Eur J Med Genet, 57(4): 163–168, 2014. PMID: 24525055.

[465] T. Yamauchi, K. Hara, S. Maeda, K. Yasuda, A. Takahashi, M. Horikoshi, M. Nakamura, H. Fujita, N. Grarup, S. Cauchi, DP. Ng, RC. Ma, T. Tsunoda, M. Kubo, H. Watada, H. Maegawa, M. Okada-Iwabu, M. Iwabu, N. Shojima, HD. Shin, G. Andersen, DR. Witte, T. Jørgensen, T. Lauritzen, A. Sandbæk, T. Hansen, T. Ohshige, S. Omori, I. Saito, K. Kaku, H. Hirose, WY. So, D. Beury, JC. Chan, KS. Park, ES. Tai, C. Ito, Y. Tanaka, A. Kashiwagi, R. Kawamori, M. Kasuga, P. Froguel, O. Pedersen, N. Kamatani, Y. Nakamura, and T. Kadowaki. A genome-wide association study in the japanese population identifies susceptibility loci for type 2 diabetes at ube2e2 and c2cd4a-c2cd4b. Nat Genet, 42(10):864–868, 2010. PMID: 20818381.

[466] RN. Bamford, E. Roessler, RD. Burdine, U. Saplakoğlu, J. dela Cruz, M. Splitt, JA. Goodship, J. Towbin, P. Bowers, GB. Ferrero, B. Marino, AF. Schier, MM. Shen, M. Muenke, and B. Casey. Loss-of-function mutations in the egf-cfc gene cfc1 are associated with human left-right laterality defects. Nat Genet, 26(3):365–369, 2000. PMID: 11062482.

[467] JM. de la Cruz, RN. Bamford, RD. Burdine, E. Roessler, AJ. Barkovich, D. Donnai, AF. Schier, and M. Muenke. A loss-of-function mutation in the cfc domain of tdgf1 is associated with human forebrain defects. Hum Genet, 110(5):422–428, 2002. PMID: 12073012.

[468] F. Cottrez, E. Boitel, C. Auriault, P. Aeby, and H. Groux. Genes specifically modulated in sensitized skins allow the detection of sensitizers in a reconstructed human skin model. development of the sens-is assay. Toxicol In Vitro, 29(4):787–802, 2015. PMID: 25724174.
