# Supplementary material for: A revamped rat reference genome improves the discovery of genetic diversity in laboratory rats
Source: Cell Genom. 2024 Mar 26;4(4):100527. doi: 10.1016/j.xgen.2024.100527 (PMC11019364; doi:10.1016/j.xgen.2024.100527)
Supplement: Document S3. Article plus supplemental information [file mmc18.pdf]

Cell Genomics

Resource

A revamped rat reference genome improves the discovery of genetic diversity in laboratory rats

Graphical abstract

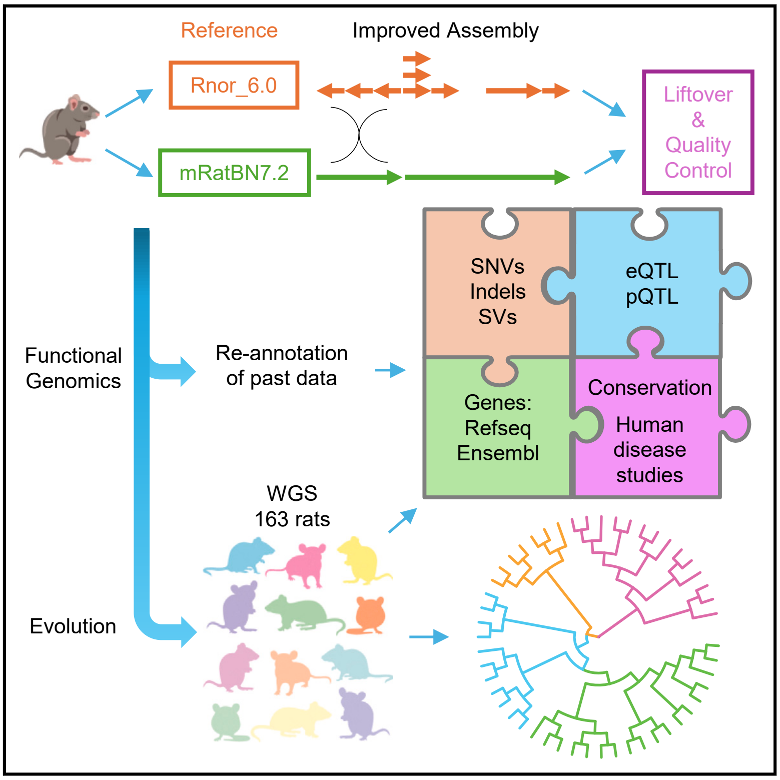

Authors

Tristan V. de Jong, Yanchao Pan, Pasi Rastas, ..., Robert W. Williams, Jun Z. Li, Hao Chen

Correspondence

junzli@med.umich.edu (J.Z.L.),
hchen@uthsc.edu (H.C.)

In brief

de Jong et al. evaluated the seventh assembly of the rat reference genome, mRatBN7.2, and found that it reduces base-level errors and increases contiguity, although some misassemblies remain. Gene annotations are now more complete. Analysis of whole genomes representing 120 rat strains/substrains revealed 20 million sequence variations. Phylogenetic analysis refined ancestral relationships among these strains. In addition, a new rat genetic map, along with annotated transcription start sites and alternative polyadenylation sites based on mRatBN7.2, is provided.

Highlights

- mRatBN7.2 is a rat reference genome with improved contiguity and accuracy
- Gene annotations, from both RefSeq and Ensembl, are improved with mRatBN7.2
- Our analysis of 120 strains/substrains of rats found 20 million sequence variations
- A refined phylogenetic tree reveals the relationships between laboratory rats

de Jong et al., 2024, Cell Genomics *4*, 100527
April 10, 2024 © 2024 The Authors. Published by Elsevier Inc.
https://doi.org/10.1016/j.xgen.2024.100527
CellPress

Cell Genomics

CellPress
OPEN ACCESS

Resource

A revamped rat reference genome improves the discovery of genetic diversity in laboratory rats

Tristan V. de Jong,[1,27] Yanchao Pan,[2,27] Pasi Rastas,[3] Daniel Munro,[4,5] Monika Tutaj,[6,7] Huda Akil,[8] Chris Benner,[9] Denghui Chen,[4] Apurva S. Chitre,[4] William Chow,[10] Vincenza Colonna,[11,12] Clifton L. Dalgard,[13] Wendy M. Demos,[6,7] Peter A. Doris,[14] Erik Garrison,[12] Aron M. Geurts,[6] Hakan M. Gunturkun,[1] Victor Guryev,[15] Thibaut Hourlier,[16] Kerstin Howe,[10] Jun Huang,[1] Ted Kalbfleisch,[17] Panjun Kim,[12] Ling Li,[12,18]

(Author list continued on next page)

[1]Department of Pharmacology, Addiction Science, and Toxicology, University of Tennessee Health Science Center, Memphis, TN, USA
[2]Department of Human Genetics, University of Michigan, Ann Arbor, MI, USA
[3]Institute of Biotechnology, University of Helsinki, Helsinki, Finland
[4]Department of Psychiatry, University of California San Diego, San Diego, CA, USA
[5]Department of Integrative Structural and Computational Biology, Scripps Research, San Diego, CA, USA
[6]Department of Physiology, Medical College of Wisconsin, Milwaukee, WI, USA
[7]Rat Genome Database, Medical College of Wisconsin, Milwaukee, WI, USA
[8]Michigan Neuroscience Institute, University of Michigan, Ann Arbor, MI, USA
[9]Department of Medicine, University of California San Diego, San Diego, CA, USA
[10]Tree of Life, Wellcome Sanger Institute, Cambridge, UK

(Affiliations continued on next page)

SUMMARY

The seventh iteration of the reference genome assembly for *Rattus norvegicus*—mRatBN7.2—corrects numerous misplaced segments and reduces base-level errors by approximately 9-fold and increases contiguity by 290-fold compared with its predecessor. Gene annotations are now more complete, improving the mapping precision of genomic, transcriptomic, and proteomics datasets. We jointly analyzed 163 short-read whole-genome sequencing datasets representing 120 laboratory rat strains and substrains using mRatBN7.2. We defined ~20.0 million sequence variations, of which 18,700 are predicted to potentially impact the function of 6,677 genes. We also generated a new rat genetic map from 1,893 heterogeneous stock rats and annotated transcription start sites and alternative polyadenylation sites. The mRatBN7.2 assembly, along with the extensive analysis of genomic variations among rat strains, enhances our understanding of the rat genome, providing researchers with an expanded resource for studies involving rats.

INTRODUCTION

Rattus norvegicus has been used in many fields of study related to human disease.[1] The earliest studies using brown rats appeared in the early 1800s[2,3] The Wistar rats, the ancestor of many laboratory strains, were bred for scientific research in 1906.[4] Over 4,000 inbred, outbred, congenic, mutant, and transgenic strains have been created and are documented in the Rat Genome Database (RGD).[5] Approximately 500 are available from the Rat Resource and Research Center.[6] Several genetic reference populations, including the HXB/BXH[7] and FXLE/LEXF[8] recombinant inbred (RI) families, are also available. Both families, together with 30 diverse classical inbred strains,[9] are now part of the Hybrid Rat Diversity Panel (HRDP), which can be used to quickly generate any of over 10,000 isogenic and replicable F1 hybrids—all of which are now essentially sequenced. The outbred N/NIH heterogeneous stock (HS) rats, derived from eight inbred strains,[10] have been increasingly used for fine mapping of physiological and behavioral traits.[11–15] To date, RGD has annotated nearly 2,400 rat quantitative trait loci (QTLs),[16] mapped using F2 crosses, RI families, and HS rats.

The *Rattus norvegicus* genome was sequenced shortly after the genomes of *Homo sapiens* and *Mus musculus*.[17] The inbred Brown Norway (BN/NHsdMcwi) strain, derived by many generations of sibling matings of stock originally from a pen-bred colony of wild rats,[4] was used to generate the reference. Several updates were released over the following decade.[18–20] Since 2014, most rat genomic and genetic research used the incomplete and problematic Rnor_6.0 assembly.[21,22] mRatBN7.2 was created in 2020 by the Darwin Tree of Life/Vertebrate Genome Project (VGP) as the new genome assembly of the BN/NHsdMcwi rat.[23] The Genome Reference Consortium (GRC) (https://www.ncbi.nih.gov/grc/rat) has adopted mRatBN7.2 as the official rat reference genome.

Here, we report extensive analyses of the improvements in mRatBN7.2 compared with Rnor_6.0. To assist the rat research

This is an open access article under the CC BY license (http://creativecommons.org/licenses/by/4.0/).

Spencer Mahaffey,[19] Fergal J. Martin,[16] Pejman Mohammadi,[20,21] Ayse Bilge Ozel,[2] Oksana Polesskaya,[4] Michal Pravenec,[22] Pjotr Prins,[12] Jonathan Sebat,[4] Jennifer R. Smith,[6,7] Leah C. Solberg Woods,[23] Boris Tabakoff,[19] Alan Tracey,[10] Marcela Uliano-Silva,[10] Flavia Villani,[12] Hongyang Wang,[24] Burt M. Sharp,[12] Francesca Telese,[4] Zhihua Jiang,[24] Laura Saba,[19] Xusheng Wang,[12,18] Terence D. Murphy,[25] Abraham A. Palmer,[4,26] Anne E. Kwitek,[6,7] Melinda R. Dwinell,[6,7] Robert W. Williams,[12] Jun Z. Li,[2,*] and Hao Chen[1,28,*]

[11]Institute of Genetics and Biophysics, National Research Council, Naples, Italy
[12]Department of Genetics, Genomics and Informatics, University of Tennessee Health Science Center, Memphis, TN, USA
[13]Department of Anatomy, Physiology & Genetics, The American Genome Center, Uniformed Services University of the Health Sciences, Bethesda, MD, USA
[14]The Brown Foundation Institute of Molecular Medicine, Center for Human Genetics, University of Texas Health Science Center, Houston, TX, USA
[15]Genome Structure and Ageing, University of Groningen, UMC, Groningen, the Netherlands
[16]European Molecular Biology Laboratory, European Bioinformatics Institute, Wellcome Genome Campus in Hinxton, Cambridgeshire, UK
[17]Gluck Equine Research Center, Department of Veterinary Science, University of Kentucky, Louisville, KY, USA
[18]Center for Proteomics and Metabolomics, St. Jude Children's Research Hospital, Memphis, TN, USA
[19]Department of Pharmaceutical Sciences, Skaggs School of Pharmacy and Pharmaceutical Sciences, University of Colorado Anschutz Medical Campus, Aurora, CO, USA
[20]Center for Immunity and Immunotherapies, Seattle Children's Research Institute, Seattle, WA, USA
[21]Department of Pediatrics, University of Washington School of Medicine, Seattle, WA, USA
[22]Institute of Physiology, Czech Academy of Sciences, Prague, Czechia
[23]Department of Internal Medicine, Section on Molecular Medicine, Wake Forest University School of Medicine, Winston-Salem, NC, USA
[24]Department of Animal Sciences, Washington State University, Pullman, WA, USA
[25]National Center for Biotechnology Information, National Library of Medicine, National Institutes of Health, Bethesda, MD, USA
[26]Institute for Genomic Medicine, University of California San Diego, La Jolla, CA, USA
[27]These authors contributed equally
[28]Lead contact
*Correspondence: junzli@med.umich.edu (J.Z.L.), hchen@uthsc.edu (H.C.)
https://doi.org/10.1016/j.xgen.2024.100527

community in transitioning to mRatBN7.2, we conducted a broad analysis of a whole-genome sequencing (WGS) dataset of 163 samples from 120 inbred rat strains and substrains. Joint variant calling led to the discovery of 19,987,273 high-quality variants from 15,804,627 sites. Additional resources created during our analysis included a rat genetic map, a comprehensive phylogenetic tree for the 120 (sub)strains, and extensive annotation of identified genes, including their transcription start sites (TSSs) and alternative polyadenylation (APA) sites. This new assembly and its associated resources create a more solid platform for research on the many dimensions of physiology, behavior, and pathobiology of rats and for more reliable and meaningful translation of findings to human populations.

RESULTS

High structural and base-level accuracy of mRatBN7.2

All sequencing data for mRatBN7.2 were generated from a male BN rat from the Medical College of Wisconsin (BN/NHsdMcwi, generation F61). The assembly is based on integrating data across multiple technologies, including long-read sequencing (PacBio CLR), 10X linked-read sequencing, BioNano DLS optical map, and Arima HiC. After automated assembly, manual curation corrected most of the apparent discrepancies among data types.[24] While mRatBN7.2 contains an alternative pseudohaplotype (GCA_015244455.1), we focused our analysis on the primary assembly (GCF_015227675.2).

Over the last six iterations of the rat reference, genome continuity has improved incrementally (Table S1). Contig N50, one measure of assembly quality, has been ~30 kb between rn1 to rn5. Rnor_6.0 was the first assembly to include some long-read data and improved contig N50 to 100.5 kb. mRatBN7.2 further improved N50 to 29.2 Mb (Figure S1). Although this measure of contiguity lags slightly behind the mouse reference genome (contig N50 = 59 Mb in GRCm39, released in 2020) and far behind the first telomere-to-telomere human genome (CHM13) (Table 1), it still marks a large improvement (~290 times higher) over Rnor_6.0 (Figure S2). In another measure, the number of contigs in mRatBN7.2 was reduced by 100-fold compared with Rnor_6.0 and is approaching the quality of GRCh38 for humans and GRCm39 for mice (Table 1).

Although aligning Rnor_6.0 with mRatBN7.2 showed a high level of structural agreement (Figures 1A and S3), we identified 36,500 discordant segments between these two assemblies that are longer than 50 bp (Figure S4). To evaluate these differences, we generated a genetic map (Table S2) using data from 378 families of 1,893 HS rats based on the recombination frequency between 150,835 markers.[15] Comparing the order of the markers and their location on the reference confirmed that the order and orientation of genomic segments are much more accurate in mRatBN7.2. For example, there is a 17.2 Mb inverted segment at proximal Chr 6 between Rnor_6.0 and mRatBN7.2 (Figure 1B). It remains inverted when the genetic distance is plotted with marker location on Rnor_6.0 (Figure 1C) but is resolved when using marker locations on mRatBN7.2 (Figure 1D). This was also true for several other regions (e.g., Figures 1E–1G for Chr 19 and Figures S5 and S6 for all autosomes). These data indicate that most of the segment-wise differences between the two assemblies are due to errors in Rnor_6.0.

Table 1. Global statistics for the rat, mouse, and human reference genomes

	Rnor_6.0	mRatBN7.2	GRCm39	GRCh38	CHM13
Year published	2014	2021	2020	2014	2021
Total sequence length	2,870,182,909	2,647,915,728	2,728,222,451	3,209,286,105	3,054,832,041
Total ungapped length	2,729,984,219	2,626,580,772	2,654,621,837	3,049,316,098	3,054,832,041
No. of scaffolds	953	176	61	455	24
Scaffold N50	145,729,302	135,012,528	130,530,862	145,138,636	154,259,566
Scaffold L50	8	8	9	9	8
No. of contigs	75,695	757	347	1,431	24
Contig N50	100,511	29,198,295	59,462,871	56,413,054	154,259,566
Contig L50	7,346	27	15	19	8
Total no. of chromosomes and plasmids	23	23	22	25	24

Genomes are downloaded from UCSC Goldenpath. Summary statistics are calculated based on the fasta files of each release using QUAST.[25]

We mapped linked-read WGS data for 36 samples from the HXB/BXH family of strains against both Rnor_6.0 and mRatBN7.2. These included four samples of the reference strain (BN/NHsdMcwi), two samples from the two parental strains—SHR/OlaIpcv and BN-Lx/Cub—and all 30 extent HXB/BXH progeny strains. The mean read depth of the entire HXB dataset, including all parentals, is 105.5× (range 23.4–184.5). From mRatBN7.2 to Rnor_6.0, the fraction of reads mapped to the reference increased by 1%–3% (Figure 2A), and regions of the genome with no coverage decreased by ∼2% (Figure 2B). Genetic variants (SNPs and indels) were identified using *Deepvariant*[26] and jointly called for the 36 samples using *GLnexus.*[27] After quality filtering (qual $\geq$ 30), we identified 8,286,401 SNPs and 3,527,568 indels in Rnor_6.0. Corresponding numbers for mRatBN7.2 are 5,088,144 SNPs and 1,615,870 indels (Figures 2C and 2D). Surprisingly, variants shared by all 36 samples—either homozygous in all samples or heterozygous in all samples—were more abundant when aligned to Rnor_6.0 (1,310,902) than to mRatBN7.2 (143,254) (Figures 2E and 2F). Because we included sequence data from 4 BN/NHsdMcwi rats, including those used for both Rnor_6.0 and mRatBN7.2, the most parsimonious explanation is that these shared variants are due to the wrong nucleotide sequence being recorded as the reference allele in the references. Therefore, mRatBN7.2 reduced base-level errors by 9.2-fold compared with Rnor_6.0.

Improved gene model annotations in mRatBN7.2

mRatBN7.2-based gene annotations, based on transcriptomic datasets and newly revised multi-species sequence alignments, were released in RefSeq 108 and Ensembl 105. We compared the Rnor6.0 and mRatBN7.2 annotation sets in RefSeq using BUSCO v.4.1.4,[28] focusing on the glires_odb10 dataset of 13,798 genes that are expected to occur in single copy in rodents. Instead of generating a *de novo* annotation with BUSCO using the Augustus or MetaEuk gene predictors, we used proteins from the new annotations, picking one longest protein per gene for analysis. BUSCO reported 98.7% of the glires_odb10 genes as complete in RefSeq 108 (single copy [S] 97.2%, duplicated [D] 1.5, fragmented [F] 0.4%, missing [M] 0.9%). In comparison, analysis of Rnor_6.0 with National Center for Biotechnology Information (NCBI)'s annotation pipeline using the same code and evidence sets showed a slightly higher fraction of fragmented and missing genes and more than double the rate of duplicated genes (S 94.4%, D 3.3%, F 0.9%, M 1.4%). The Ensembl annotation was evaluated using BUSCO v.5.3.2 with the lineage dataset "glires_odb10/2021-02-19." The "complete and single-copy BUSCO" score improved from 93.6% to 95.3%, and the overall score improved from 96.5% in Rnor_6.0 to 97.0% in mRatBN7.2. Both annotation sets demonstrate that mRatBN7.2 is a better foundation for gene annotation, with improved representation of protein-coding genes.

RefSeq (release 108) annotated 42,167 genes, each associated with a unique NCBI GeneID. These included 22,228 protein-coding genes, 7,888 long non-coding RNA (lncRNA), 1,288 small nucleolar RNA (snoRNA), 1,026 small-nuclei RNA (snRNA), and 7,972 pseudogenes. Ensembl (release 107) annotation contained 30,559 genes identified with unique "ENSRNOG" stable IDs. These included 23,096 protein-coding genes, 2,488 lncRNA, 1,706 snoRNA, 1,512 snRNA, and 762 pseudogenes. Although the two transcriptomes have similar numbers of protein-coding genes, RefSeq annotates many more lncRNA (more than 3×) and pseudogenes (almost 10×), and only RefSeq annotates tRNA.

Comparing individual genes across the two annotation sets, Ensembl BioMart reported 23,074 Ensembl genes with an NCBI GeneID, and NCBI Gene reported 24,115 RefSeq genes with an Ensembl ID (Table S3). We note that 2,319 protein-coding genes were annotated with different names (Table S4) by Ensembl and RefSeq. While many of these were caused by the lack of formal gene names in one of the sources, some of them were annotated with distinct names. For example, some widely studied genes, such as *Bcl2*, *Cd4*, *Adrb2*, etc., were not annotated with GeneID in Ensembl BioMart. We found that 18,722 gene symbols were annotated in both RefSeq and Ensembl. Among the 22,247 gene symbols found only in RefSeq, 20,185 were genes without formal names. Further, RefSeq contained a total of 100,958 transcripts, with an average of 2.9 (range: 1–51) transcripts per gene. Ensembl had 54,991 transcripts, with an average of 1.8 (range: 1–10) transcripts per gene (Figure S7).

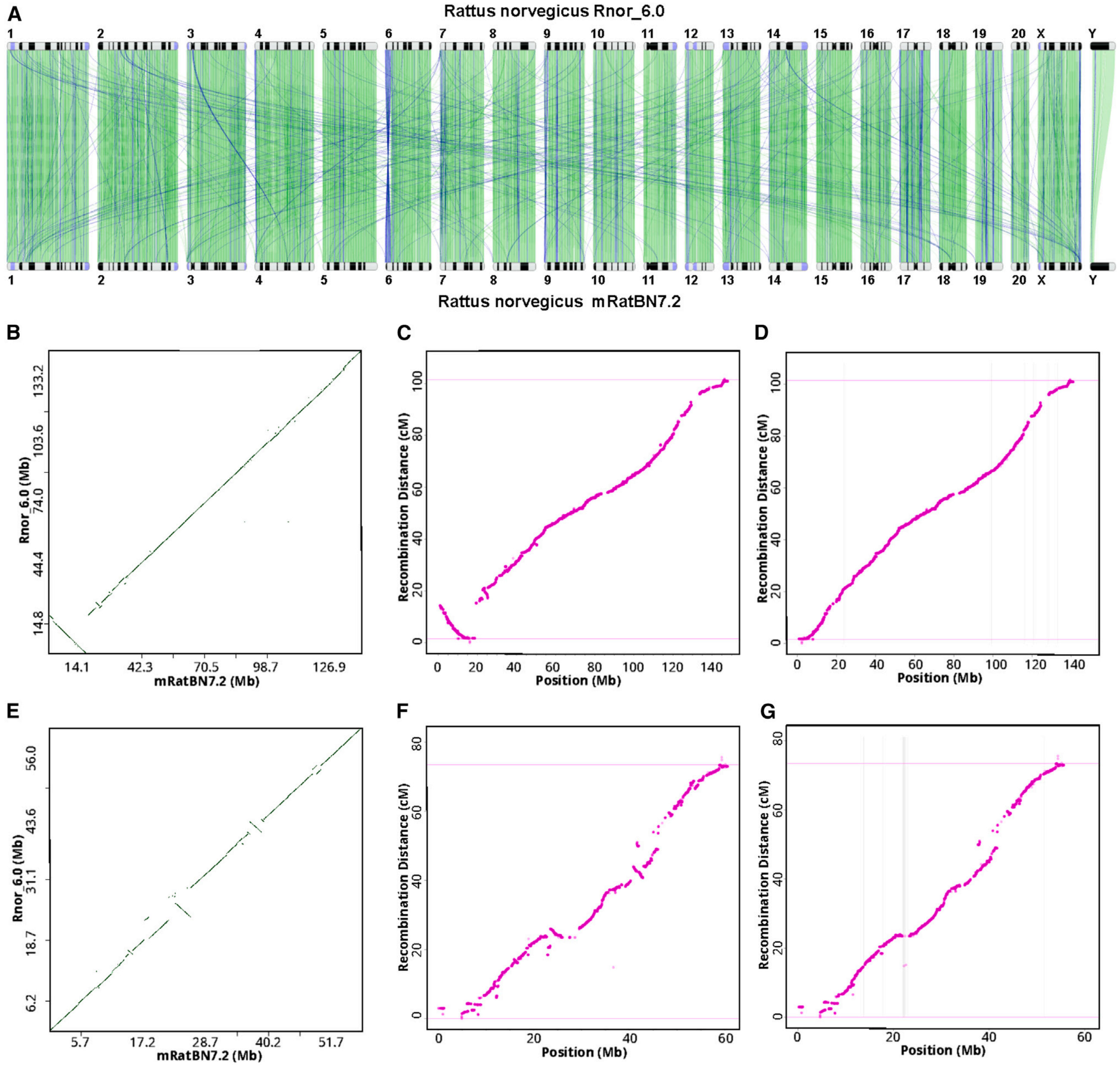

Figure 1. mRatBN7.2 corrects structural errors in Rnor_6.0

(A) Genome-wide comparison between Rnor_6.0 and mRatBN7.2 showed many structural differences, such as a large inversion at proximal Chr 6 and many translocations between chromosomes. Image generated using the NCBI Comparative Genome Viewer. Numbers indicate chromosomes. Green lines indicate sequences in the forward alignment. Blue lines indicate reverse alignment.

(B) The large inversion on proximal Chr 6 is shown in a dot plot between Rnor_6.0 and mRatBN7.2.

(C) A rat genetic map generated using 150,835 binned markers from 1,893 heterogeneous stock rats showed an inversion at proximal Chr 6 between genetic distance and physical distance based on Rnor_6.0, indicating that the inversion is caused by assembly errors in Rnor_6.0.

(D) Marker order and genetic distance from the genetic map on Chr 6 are in agreement with physical distance based on mRatBN7.2, indicating that the mis-assembly is fixed.

(E–G) Genetic map confirms that many assembly errors on Chr 19 in Rnor_6.0 are fixed in mRatBN7.2.

mRatBN7.2 improved the mapping of WGS data

We compared mapping results of long-read datasets. Unmapped read numbers declined from 298,422 in Rnor_6.0 to 260,947 in mRatBN7.2, a 12.6% reduction using a PacBio CLR dataset from an SHR rat with a total of 4,508,208 reads. Mapping of Nanopore data from one outbred HS rat (~55× coverage) showed much lower secondary alignment—from ~10 million in Rnor_6.0 to 4 million in mRatBN7.2. Similar to the linked-read

CellPress
OPEN ACCESS

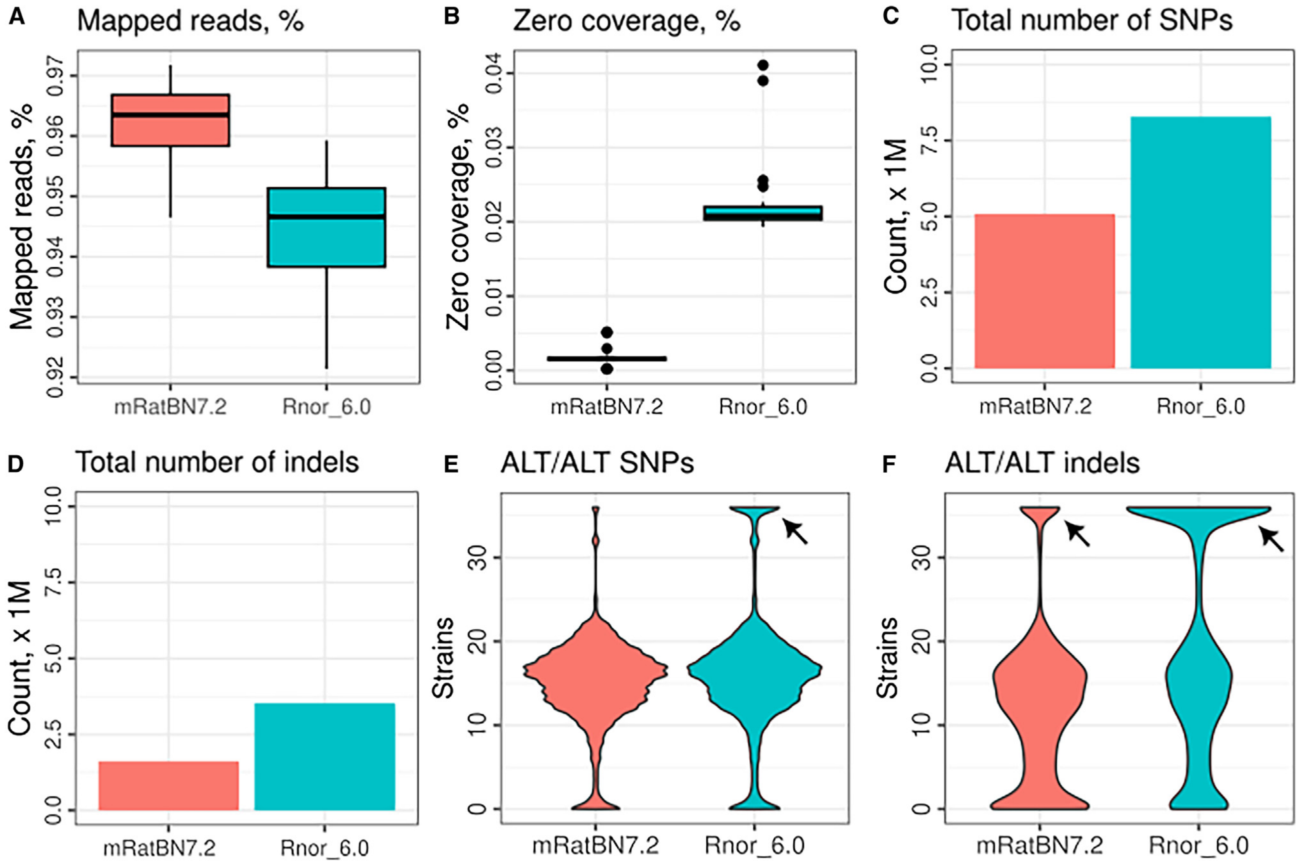

Figure 2. mRatBN7.2 improved mapping statistics of whole-genome sequencing data

Summary statistics from mapping 36 HXB/BXH WGS samples against Rnor_6.0 and mRatBN7.2 were compared. Using mRatBN7.2 increased the percentage of reads mapped (A), reduced regions on the reference genome with zero coverage (B), total number of SNPs (C), and indels (D). The presence of a large number of SNPs (E) and indels (F) that are shared by all samples (arrows), including BN/NHsdMcwi, indicates that they are base-level errors in the reference genome.

data, structural variants (SVs) detected in the Nanopore dataset were reduced (Figure S8) using mRatBN7.2.

We also evaluated the effect of reference genome on the identification of SVs. We identified 19,538 unique SVs against Rnor_6.0 in the HXB dataset. In contrast, only 5,458 SVs were found using mRatBN7.2 (see STAR Methods, key resources table, https://doi.org/10.5281/zenodo.10398554). We illustrated these findings by focusing on a small panel of eight samples (Figure S9).

mRatBN7.2 improved the analysis of transcriptomic and proteomic data

We analyzed an RNA sequencing (RNA-seq) dataset of 352 HS rat brains (see https://RatGTEx.org) to compare the effect of the reference genome. The fraction of reads aligned to the reference increased from 97.4% in Rnor_6.0 to 98.3% in mRatBN7.2. The average percentage of reads aligned concordantly only once to the reference genome increased from 89.3% to 94.6%. The average percentage of reads aligned to the Ensembl transcriptome increased from 67.7% to 74.8%. Likewise, we examined the alignment of RNA-seq data from ribosomal RNA-depleted total RNA and short RNA in the HRDP. For the total RNA (>200 bp) samples, alignment to the genome increased from 92.4% to 94.0%, while the percentage of reads aligned concordantly only once to the reference increased from 76.1% to 79.2%. For the short RNA (<200 bp; targeting transcripts 20–50 bp long), genome alignment increased from 95.0% to 96.2% and unique alignment increased from 33.2% to 35.9%. In an snRNA-seq dataset (Figures S10A–S10C), the percentage of reads that mapped confidently to the reference increased from 87.4% on Rnor_6.0 to 91.4% on mRatBN7.2. In contrast, reads mapped with high quality to intergenic regions were reduced from 24.5% on Rnor_6.0 to 10.3% on mRatBN7.2.

We analyzed datasets containing information about transcript start and polyadenylation in a capped short RNA-seq dataset. The rate of unique alignment of TSSs to the reference genome increased by 5% in mRatBN7.2 (Figures S10D and S10E). In this dataset, we identified 42,420 TSSs when using Rnor_6.0 and 44,985 sites when using mRatBN7.2 (see STAR Methods, key resources table, https://doi.org/10.5281/zenodo.10398387). We analyzed 83 whole-transcriptome termini site sequencing[29] datasets using total RNA derived from rat brains and found that 76.97% were mapped against Rnor_6.0, while 80.49% were mapped against mRatBN7.2 (Table S5). We identified 167,136

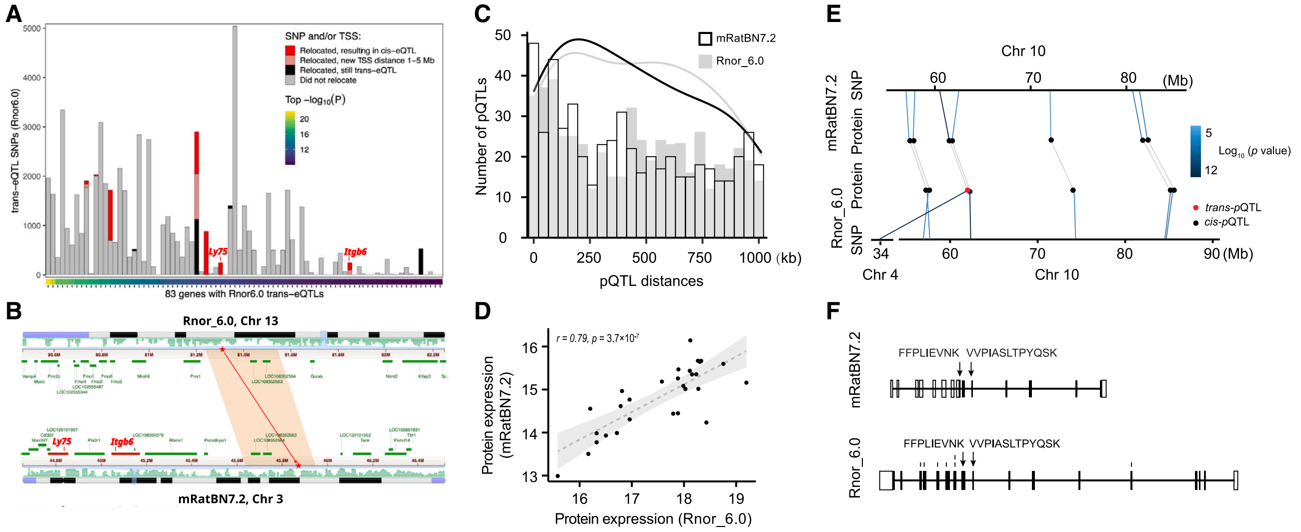

Figure 3. mRatBN7.2 improves eQTL and proteomic analysis

Genome misassembly is associated with increased rates of calling spurious *trans*-eQTLs.

(A) Each column represents a gene for which at least one *trans*-eQTL was found at $p < 1 \times 10^{-8}$ using Rnor_6.0. The color of bars indicate the number of *trans*-eQTL SNP-gene pairs in which the SNP and/or gene transcription start site (TSS) relocated to a different chromosome in mRatBN7.2 and whether the relocation would result in a reclassification to *cis*-eQTL (TSS distance < 1 Mb) or ambiguous (TSS distance is between 1–5 Mb).

(B) Genomic location of one relocated *trans*-eQTL SNP from (A). The SNP is in a segment of Chr 13 in Rnor_6.0 that was relocated to Chr 3 in mRatBN7.2 (red stars), reclassifying the eQTL from *trans*-eQTL to *cis*-eQTL for both *Ly75* and *Itgb6* genes (red bars).

(C) Histogram showing the distance between *cis*-pQTLs and TSS of the corresponding proteins. The distances of pQTLs in mRatBN7.2 tend to be closer than those in Rnor_6.0.

(D) An example of *trans*-pQTL in Rnor_6.0 was detected as a *cis*-pQTL in mRatBN7.2.

(E) Correlation of expression of the protein (the example in B) in Rnor_6.0 and mRatBN7.2.

(F) Different annotations of the exemplar gene in Rnor_6.0 and mRatBN7.2.

APA sites using Rnor_6.0 and 73,124 APA sites using mRatBN7.2 (see STAR Methods, key resources table, https://doi.org/10.5281/zenodo.10398476). For Rnor_6.0, only 76.26% APA sites were assigned to the genomic regions with 18,177 annotated genes (Table S5). In contrast, 81.67% APA sites were mapped to 20,102 annotated genes on mRatBN7.2 (Table S5).

We examined the impact of the upgraded reference assembly on the yield and relative numbers of expression QTL (eQTL) using a large RNA-seq dataset for nucleus accumbens.[30] We identified associations that would be labeled as *trans*-eQTLs using one reference but *cis*-eQTL using the other, due to relocation of the SNP and/or the TSS. The expectation is that assembly errors will give rise to spurious *trans*-eQTLs. We found seven genes associated with one or more strong *trans*-eQTL SNPs using Rnor_6.0 that converted to *cis*-eQTLs using mRatBN7.2 (Figure 3). This constitutes 5.2% (3,261 of 63,148) of the Rnor_6.0 *trans*-eQTL SNP-gene pairs. In contrast, only 0.01% (51 of 404,302) of the Rnor_6.0 *cis*-eQTL SNP-gene pairs became *trans*-eQTLs when using mRatBN7.2. Given the much lower probability of a distant SNP-gene pair remapping to be in close proximity than vice versa under a null model of random relocations, this demonstrates a clear improvement in the accuracy and interpretation of eQTLs when using mRatBN7.2.

To evaluate the effect of reference genome on proteome data, we analyzed a set of data from 29 HXB/BXH strains. At a peptide-identification false discovery rate (FDR) of 1%, we identified and quantified 8,002 unique proteins on Rnor_6.0 compared with 8,406 unique proteins on mRatBN7.2 (5% increase). For protein local expression quantitative trait locus (i.e., *cis*-pQTL), 536 were identified using Rnor_6.0 and 541 were identified using mRatBN7.2 at FDR < 5%. Distances between pQTL peaks and the corresponding gene start site tended to be shorter on mRatBN7.2 than on Rnor_6.0 (Figure 3C). Similar to eQTLs, four proteins with *trans*-pQTL in Rnor_6.0 were converted to *cis*-pQTL using mRatBN7.2. For example, RPA1 protein (Chr10:60,148,794–60,199,949 bp in mRatBN7.2) mapped as a significant *trans*-pQTL ($p = 1.71 \times 10^{-12}$) on Chr 4 in Rnor_6.0 but as a significant *cis*-pQTL using mRatBN7.2 ($p = 4.12 \times 10^{-6}$) (Figure 3E). In addition, the expression of RPA1 protein displayed a high correlation between mRatBN7.2 and in Rnor_6.0 ($r = 0.79$; $p = 3.7 \times 10^{-7}$) (Figure 3D). Lastly, the annotations for RPA1 were different between the two references (Figure 3F).

WGS mapping data suggesting potential errors remaining in mRatBN7.2

We analyzed a WGS dataset of 163 inbred rats containing 12 BN samples and 151 non-BN samples, which represented two RI panels and more than 30 inbred strains. After read mapping to mRatBN7.2 and variant calling, we used the anomalies in the genotype data to further assess the quality of mRatBN7.2. This analysis was performed at two levels to detect issues at both the segment level and the individual nucleotide level.

First, we analyzed segment-wise distribution patterns of heterozygous genotypes and no-calls. Since inbred lines are expected

CellPress
OPEN ACCESS

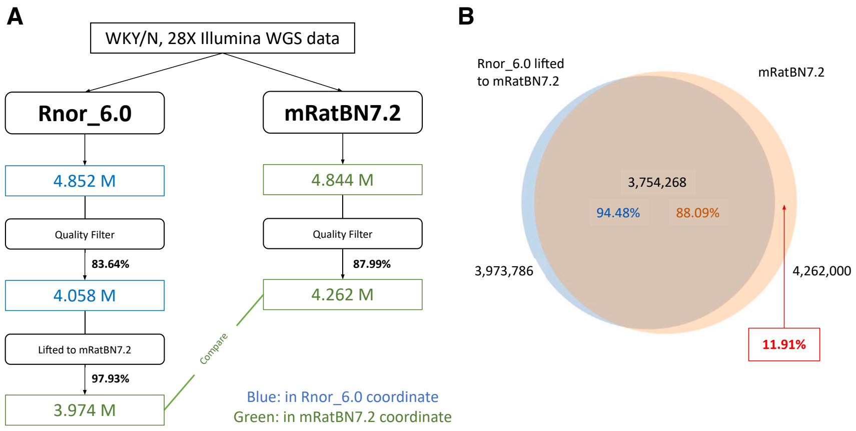

Figure 4. Using WGS data to assess the quality of the Liftover from Rnor_6.0 to mRatBN7.2

(A) Overview of the workflow using a real WGS sample from a WKY rat. A higher portion of variants passed the quality filter for mRatBN7.2. Among them, 97.93% of the variants were liftable from Rnor_6.0 to mRatBN7.2.

(B) The overlap between variants lifted from Rnor_6.0 and variants obtained by direct mapping sequence data to mRatBN7.2. Approximately 11.9% of the variants that were found from direct mapping were missing from the Liftover.

to show a negligible number of heterozygous sites, genomic regions with an unusually high density of heterozygous genotypes may indicate a segment-wise assembly error. For example, if two segments are tandem repeats of each other and have been "folded" into a single segment in the reference assembly, twice as many reads will map to this region and will produce a high density of heterozygous or multiple-allelic sites. We focused on the 12 BN samples, because BN is the basis of the reference. In all, we identified 673 such "flagged regions," with an average length of 52,199 bp, which collectively covered ~1.4% of the genome (Table S6). This rate is much reduced from that of Rnor_6.0.[22] Furthermore, regions with high heterozygosity were observed among the 151 non-BN samples outside the list of 673 flagged regions. These regions are likely to reflect shared structural variants in the non-BN samples.

We further examined per-site anomalies by identifying sites with Alt/Alt genotypes in most of the 163 strains, including the 12 BN strains. These 129,186 shared variant sites consisted of 33,550 SNPs and 95,636 indels (Table S7). Among them, 117,901 were homozygous alternative genotypes and 11,285 were heterozygous in more than 156 samples (Figure S11). The read depth was 32.2 ± 10.1 for homozygous and 66.3 ± 26.2 for heterozygous variants. Because all samples were inbred, the doubling of read depth for the heterozygous variants strongly suggests that they mapped to regions of the reference genome with collapsed repetitive sequences. This is supported by the location of these variants: homozygous SNPs were more evenly distributed, and heterozygous variants were often clustered in a region (Figure S12). These results indicated that many of the potential errors in mRatBN7.2 are caused by the collapse of repetitive sequences. Functionally, these regions of potential misassembly impact some well-studied genes, such as *Chat*, *Egfr*, *Gabrg2*, and *Grin2a*. After removing the likely errors, we annotated the VCF files resulting from the joint variant calling of 163 samples (see STAR Methods, key resources table, https://doi.org/10.5281/zenodo.10398344) and used these for subsequent analysis.

Complexities in transitioning from Rnor_6.0 to mRatBN7.2

Liftover tools can convert genomic coordinates between different versions of the reference assembly. However, Liftover can only return results for regions with one-to-one matches between the two references. We examined Liftover from Rnor_6.0 to mRatBN7.2 by testing a mock set of variant sites evenly distributed at 1 kb intervals. Of the 2.78 M simulated variants, 92.1% were successfully lifted. Thus, ~8% of the Rnor_6.0 genome does not have a unique match in BN7.2. These "unliftable" sites do not distribute evenly along Rnor_6.0 (Figure S13); rather, they tend to aggregate in regions with no credible match, i.e., lost in BN7.2 (Figure S14).

The rate of "Liftover loss" varies by the type of genomic feature, and as such will be study dependent. We used WGS data for one sample to call variants on Rnor_6.0, then lifted them to mRatBN7.2 and compared the results against the variants called directly on mRatBN7.2. Variants called on mRatBN7.2 had a higher proportion (87.99%) of passing the quality filter than those called on Rnor_6.0 (83.64%), and 97.93% of the variants called on Rnor_6.0 were liftable to mRatBN7.2 (Figure 4A). Among the lifted variant sites, 94.48% had a match in the variant set called directly. However, 11.91% of the variants called on mRatBN7.2 were absent in the lifted variant set (Figure 4B). Thus, complete remapping of the data to the new reference is preferable despite its time and resource costs.

A comprehensive survey of the genomic landscape of *Rattus norvegicus* based on mRatBN7.2

To facilitate a smooth transition to mRatBN7.2, we conducted a joint analysis of WGS data. We collected WGS for a panel of 163 rats (hereafter referred to as RatCollection, Table S8). RatCollection includes all 30 HXB/BXH strains, 25 FXLE/LEXF strains, and 33 other inbred strains. In total, we covered 88 strains (120 samples at the substrain level)—approximately 80% of the HRDP.

The mean depth of coverage of these samples was 60.4 ± 39.3. Additional sample statistics are provided in Table S9. After removing variants that were potential errors in mRatBN7.2 (above) and filtering for site quality (qual ≥ 30), we identified 19,987,273 variants (12,661,110 SNPs, 3,805,780 insertions, and 3,514,345 deletions) across 15,804,627 variant sites (Table 2). Across the genome, 89.4% of the sites were bi-allelic, and the mean variant density was 5.96 ± 2.20/kb (mean ± SD). The highest variant density of 30.5/kb was found on Chr 4 at 98 Mb (Figure S15). Most (97.9% ± 1.4%) of the variants were

Table 2. Genetic variants in laboratory populations

	Variants sites	Variants	Variant type: SNPs	Indels	Mixed[a]	Predicted impact: High	Low	Moderate	Alt/Alt homozygous % (mean ± SD)	Genotype qual (mean ± SD)
RatCollection	15,804,627	19,987,273	12,661,110	7,313,702	12,461	18,646	262,685	125,523	97.9 ± 1.4	70.1 ± 21.2
HS progenitors	12,418,243	16,438,302	9,947,112	6,479,485	11,705	6,980	205,316	94,659	98.5 ± 0.3	71.4 ± 22.0
FXLE/LEXF[b]	9,183,562	13,070,345	7,036,182	6,023,391	10,772	13,988	143,623	66,186	96.8 ± 2.4	72.3 ± 21.0
HXB/BXH[b]	7,520,223	11,256,227	5,705,592	5,541,195	9,440	10,121	115,081	53,819	97.9 ± 1.2	72.2 ± 22.8
SS/SR	7,171,447	10,522,763	5,544,220	4,969,398	9,145	8,606	111,658	51,149	98.0 ± 0.9	72.3 ± 21.8
LL/LN/LH	6,923,575	10,112,755	5,433,556	4,670,291	8,908	4,142	111,040	52,364	98.5 ± 0.3	73.2 ± 21.4

The RatCollection includes 163 rats (88 strains and 32 substrains, with some biological replicates). The HRDP contains the HXB/BXH and FXLE/LEXF panels as well as 30 or so classic inbreds. Our analysis includes ~80% of the HRDP. The variants were jointly called using Deepvariant and GLNexus. Variant impact was annotated using SnpEff.
[a]Variants that are combinations of insertions, deletions, or SNPs.
[b]Including parental strains.

homozygous at the sample level, confirming the inbred nature of most strains, with a few exceptions (Figure S16).

To analyze the phylogenetic relationships of these 120 strains/substrains, we created an identity-by-state (IBS) matrix using 11,585,238 high-quality bi-allelic SNPs (Table S10). Distance-based phylogenetic trees of all strains and substrains are shown in Figure 5A. The mean IBS for classic inbred strains was 72.30% ± 2.35%, where BN has the smallest IBS (64.76%).

This phylogenetic tree included all major populations of laboratory rats, such as the eight inbred progenitor strains of the outbred HS rats (ACI/N, BN/SsN, BUF/N, F344/N, M520/N, MR/N, WKY/N, and WN/N).[10] The number of sites with a non-reference allele in each of these strains is shown in Figure 5B; WKY/N contributed the largest number of non-reference alleles (Figure 5C). The distribution of the variant sites from all eight strains across the chromosomes is shown in Figure 5D. Collectively, these 8 strains accounted for 78.6% of all non-reference alleles in the RatCollection. Conversely, 141,556 of these variant sites were not found in any other strains. Although none of these founder strains are alive today, based on IBS, we identified 6 living proxies of the HS progenitors that were over 99.5% similar to the original progenitor strains: ACI/EurMcwi, BN/NHsdMcwi, F344/DuCrl, M520/NRrrcMcwi, MR/NRrrc, and WKY/NHsd (Table S11). The best matches of the remaining strains were much less similar: BUF/Mna was the best approximation (73.6%) to BUF/N, and WAG/RijCrl was the closest (72.0%) to WN/N.

Our phylogenetic tree also included two families of RI rats. The HXB/BXH family was generated from SHR/OlaIpcv and BN-Lx/Cub. Together with their parental strains, we identified 7,520,223 variant sites in this population (Table 2). Approximately 24.1%–53.5% of the alleles at these sites of each RI strain were derived from SHR/OlaIpcv. While the majority of the strains were highly inbred, with close to 98% of the variants being homozygous (Figure S17), one exception was BXH2, in which 7.7% of variants were heterozygous (Figure S16), likely due to a recent breeding error. The LEXF/FXLE RI family was generated from LE/Stm and F344/Stm. We discovered 9,183,562 variant sites from the parental strains and 25 strains of this family (Table 2). We expect the final variant count of the LEXF/FXLE panel to be similar because both parental strains are included in our analysis. The overall rate of homozygous variants in the FXLE/LEXF family (96.8% ± 2.4%) was lower than in other inbred rats (Figure S16). In particular, 15.6% of the sites from the FXLE24 samples are heterozygous, indicating likely breeding errors.

In addition, our analysis also included sets of two or three strains generated by selective breeding for certain traits, such as the Dahl salt-snsitive (SS) and Dahl salt-resistant (SR) strains for studying hypertension. These two strains contain 7,171,447 variant sites compared with mRatBN7.2 (Table 2), with 1,024,283 variants unique to SR and 920,234 variants unique to SS. These strain-specific variants were found throughout the genome (Figure S18). A similar pattern was found for the Lyon hypertensive (LH), hypotensive (LL), and normotensive (LN) rats (Table 2) selected from outbred Sprague-Dawley rats for studying blood pressure regulation.[31] Only 281,972, 289,112, and 262,574 variants were unique to LH, LL, and LN, respectively. In agreement with a prior report,[32] these variants were clustered in a handful of genomic hotspots (Figure S19).

Impact of rat variants on genetic studies of human diseases

We used SnpEff (v.5.0e) to predict the impact of the 19,987,273 variants in the RatCollection based on RefSeq annotation. Among these, 18,646 variants near coding genes were predicted to have a high impact (i.e., causing protein truncation, loss of function, or triggering nonsense-mediated decay, etc.) on 6,667 genes, including 3,930 protein-coding genes (Table S12).

Among the predicted high-impact variants, annotation by RGD disease ontology identified 2,601 variants affecting 2,079 genes that were associated with 3,261 distinct disease terms. Cancer/tumor (878), psychiatric (612), intellectual disability (505), epilepsy (319), and cardiovascular (304) comprised the top 5 disease terms, with many genes associated with more than one disease term. A mosaic representation of the number of high-impact variants per disease term for each strain is shown in (Figure S20). The top disease categories for the two RI panels are comparable, including cancer, psychiatric disorders, and cardiovascular disease. The disease ontology annotations for

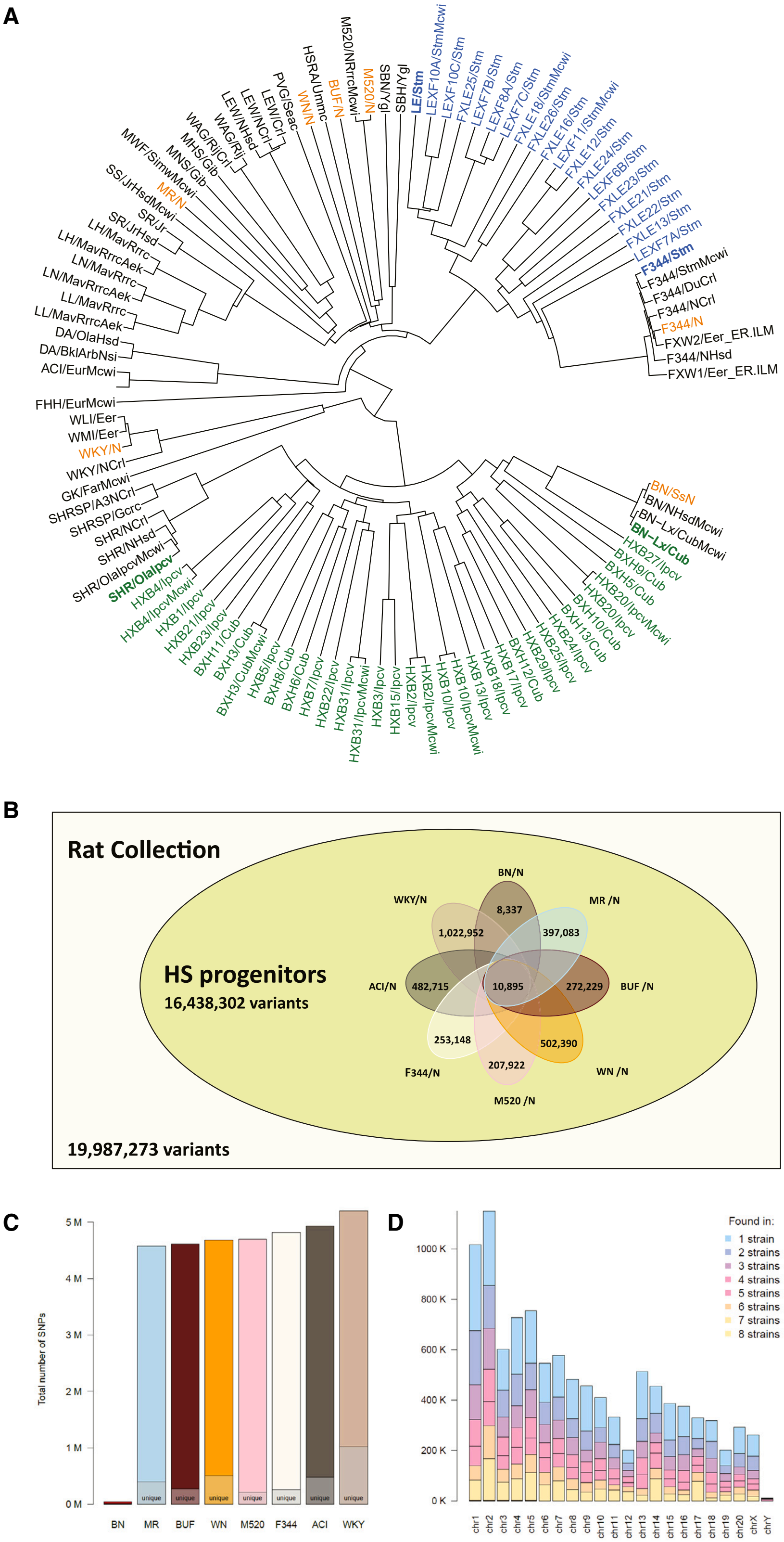

Figure 5. Phylogenetic relationship and genetic diversity of 120 strain/substrains of laboratory rats

(A) The phylogenetic tree was constructed using 11.6 million biallelic SNPs from 163 samples. Strains/substrains with duplicated samples were condensed. Strains highlighted with bold fonts are parental strains for RI panels. Green, HXB/BXH RI panel; blue, FXLE/LEXF RI panel; orange, progenitors of the HS outbred population.

(B) The HS progenitors contain 16,438,302 variants (i.e., 82.2% of the variants in our collection of 120 strain/substrains) based on analysis using mRatBN7.2. Among these, 10,895 are shared by all eight progenitor strains. The number of variants that are unique to each specific founder is noted.

(C) The total number of variants per strain, with the total number unique to each strain marked.

(D) The number of variants shared across strains per chromosome.

variants in the HXB/BXH, FXLE/LEXF RI panels, and SS/SR, as well as LL/LN/LH selective bred strains, are summarized in Figures S21 and S22 and Tables S13 and S14.

We further annotated genes using the human genome-wide association studies (GWASs) catalog.[33] Among the rat genes with high-impact variants, 2,034 have human orthologs with genome-wide significant hits associated with 1,393 mapped traits (Table S15). The most frequent variant type among these rat genes was frameshift (1,557 genes), followed by splice donor variant (136 genes) and gain of stop codon (116 genes). Although rats and humans do not share the same variants, strain with these variants can potentially be a useful model for related human diseases at the gene level.

DISCUSSION

We systematically evaluated mRatBN7.2 and confirmed that it improved assembly quality over its predecessor, Rnor_6.0. As a result, mRatBN7.2 improved the analysis of omics datasets and now enables powerful and efficient genome-to-phenome analysis of 20 million variants segregating in the laboratory rat.

The improvements in mRatBN7.2 are based on new assembly methods and long-read data.[23] However, the PacBio CLR reads used in mRatBN7.2 have lower base accuracy than Illumina short reads.[34,35] Although Illumina data were used to polish the assembly,[23] polishing methods do not correct all base-level errors.[34,36] Our joint analysis of 163 WGS datasets identified 129,186 sites with likely nucleotide errors in mRatBN7.2. Another source of potential error is the assembly method itself. mRatBN7.2 was assembled with VGP pipeline v.1.6,[23,37] which assembles the long reads into a diploid genome containing a primary and an alternative assembly.[38] This pipeline is well suited to assemble diploid genomes. When applied to BN/NHsdMcwi, a fully inbred rat, the pipeline classified some duplications with small variants as two haploids, resulting in a collapsed repeat.[39] A telomere-to-telomere assembly[40] is needed to fully resolve these issues.

Liftover enables direct translation of genomic coordinates between different references, thereby reducing the cost of transitioning to a new reference. We found that 92.05% of simulated variants and 97.93% of variants from a WKY/N sample identified on Rnor_6.0 were lifted successfully to mRatBN7.2. This difference is attributable to the large number of simulated variants located in regions of low complexity. While promising, we found that reanalysis of the original sequencing data using mRatBN7.2 discovered an additional 507,700 variant sites, located primarily in regions not present in Rnor_6.0. Thus, remapping to mRatBN7.2 is preferred over using Liftover even though the latter is more convenient.

To facilitate the transition for studies of specific rat models, we mapped WGS data of 88 strains (120 substrains and 163 samples) to mRatBN7.2. Joint analysis identified 20.0 million variants from 15.8 million sites. This analysis expanded many prior analyses of rat genomes. For example, Baud et al.[11] analyzed 8 strains against Rnor3.4 and found 7.9 million variants, Atanur et al.[41] analyzed 27 rat strains against Rnor3.4 and reported 13.1 million variants, and Hermsen et al.[42] analyzed 40 strains against Rnor5.0 and reported 12.2 million variants. Most recently, Ramdas et al.[22] analyzed 8 strains and reported 16.4 million variants. In addition to using the latest reference genome and an expanded number of strains, our pipeline depends on Deepvariant and GLNexus, which have been shown to improve call set quality, especially on indels.[27,43] Thus, our data provide the most comprehensive analysis of genetic variants in the laboratory rat population to date and, of comparative interest, is twice the variant count of the highly used mouse BXD family.[44,45]

This collection of rats represented a wide variety of rats that are used in genetics studies today, such as the full HXB/BXH panel and 27 strains/substrains of the FXLE/LEXF RI panel. Together with the inbred strains, they cover about 80% of the rats in the HRDP.[46] The new variant set from our analysis provide a much-needed boost in mapping precision.[8,46,47]

The outbred N/NIH HS rats are another widely used rat genetic mapping population.[13] Our analysis is in agreement with Ramdas et al.[22] in identifying 16 million variants in the progenitors. Updated genetic data will be useful in HS genetic mapping studies, such as for the imputation of variants. Although live colonies of the original NIH HS progenitor strains are no longer available, we identified six living substrains that are genetically almost identical to the original progenitors (99.5% IBS), while the closest match to the other two strains has IBS of approximately 70% (Table S11). These living substrains will be useful in many ways, such as studying the effect of variants identified in genetic mapping studies. Our analysis also included groups of inbred rats segregated by less than 2 million variants. For example, the LH/LN/LL family,[31,32] the SS/SR family (Table S14), as well as several pairs of near isogenic lines with distinctive phenotypes. These rats can be exploited to identify causal variants using reduced complexity crosses.[48] Updated genotyping data for these populations will benefit all ongoing studies that use these rats.

A trove of genetic associations has been established using laboratory rats. For example, studies using RI panels have identified loci that affect cardiovascular disease,[49] hypertension,[50,51] diabetes,[52] immunity,[52] tumorigenesis,[53] and tissue-specific gene expression profiles.[54] Using the outbred HS rats, Baud et al.[11] reported 355 QTLs associated with phenotypes representing six diseases. Other studies using the HS population have revealed genetic control of behavior,[15,55] obesity,[56] and metabolic phenotypes.[57] Many of these data are available from the RGD.[16] While all these data were analyzed using previous versions of the reference genome, reanalyzing them using updated genomic data could lead to novel discoveries.[58]

By generating F1 hybrids from these inbred lines with sequenced genomes, novel phenotypes can be mapped onto completely defined genomes: the 82 sequenced HRDP strains can produce any of 6,642 isogenic but entirely replicable F1 hybrids with completely defined genomes. Studies of these "sequenced" F1 hybrids avoid the homozygosity of the parental HRDP strains and enable a new phase of genome-phenome mapping and prediction.[44] While several genetic mapping studies using the HRDP are currently underway, the large number of variants in the HRDP (cf., the hybrid mouse diversity panel contains about 4 million SNPs[59]) and WGS-based genotype data will further encourage the use of this resource.

We identified 18,646 variations likely to have a high impact on 6,667 genes and many more variants predicted to have regulatory

effects on gene expression (Table S13). Some of these variants are supported by the literature. For example, the SS rats develop renal lesions with hypertension and have high impact mutations in *Capn1*[60] and *Procr*,[61] both associated with kidney injuries, as well as in *Klk1c12*, associated with hypertension.[62] These annotations identified many genes with variants that either result in a gain of STOP codon or cause a frameshift, which could lead to the loss of function. Strains harboring these variants can be explored to investigate the function of these genes.[63]

These functional annotations are highly relevant when the human ortholog of these genes is associated with certain traits in human GWASs (Table S15). For example, *CDHR3* is associated with smoking cessation.[64] A gain of STOP mutation in the *Cdhr3* gene was found in only one parent of both RI panels (SHR/OlaIpcv and LE/Stm). Using these RI panels to study the reinstatement of nicotine self-administration, a model for smoking cessation, will likely provide insights into the role of *CDHR3* in this behavior. Furthermore, a near-complete catalog of strain-specific alleles and functional prediction provide a stable source of potential candidate variants for interpreting genetic mapping results.

Our phylogenetic analysis agrees with those published previously[41,65] and confirmed that BN/NHsdMcwi is an outgroup to all other common laboratory rats (Figure 5A). This is consistent with its derivation from a pen-bred colony of wild-caught rats[4] and previous studies that included wild rats.[66] Thus, mapping sequence data from other strains to the BN reference yields a greater number of variants (but at slightly reduced mapping quality) than using a hypothetical reference that is genetically closer to the commonly used laboratory strains. This so-called reference bias has been observed in genomic[67] and transcriptomic data analyses.[68] It should be noted that no individual strain is a perfect representation of a population. Instead, the nascent field of pangenomics,[69] where the genomes of all strains can be directly compared with each other, provides a promising future in which all variants can be compared between individuals directly without the use of a single reference genome. While this pangenomic approach can be applied to short-read data,[70] it will be especially powerful when individual genomes are all assembled from long-read sequence data, some of which are already available.[71] This approach will enable a complete catalog of all genomic variants, including SVs and repeats, that differ between individuals.

Additional rat genomic resources we generated include a new rat genetic map with 150,835 markers. We envision that this map will have multiple applications. For example, this map can be integrated into the *de novo* assembly process, as demonstrated by the new assembly of the stickleback genome.[72,73] Unlike human and mouse genomes,[74] functional elements in the rat remain poorly annotated. Our analysis produced a list of TSS locations for genes expressed in the prefrontal cortex or nucleus accumbens and APA sites of genes expressed in the brain. These new datasets will further the study of gene regulatory mechanisms in rats.

Research using the *Rattus norvegicus* has made important contributions to the understanding of human physiology and diseases. An updated reference genome provides not only a valuable resource for future studies but also opportunities for analyzing existing data to gain new insights. The rich literature on laboratory rats, combined with the complex genomic landscape revealed in our survey, demonstrates that the rat is an excellent model organism for the next chapter of biomedical research.

Limitations of the study

While our comparative analyses reported many improvements in mRatBN7.2 over Rnor_6.0, we also found that mRatBN7.2 contains a few hundred segments with potential disassembly, as well as base-level errors. These segments affect gene annotation and the accuracy and completeness of Liftover. While we are confident in the informatic analysis of these segments, we did not empirically validate these findings. Future iterations of the rat reference genome need to reach telomere-to-telomere completion and eventually be expanded to a multi-strain pangenome reference.

STAR★METHODS

Detailed methods are provided in the online version of this paper and include the following:

- KEY RESOURCES TABLE
- RESOURCE AVAILABILITY
  - Lead contact
  - Materials availability
  - Data and code availability
- EXPERIMENTAL MODEL AND STUDY PARTICIPANT DETAILS
- METHOD DETAILS
  - Calculating genome assembly statistics
  - Analysis of WGS data
  - Sample quality control
  - Identification of genomic regions with potential mis-assembly in mRatBN7.2
  - Evaluating Liftover
  - Identify live strains that are close to HS progenitors
  - Constructing a genetic map using genetic data from a large HS cohort
  - Phylogenetic tree
  - RNA-seq data
  - eQTL relocation analysis
  - Capped small (cs)RNA-seq data
  - Single nuclei (sn) RNA-seq data
  - Transcriptome termini site sequencing
  - Brain proteome data
  - Identifying potentially mislabeled samples
  - Ensembl annotation
  - RefSeq annotation

SUPPLEMENTAL INFORMATION

Supplemental information can be found online at https://doi.org/10.1016/j.xgen.2024.100527.

ACKNOWLEDGMENTS

This work is supported by the Academy of Finland (grant no. 343656) to P.R.; NIH NHLBI R01HL064541 and P01HL149620 and Office of the Director

R24OD024617 to M.T.; NIH NIDA U01DA043098 to H.A.; NIH NIDA U01DA051972 to C.B.; NIH NHLBI R01HL064541 and Office of the Director R24OD024617 to W.M.D.; NIH NHLBI P01HL149620 and Office of the Director R24OD024617 to A.M.G.; Wellcome Trust WT222155/Z/20/Z to T.H.; NIH R01HG011252 to T.K.; Wellcome Trust WT222155/Z/20/Z to F.J.M.; NIH R01GM140287 to P.M.; a program from the National Institute for Research of Metabolic and Cardiovascular Diseases (Program EXCELES, ID project no. LX22NPO5104) funded by the European Union – Next Generation EU to M.P.; National Institute of Food and Agriculture, United States Department of Agriculture (2016-67015-24470/2020-67015-31733/2022-51300-38058/2023-67015-39566/2023-67015-40080) to Z.J.; NIH NIDA U01DA051234 to J.S.; NIH NHLBI R01HL064541, NHGRI U24HG010859, and Office of the Director R24OD024617 to J.R.S.; NIH NIDA P50DA037844 to L.C.S.W. (HS rats); NIH NIAAA R24AA013162 to B.T.; NIH NIDA U01DA050239 and U01DA051972 to F.T.; NIH NIDA P30DA044223 to L.S.; the National Center for Biotechnology Information of the National Library of Medicine (NLM), National Institutes of Health to T.D.M.; NIH NHLBI R01HL064541 and P01HL149620, NHGRI U24HG010859, and Office of the Director R24OD024617 to A.E.K.; NIH Office of the Director grant R24OD024617 to M.R.D. (HRDP); NIH NIDA U01DA047638 and P30DA044223 to R.W.W.; NIH NIDA U01DA043098 to J.Z.L.; and NIH NIDA U01DA047638, P50DA037844, and R01DA048017 to H.C. The majority of the computation for this work was performed on the University of Tennessee Infrastructure for Scientific Applications and Advanced Computing (ISAAC) computational resources.

This work is dedicated to the memory of Dr. Mary Shimoyama.

AUTHOR CONTRIBUTIONS

Conceptualization, A.A.P., R.W.W., J.Z.L., and H.C.; formal analysis, T.V.d.J., Y.P., P.R., D.M., M.T., X.W., T.D.M., J.Z.L., and H.C.; visualization, T.V.d.J., Y.P., P.R., D.M., F.T., Z.J., L.S., X.W., and J.Z.L.; investigation, H.A., C.B., D.C., A.S.C., W.C., V.C., W.M.D., P.A.D., E.G., A.M.G., H.M.G., V.G., T.H., K.H., J.H., T.K., P.K., L.L., S.M., F.J.M., P.M., A.B.O., O.P., P.P., J.S., J.R.S., L.C.S.W., B.T., A.T., M.U.-S., F.V., H.W., F.T., Z.J., and L.S.; resources, C.L.D., M.P., A.E.K., and M.R.D.; data curation, W.M.D., A.M.G., J.R.S., A.E.K., and M.R.D.; writing – original draft, T.V.d.J., Y.P., P.R., D.M., T.H., L.S., X.W., T.D.M., J.Z.L., and H.C.; writing – review & editing, A.M.G., B.M.S., L.S., X.W., T.D.M., A.A.P., A.E.K., M.R.D., R.W.W., J.Z.L., and H.C.; supervision, J.Z.L. and H.C.

DECLARATION OF INTERESTS

The authors declare no competing interests.

Received: October 2, 2023
Revised: December 26, 2023
Accepted: February 29, 2024
Published: March 26, 2024

REFERENCES

1. Parker, C.C., Chen, H., Flagel, S.B., Geurts, A.M., Richards, J.B., Robinson, T.E., Solberg Woods, L.C., and Palmer, A.A. (2014). Rats are the smart choice: Rationale for a renewed focus on rats in behavioral genetics. Neuropharmacology *76*, 250–258.
2. Richter, C.P. (1954). The effects of domestication and selection on the behavior of the Norway rat. J. Natl. Cancer Inst. *15*, 727–738.
3. Hulme-Beaman, A., Orton, D., and Cucchi, T. (2021). The origins of the domesticate brown rat (Rattus norvegicus) and its pathways to domestication. Anim. Front. *11*, 78–86.
4. Modlinska, K., and Pisula, W. (2020). The Norway rat, from an obnoxious pest to a laboratory pet. Elife *9*, e50651. https://doi.org/10.7554/eLife.50651.
5. Smith, J.R., Hayman, G.T., Wang, S.-J., Laulederkind, S.J.F., Hoffman, M.J., Kaldunski, M.L., Tutaj, M., Thota, J., Nalabolu, H.S., Ellanki, S.L.R., et al. (2020). The Year of the Rat: The Rat Genome Database at 20: a multi-species knowledgebase and analysis platform. Nucleic Acids Res. *48*, D731–D742.
6. RRRC (2021). Rat Resource & Research Center - Rat Models. https://www.rrrc.us/.
7. Pravenec, M., Klír, P., Kren, V., Zicha, J., and Kunes, J. (1989). An analysis of spontaneous hypertension in spontaneously hypertensive rats by means of new recombinant inbred strains. J. Hypertens. *7*, 217–221.
8. Voigt, B., Kuramoto, T., Mashimo, T., Tsurumi, T., Sasaki, Y., Hokao, R., and Serikawa, T. (2008). Evaluation of LEXF/FXLE rat recombinant inbred strains for genetic dissection of complex traits. Physiol. Genomics *32*, 335–342.
9. Tabakoff, B., Smith, H., Vanderlinden, L.A., Hoffman, P.L., and Saba, L.M. (2019). Networking in Biology: The Hybrid Rat Diversity Panel. In Rat Genomics, G. Hayman, J. Smith, M. Dwinell, and M. Shimoyama, eds. (Humana), pp. 213–231.
10. Hansen, C., and Spuhler, K. (1984). Development of the National Institutes of Health genetically heterogeneous rat stock. Alcohol Clin. Exp. Res. *8*, 477–479.
11. Rat Genome Sequencing and Mapping Consortium (2013). Combined sequence-based and genetic mapping analysis of complex traits in outbred rats. Nat. Genet. *45*, 767–775.
12. Woods, L.C.S., and Mott, R. (2017). Heterogeneous Stock Populations for Analysis of Complex Traits. In Systems Genetics, K. Schughart and R. Williams, eds. (Humana Press), pp. 31–44.
13. Solberg Woods, L.C., and Palmer, A.A. (2019). Using Heterogeneous Stocks for Fine-Mapping Genetically Complex Traits. In Rat Genomics, G. Hayman, J. Smith, M. Dwinell, and M. Shimoyama, eds. (Humana), pp. 233–247.
14. Chitre, A.S., Polesskaya, O., Holl, K., Gao, J., Cheng, R., Bimschleger, H., Garcia Martinez, A., George, T., Gileta, A.F., Han, W., et al. (2020). Genome-Wide Association Study in 3,173 Outbred Rats Identifies Multiple Loci for Body Weight, Adiposity, and Fasting Glucose. Obesity *28*, 1964–1973.
15. Gunturkun, M.H., Wang, T., Chitre, A.S., Garcia Martinez, A., Holl, K., St Pierre, C., Bimschleger, H., Gao, J., Cheng, R., Polesskaya, O., et al. (2022). Genome-Wide Association Study on Three Behaviors Tested in an Open Field in Heterogeneous Stock Rats Identifies Multiple Loci Implicated in Psychiatric Disorders. Front. Psychiatry *13*, 790566.
16. Kaldunski, M.L., Smith, J.R., Hayman, G.T., Brodie, K., De Pons, J.L., Demos, W.M., Gibson, A.C., Hill, M.L., Hoffman, M.J., Lamers, L., et al. (2022). The Rat Genome Database (RGD) facilitates genomic and phenotypic data integration across multiple species for biomedical research. Mamm. Genome *33*, 66–80.
17. Gibbs, R.A., Weinstock, G.M., Metzker, M.L., Muzny, D.M., Sodergren, E.J., Scherer, S., Scott, G., Steffen, D., Worley, K.C., Burch, P.E., et al. (2004). Genome sequence of the Brown Norway rat yields insights into mammalian evolution. Nature *428*, 493–521.
18. Worley, K.C., Weinstock, G.M., and Gibbs, R.A. (2008). Rats in the genomic era. Physiol. Genomics *32*, 273–282.
19. Twigger, S.N., Pruitt, K.D., Fernández-Suárez, X.M., Karolchik, D., Worley, K.C., Maglott, D.R., Brown, G., Weinstock, G., Gibbs, R.A., Kent, J., et al. (2008). What everybody should know about the rat genome and its online resources. Nat. Genet. *40*, 523–527.
20. van Heesch, S., Kloosterman, W.P., Lansu, N., Ruzius, F.-P., Levandowsky, E., Lee, C.C., Zhou, S., Goldstein, S., Schwartz, D.C., Harkins, T.T., et al. (2013). Improving mammalian genome scaffolding using large insert mate-pair next-generation sequencing. BMC Genom. *14*, 257.
21. Tutaj, M., Smith, J.R., and Bolton, E.R. (2019). Rat Genome Assemblies, Annotation, and Variant Repository. In Rat Genomics, G. Hayman, J. Smith, M. Dwinell, and M. Shimoyama, eds. (Humana), pp. 43–70.
22. Ramdas, S., Ozel, A.B., Treutelaar, M.K., Holl, K., Mandel, M., Woods, L.C.S., and Li, J.Z. (2019). Extended regions of suspected mis-assembly in the rat reference genome. Sci. Data *6*, 39.

23. Howe, K., Dwinell, M., Shimoyama, M., Corton, C., Betteridge, E., Dove, A., Quail, M.A., Smith, M., Saba, L., Williams, R.W., et al. (2021). The genome sequence of the Norway rat, *Rattus norvegicus* Berkenhout 1769. Wellcome Open Res. *6*, 118.

24. Howe, K., Chow, W., Collins, J., Pelan, S., Pointon, D.-L., Sims, Y., Torrance, J., Tracey, A., and Wood, J. (2021). Significantly improving the quality of genome assemblies through curation. GigaScience *10*, giaa153. https://doi.org/10.1093/gigascience/giaa153.

25. Mikheenko, A., Prjibelski, A., Saveliev, V., Antipov, D., and Gurevich, A. (2018). Versatile genome assembly evaluation with QUAST-LG. Bioinformatics *34*, i142–i150.

26. Poplin, R., Chang, P.-C., Alexander, D., Schwartz, S., Colthurst, T., Ku, A., Newburger, D., Dijamco, J., Nguyen, N., Afshar, P.T., et al. (2018). A universal SNP and small-indel variant caller using deep neural networks. Nat. Biotechnol. *36*, 983–987. https://doi.org/10.1038/nbt.4235.

27. Yun, T., Li, H., Chang, P.-C., Lin, M.F., Carroll, A., and McLean, C.Y. (2021). Accurate, scalable cohort variant calls using DeepVariant and GLnexus. Bioinformatics *36*, 5582–5589. https://doi.org/10.1093/bioinformatics/btaa1081.

28. Manni, M., Berkeley, M.R., Seppey, M., Simão, F.A., and Zdobnov, E.M. (2021). BUSCO Update: Novel and Streamlined Workflows along with Broader and Deeper Phylogenetic Coverage for Scoring of Eukaryotic, Prokaryotic, and Viral Genomes. Mol. Biol. Evol. *38*, 4647–4654.

29. Zhou, X., Li, R., Michal, J.J., Wu, X.-L., Liu, Z., Zhao, H., Xia, Y., Du, W., Wildung, M.R., Pouchnik, D.J., et al. (2016). Accurate Profiling of Gene Expression and Alternative Polyadenylation with Whole Transcriptome Termini Site Sequencing (WTTS-Seq). Genetics *203*, 683–697.

30. Munro, D., Wang, T., Chitre, A.S., Polesskaya, O., Ehsan, N., Gao, J., Gusev, A., Woods, L.C.S., Saba, L.M., Chen, H., et al. (2022). The regulatory landscape of multiple brain regions in outbred heterogeneous stock rats. Nucleic Acids Res. *50*, 10882–10895. https://doi.org/10.1093/nar/gkac912.

31. Martín-Gálvez, D., Dunoyer de Segonzac, D., Ma, M.C.J., Kwitek, A.E., Thybert, D., and Flicek, P. (2017). Genome variation and conserved regulation identify genomic regions responsible for strain specific phenotypes in rat. BMC Genom. *18*, 986.

32. Ma, M.C.J., Atanur, S.S., Aitman, T.J., and Kwitek, A.E. (2014). Genomic structure of nucleotide diversity among Lyon rat models of metabolic syndrome. BMC Genom. *15*, 197.

33. Buniello, A., MacArthur, J.A.L., Cerezo, M., Harris, L.W., Hayhurst, J., Malangone, C., McMahon, A., Morales, J., Mountjoy, E., Sollis, E., et al. (2019). The NHGRI-EBI GWAS Catalog of published genome-wide association studies, targeted arrays and summary statistics 2019. Nucleic Acids Res. *47*, D1005–D1012.

34. Koren, S., Phillippy, A.M., Simpson, J.T., Loman, N.J., and Loose, M. (2019). Reply to "Errors in long-read assemblies can critically affect protein prediction.". Nat. Biotechnol. *37*, 127–128.

35. Watson, M., and Warr, A. (2019). Errors in long-read assemblies can critically affect protein prediction. Nat. Biotechnol. *37*, 124–126.

36. Sacristán-Horcajada, E., González-de la Fuente, S., Peiró-Pastor, R., Carrasco-Ramiro, F., Amils, R., Requena, J.M., Berenguer, J., and Aguado, B. (2021). ARAMIS: From systematic errors of NGS long reads to accurate assemblies. Brief. Bioinform. *22*, bbab170. https://doi.org/10.1093/bib/bbab170.

37. Rhie, A., McCarthy, S.A., Fedrigo, O., Damas, J., Formenti, G., Koren, S., Uliano-Silva, M., Chow, W., Fungtammasan, A., Kim, J., et al. (2021). Towards complete and error-free genome assemblies of all vertebrate species. Nature *592*, 737–746.

38. Chin, C.-S., Peluso, P., Sedlazeck, F.J., Nattestad, M., Concepcion, G.T., Clum, A., Dunn, C., O'Malley, R., Figueroa-Balderas, R., Morales-Cruz, A., et al. (2016). Phased diploid genome assembly with single-molecule real-time sequencing. Nat. Methods *13*, 1050–1054.

39. de Jong, T.V., Chen, H., Brashear, W.A., Kochan, K.J., Hillhouse, A.E., Zhu, Y., Dhande, I.S., Hudson, E.A., Sumlut, M.H., Smith, M.L., et al. (2022). mRatBN7.2: familiar and unfamiliar features of a new rat genome reference assembly. Physiol. Genomics *54*, 251–260.

40. Nurk, S., Koren, S., Rhie, A., Rautiainen, M., Bzikadze, A.V., Mikheenko, A., Vollger, M.R., Altemose, N., Uralsky, L., Gershman, A., et al. (2022). The complete sequence of a human genome. Science *376*, 44–53.

41. Atanur, S.S., Diaz, A.G., Maratou, K., Sarkis, A., Rotival, M., Game, L., Tschannen, M.R., Kaisaki, P.J., Otto, G.W., Ma, M.C.J., et al. (2013). Genome sequencing reveals loci under artificial selection that underlie disease phenotypes in the laboratory rat. Cell *154*, 691–703.

42. Hermsen, R., de Ligt, J., Spee, W., Blokzijl, F., Schäfer, S., Adami, E., Boymans, S., Flink, S., van Boxtel, R., van der Weide, R.H., et al. (2015). Genomic landscape of rat strain and substrain variation. BMC Genom. *16*, 357.

43. Supernat, A., Vidarsson, O.V., Steen, V.M., and Stokowy, T. (2018). Comparison of three variant callers for human whole genome sequencing. Sci. Rep. *8*, 17851.

44. Ashbrook, D.G., Arends, D., Prins, P., Mulligan, M.K., Roy, S., Williams, E.G., Lutz, C.M., Valenzuela, A., Bohl, C.J., Ingels, J.F., et al. (2021). A platform for experimental precision medicine: The extended BXD mouse family. Cell Syst. *12*, 235–247.e9.

45. Ashbrook, D.G., Sasani, T., Maksimov, M., Gunturkun, M.H., Ma, N., Villani, F., Ren, Y., Rothschild, D., Chen, H., Lu, L., et al. (2022). Private and sub-family specific mutations of founder haplotypes in the BXD family reveal phenotypic consequences relevant to health and disease. Preprint at bioRxiv. https://doi.org/10.1101/2022.04.21.489063.

46. Pattee, J., Vanderlinden, L.A., Mahaffey, S., Hoffman, P., Tabakoff, B., and Saba, L.M. (2022). Evaluation and characterization of expression quantitative trait analysis methods in the Hybrid Rat Diversity Panel. Front. Genet. *13*, 947423.

47. Senko, A.N., Overall, R.W., Silhavy, J., Mlejnek, P., Malínská, H., Hüttl, M., Marková, I., Fabel, K.S., Lu, L., Stuchlik, A., et al. (2022). Systems genetics in the rat HXB/BXH family identifies Tti2 as a pleiotropic quantitative trait gene for adult hippocampal neurogenesis and serum glucose. PLoS Genet. *18*, e1009638.

48. Bryant, C.D., Smith, D.J., Kantak, K.M., Nowak, T.S., Jr., Williams, R.W., Damaj, M.I., Redei, E.E., Chen, H., and Mulligan, M.K. (2020). Facilitating Complex Trait Analysis via Reduced Complexity Crosses. Trends Genet. *36*, 549–562.

49. Witte, F., Ruiz-Orera, J., Mattioli, C.C., Blachut, S., Adami, E., Schulz, J.F., Schneider-Lunitz, V., Hummel, O., Patone, G., Mücke, M.B., et al. (2021). A trans locus causes a ribosomopathy in hypertrophic hearts that affects mRNA translation in a protein length-dependent fashion. Genome Biol. *22*, 191.

50. Pravenec, M., Kožich, V., Krijt, J., Sokolová, J., Zídek, V., Landa, V., Mlejnek, P., Šilhavý, J., Šimáková, M., Škop, V., et al. (2016). Genetic Variation in Renal Expression of Folate Receptor 1 (Folr1) Gene Predisposes Spontaneously Hypertensive Rats to Metabolic Syndrome. Hypertension *67*, 335–341.

51. Pravenec, M., Churchill, P.C., Churchill, M.C., Viklicky, O., Kazdova, L., Aitman, T.J., Petretto, E., Hubner, N., Wallace, C.A., Zimdahl, H., et al. (2008). Identification of renal Cd36 as a determinant of blood pressure and risk for hypertension. Nat. Genet. *40*, 952–954.

52. Heinig, M., Petretto, E., Wallace, C., Bottolo, L., Rotival, M., Lu, H., Li, Y., Sarwar, R., Langley, S.R., Bauerfeind, A., et al. (2010). A trans-acting locus regulates an anti-viral expression network and type 1 diabetes risk. Nature *467*, 460–464.

53. Lu, L.M., Shisa, H., Tanuma, J., and Hiai, H. (1999). Propylnitrosourea-induced T-lymphomas in LEXF RI strains of rats: genetic analysis. Br. J. Cancer *80*, 855–861.

54. Hubner, N., Wallace, C.A., Zimdahl, H., Petretto, E., Schulz, H., Maciver, F., Mueller, M., Hummel, O., Monti, J., Zidek, V., et al. (2005). Integrated

transcriptional profiling and linkage analysis for identification of genes underlying disease. Nat. Genet. *37*, 243–253.

55. Holl, K., He, H., Wedemeyer, M., Clopton, L., Wert, S., Meckes, J.K., Cheng, R., Kastner, A., Palmer, A.A., Redei, E.E., and Solberg Woods, L.C. (2018). Heterogeneous stock rats: a model to study the genetics of despair-like behavior in adolescence. Genes Brain Behav. *17*, 139–148.

56. Keele, G.R., Prokop, J.W., He, H., Holl, K., Littrell, J., Deal, A., Francic, S., Cui, L., Gatti, D.M., Broman, K.W., et al. (2018). Genetic Fine-Mapping and Identification of Candidate Genes and Variants for Adiposity Traits in Outbred Rats. Obesity *26*, 213–222.

57. Solberg Woods, L.C., Holl, K.L., Oreper, D., Xie, Y., Tsaih, S.-W., and Valdar, W. (2012). Fine-mapping diabetes-related traits, including insulin resistance, in heterogeneous stock rats. Physiol. Genomics *44*, 1013–1026.

58. Lemen, P.M., Hatoum, A.S., Dickson, P.E., Mittleman, G., Agrawal, A., Reiner, B.C., Berrettini, W., Ashbrook, D., Gunturkun, H., Mulligan, M.K., et al. (2022). Opiate responses are controlled by interactions of *Oprm1* and *Fgf12* loci in the murine BXD family: Correspondence to human GWAS finding. Preprint at bioRxiv. https://doi.org/10.1101/2022.03.11.483993.

59. Lusis, A.J., Seldin, M.M., Allayee, H., Bennett, B.J., Civelek, M., Davis, R.C., Eskin, E., Farber, C.R., Hui, S., Mehrabian, M., et al. (2016). The Hybrid Mouse Diversity Panel: a resource for systems genetics analyses of metabolic and cardiovascular traits. J. Lipid Res. *57*, 925–942.

60. Ulusoy, S., Ozkan, G., Alkanat, M., Mungan, S., Yuluğ, E., and Orem, A. (2013). Perspective on rhabdomyolysis-induced acute kidney injury and new treatment options. Am. J. Nephrol. *38*, 368–378.

61. Kang, K., Nan, C., Fei, D., Meng, X., Liu, W., Zhang, W., Jiang, L., Zhao, M., Pan, S., and Zhao, M. (2013). Heme oxygenase 1 modulates thrombomodulin and endothelial protein C receptor levels to attenuate septic kidney injury. Shock *40*, 136–143.

62. Yuan, G., Deng, J., Wang, T., Zhao, C., Xu, X., Wang, P., Voltz, J.W., Edin, M.L., Xiao, X., Chao, L., et al. (2007). Tissue kallikrein reverses insulin resistance and attenuates nephropathy in diabetic rats by activation of phosphatidylinositol 3-kinase/protein kinase B and adenosine 5'-monophosphate-activated protein kinase signaling pathways. Endocrinology *148*, 2016–2026.

63. Osipova, E., Barsacchi, R., Brown, T., Sadanandan, K., Gaede, A.H., Monte, A., Jarrells, J., Moebius, C., Pippel, M., Altshuler, D.L., et al. (2023). Loss of a gluconeogenic muscle enzyme contributed to adaptive metabolic traits in hummingbirds. Science *379*, 185–190.

64. Obeidat, M. 'en, Zhou, G., Li, X., Hansel, N.N., Rafaels, N., Mathias, R., Ruczinski, I., Beaty, T.H., Barnes, K.C., Paré, P.D., and Sin, D.D. (2018). The genetics of smoking in individuals with chronic obstructive pulmonary disease. Respir. Res. *19*, 59.

65. Canzian, F. (1997). Phylogenetics of the laboratory rat Rattus norvegicus. Genome Res. *7*, 262–267.

66. Mashimo, T., Voigt, B., Tsurumi, T., Naoi, K., Nakanishi, S., Yamasaki, K.-I., Kuramoto, T., and Serikawa, T. (2006). A set of highly informative rat simple sequence length polymorphism (SSLP) markers and genetically defined rat strains. BMC Genet. *7*, 19.

67. Chen, N.-C., Solomon, B., Mun, T., Iyer, S., and Langmead, B. (2021). Reference flow: reducing reference bias using multiple population genomes. Genome Biol. *22*, 8.

68. Munger, S.C., Raghupathy, N., Choi, K., Simons, A.K., Gatti, D.M., Hinerfeld, D.A., Svenson, K.L., Keller, M.P., Attie, A.D., Hibbs, M.A., et al. (2014). RNA-Seq alignment to individualized genomes improves transcript abundance estimates in multiparent populations. Genetics *198*, 59–73.

69. Eizenga, J.M., Novak, A.M., Sibbesen, J.A., Heumos, S., Ghaffaari, A., Hickey, G., Chang, X., Seaman, J.D., Rounthwaite, R., Ebler, J., et al. (2020). Pangenome Graphs. Annu. Rev. Genomics Hum. Genet. *21*, 139–162.

70. Villani, F., Guarracino, A., Ward, R.R., Green, T., Emms, M., Pravenec, M., Prins, P., Garrison, E., Williams, R.W., Chen, H., and Colonna, V. (2024). Pangenome reconstruction in rats enhances genotype-phenotype mapping and novel variant discovery. Preprint at bioRxiv. https://doi.org/10.1101/2024.01.10.575041.

71. Kalbfleisch, T.S., Hussien AbouEl Ela, N.A., Li, K., Brashear, W.A., Kochan, K.J., Hillhouse, A.E., Zhu, Y., Dhande, I.S., Kline, E.J., Hudson, E.A., et al. (2023). The Assembled Genome of the Stroke-Prone Spontaneously Hypertensive Rat. Hypertension *80*, 138–146.

72. Rastas, P. (2020). Lep-Anchor: automated construction of linkage map anchored haploid genomes. Bioinformatics *36*, 2359–2364.

73. Kivikoski, M., Rastas, P., Löytynoja, A., and Merilä, J. (2021). Automated improvement of stickleback reference genome assemblies with Lep-Anchor software. Mol. Ecol. Resour. *21*, 2166–2176.

74. ENCODE Project Consortium (2012). An integrated encyclopedia of DNA elements in the human genome. Nature *489*, 57–74.

75. Duttke, S.H., Montilla-Perez, P., Chang, M.W., Li, H., Chen, H., Carrette, L.L.G., de Guglielmo, G., George, O., Palmer, A.A., Benner, C., and Telese, F. (2022). Glucocorticoid Receptor-Regulated Enhancers Play a Central Role in the Gene Regulatory Networks Underlying Drug Addiction. Front. Neurosci. *16*, 858427.

76. O'Leary, N.A., Wright, M.W., Brister, J.R., Ciufo, S., Haddad, D., McVeigh, R., Rajput, B., Robbertse, B., Smith-White, B., Ako-Adjei, D., et al. (2016). Reference sequence (RefSeq) database at NCBI: current status, taxonomic expansion, and functional annotation. Nucleic Acids Res. *44*, D733–D745.

77. Kalikar, S., Jain, C., Vasimuddin, M., and Misra, S. (2022). Accelerating minimap2 for long-read sequencing applications on modern CPUs. Nat. Comput. Sci. *2*, 78–83.

78. Li, H., and Durbin, R. (2010). Fast and accurate long-read alignment with Burrows-Wheeler transform. Bioinformatics *26*, 589–595.

79. Poplin, R., Ruano-Rubio, V., DePristo, M.A., Fennell, T.J., Carneiro, M.O., Van der Auwera, G.A., Kling, D.E., Gauthier, L.D., Levy-Moonshine, A., Roazen, D., et al. (2018). Scaling accurate genetic variant discovery to tens of thousands of samples. Preprint at bioRxiv. https://doi.org/10.1101/201178.

80. Li, H. (2018). Minimap2: pairwise alignment for nucleotide sequences. Bioinformatics *34*, 3094–3100.

81. Jeffares, D.C., Jolly, C., Hoti, M., Speed, D., Shaw, L., Rallis, C., Balloux, F., Dessimoz, C., Bähler, J., and Sedlazeck, F.J. (2017). Transient structural variations have strong effects on quantitative traits and reproductive isolation in fission yeast. Nat. Commun. *8*, 14061.

82. Cingolani, P., Platts, A., Wang, L.L., Coon, M., Nguyen, T., Wang, L., Land, S.J., Lu, X., and Ruden, D.M. (2012). A program for annotating and predicting the effects of single nucleotide polymorphisms, SnpEff: SNPs in the genome of Drosophila melanogaster strain w1118; iso-2; iso-3. Fly *6*, 80–92.

83. Tarailo-Graovac, M., and Chen, N. (2009). Using RepeatMasker to identify repetitive elements in genomic sequences. Curr. Protoc. Bioinformatics *25*, 4.10.1–4.10.14.

84. Gunturkun, M.H., Flashner, E., Wang, T., Mulligan, M.K., Williams, R.W., Prins, P., and Chen, H. (2022). GeneCup: mining PubMed and GWAS catalog for gene–keyword relationships. G3 (Bethesda). *12*, jkac059.

85. Hinrichs, A.S., Karolchik, D., Baertsch, R., Barber, G.P., Bejerano, G., Clawson, H., Diekhans, M., Furey, T.S., Harte, R.A., Hsu, F., et al. (2006). The UCSC Genome Browser Database: update 2006. Nucleic Acids Res. *34*, D590–D598.

86. Purcell, S., Neale, B., Todd-Brown, K., Thomas, L., Ferreira, M.A.R., Bender, D., Maller, J., Sklar, P., de Bakker, P.I.W., Daly, M.J., and Sham, P.C. (2007). PLINK: a tool set for whole-genome association

and population-based linkage analyses. Am. J. Hum. Genet. *81*, 559–575.

87. Rastas, P. (2017). Lep-MAP3: robust linkage mapping even for low-coverage whole genome sequencing data. Bioinformatics *33*, 3726–3732.
88. Danecek, P., Bonfield, J.K., Liddle, J., Marshall, J., Ohan, V., Pollard, M.O., Whitwham, A., Keane, T., McCarthy, S.A., Davies, R.M., et al. (2021). Twelve years of SAMtools and BCFtools. GigaScience *10*, giab008. https://doi.org/10.1093/gigascience/giab008.
89. Tamura, K., Stecher, G., and Kumar, S. (2021). MEGA11: Molecular Evolutionary Genetics Analysis Version 11. Mol. Biol. Evol. *38*, 3022–3027.
90. Yu, G., Smith, D.K., Zhu, H., Guan, Y., and Lam, T.T.-Y. (2017). Ggtree : An r package for visualization and annotation of phylogenetic trees with their covariates and other associated data. Methods Ecol. Evol. *8*, 28–36.
91. Dobin, A., Davis, C.A., Schlesinger, F., Drenkow, J., Zaleski, C., Jha, S., Batut, P., Chaisson, M., and Gingeras, T.R. (2013). STAR: ultrafast universal RNA-seq aligner. Bioinformatics *29*, 15–21.
92. Duttke, S.H., Chang, M.W., Heinz, S., and Benner, C. (2019). Identification and dynamic quantification of regulatory elements using total RNA. Genome Res. *29*, 1836–1846.
93. Delaneau, O., Ongen, H., Brown, A.A., Fort, A., Panousis, N.I., and Dermitzakis, E.T. (2017). A complete tool set for molecular QTL discovery and analysis. Nat. Commun. *8*, 15452.
94. UniProt Consortium (2019). UniProt: a worldwide hub of protein knowledge. Nucleic Acids Res. *47*, D506–D515.
95. Gileta, A.F., Gao, J., Chitre, A.S., Bimschleger, H.V., St Pierre, C.L., Gopalakrishnan, S., and Palmer, A.A. (2020). Adapting Genotyping-by-Sequencing and Variant Calling for Heterogeneous Stock Rats. G3 (Bethesda) *10*, 2195–2205.
96. Zhou, J.L., de Guglielmo, G., Ho, A.J., Kallupi, M., Li, H.-R., Chitre, A.S., Carrette, L.L.G., George, O., Palmer, A.A., McVicker, G., and Telese, F. (2022). Cocaine addiction-like behaviors are associated with long-term changes in gene regulation, energy metabolism, and GABAergic inhibition within the amygdala. Preprint at bioRxiv. https://doi.org/10.1101/2022.09.08.506493.
97. Jeffs, B., Negrin, C.D., Graham, D., Clark, J.S., Anderson, N.H., Gauguier, D., and Dominiczak, A.F. (2000). Applicability of a "speed" congenic strategy to dissect blood pressure quantitative trait loci on rat chromosome 2. Hypertension *35*, 179–187.
98. Aken, B.L., Ayling, S., Barrell, D., Clarke, L., Curwen, V., Fairley, S., Fernandez Banet, J., Billis, K., García Girón, C., Hourlier, T., et al. (2016). The Ensembl Gene Annotation System. Database *2016*, baw093. https://doi.org/10.1093/database/baw093.
99. Altschul, S.F., Gish, W., Miller, W., Myers, E.W., and Lipman, D.J. (1990). Basic local alignment search tool. J. Mol. Biol. *215*, 403–410.
100. Kozomara, A., Birgaoanu, M., and Griffiths-Jones, S. (2019). miRBase: from microRNA sequences to function. Nucleic Acids Res. *47*, D155–D162.
101. Gruber, A.R., Lorenz, R., Bernhart, S.H., Neuböck, R., and Hofacker, I.L. (2008). The Vienna RNA Websuite. Nucleic Acids Res. *36*, W70–W74.
102. Kalvari, I., Argasinska, J., Quinones-Olvera, N., Nawrocki, E.P., Rivas, E., Eddy, S.R., Bateman, A., Finn, R.D., and Petrov, A.I. (2018). Rfam 13.0: shifting to a genome-centric resource for non-coding RNA families. Nucleic Acids Res. *46*, D335–D342.
103. Nawrocki, E.P., and Eddy, S.R. (2013). Infernal 1.1: 100-fold faster RNA homology searches. Bioinformatics *29*, 2933–2935.
104. McGarvey, K.M., Goldfarb, T., Cox, E., Farrell, C.M., Gupta, T., Joardar, V.S., Kodali, V.K., Murphy, M.R., O'Leary, N.A., Pujar, S., et al. (2015). Mouse genome annotation by the RefSeq project. Mamm. Genome *26*, 379–390.

STAR★METHODS

KEY RESOURCES TABLE

REAGENT or RESOURCE	SOURCE	IDENTIFIER
Deposited data		
Structural variants in HXB RI panel (VCF)	This paper	https://doi.org/10.5281/zenodo.10398554
Join calling of 163 WGS rat samples (VCF)	This paper	https://doi.org/10.5281/zenodo.10398344
Transcription start site for 2 brain regions	This paper	https://doi.org/10.5281/zenodo.10398387
Alternative polyadenylation sites	This paper	https://doi.org/10.5281/zenodo.10398476
Potential misassembled regions	This paper	https://doi.org/10.5281/zenodo.10428255
Chromosomal dot plots between Rnor_6.0 and mRatBN7.2	This paper	https://doi.org/10.5281/zenodo.10515796
The order of genetic markers and the distances from a rat genetic map compared to their locations in Rnor_6.0	This paper	https://doi.org/10.5281/zenodo.10552387
The order of genetic markers and the distances from a rat genetic map compared to their locations in mRatBN7.2	This paper	https://doi.org/10.5281/zenodo.10552453
Code and data for phylogenetic analysis	This paper	https://doi.org/10.5281/zenodo.10520242
nucleus accumbens core eQTL dataset	Munro et al.[30]	NAcc, 75 samples
csRNA-seq data	Duttke et al.[75]	N/A
Annotation of the mRatBN7.2 assembly	O'Leary et al.[76]	Release 108
Code and computational notebook	This paper	https://doi.org/10.5281/zenodo.10552887
Experimental models: Organisms/strains		
Sample metadata for RatCollection	This paper	https://doi.org/10.5281/zenodo.10552790
Software and algorithms		
QUAST	Mikheenko et al.[25]	N/A
paftools	Kalikar et al.[77]	N/A
BWA mem	Li and Durbin[78]	N/A
GATK	Poplin et al.[79]	N/A
LongRanger		N/A
Minimap2	Li[80]	N/A
Deepvariant	Poplin et al.[26]	N/A
GLNexus	Yun et al.[27]	N/A
SURVIVOR	Jeffares et al.[81]	N/A
SnpEff	Cingolani et al.[82]	N/A
RepeatMasker	Tarailo-Graovac and Chen[83]	N/A
GeneCup	Gunturkun et al.[84]	N/A
UCSC Liftover tool	Hinrichs et al.[85]	N/A
PLINK	Purcell et al.[86]	N/A
Lep-MAP3	Rastas[87]	N/A
bcftools	Danecek et al.[88]	N/A
MEGA	Tamura et al.[89]	N/A
ggtree	Yu et al.[90]	N/A
STAR aligner	Dobin et al.[91]	N/A
HOMER's findPeaks tool	Duttke et al.[92]	N/A
QTLtools	Delaneau et al.[93]	N/A
UniProt	UniProt Consortium[94]	N/A

CellPress
OPEN ACCESS

RESOURCE AVAILABILITY

Lead contact

Further information and requests for resources should be directed to and will be fulfilled by the lead contact, Hao Chen (hchen@uthsc.edu).

Materials availability

The Hybrid Rat Diversity Panel (HRDP) consists of 96 inbred strains, and includes 30 classic inbred strains and both of the large RI families discussed in the prior sections (BXH/HXB and LEXF/FXLE). The panel is being cryo-resuscitated and cryopreserved at the Medical College of Wisconsin for use by the scientific community.

Data and code availability

- The RatCollection contains 163 WGS samples from 88 strains and 32 substrains. It includes new data from 128 rats (key resources table) and 36 datasets downloaded from NIH SRA. The detailed sample metadata are provided in the Table S8. A total of 82 members of the HRDP have been sequenced using Illumina short-read technology, including all 30 extant HXB strains, 23 of 27 FXLE/LEXF strains, and 25 of 30 classic inbred strains. RatCollection includes all 30 strains of the HXB/BXH family, 27 strains in the FXLE/LEXF family, and 33 other inbred strains. In total, we covered 88 strains and 32 substrains. It contains approximately 80% of the HRDP. WGS data generated for this work have been uploaded to NIH SRA (see Table S8 for SRA IDs). Other resources are available from Zenodo (See key resources table). The code for the custom R, Python and Bash scripts for data analysis is available from a github repository: https://github.com/hanyoupan/rat-manuscript (https://doi.org/10.5281/zenodo.10552887)
- Any additional information required to reanalyze the data reported in this paper is available from the lead contact upon request.

EXPERIMENTAL MODEL AND STUDY PARTICIPANT DETAILS

All rats used for whole genome sequencing are naive adult males. Their strain and substrain identification as well as RRIDs are provided in the Table S8.

METHOD DETAILS

Calculating genome assembly statistics

We obtained all assemblies from UCSC Goldenpath, with the exception of CHM13_T2T_v1.1, which was downloaded from the T2T Consortium GitHub page. We used QUAST[25] to calculate common assembly metrics, such as contig and scaffold N50, using a consistent standard across all assemblies. We defined each entry in the fasta file as a scaffold, breaking them into contigs based on continuous Ns of 10 or more. No scaffolds below a certain length were excluded from the analysis.[23] Our scripts and intermediate results can be found in the supplementary. Nx plots were generated using a custom Python script and the fasta files as inputs. Structural differences between Rnor_6.0 and mRatBN7.2 were evaluated using paftools.[77]

Analysis of WGS data

We used different methods for mapping data, based on the sequencing technologies. For illumina short read data, fastq files were mapped to the reference genome (either Rnor_6.0 or mRatBN7.2) using BWA mem.[78] GATK[79] was then used to mark PCR duplicates in the bam files. For 10x chromium linked reads, fastq files were mapped against the reference genomes using LongRanger (version 2.2.2). Long read data were mapped using Minimap2,[80] Deepvariant[26] (ver 1.0.0) was then used to call variants for each sample. Joint calling of variants for all the samples was conducted using GLNexus.[27] Large SVs detected by LongRanger were merged using SURVIVOR[81] per reference genome. SVs detected in less than two samples were removed. Variants with the QUAL score less than 30 were removed. The impact of variants were predicted using SnpEff,[82] using RefSeq annotations. Overlap between SNPs and repetitive regions were identified with RepeatMasker.[83] Disease ontology was retrieved from the Rat Genome Database.[16] Disease associations were related to nearest genes as predicted by SnpEff. Subsequent analyses were conducted using custom scripts in R or bash. Circular plots were generated with the Circos plots package in R. Functional consequences of variants on genes were searched in PubMed via GeneCup.[84]

Sample quality control

To ensure the quality of data from 168 rats, we conducted a thorough examination of the missing call rate and read depth per sample. We determined that a per sample missing rate of 4% and average read depth of 10 were appropriate based on the data distribution. We identified and removed 4 samples with high missing rates (SHR/NCrlPrin_BT.ILM, WKY/NHsd_TA.ILM, GK/Ox_TA.ILM, FHL/EurMcwi_TA.ILM) and 2 samples with low read depth (WKY/NHsd_TA.ILM, BBDP/Wor_TA.ILM), resulting in a total of 5 samples removed.

Identification of genomic regions with potential mis-assembly in mRatBN7.2

We used WGS-derived genotype data for 163 samples to detect genomic regions with unusually high densities of heterozygous genotypes (i.e., the "high-het" regions). Since the samples are from inbred animals, high-het regions could arise from tandemly repeated segments in the BN genome "folded" into a single region in mRatBN7.2. While read-depth data represent an independent source of information, here we focused on the distribution patterns of heterogeneous genotypes in the 12 BN animals. Indeed, along the genome, the per-site heterozygote counts, ranging 0 to 12 across the 12 BN samples, tend to be high in certain discrete regions, which also tend to have high counts of NA (the "no-calls"). We used the het+NA counts as the scanning statistics for segment-wise switches between a high-state and a low-state, akin to using arrayCGH intensity data or sequencing read depths to detect DNA copy-number variants (CNVs). Specifically, we added 2 to the per-site het+NA counts, to mimic the situation with 2 DNA copies in baseline regions and up to 14 copies in the high-regions. The logged values, ranging from log2(2) to log2(14), are "segmented" by using the segment command in R package "DNAcopy," with parameters min.width = 5,alpha = 0.001. The results tend to be over-segmented, and they are merged by a custom R script with the following merging rules. Starting from the first three segments, we merge the first and the second segments if one of the four conditions are met: (1) both have mean values above 1.5, which is an empirically derived threshold in our log2(x+2) scale, (2) both have mean values below 1.5, (3) the three segments are high-low-high but the middle segment is less than 5 kb, hence a "positive flicker," or (4) the three segments are low-high-low with the middle segment shorter than 5 kb: a "negative flicker." If the first two segments are merged, the next "triplet" to be evaluated are the merged 1–2, the previous #3 segment, and the newly called-up $4 segment. If no merging is executed, the scan moves by one segment to evaluate the next "triplet": #2–4. This operation was repeated until we reached the end of the chromosome. Altering the merging parameters will yield a somewhat different set of "flagged" regions. The current merged list contains 673 high-segments over the 20 autosomes, covering about 1.4% of mRatBN7.2 and having an average length of 52,199 nt. Future iterations will incorporate distribution patterns of read depth, multi-allelic sites and, if possible, linked read information. Similar scans can be performed for the 151 non-BN samples. Our preliminary data show that most of the regions flagged by the 12 BN samples also show high Het+NA counts in non-BN samples. In addition, some other heterozygous genotypes in non-BN samples fall outside the flagged regions, either individually or in new segments not flagged in BN samples. Many of them likely represent single-site or segment-wise differences between these strains and the reference strain: BN, and they may vary in a strain-specific fashion.

Evaluating Liftover

The Liftover tool is evaluated using both simulated and real data. The simulated data is an evenly 1000-base pair-spaced bed file covering Rnor_6.0 (2,782,023 sites within the bed file). The simulated dataset is used to study what portion of the genome is liftable, and the distribution of the unliftable and liftable sites. The real dataset is used to evaluate the accuracy and utility of the Liftover process, we compared the variants obtained through the Liftover process to those directly called from the data. We mapped WGS data from a WKY/N rat to both Rnor_6.0 and mRatBN7.2 and called variants against each respective genome. We then used the UCSC Liftover tool[85] and corresponding chain files to lift these variants to the other reference genome. The resulting lifted variant sets are then compared to the directly called variant sets.

Identify live strains that are close to HS progenitors

Currently, there are two colonies of the HS rats: one located at Wake Forest University (RRID: RGD_13673907), which was moved from the Medical College of Wisconsin (RRID: RGD_2314009); the University of California San Diego houses the other colony (RID: RGD_155269102). While the original progenitor population is no longer alive, we obtained DNA samples that were preserved in 1984, when the HS colony was created. Using the 163-by-163 IBS matrix previously generated (see method: tree generation). We identified six closely-related substrains of the progenitors that were over 99.5% similar to the original strains based on identity by state (IBS): ACI/EurMcwi, BN/NHsdMcwi, F344/DuCrl, M520/NRrrcMcwi, MR/NRrrc WKY/NHsd. The best matches of the remaining strains were less similar: BUF/Mna (73.6%) for BUF/N and WAG/RijCrl (72.0%) for WN/N. Better alternatives for these two strains may be identified in the future as more inbred rat strains are sequenced. At the time of this report, all 8 of these inbred strains are available from Hybrid Rat Diversity Program at the Medical College of Wisconsin (see https://rgd.mcw.edu/wg/hrdp_panel/for strain availability and contact details).

Constructing a genetic map using genetic data from a large HS cohort

The collection of genotypes from 1893 HS rats and 753 parents (378 families) was described previously.[15] Briefly, genotypes were determined using genotyping-by-sequencing.[95] This produced approximately 3.5 million SNP with an estimated error rate <1%. Variants for X- and Y chromosomes were not called. The genotype data used for this study can be accessed from the C-GORD database at https://doi.org/10.48810/P44W2 or through https://www.genenetwork.org. The genotype data were further cleaned to remove monomorphic SNPs. Genotypes with high Mendelian inheritance error rates (>2% error across the cohort) were identified by PLINK[86] and removed. We further used an unbiased selection procedure, which yielded a final list of 150,835 binned markers distributed across the genome. Mean distance between markers is 18.5 kb across the genome. We used Lep-MAP3 (LM3)[87] to construct the genetic map. The following LM3 functions were used: 1) the ParentCall2 function was used to call parental genotypes by taking into account the genotype information of grandparents, parents, and offspring; 2) the Filtering2 function was used to remove those markers with segregation distortion or those with missing data using the default setting of LM3; and 3) the OrderMarkers2 function

was used to compute cM distances (i.e., recombination rates) between all adjacent markers per chromosome. The resulting map had consistent marker order that supported the mRatBN7.2.

Phylogenetic tree

We used bcftools[88] to filter for high-quality, bi-allelic SNP sites from the VCF files for the 20 autosomes. We then employed PLINK[86] to calculate a pairwise identity-by-state (IBS) matrix using the 11.5 M variants. We imported the resulting matrix into R and converted it to a meg format as described in the MEGA manual. We then used MEGA[89] to construct a distance-based UPGMA tree using the meg file as input. We used MEGA's "flip subtree" function to adjust the position of a few internal nodes for improved visualization using the MEGA GUI. The modified tree was exported as a nwk file. We then imported this nwk file into R and used the ggtree[90] package to plot the phylogenetic tree. There is a pdf file document the detailed steps of phylogenetics analysis available in key resources table "Code and data for phylogenetic analysis".

RNA-seq data

RNA-seq data was downloaded from RatGTEx.[30] Brains were extracted from 88 HS rats. Rats were housed under standard laboratory conditions. Rat brains were extracted and cryosectioned into 60 μm sections, which were mounted onto RNase-free glass slides. Slides were stored in −80°C until dissection and before RNA-extraction post dissection. AllPrep DNA/RNA mini kit (Qiagen) was used to extract RNA. RNA-seq was performed on mRNA from each brain region using Illumina HiSeq 4000 to obtain 100 bp single-end reads for 435 samples. RNA-Seq reads were aligned to the Rnor_6.0 and mRatBN7.2 genomes from Ensembl using STAR v2.7.8a.[91]

eQTL relocation analysis

We obtained the nucleus accumbens core (NAcc, 75 samples) eQTL dataset from RatGTEx[30] (https://ratgtex.org), which was mapped using Rnor_6.0. We considered associations with $p < 1 \times^{-8}$ between any observed SNP and any gene. We labeled those for which the SNP was within 1Mb of the gene's transcription start site as *cis*-eQTLs, and those with TSS distance greater than 5Mb, or with SNP and gene on different chromosomes, as *trans*-eQTLs. SNP-gene pairs with TSS distance 1-5Mb were not counted in either group. We estimated the set of cross-chromosome genome segment translocations between Rnor_6.0 and mRatBN7.2 using Minimap2[80] with the "asm5" setting. Examples of the relocations were visualized using the NCBI Comparative Genome Viewer (https://ncbi.nlm.nih.gov/genome/cgv/).

Capped small (cs)RNA-seq data

csRNA-seq data used for the alignment metrics were previously published.[75] Briefly, small RNAs of ∼15–60 nt were size selected by denaturing gel electrophoresis starting from total RNA extracted from 14 rat brain tissue dissections. For csRNA libraries, cap selection was followed by decapping, adapter ligation, and sequencing. For input libraries, 10% of small RNA input was used for decapping, adapter ligation, and sequencing. After library quality check by gel electrophoresis, the samples were sequenced using the Illumina NextSeq 500 platform using 75 cycles single end. Sequencing reads were aligned to the Rnor_6.0 and rat mRatBN7.2 genome assembly using STAR v2.5.3a[91] aligner with default parameters. Transcriptional start regions were defined using HOMER's findPeaks tool.[92]

Single nuclei (sn) RNA-seq data

snRNA-seq data used for the alignment metrics were obtained from rat amygdala using the Droplet-based Chromium Single-Cell 3′ solution (10x Genomics, v3 chemistry), as previously described.[96] Briefly, nuclei were isolated from frozen brain tissues and purified by flow cytometry. Sorted nuclei were counted and 12,000 were loaded onto a Chromium Controller (10x Genomics). Libraries were generated using the Chromium Single-Cell 3′ Library Construction Kit v3 (10x Genomics, 1000075) with the Chromium Single-Cell B Chip Kit (10x Genomics, 1000153) and the Chromium i7 Multiplex Kit for sample indexing (10x Genomics, 120262) according to manufacturer specifications. Final library concentration was assessed by Qubit dsDNA HS Assay Kit (Thermo-Fischer Scientific) and post library QC was performed using Tapestation High Sensitivity D1000 (Agilent) to ensure that fragment sizes were distributed as expected. Final libraries were sequenced using the NovaSeq6000 (Illumina). Sequencing reads were aligned to the Rnor_6.0 and rat mRatBN7.2 genome assembly using Cell Ranger 3.1.0.

Transcriptome termini site sequencing

A total of 83 WTTS-seq (whole transcriptome termini site sequencing[29]) libraries were constructed individually using total RNA samples derived from several brain tissues of rats. Library sequencing produced a total of 312,092,803 raw reads, but the mapped reads were 240,225,046 (76.97%) on Rnor6.0, while 251,188,567 (80.49%) on mRatBN7.2, respectively. Using 25 reads per clustered site as a cutoff, we identified 173,124 APA sites mapped to the new reference genome, while 167,136 APA sites were assigned to the old reference genome. For Rnor6.0, only 127,460 (76.26%) APA sites were assigned to the genome regions with 18,177 annotated genes. In contrast, 141,399 (81.67%) APA sites were mapped to the 20,102 annotated genes on mRatBN7.2. In brief, our results provide evidence that mRatBN7.2 has improved qualities of both genome assembly and gene annotation in rats.

Brain proteome data

Deep proteome data were generated using whole brain tissue from both parents and 29 members of the HXB family, one male and one female per strain. Proteins in these samples were identified and quantified using the tandem-mass-tag (TMT) labeling strategy coupled with two-dimensional liquid chromatography-tandem mass spectrometry (LC/LC-MS/MS). We used the QTLtools program[93] for protein expression quantitative trait locus (pQTL) mapping. *cis*-pQTL are defined when the transcriptional start sites for the tested protein are located within ±1 Mb of each other.

Identifying potentially mislabeled samples

Phylogenetic analysis identified that the metadata of 15 samples contradicted their genetic relationships. These contradictions could happen at many steps during breeding, samples collection, sequencing, or data analysis and the true source is difficult to pinpoint. An advantage of having multiple biological samples for each strain is that these inconsistencies can be identified. One of the 15 samples is mislabeled and the correct label can be inferred (sample name denoted with ***); two samples are mislabeled and the correct labels cannot be inferred (sample names denoted with **); 12 samples are potentially mislabeled (sample names denoted with *). Details of these samples are described below.

(1) There is enough evidence to indicate that this sample is mislabeled, and we can conclusively infer what the true label is. One sample falls into this category: F344/Stm_HCJL.CRM. Despite being named F344, this sample has less than 70% IBS with the other 14 F344 samples, yet has about 99% IBS with 2 LE samples from different institutes (Table S10). We think this sample is mislabeled as F344, and the true label should be LE. Therefore, we changed the sample name accordingly and appended *** to the end of the sample name to denote such a change has been made.

(2) There is enough evidence to indicate that this sample is mislabeled, but we cannot conclusively infer what the true label is. Two samples fall into this category: LE/Stm_HCJL.CRM has about 83% IBS with the other 3 LE samples from different institutes (Table S10). The identity of this sample is likely one of the LEXF or FXLE recombinant inbred, but there isn't another sample that has high IBS with it. We think this sample is mislabeled, but the true label is unknown. WKY/Gla_TA.ILM is a sample we downloaded from SRA. This WKY substrain (WKY/Gla) clusters closer to SHR strains than other WKY substrains. The same pattern was also observed in one prior study,[41] and was thought to be caused by the incomplete inbreeding before sample distribution. To further investigate this, we performed regional similarity analysis and found that the pattern observed is consistent with that of a congenic strain created by using SHR as the recipient and WKY as the donor. A literature search confirmed that such strains were indeed once created at the same institute from where WKY/Gla was derived..[97] We think this sample is mislabeled, but we don't know what the correct label should be.

(3) There is evidence to suggest that this sample could potentially be mislabeled, but evidence is not conclusive. A total of 12 samples fall in this category. The majority of the samples of the same substrain but sequenced by different institutes have IBS over 99%; however, we observed a few instances of unexpected low IBS between samples of the same strain but with unexpected high IBS between samples of a different strain. These could be caused by the mis-labeling at either of the institutes. Although we think these samples are at the risk of being mislabeled, it is also possible that individual differences with these strains/substrains could be a cause of the unexpected IBS values. For example, both 7.7% of the variants of the BXH2 samples are heterozygous, while the rate of heterozygosity in the LEXF/FXLE in general is higher than the rest of the inbred strains. These pairs include (Table S10):

- BXH2_MD.ILM & BXH2_HCRW.CRM: unexpected low IBS at 92%
- LEXF4_MD.ILM & LEXF5_HCJL.CRM: unexpected high IBS at 99%
- LEXF3_MD.ILM & LEXF4_HCJL.CRM: unexpected high IBS at 99%
- LEXF1A_MD.ILM & LEXF1C_HCJL.CRM: unexpected high IBS at 99%
- LEXF1C_MD.ILM & LEXF2A_HCJL.CRM: unexpected high IBS at 99%
- LEXF2B_MD.ILM & LEXF1A_HCJL.CRM: unexpected high IBS at 98%
- LEXF4_MD.ILM & LEXF4_HCJL.CRM: unexpected low IBS at 83%
- LEXF1A_MD.ILM & LEXF1A_HCJL.CRM: unexpected low IBS at 84%
- LEXF1C_MD.ILM & LEXF1C_HCJL.CRM: unexpected low IBS at 95%

Ensembl annotation

Annotation of the assembly was created via the Ensembl gene annotation system.[98] A set of potential transcripts was generated using multiple techniques: primarily through alignment of transcriptomic datasets, cDNA sequences, curated evidence, and also through gap filling with protein-to-genome alignments of a subset of mammalian proteins with experimental evidence from UniProt.[94] Additionally, a whole genome alignment was generated between the genome and the GRCm39 mouse reference genome using LastZ and the resulting alignment was used to map the coding regions of mouse genes from the GENCODE reference set.

The short-read RNA-seq data was retrieved from two publicly available projects; PRJEB6938, representing a wide range of different tissue samples such as liver or kidney, and PRJEB1924 which is aimed at understanding olfactory receptor genes. A subset of samples from a long-read sequencing project PRJNA517125 (SRR8487230, SRR8487231) were selected to provide high quality full length cDNAs.

From the 104 Ensembl release annotation on the rat assembly Rnor_6.0, we retrieved the sequences of manually annotated transcripts from the HAVANA manual annotation team. These were primarily clinically relevant transcripts, and represented high confidence cDNA sequences. Using the *Rattus norvegicus* taxonomy id 10116, cDNA sequences were downloaded from ENA and sequences with the accession prefix 'NM' from RefSeq.[76]

The UniProt mammalian proteins had experimental evidence for existence at the protein or transcript level (protein existence level 1 and 2).

At each locus, low quality transcript models were removed, and the data were collapsed and consolidated into a final gene model plus its associated non-redundant transcript set. When collapsing the data, priority was given to models derived from transcriptomic data, cDNA sequences and manually annotated sequences. For each putative transcript, the coverage of the longest open reading frame was assessed in relation to known vertebrate proteins, to help differentiate between true isoforms and fragments. In loci where the transcriptomic data were fragmented or missing, homology data was used to gap fill if a more complete cross-species alignment was available, with preference given to longer transcripts that had strong intron support from the short-read data.

Gene models were classified, based on the alignment quality of their supporting evidence, into three main types: protein-coding, pseudogene, and long non-coding RNA. Models with hits to known proteins, and few structural abnormalities (i.e., they had canonical splice sites, introns passing a minimum size threshold, low level of repeat coverage) were classified as protein-coding. Models with hits to known protein, but having multiple issues in their underlying structure, were classified as pseudogenes. Single-exon models with a corresponding multi-exon copy elsewhere in the genome were classified as processed pseudogenes.

If a model failed to meet the criteria of any of the previously described categories, did not overlap a protein-coding gene, and had been constructed from transcriptomic data then it was considered as a potential lncRNA. Potential lncRNAs were filtered to remove transcripts that did not have at least two valid splice sites or cover 1000bp (to remove transcriptional noise).

A separate pipeline was run to annotate small non-coding genes. miRNAs were annotated via a BLAST[99] of miRbase[100] against the genome, before passing the results into RNAfold.[101] Poor quality and repeat-ridden alignments were discarded. Other types of small non-coding genes were annotated by scanning Rfam[102] against the genome and passing the results into Infernal.[103]

The annotation for the rat assembly was made available as part of Ensembl release 105.

RefSeq annotation

Annotation of the mRatBN7.2 assembly was generated for NCBI's RefSeq dataset[76] using NCBI's Eukaryotic Genome Annotation Pipeline.[104] The annotation, referred to as NCBI *Rattus norvegicus* Annotation Release 108, includes gene models from curated and computational sources for protein-coding and non-coding genes and pseudogenes, and is available from NCBI's genome FTP site and web resources.

Most protein-coding genes and some non-coding genes are represented by at least one known RefSeq transcript, labeled by the method "BestRefSeq" and assigned a transcript accession starting with NM_ or NR_, and corresponding RefSeq proteins designated with NP_ accessions. These are predominantly based on rat mRNAs subject to manual and automated curation by the RefSeq team for over 20 years, including automated quality analyses and comparisons to the Rnor_6.0 and mRatBN7.2 assemblies to refine the annotations. Nearly 80% of the protein-coding genes in AR108 include at least one NM_ RefSeq transcript, of which 33% have been fully reviewed by RefSeq curators.

Additional gene, transcript, and protein models were predicted using NCBI's Gnomon algorithm using alignments of transcripts, proteins, and RNA-seq data as evidence. The evidence datasets used for Release 108 are described at https://www.ncbi.nlm.nih.gov/genome/annotation_euk/Rattus_norvegicus/108/, and included alignments of available rat mRNAs and ESTs, 10.7 billion RNA-seq reads from 303 SRA runs from a wide range of samples, 1 million Oxford Nanopore or PacBio transcript reads from 5 SRA runs, and known RefSeq proteins from human, mouse, and rat. BestRefSeq and Gnomon models were combined to generate the final annotation, compared to the previous Release 106 annotation of Rnor_6.0 to retain GeneID, transcript, and protein accessions for equivalent annotations, and compared to the RefSeq annotation of human GRCh38 to identify orthologous genes. Gene nomenclature was based on data from RGD, curated names, and human orthologs.

Cell Genomics, Volume *4*

Supplemental information

A revamped rat reference genome improves the discovery of genetic diversity in laboratory rats

Tristan V. de Jong, Yanchao Pan, Pasi Rastas, Daniel Munro, Monika Tutaj, Huda Akil, Chris Benner, Denghui Chen, Apurva S. Chitre, William Chow, Vincenza Colonna, Clifton L. Dalgard, Wendy M. Demos, Peter A. Doris, Erik Garrison, Aron M. Geurts, Hakan M. Gunturkun, Victor Guryev, Thibaut Hourlier, Kerstin Howe, Jun Huang, Ted Kalbfleisch, Panjun Kim, Ling Li, Spencer Mahaffey, Fergal J. Martin, Pejman Mohammadi, Ayse Bilge Ozel, Oksana Polesskaya, Michal Pravenec, Pjotr Prins, Jonathan Sebat, Jennifer R. Smith, Leah C. Solberg Woods, Boris Tabakoff, Alan Tracey, Marcela Uliano-Silva, Flavia Villani, Hongyang Wang, Burt M. Sharp, Francesca Telese, Zhihua Jiang, Laura Saba, Xusheng Wang, Terence D. Murphy, Abraham A. Palmer, Anne E. Kwitek, Melinda R. Dwinell, Robert W. Williams, Jun Z. Li, and Hao Chen

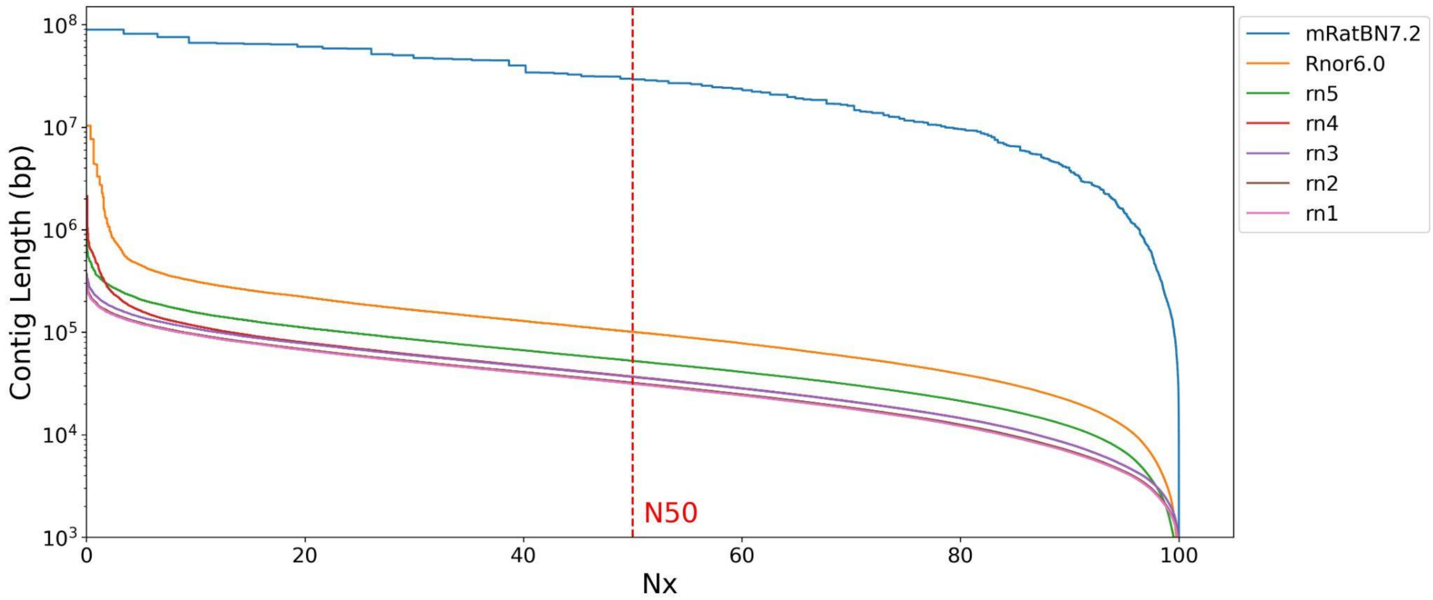

Figure S1 (related to Figure 1). The Contig Nx plot for mRatBN7.2 and 6 prior rat reference genomes. The red dashed line indicates the contig N50 values. Small improvements in contig continuity are observed for the previous updates, with the most significant improvement (~290X) coming from mRatBN7.2

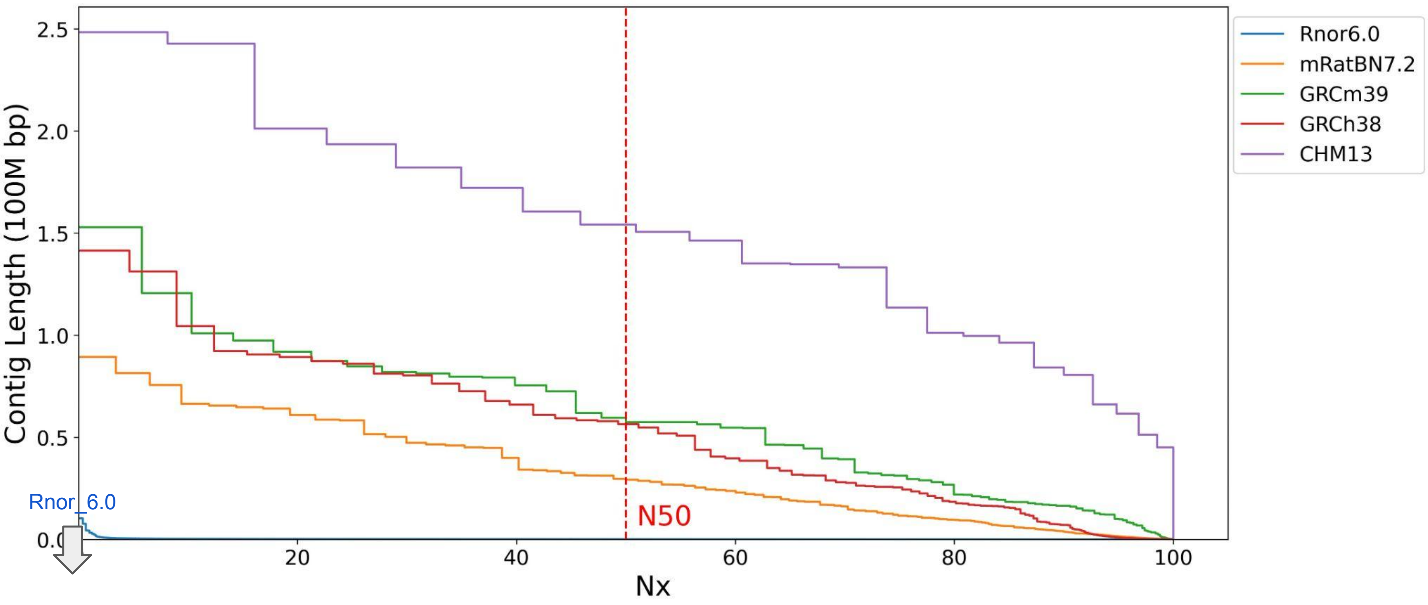

Figure S2 (related to Figure 1). The Contig Nx plot for the Rat, Mouse, and Human reference genomes. The red dashed line indicates the contig N50 values. The Nx curve for Rnor6.0 is very low and partially overlapping with the X axis. The CHM13, being the first truly gapless human genome, is in its own tier in terms of assembly continuity. The GRCh38 released in 2014 and the GRCm39 released in 2020 have similar continuity. Although mRatBN7.2 is still lagging behind in continuity compared to the current human and mouse reference genomes, it represent a very significant improvement over the current rat reference genome Rnor_6.0 (Orange vs. Cyan line).

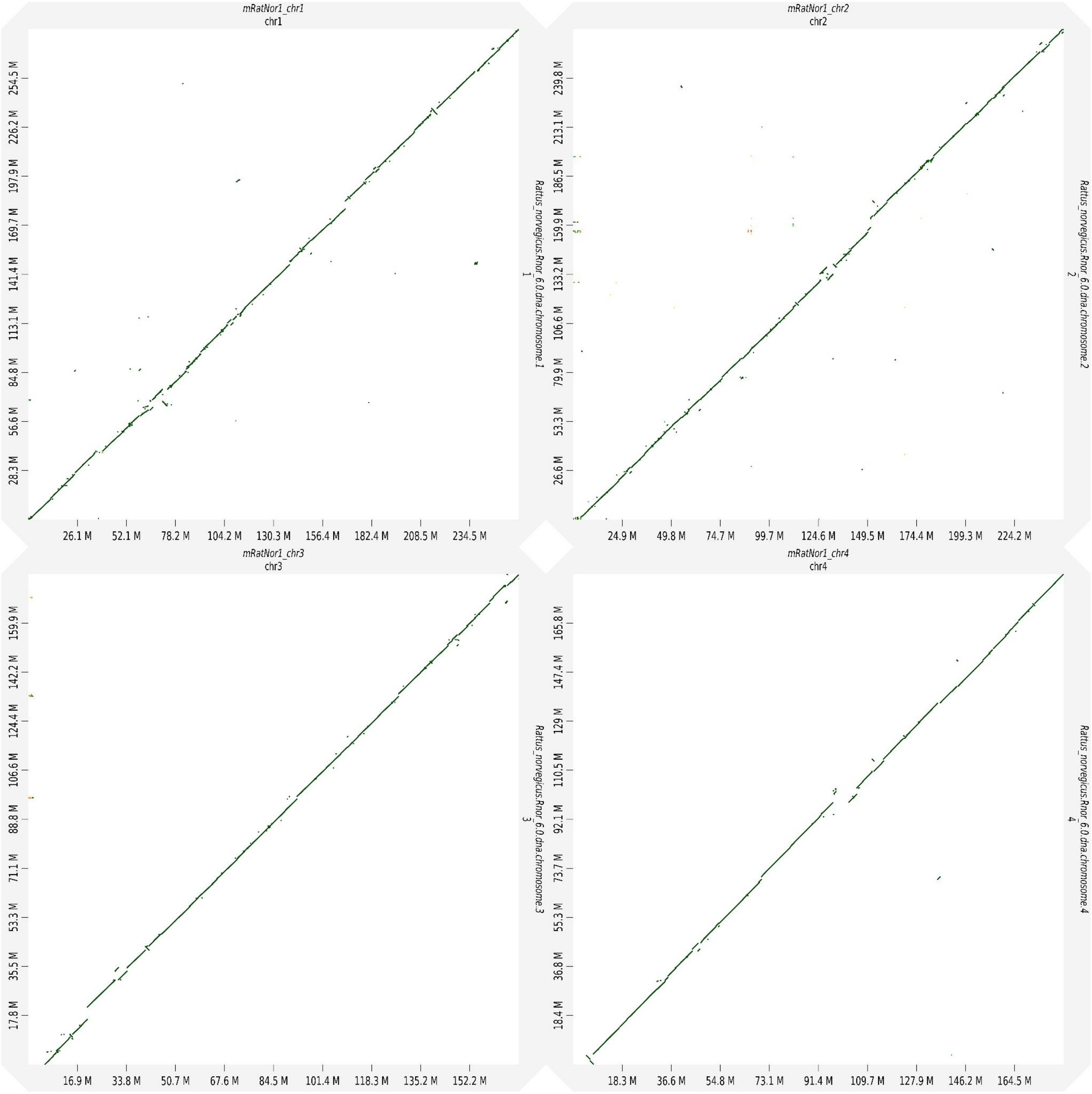

Figure S3 (related to Figure 1). Chromosomal dot plots between Rnor_6.0 and mRatBN7.2. mRatBN7.2 is on the x-axis and Rnor_6.0 is on the y-axis. mRatNor1 is the initial name of the assembly released by the Darwin Tree of Life/Vertebrate Genome Project. chr1-chr4 are shown here. To see the complete set, see the Key Resources Table "Chromosomal dot plots between Rnor_6.0 and mRatBN7.2" https://doi.org/10.5281/zenodo.10515796

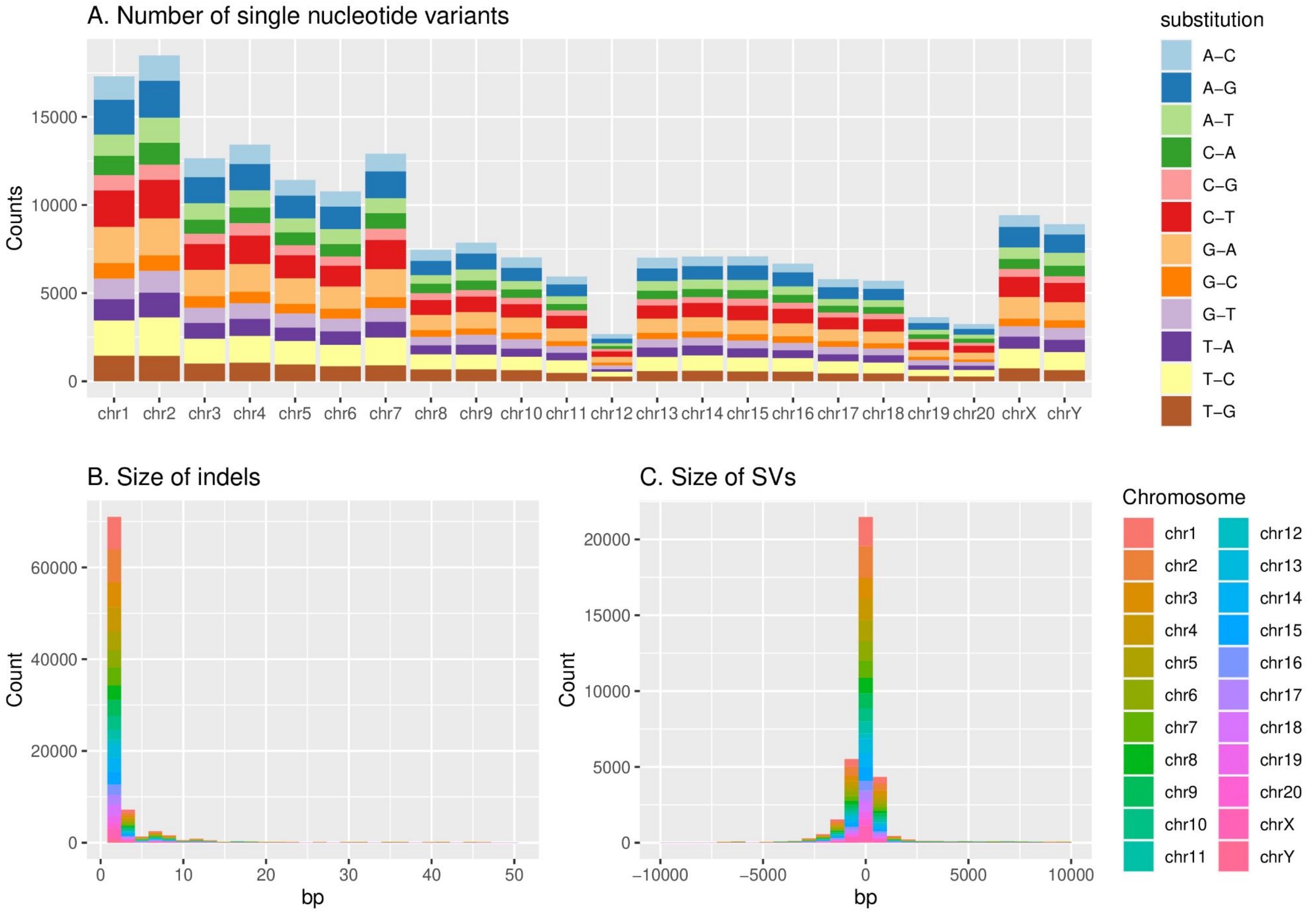

Figure S4 (related to Figure 1). SNP and structural variants between Rnor_6.0 and mRatBN7.2. A) The substitution frequency per chromosome for Rnor_6.0 and mRatBN7.2. **B)** The size of indels between Rnor 6.0 and mRatBN7.2. **C)** The size of SVs between Rnor6.0 and mRatBN7.2.

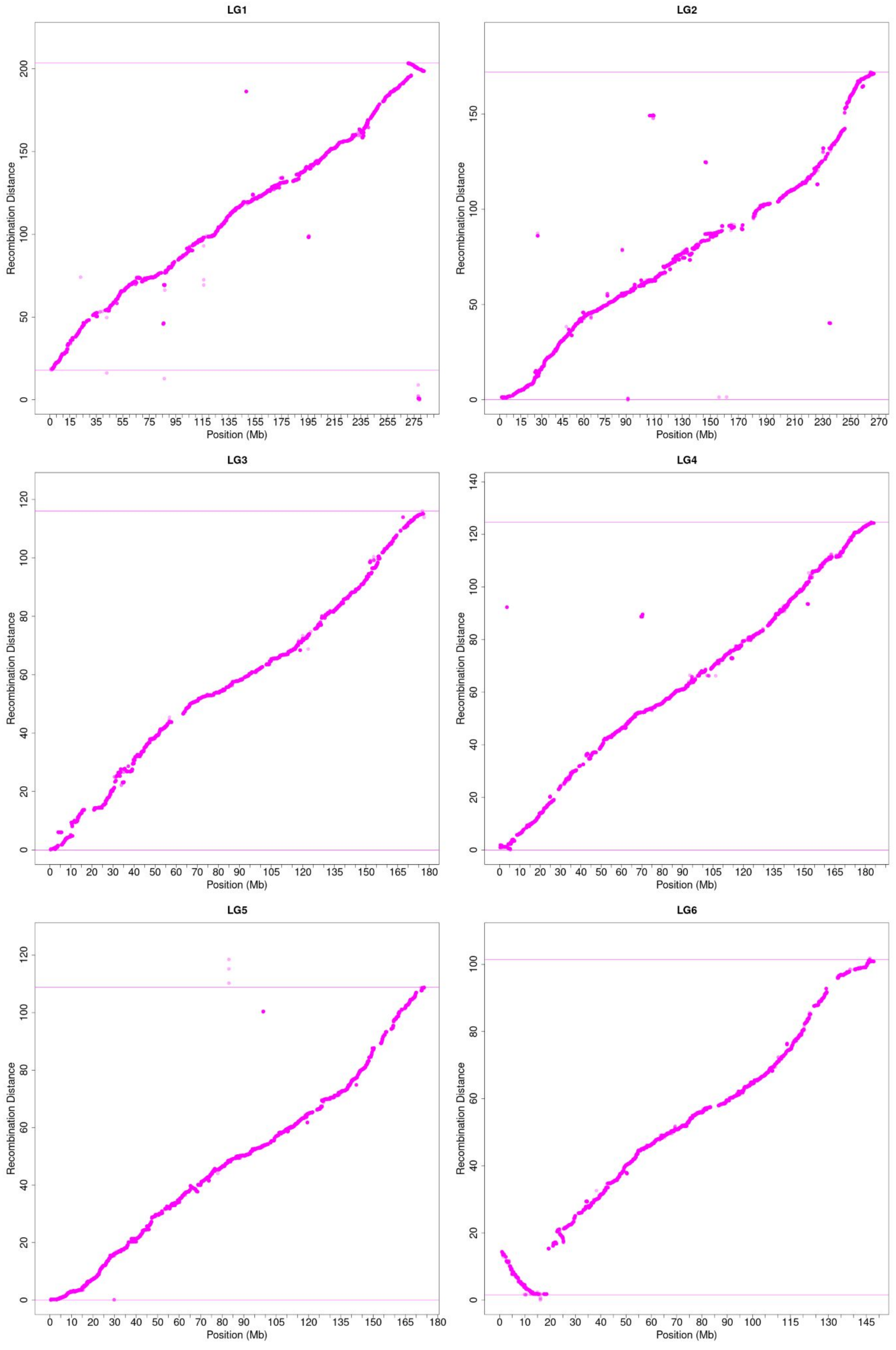

Figure S5 (related to Figure 1). The order of genetic markers and the distances from a rat genetic map compared to their locations in Rnor_6.0. Note the large inversion at proximal Chr 6. chr1-chr6 are shown here. To see the complete set, see the Key Resources Table "The order of genetic markers and the distances from a rat genetic map compared to their locations in Rnor_6.0" https://doi.org/10.5281/zenodo.10520087

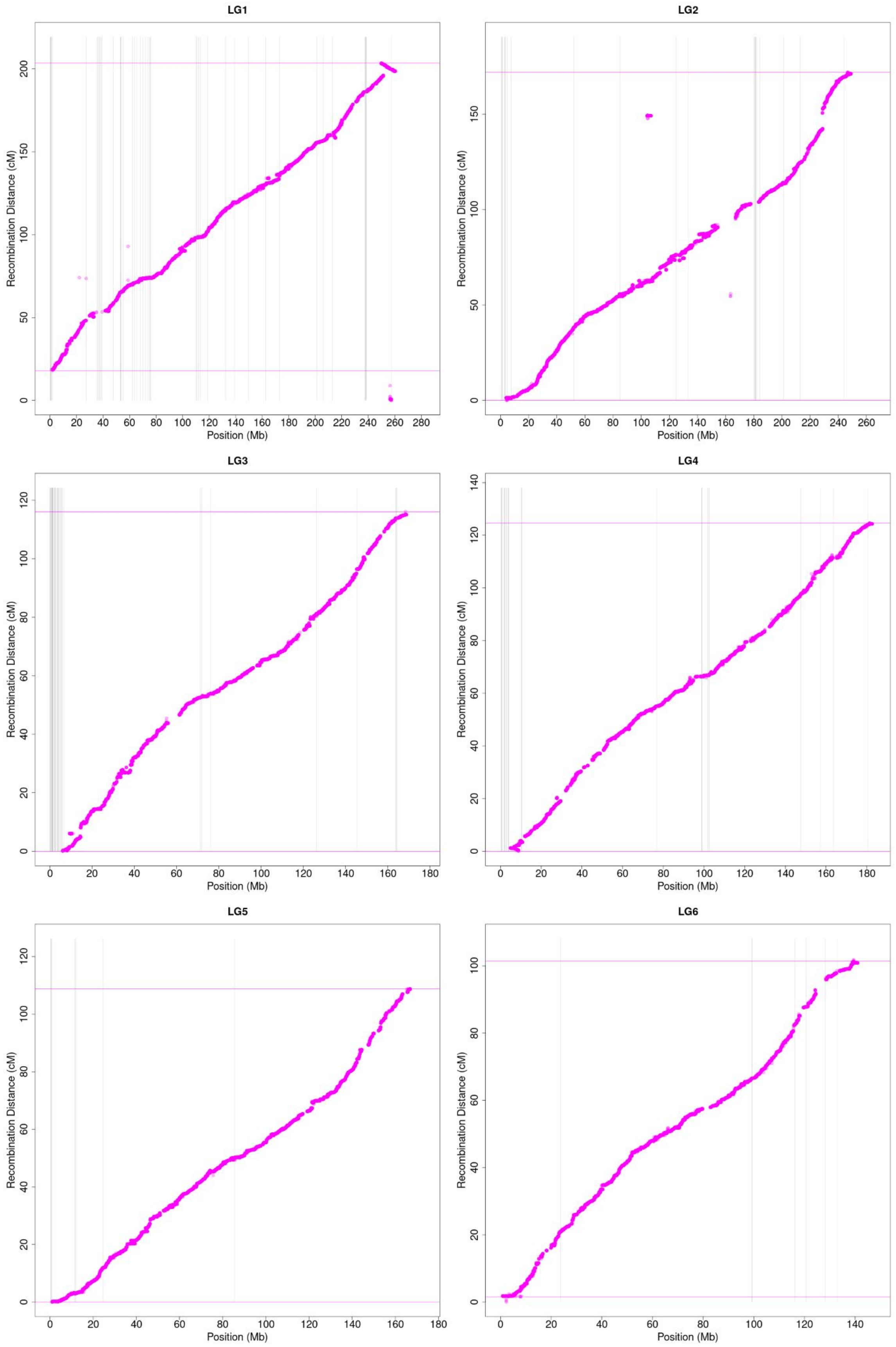

Figure S6 (related to Figure 1). The order of genetic markers and the distances from a rat genetic map compared to their locations in mRatBN7.2 chr1-chr6 are shown here. To see the complete set, see the Key Resources Table "The order of genetic markers and the distances from a rat genetic map compared to their locations in mRatBN7.2" https://doi.org/10.5281/zenodo.10520119

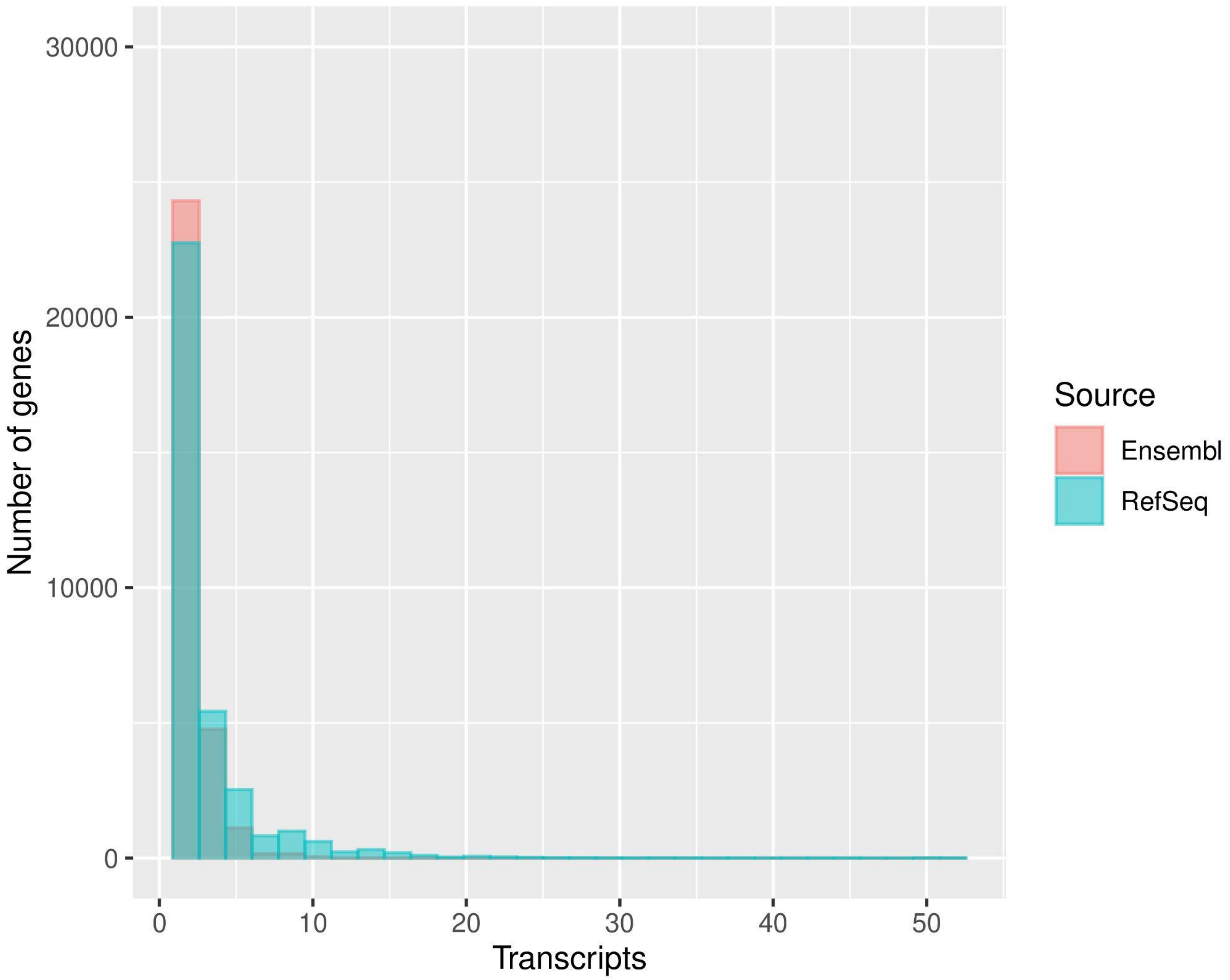

Figure S7 (related to Figure 1). The number of transcripts annotated by Ensembl and RefSeq. Refseq annotated a greater number of genes with multiple transcripts than Ensembl. The average number of transcripts per gene was 2.9 for RefSeq and 1.8 for Ensembl.

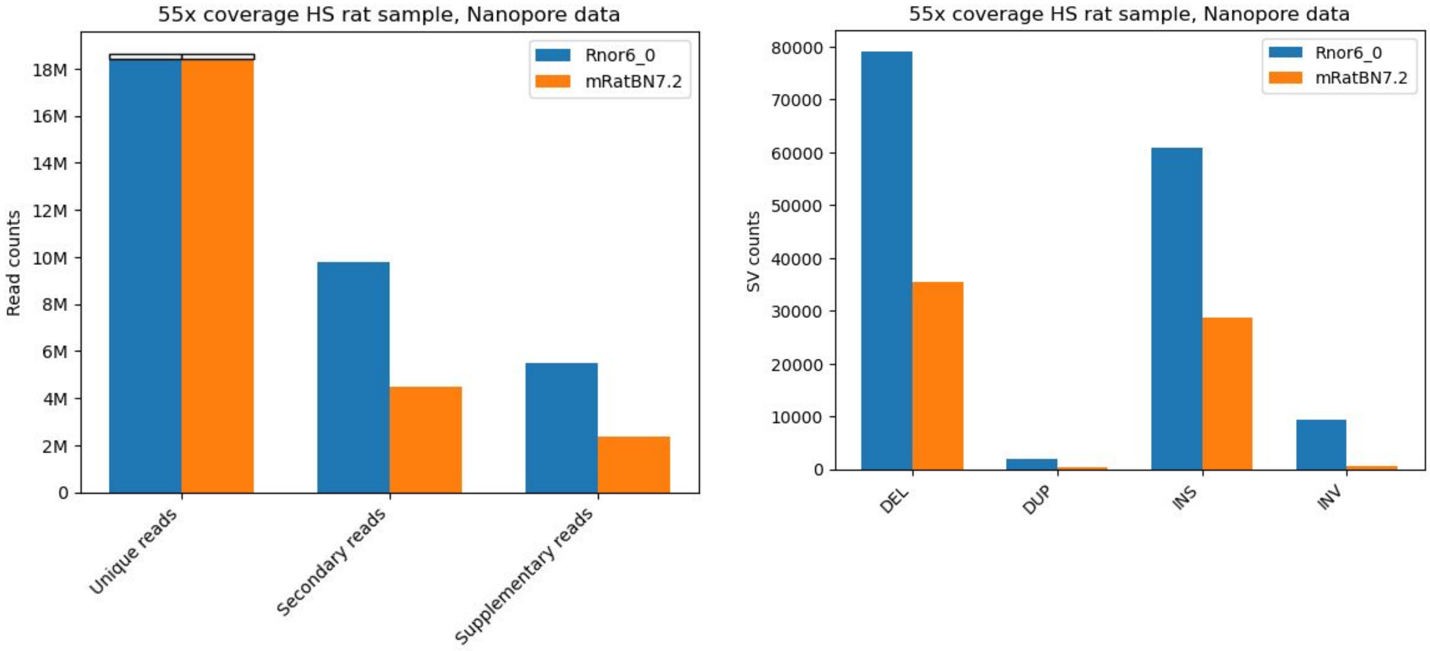

Figure S8 (related to Figure 2). Mapping results of nanopore data of one HS rat with 55x coverage. Using mRatBN7.2 as the reference decreased the number of structural variants compared to using Rnor_6.0 as the reference. In the context of our other findings, this is likely due to the improved quality of the reference genome.

A

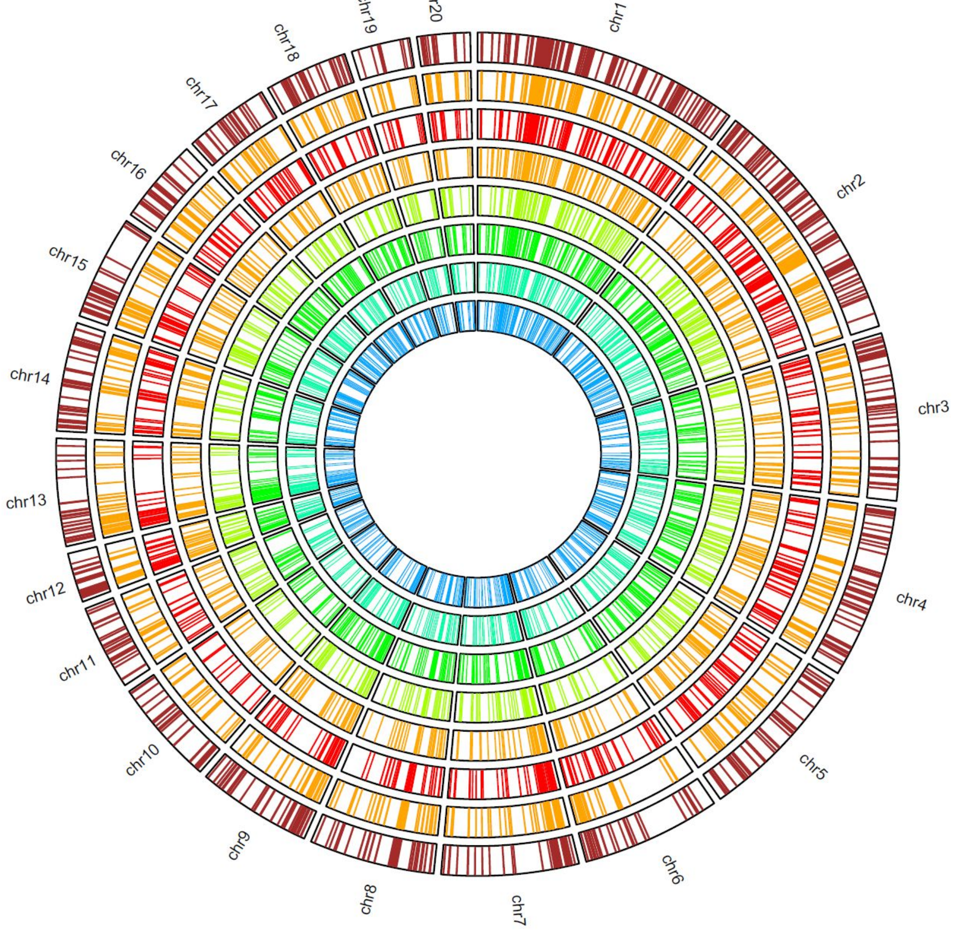

B

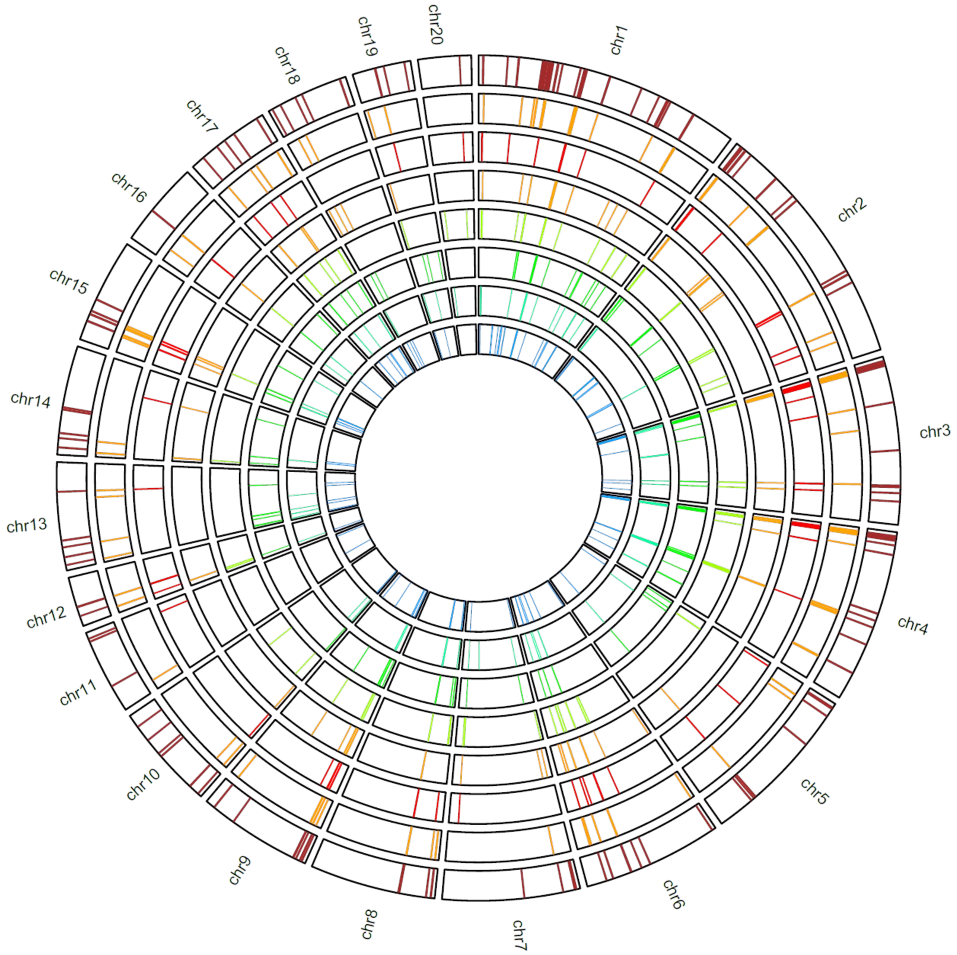

Figure S9 (related to Figure 2). Locations of large deletions from multiple linked-read samples. A) Rnor_6.0 B) mRatBN7.2. From outer circle to inner circle are the following strains: BN, SHR/Olalpcv, BXH10, BXH8, BXH2, HXB17, HXB2, and HXB21.

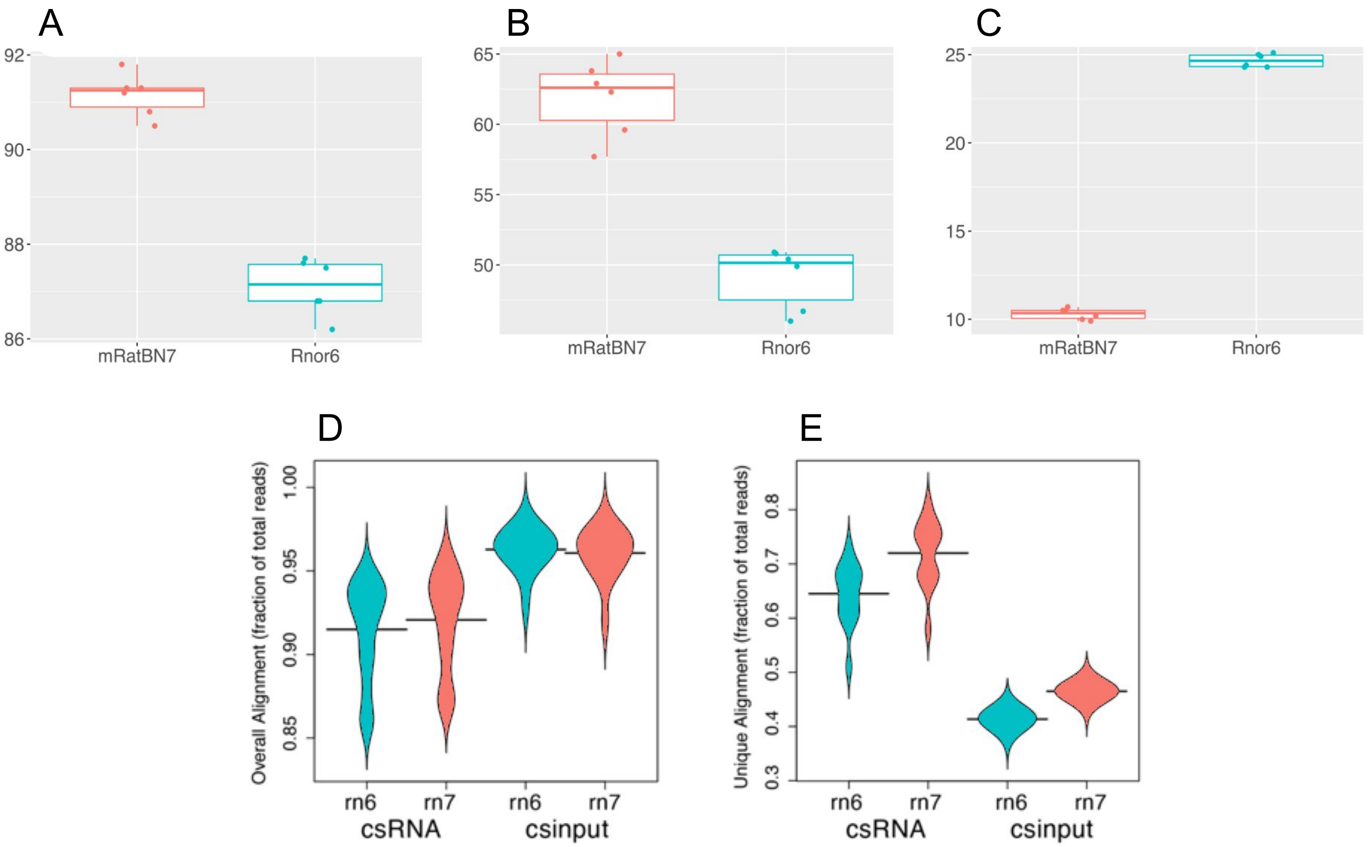

Figure S10 (related to Figure 2). Mapping metrics of single nuclei RNA-seq and small capped RNA-seq data generated using rat brain tissues. Percentage of snRNA-seq reads mapped to A) the genome, B) the transcriptome, and C) an intergenic region of the genome for mRatBN7.2 and Rnor_6.0. D) Overall mapping rates of csRNA-seq data to two reference genomes. E) Unique alignment of csRNA-seq data to two reference genomes.

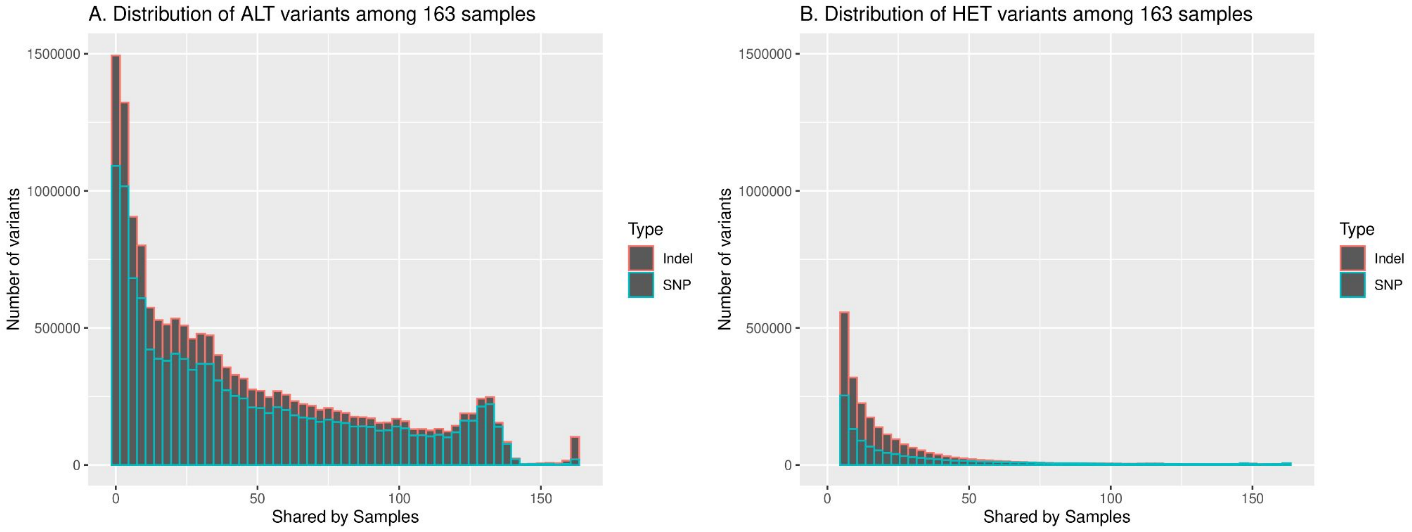

Figure S11 (related to Figure 6). Distribution of the total number of variants shared by 1 or more samples across all 163 samples for A) Homozygous alternative SNPs/Indels. The lack of variants shared by approximately 140-155 samples and the distinct peak after 155 samples indicate variants shared by more than 156 samples are likely caused by errors in the reference genome. B) Heterozygous SNPs/Indels. Similar pattern as A can be observed.

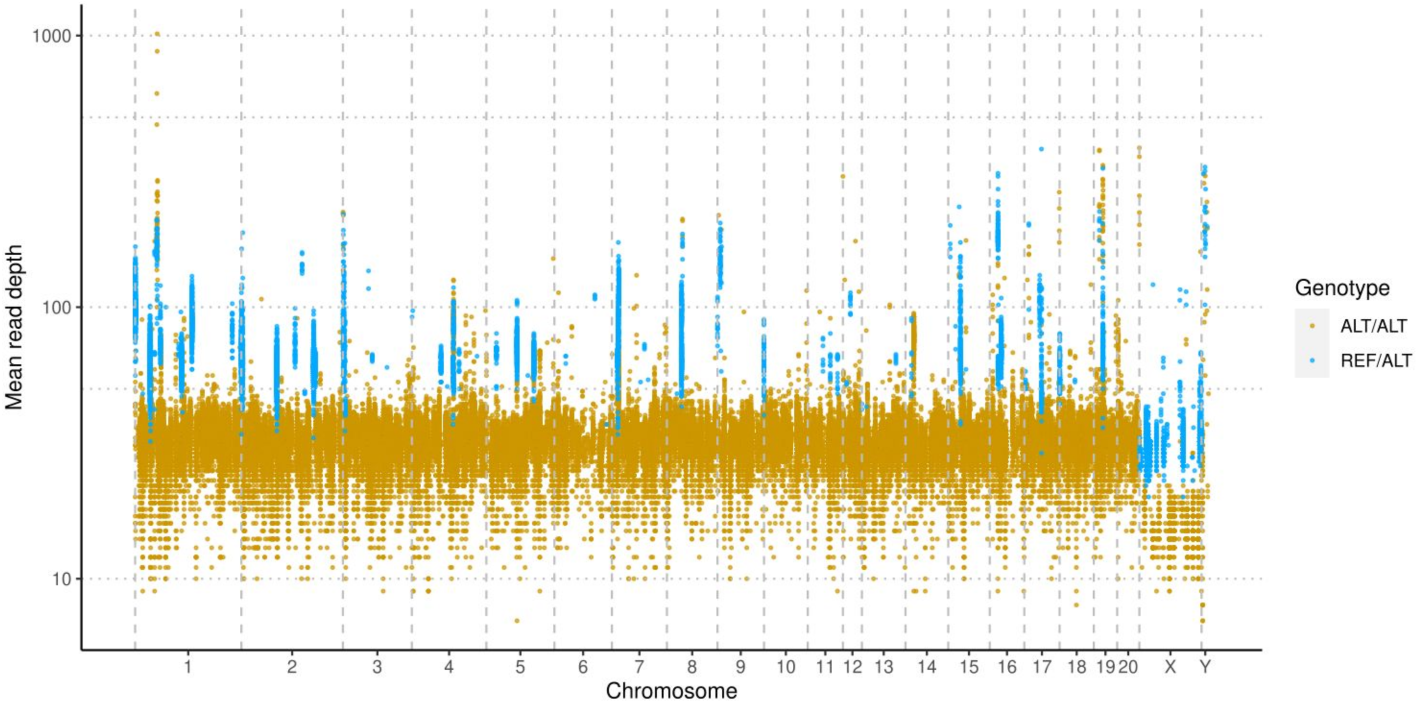

Figure S12 (related to Figure 6). SNPs and indels indicate remaining errors in mRatBN7.2. Base-level errors are indicated by homozygous variants that are shared by the majority (i.e. more than 153 out of 163) of samples, including all seven BN/NHsdMcwi rats, one of them is part of the data used to assemble mRatBN7.2. Variants that are heterozygous for the majority of the samples are clustered in a few regions and have significantly higher read-depth. This suggests that they originated from collapsed repeats in mRatBN7.2.

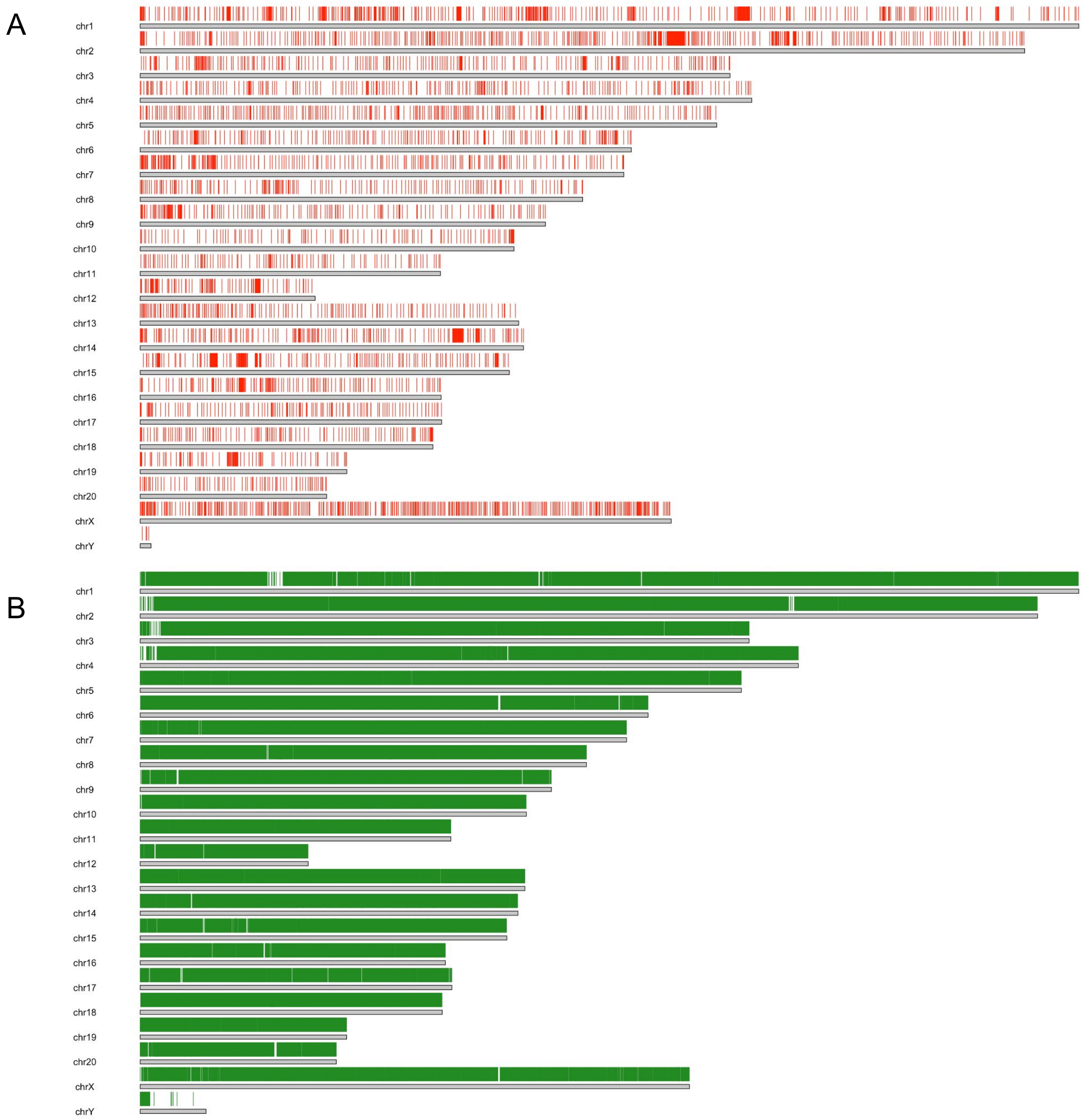

Figure S13 (related to Figure 4). Simulated liftover analysis: unliftable and lifted site distribution. An evenly 1000 bp spaced bed file covering Rnor_6.0 is generated and then lifted to mRatBN7.2. Out of the 2,782,023 sites, 92.04% are liftable, 7.96% are not liftable. A) The distribution of the unliftable sites are plotted on Rnor_6.0. B) The distribution of the lifted sites are plotted on mRatBN7.2. The data are subsampled by 50 for better visualization.

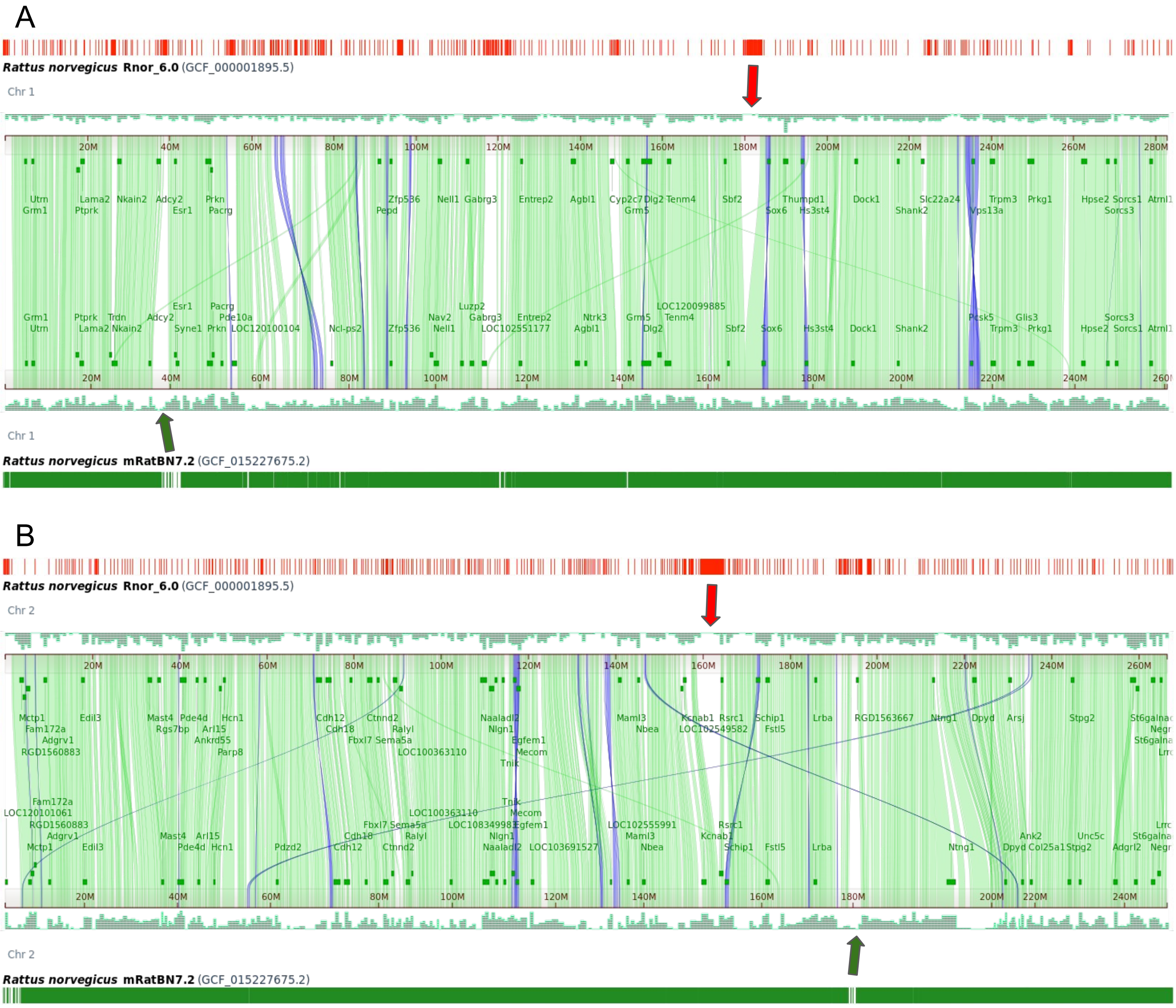

Figure S14 (related to Figure 4). Simulated liftover analysis and comparative genome view. Top red track is the unliftable sites distributed on the Rnor_6.0. Bottom green track is the lifted sites distributed on the mRatBN7.2. The middle is the NCBI comparative genome viewer between chr1 of Rnor_6.0 and mRatBN7.2. The red arrow highlights a region that's not liftable in Rnor_6.0. According to the comparative genome view, the region has no corresponding region in mRatBN7.2 (not just chr1). The green arrow highlights a regiono in mRatBN7.2 that has no lifted sites from Rnor_6.0. According to the comparative genome view, this region has matching region in Rnor_6.0. A) Chromosome 1. B) Chromosome 2.

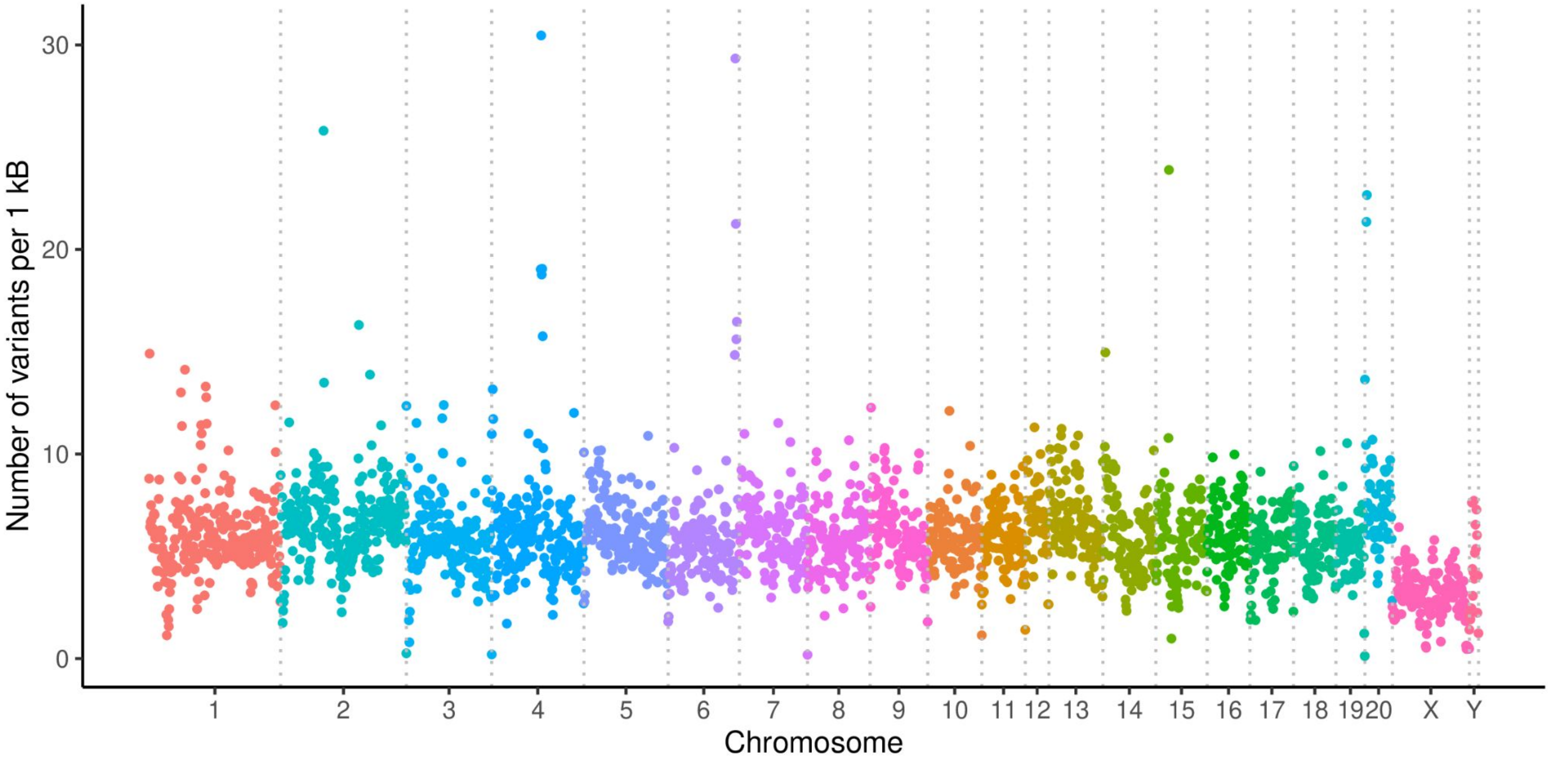

Figure S15 (related to Figure 5). Density of variants across the genome in a collection of 163 rat WGS samples. The mean variant density was 5.96 ± 2.20/Kb (mean ± SD). The highest variant density of 30.5/Kb was found on Chr 4 at 98 Mb.

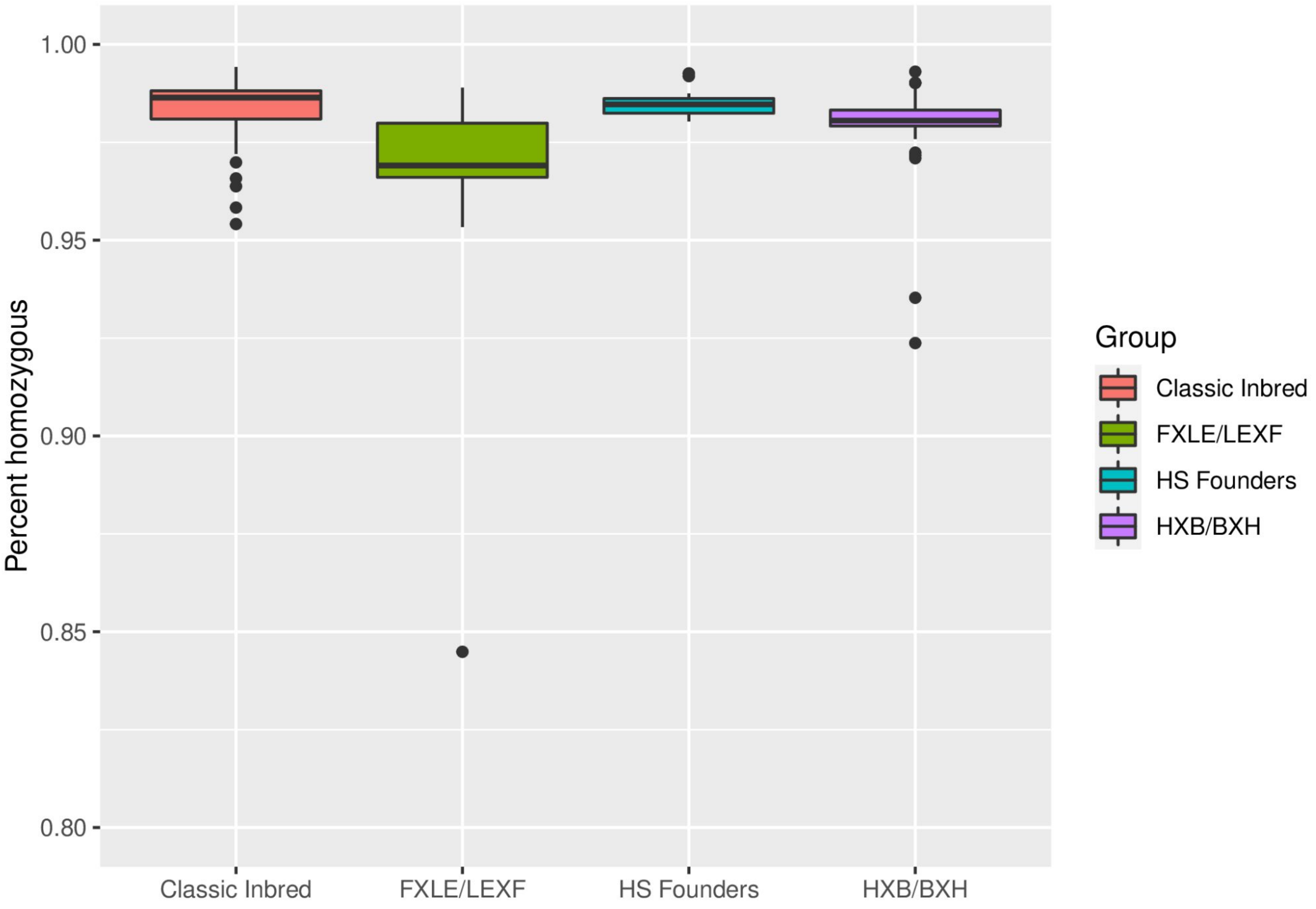

Figure S16 (related to Figure 5). Homozygosity of inbred strains. Variants in most sample were homozygous, confirming the inbred nature of most strains. A few exceptions were noted. For example, 15.6% of the variants from FXLE24 were heterozygous. Additionally, BXH2, which we sequenced two samples, has ~7.7% heterozygous variants.

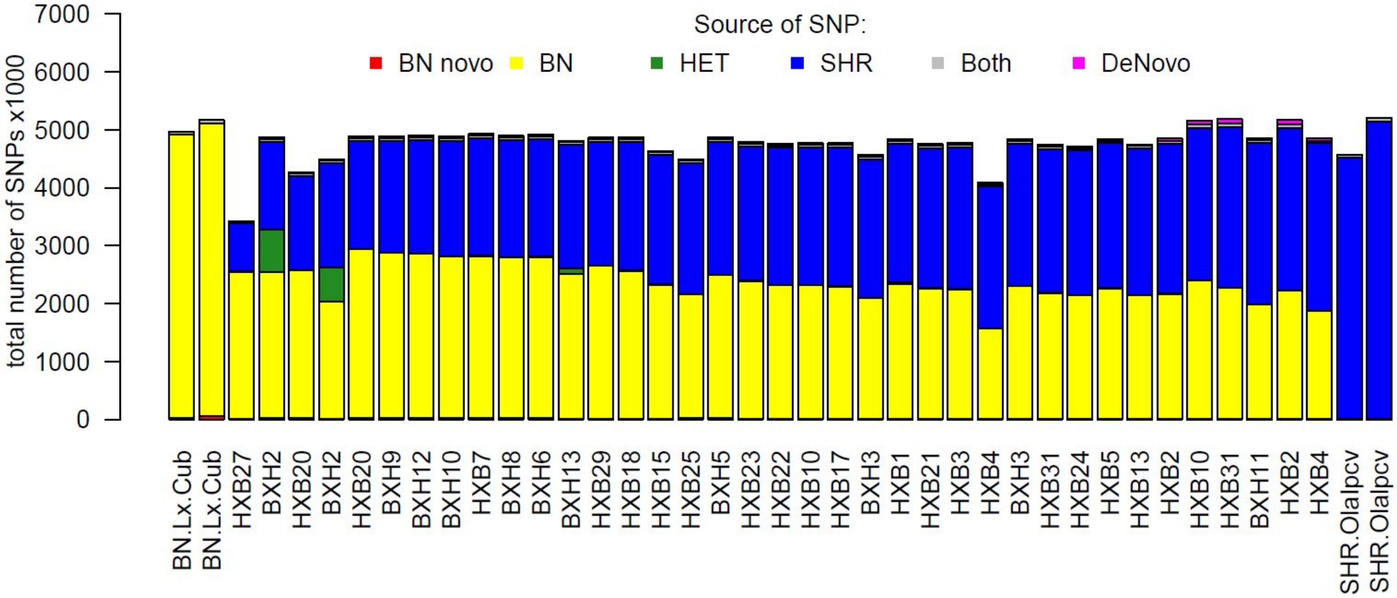

Figure S17 (related to Figure 5). The majority of the variants in HXB/BXH RI panel are inherited from the parental strains. De Novo Mutations on BN.Lx (Red) Mutations originating from BN (Yellow), Heterozygous (Green), Mutations originating from SHR (Blue), SNPs found on both BN and SHR (Grey), De Novo SNPs from neither parental strains (Pink).

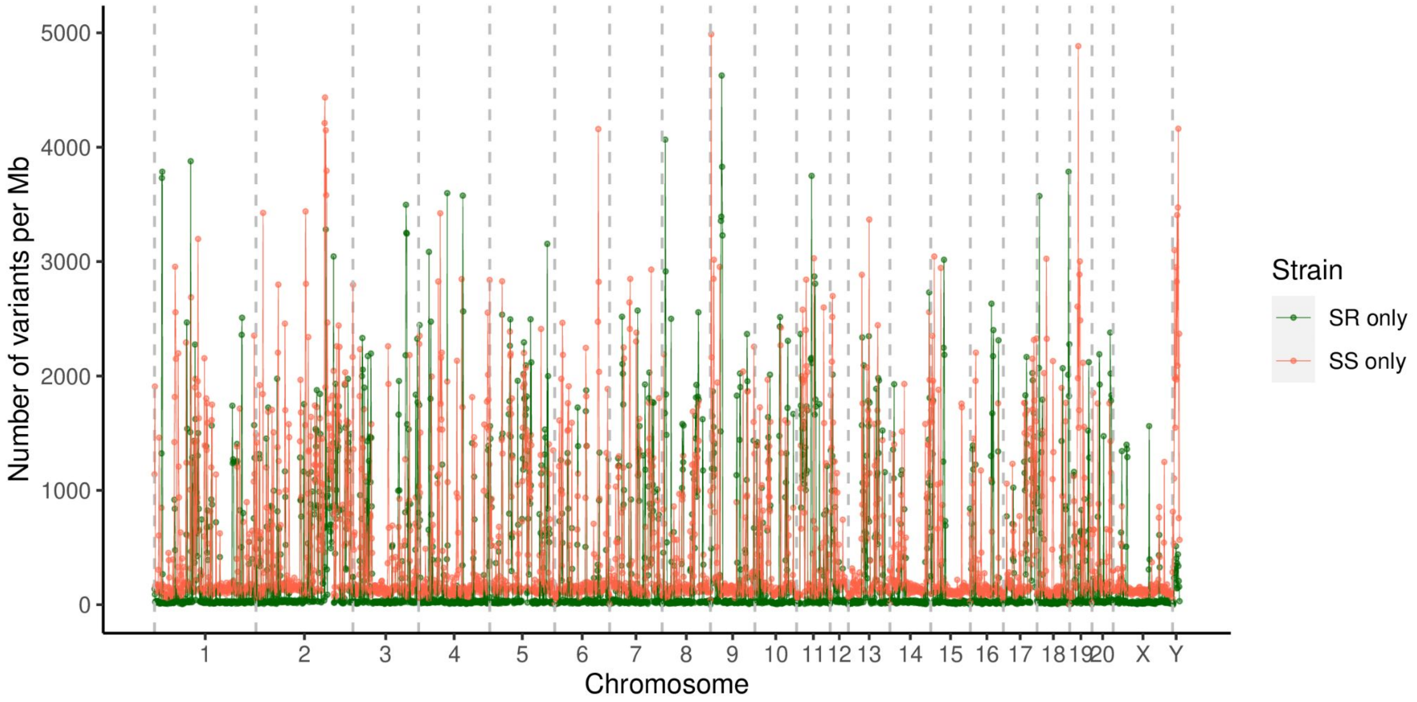

Figure S18 (related to Figure 5). Distribution of variants different between SS and SR rats. Total number of variants unique to either SS or SR rats across the genome. The SS strain contains more unique variants than SR.

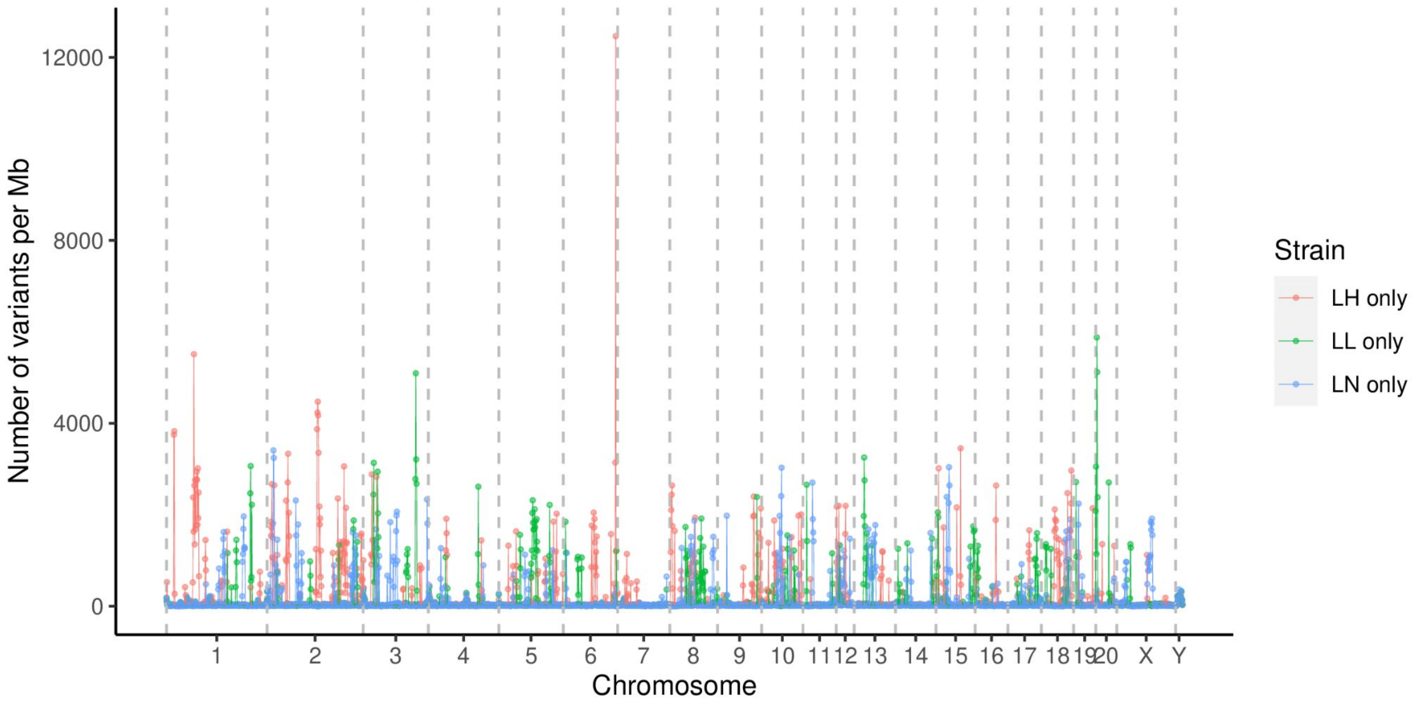

Figure S19 (related to Figure 5). Distribution of variants unique to LL/LH/LN rats. Strain-specific variants tend to cluster by strain.

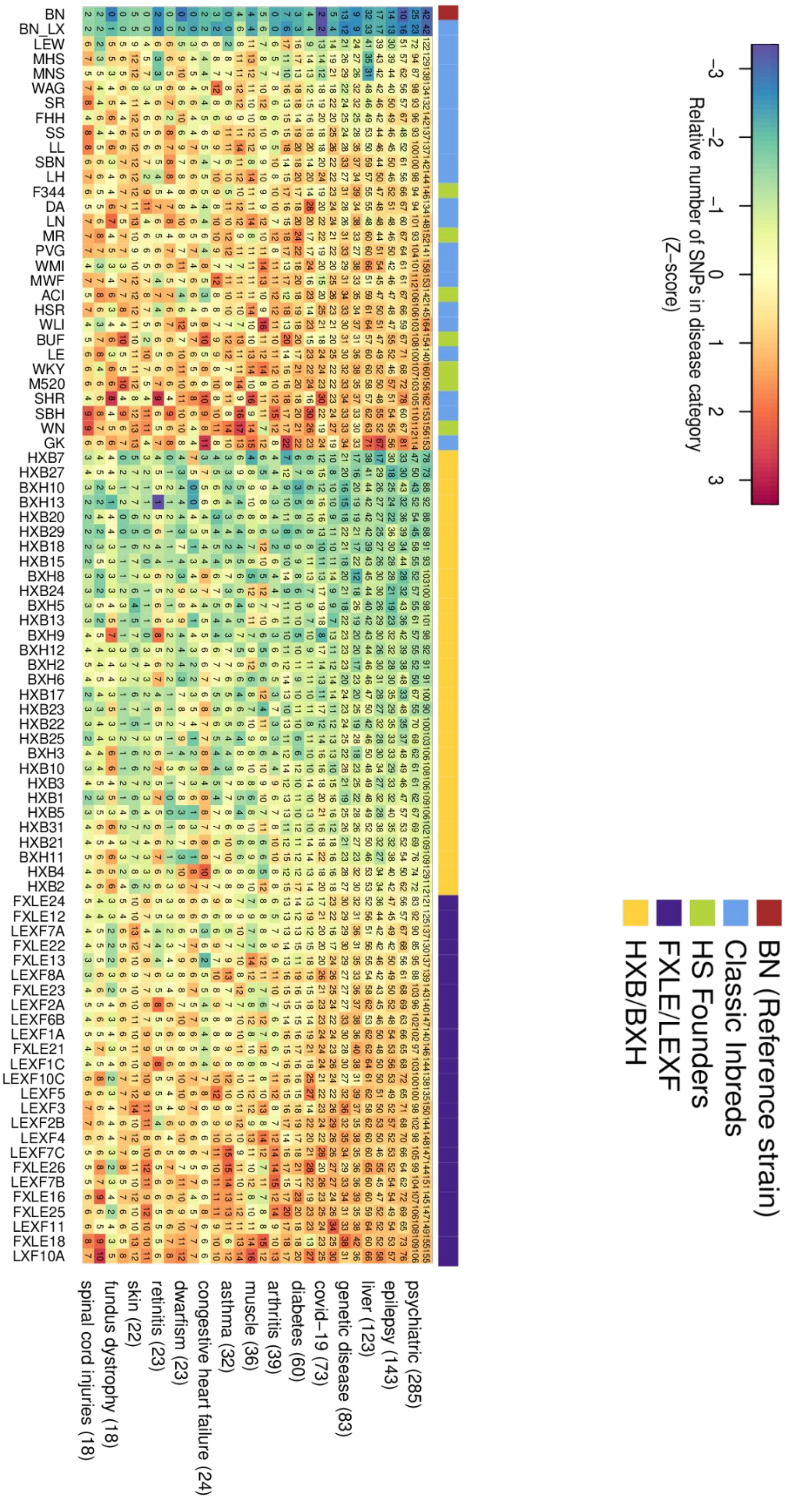

Figure S20 (related to Figure 5). Overview of disease ontology annotation of variants in RatCollection. Heat maps shows relative number of SNPs in proximity of genes associated per disease category within the reference, classic inbreds, HS progenitors and recombinant inbred panels.

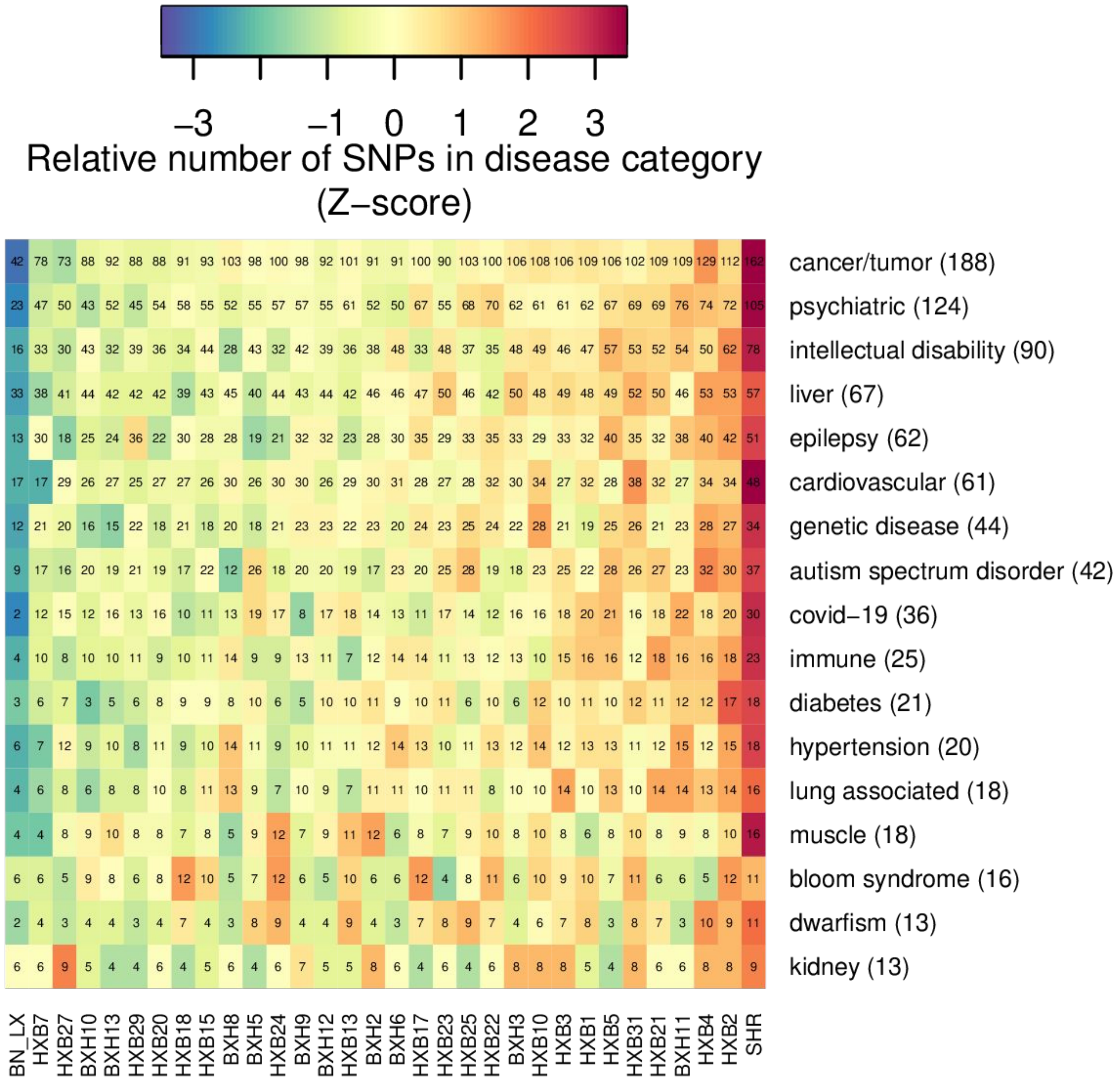

Figure S21 (related to Figure 5). Disease ontology of genetic variants found in the HXB/BXH RI panel. Numbers within the grid show the absolute number of SNPs with high impact on genes within the disease annotation. Numbers following the disease name shows the total number of annotated genes with at least 1 high impact variant within the panel.

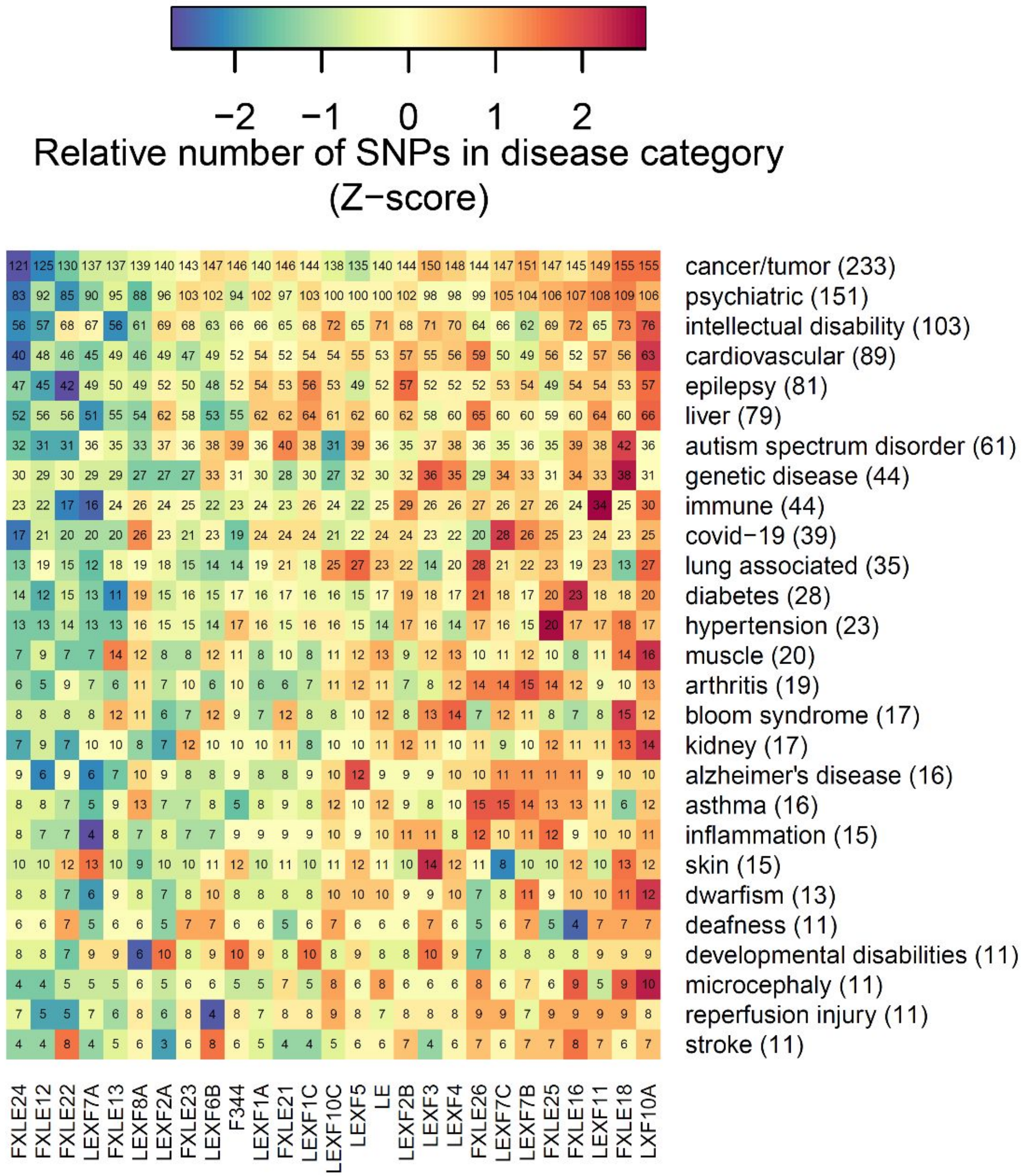

Figure S22 (related to Figure 5). Disease ontology annotation of variants in the LEXF/FXLE RI panel. Numbers within the grid show the absolute number of SNPs with high impact on genes within the disease annotation. Numbers following the disease name shows the total number of annotated genes with at least 1 high impact variant within the panel.

Supplemental references in Excel files

[1] L. Wu, W. Shi, J. Long, X. Guo, K. Michailidou, J. Beesley, MK. Bolla, XO. Shu, Y. Lu, Q. Cai, F. Al-Ejeh, E. Rozali, Q. Wang, J. Dennis, B. Li, C. Zeng, H. Feng, A. Gusev, RT. Barfield, IL. Andrulis, H. Anton-Culver, V. Arndt, KJ. Aronson, PL. Auer, M. Barrdahl, C. Baynes, MW. Beckmann, J. Benitez, M. Bermisheva, C. Blomqvist, NV. Bogdanova, SE. Bojesen, H. Brauch, H. Brenner, L. Brinton, P. Broberg, SY. Brucker, B. Burwinkel, T. Caldés, F. Canzian, BD. Carter, JE. Castelao, J. Chang-Claude, X. Chen, TD. Cheng, H. Christiansen, CL. Clarke, M. Collée, S. Cornelissen, FJ. Couch, D. Cox, A. Cox, SS. Cross, JM. Cunningham, K. Czene, MB. Daly, P. Devilee, KF. Doheny, T. Dörk, I. Dos-Santos-Silva, M. Dumont, M. Dwek, DM. Eccles, U. Eilber, AH. Eliassen, C. Engel, M. Eriksson, L. Fachal, PA. Fasching, J. Figueroa, D. Flesch-Janys, O. Fletcher, H. Flyger, L. Fritschi, M. Gabrielson, M. Gago-Dominguez, SM. Gapstur, M. García-Closas, MM. Gaudet, M. Ghoussaini, GG. Giles, MS. Goldberg, DE. Goldgar, A. González-Neira, P. Guénel, E. Hahnen, CA. Haiman, N. Håkansson, P. Hall, E. Hallberg, U. Hamann, P. Harrington, A. Hein, B. Hicks, P. Hillemanns, A. Hollestelle, RN. Hoover, JL. Hopper, G. Huang, K. Humphreys, DJ. Hunter, A. Jakubowska, W. Janni, EM. John, N. Johnson, K. Jones, ME. Jones, A. Jung, R. Kaaks, MJ. Kerin, E. Khusnutdinova, VM. Kosma, VN. Kristensen, D. Lambrechts, L. Le Marchand, J. Li, S. Lindström, J. Lissowska, WY. Lo, S. Loibl, J. Lubinski, C. Luccarini, MP. Lux, RJ. MacInnis, T. Maishman, IM. Kostovska, A. Mannermaa, JE. Manson, S. Margolin, D. Mavroudis, H. Meijers-Heijboer, A. Meindl, U. Menon, J. Meyer, AM. Mulligan, SL. Neuhausen, H. Nevanlinna, P. Neven, SF. Nielsen, BG. Nordestgaard, OI. Olopade, JE. Olson, H. Olsson, P. Peterlongo, J. Peto, D. Plaseska-Karanfilska, R. Prentice, N. Presneau, K. Pylkäs, B. Rack, P. Radice, N. Rahman, G. Rennert, HS. Rennert, V. Rhenius, A. Romero, J. Romm, A. Rudolph, E. Saloustros, DP. Sandler, EJ. Sawyer, MK. Schmidt, RK. Schmutzler, A. Schneeweiss, RJ. Scott, CG. Scott, S. Seal, M. Shah, MJ. Shrubsole, A. Smeets, MC. Southey, JJ. Spinelli, J. Stone, H. Surowy, AJ. Swerdlow, RM. Tamimi, W. Tapper, JA. Taylor, MB. Terry, DC. Tessier, A. Thomas, K. Thöne, RAEM. Tollenaar, D. Torres, T. Truong, M. Untch, C. Vachon, D. Van Den Berg, D. Vincent, Q. Waisfisz, CR. Weinberg, C. Wendt, AS. Whittemore, H. Wildiers, WC. Willett, R. Winqvist, A. Wolk, L. Xia, XR. Yang, A. Ziogas, E. Ziv, AM. Dunning, PDP. Pharoah, J. Simard, RL. Milne, SL. Edwards, P. Kraft, DF. Easton, G. Chenevix-Trench, and W. Zheng. A transcriptome-wide association study of 229,000 women identifies new candidate susceptibility genes for breast cancer. Nat Genet, 50(7):968–978, 2018. PMCID: PMC6314198, PMID: 29915430.

[2] PA. Northcott, Y. Nakahara, X. Wu, L. Feuk, DW. Ellison, S. Croul, S. Mack, PN. Kongkham, J. Peacock, A. Dubuc, YS. Ra, K. Zilberberg, J. McLeod, SW. Scherer, J. Sunil Rao, CG. Eberhart, W. Grajkowska, Y. Gillespie, B. Lach, R. Grundy, IF. Pollack, RL. Hamilton, T. Van Meter, CG. Carlotti, F. Boop, D. Bigner, RJ. Gilbertson, JT. Rutka, and MD. Taylor. Multiple recurrent genetic events converge on control of histone lysine methylation in medulloblastoma. Nat Genet, 41(4):465–472, 2009. PMCID: PMC4454371, PMID: 19270706.

[3] H. Guo, MH. Duyzend, BP. Coe, C. Baker, K. Hoekzema, J. Gerdts, TN. Turner, MC. Zody, JS. Beighley, SC. Murali, BJ. Nelson, MJ. Bamshad, DA. Nickerson, RA. Bernier, and EE. Eichler. Genome sequencing identifies multiple deleterious variants in autism

patients with more severe phenotypes. Genet Med, 21(7):1611–1620, 2019. PMCID: PMC6546556, PMID: 30504930.

[4] EA. Stahl, G. Breen, AJ. Forstner, A. McQuillin, S. Ripke, V. Trubetskoy, M. Mattheisen, Y. Wang, JRI. Coleman, HA. Gaspar, CA. de Leeuw, S. Steinberg, JMW. Pavlides, M. Trzaskowski, EM. Byrne, TH. Pers, PA. Holmans, AL. Richards, L. Abbott, E. Agerbo, H. Akil, D. Albani, N. Alliey-Rodriguez, TD. Als, A. Anjorin, V. Antilla, S. Awasthi, JA. Badner, M. Bækvad-Hansen, JD. Barchas, N. Bass, M. Bauer, R. Belliveau, SE. Bergen, CB. Pedersen, E. Bøen, MP. Boks, J. Boocock, M. Budde, W. Bunney, M. Burmeister, J. Bybjerg-Grauholm, W. Byerley, M. Casas, F. Cerrato, P. Cervantes, K. Chambert, AW. Charney, D. Chen, C. Churchhouse, TK. Clarke, W. Coryell, DW. Craig, C. Cruceanu, D. Curtis, PM. Czerski, AM. Dale, S. de Jong, F. Degenhardt, J. Del-Favero, JR. DePaulo, S. Djurovic, AL. Dobbyn, A. Dumont, T. Elvsåshagen, V. Escott-Price, CC. Fan, SB. Fischer, M. Flickinger, TM. Foroud, L. Forty, J. Frank, C. Fraser, NB. Freimer, L. Frisén, K. Gade, D. Gage, J. Garnham, C. Giambartolomei, MG. Pedersen, J. Goldstein, SD. Gordon, K. Gordon-Smith, EK. Green, MJ. Green, TA. Greenwood, J. Grove, W. Guan, J. Guzman-Parra, ML. Hamshere, M. Hautzinger, U. Heilbronner, S. Herms, M. Hipolito, P. Hoffmann, D. Holland, L. Huckins, S. Jamain, JS. Johnson, A. Juréus, R. Kandaswamy, R. Karlsson, JL. Kennedy, S. Kittel-Schneider, JA. Knowles, M. Kogevinas, AC. Koller, R. Kupka, C. Lavebratt, J. Lawrence, WB. Lawson, M. Leber, PH. Lee, SE. Levy, JZ. Li, C. Liu, S. Lucae, A. Maaser, DJ. MacIntyre, PB. Mahon, W. Maier, L. Martinsson, S. McCarroll, P. McGuffin, MG. McInnis, JD. McKay, H. Medeiros, SE. Medland, F. Meng, L. Milani, GW. Montgomery, DW. Morris, TW. Mühleisen, N. Mullins, H. Nguyen, CM. Nievergelt, AN. Adolfsson, EA. Nwulia, C. O'Donovan, LMO. Loohuis, APS. Ori, L. Oruc, U. Ösby, RH. Perlis, A. Perry, A. Pfennig, JB. Potash, SM. Purcell, EJ. Regeer, A. Reif, CS. Reinbold, JP. Rice, F. Rivas, M. Rivera, P. Roussos, DM. Ruderfer, E. Ryu, C. Sánchez-Mora, AF. Schatzberg, WA. Scheftner, NJ. Schork, C. Shannon Weickert, T. Shehktman, PD. Shilling, E. Sigurdsson, C. Slaney, OB. Smeland, JL. Sobell, C. Søholm Hansen, AT. Spijker, D. St Clair, M. Steffens, JS. Strauss, F. Streit, J. Strohmaier, S. Szelinger, RC. Thompson, TE. Thorgeirsson, J. Treutlein, H. Vedder, W. Wang, SJ. Watson, TW. Weickert, SH. Witt, S. Xi, W. Xu, AH. Young, P. Zandi, P. Zhang, S. Zöllner, R. Adolfsson, I. Agartz, M. Alda, L. Backlund, BT. Baune, F. Bellivier, WH. Berrettini, JM. Biernacka, DHR. Blackwood, M. Boehnke, AD. Børglum, A. Corvin, N. Craddock, MJ. Daly, U. Dannlowski, T. Esko, B. Etain, M. Frye, JM. Fullerton, ES. Gershon, M. Gill, F. Goes, M. Grigoroiu-Serbanescu, J. Hauser, DM. Hougaard, CM. Hultman, I. Jones, LA. Jones, RS. Kahn, G. Kirov, M. Landén, M. Leboyer, CM. Lewis, QS. Li, J. Lissowska, NG. Martin, F. Mayoral, SL. McElroy, AM. McIntosh, FJ. McMahon, I. Melle, A. Metspalu, PB. Mitchell, G. Morken, O. Mors, PB. Mortensen, B. Müller-Myhsok, RM. Myers, BM. Neale, V. Nimgaonkar, M. Nordentoft, MM. Nöthen, MC. O'Donovan, KJ. Oedegaard, MJ. Owen, SA. Paciga, C. Pato, MT. Pato, D. Posthuma, JA. Ramos-Quiroga, M. Ribasés, M. Rietschel, GA. Rouleau, M. Schalling, PR. Schofield, TG. Schulze, A. Serretti, JW. Smoller, H. Stefansson, K. Stefansson, E. Stordal, PF. Sullivan, G. Turecki, AE. Vaaler, E. Vieta, JB. Vincent, T. Werge, JI. Nurnberger, NR. Wray, A. Di Florio, HJ. Edenberg, S. Cichon, RA. Ophoff, LJ. Scott, OA. Andreassen, J. Kelsoe, and P. Sklar. Genome-wide association study identifies 30 loci associated with bipolar disorder. Nat Genet, 51(5):793–803, 2019. PMCID: PMC6956732, PMID: 31043756.

[5] S. Richards, N. Aziz, S. Bale, D. Bick, S. Das, J. Gastier-Foster, WW. Grody, M. Hegde, E. Lyon, E. Spector, K. Voelkerding, and HL. Rehm. Standards and guidelines for the

interpretation of sequence variants: a joint consensus recommendation of the american college of medical genetics and genomics and the association for molecular pathology. Genet Med, 17(5):405–424, 2015. PMCID: PMC4544753, PMID: 25741868.

[6] K. Downes, K. Megy, D. Duarte, M. Vries, J. Gebhart, S. Hofer, O. Shamardina, SVV. Deevi, J. Stephens, R. Mapeta, S. Tuna, N. Al Hasso, MW. Besser, N. Cooper, L. Daugherty, N. Gleadall, D. Greene, M. Haimel, H. Martin, S. Papadia, S. Revel-Vilk, S. Sivapalaratnam, E. Symington, W. Thomas, C. Thys, A. Tolios, CJ. Penkett, WH. Ouwehand, S. Abbs, MA. Laffan, E. Turro, I. Simeoni, AD. Mumford, YMC. Henskens, I. Pabinger, K. Gomez, and K. Freson. Diagnostic high-throughput sequencing of 2396 patients with bleeding, thrombotic, and platelet disorders. Blood, 134(23):2082–2091, 2019. PMCID: PMC6993014, PMID: 31064749.

[7] ER. Riggs, EF. Andersen, AM. Cherry, S. Kantarci, H. Kearney, A. Patel, G. Raca, DI. Ritter, ST. South, EC. Thorland, D. Pineda-Alvarez, S. Aradhya, and CL. Martin. Technical standards for the interpretation and reporting of constitutional copy-number variants: a joint consensus recommendation of the american college of medical genetics and genomics (acmg) and the clinical genome resource (clingen). Genet Med, 22(2):245–257, 2020. PMCID: PMC7313390, PMID: 31690835.

[8] K. Nykamp, M. Anderson, M. Powers, J. Garcia, B. Herrera, YY. Ho, Y. Kobayashi, N. Patil, J. Thusberg, M. Westbrook, and S. Topper. Sherloc: a comprehensive refinement of the acmg-amp variant classification criteria. Genet Med, 19(10):1105–1117, 2017. PMCID: PMC5632818, PMID: 28492532.

[9] HM. Kearney, EC. Thorland, KK. Brown, F. Quintero-Rivera, and ST. South. American college of medical genetics standards and guidelines for interpretation and reporting of postnatal constitutional copy number variants. Genet Med, 13(7):680–685, 2011. PMID: 21681106.

[10] M. Assoum, C. Philippe, B. Isidor, L. Perrin, P. Makrythanasis, N. Sondheimer, C. Paris, J. Douglas, G. Lesca, S. Antonarakis, H. Hamamy, T. Jouan, Y. Duffourd, S. Auvin, A. Saunier, A. Begtrup, C. Nowak, N. Chatron, D. Ville, K. Mireskandari, P. Milani, P. Jonveaux, G. Lemeur, M. Milh, M. Amamoto, M. Kato, M. Nakashima, N. Miyake, N. Matsumoto, A. Masri, C. Thauvin-Robinet, JB. Rivière, L. Faivre, and J. Thevenon. Autosomal-recessive mutations in ap3b2, adaptor-related protein complex 3 beta 2 subunit, cause an early-onset epileptic encephalopathy with optic atrophy. Am J Hum Genet, 99(6):1368–1376, 2016. PMCID: PMC5142104, PMID: 27889060.

[11] Y. Xiong, Y. Liu, L. Cao, D. Wang, M. Guo, A. Jiang, D. Guo, W. Hu, J. Yang, Z. Tang, H. Wu, Y. Lin, M. Zhang, Q. Zhang, M. Shi, Y. Liu, Y. Zhou, K. Lan, and Y. Chen. Transcriptomic characteristics of bronchoalveolar lavage fluid and peripheral blood mononuclear cells in covid-19 patients. Emerg Microbes Infect, 9(1):761–770, 2020. PMCID: PMC7170362, PMID: 32228226.

[12] CA. Moore, CA. Parkin, Y. Bidet, and PW. Ingham. A role for the myoblast city homologues dock1 and dock5 and the adaptor proteins crk and crk-like in zebrafish myoblast fusion. Development, 134(17):3145–3153, 2007. PMID: 17670792.

[13] JF. Côté and K. Vuori. Gef what? dock180 and related proteins help rac to polarize cells in

new ways. Trends Cell Biol, 17(8):383–393, 2007. PMCID: PMC2887429, PMID: 17765544.

[14] KV. Pajcini, JH. Pomerantz, O. Alkan, R. Doyonnas, and HM. Blau. Myoblasts and macrophages share molecular components that contribute to cell-cell fusion. J Cell Biol, 180(5):1005–1019, 2008. PMCID: PMC2265408, PMID: 18332221.

[15] S. Kumari, V. Borroni, A. Chaudhry, B. Chanda, R. Massol, S. Mayor, and FJ. Barrantes. Nicotinic acetylcholine receptor is internalized via a rac-dependent, dynamin-independent endocytic pathway. J Cell Biol, 181(7): 1179–1193, 2008. PMCID: PMC2442195, PMID: 18591431.

[16] M. Laurin, N. Fradet, A. Blangy, A. Hall, K. Vuori, and JF. Côté. The atypical rac activator dock180 (dock1) regulates myoblast fusion in vivo. Proc Natl Acad Sci U S A, 105(40):15446–15451, 2008. PMCID: PMC2563090, PMID: 18820033.

[17] F. Sanematsu, M. Hirashima, M. Laurin, R. Takii, A. Nishikimi, K. Kitajima, G. Ding, M. Noda, Y. Murata, Y. Tanaka, S. Masuko, T. Suda, C. Meno, JF. Côté, T. Nagasawa, and Y. Fukui. Dock180 is a rac activator that regulates cardiovascular development by acting downstream of cxcr4. Circ Res, 107(9):1102–1105, 2010. PMCID: PMC4640903, PMID: 20829512.

[18] G. Gadea and A. Blangy. Dock-family exchange factors in cell migration and disease. Eur J Cell Biol, 93(10-12): 466–477, 2014. PMID: 25022758.

[19] PT. Hallock, S. Chin, S. Blais, TA. Neubert, and DJ. Glass. Sorbs1 and -2 interact with crkl and are required for acetylcholine receptor cluster formation. Mol Cell Biol, 36(2):262–270, 2016. PMCID: PMC4719301, PMID: 26527617.

[20] V. Ubba, UK. Soni, S. Chadchan, VK. Maurya, V. Kumar, R. Maurya, H. Chaturvedi, R. Singh, A. Dwivedi, and RK. Jha. Rhog-dock1-rac1 signaling axis is perturbed in dhea-induced polycystic ovary in rat model. Reprod Sci, 24(5):738–752, 2017. PMID: 27662902.

[21] A. Entwistle, RJ. Zalin, AE. Warner, and S. Bevan. A role for acetylcholine receptors in the fusion of chick myoblasts. J Cell Biol, 106(5):1703–1712, 1988. PMCID: PMC2115056, PMID: 3372592.

[22] T. Drgon, PW. Zhang, C. Johnson, D. Walther, J. Hess, M. Nino, and GR. Uhl. Genome wide association for addiction: replicated results and comparisons of two analytic approaches. PLoS One, 5(1):e8832, 2010. PMCID: PMC2809089, PMID: 20098672.

[23] O. Baspinar, M. Kilinc, A. Balat, MA. Celkan, and Y. Coskun. Long tortuous aorta in a child with larsen syndrome. Can J Cardiol, 21(3):299–301, 2005. PMID: 15776121.

[24] M. Renard, T. Holm, R. Veith, BL. Callewaert, LC. Adès, O. Baspinar, A. Pickart, M. Dasouki, J. Hoyer, A. Rauch, P. Trapane, MG. Earing, PJ. Coucke, LY. Sakai, HC. Dietz, AM. De Paepe, and BL. Loeys. Altered tgfbeta signaling and cardiovascular manifestations in patients with autosomal recessive cutis laxa type i caused by fibulin-4 deficiency. Eur J Hum Genet, 18(8):895–901, 2010. PMCID: PMC2987390, PMID: 20389311.

[25] V. Mathieu, C. Pirker, WM. Schmidt, S. Spiegl-Kreinecker, D. Lötsch, P. Heffeter, B. Hegedus, M. Grusch, R. Kiss, and W. Berger. Aggressiveness of human melanoma xenograft models is promoted by aneuploidy-driven gene expression deregulation. Oncotarget, 3(4):399–413, 2012. PMCID: PMC3380575, PMID: 22535842.

[26] SK. Lee, MS. Park, and MJ. Nam. Aspirin has antitumor effects via expression of calpain gene in cervical cancer cells. J Oncol, 2008:285374, 2008. PMCID: PMC2648633, PMID: 19266085.

[27] DB. Hoffmann, SK. Williams, J. Bojcevski, A. Müller, C. Stadelmann, V. Naidoo, BA. Bahr, R. Diem, and R. Fairless. Calcium influx and calpain activation mediate preclinical retinal neurodegeneration in autoimmune optic neuritis. J Neuropathol Exp Neurol, 72(8):745–757, 2013. PMID: 23860028.

[28] A. Wessmann, T. Goedde, A. Fischer, P. Wohlsein, H. Hamann, O. Distl, and A. Tipold. Hereditary ataxia in the jack russell terrier–clinical and genetic investigations. J Vet Intern Med, 18(4):515–521, 2004. PMID: 15320590.

[29] M. Cachin and M. Vandevelde. Congenital tremor with spongy degeneration of the central nervous system in two puppies. J Vet Intern Med, 5(2):87–90, 1991. PMID: 2061870.

[30] K. Simpson, S. Eminaga, and GB. Cherubini. Hereditary ataxia in jack russell terriers in the uk. Vet Rec, 170 (21):548, 2012. PMID: 22634896.

[31] A. Vanhaesebrouck, R. Franklin, L. Van Ham, and S. Bhatti. Hereditary ataxia, myokymia and neuromyotonia in jack russell terriers. Vet Rec, 171(5):131–132, 2012. PMID: 22872628.

[32] OP. Forman, L. De Risio, and CS. Mellersh. Missense mutation in capn1 is associated with spinocerebellar ataxia in the parson russell terrier dog breed. PLoS One, 8(5):e64627, 2013. PMCID: PMC3669408, PMID: 23741357.

[33] T. Palmer. Hereditary ataxia in jack russell terriers in the uk. Vet Rec, 174(10):258, 2014. PMID: 24736825.

[34] GB. Cherubini. Hereditary ataxia in jack russell terriers in the uk. Vet Rec, 174(10):258, 2014. PMID: 24736826.

[35] Z. Gan-Or, N. Bouslam, N. Birouk, A. Lissouba, DB. Chambers, J. Vérièpe, A. Androschuk, SB. Laurent, D. Rochefort, D. Spiegelman, A. Dionne-Laporte, A. Szuto, M. Liao, DA. Figlewicz, A. Bouhouche, A. Benomar, M. Yahyaoui, R. Ouazzani, G. Yoon, N. Dupré, O. Suchowersky, FV. Bolduc, JA. Parker, PA. Dion, P. Drapeau, GA. Rouleau, and B. Ouled Amar Bencheikh. Mutations in capn1 cause autosomal-recessive hereditary spastic paraplegia. Am J Hum Genet, 98(5):1038–1046, 2016. PMCID: PMC4863665, PMID: 27153400.

[36] Z. Gan-Or, N. Bouslam, N. Birouk, A. Lissouba, DB. Chambers, J. Vérièpe, A. Androschuk, SB. Laurent, D. Rochefort, D. Spiegelman, A. Dionne-Laporte, A. Szuto, M. Liao, DA. Figlewicz, A. Bouhouche, A. Benomar, M. Yahyaoui, R. Ouazzani, G. Yoon, N. Dupré, O. Suchowersky, FV. Bolduc, JA. Parker, PA. Dion, P. Drapeau, GA. Rouleau, and B. Ouled Amar Bencheikh. Mutations in capn1 cause autosomal-recessive hereditary

spastic paraplegia. Am J Hum Genet, 98(6):1271, 2016. PMCID: PMC4908182, PMID: 27259058.

[37] Y. Wang, J. Hersheson, D. Lopez, M. Hammer, Y. Liu, KH. Lee, V. Pinto, J. Seinfeld, S. Wiethoff, J. Sun, R. Amouri, F. Hentati, N. Baudry, J. Tran, AB. Singleton, M. Coutelier, A. Brice, G. Stevanin, A. Durr, X. Bi, H. Houlden, and M. Baudry. Defects in the capn1 gene result in alterations in cerebellar development and cerebellar ataxia in mice and humans. Cell Rep, 16(1):79–91, 2016. PMCID: PMC4927383, PMID: 27320912.

[38] TW. Lewis and CS. Mellersh. Changes in mutation frequency of eight mendelian inherited disorders in eight pedigree dog populations following introduction of a commercial dna test. PLoS One, 14(1):e0209864, 2019. PMCID: PMC6334900, PMID: 30650096.

[39] WJ. Hartley and AC. Palmer. Ataxia in jack russell terriers. Acta Neuropathol, 26(1):71–74, 1973. PMID: 4747697.

[40] N. Weinhold, A. Jacobsen, N. Schultz, C. Sander, and W. Lee. Genome-wide analysis of noncoding regulatory mutations in cancer. Nat Genet, 46(11):1160–1165, 2014. PMCID: PMC4217527, PMID: 25261935.

[41] E. Castellsagué, S. González, M. Nadal, O. Campos, E. Guinó, M. Urioste, I. Blanco, T. Frebourg, and G. Capellá. Detection of apc gene deletions using quantitative multiplex pcr of short fluorescent fragments. Clin Chem, 54(7):1132–1140, 2008. PMID: 18487285.

[42] M. De Rosa, M. Galatola, S. Borriello, F. Duraturo, S. Masone, and P. Izzo. Implication of adenomatous polyposis coli and mutyh mutations in familial colorectal polyposis. Dis Colon Rectum, 52(2):268–274, 2009. PMID: 19279422.

[43] A. Rohlin, Y. Engwall, K. Fritzell, K. Göransson, A. Bergsten, Z. Einbeigi, M. Nilbert, P. Karlsson, J. Björk, and M. Nordling. Inactivation of promoter 1b of apc causes partial gene silencing: evidence for a significant role of the promoter in regulation and causative of familial adenomatous polyposis. Oncogene, 30(50):4977–4989, 2011. PMCID: PMC3240859, PMID: 21643010.

[44] E. Turro, WJ. Astle, K. Megy, S. Gräf, D. Greene, O. Shamardina, HL. Allen, A. Sanchis-Juan, M. Frontini, C. Thys, J. Stephens, R. Mapeta, OS. Burren, K. Downes, M. Haimel, S. Tuna, SVV. Deevi, TJ. Aitman, DL. Bennett, P. Calleja, K. Carss, MJ. Caulfield, PF. Chinnery, PH. Dixon, DP. Gale, R. James, A. Koziell, MA. Laffan, AP. Levine, ER. Maher, HS. Markus, J. Morales, NW. Morrell, AD. Mumford, E. Ormondroyd, S. Rankin, A. Rendon, S. Richardson, I. Roberts, NBA. Roy, MA. Saleem, KGC. Smith, H. Stark, RYY. Tan, AC. Themistocleous, AJ. Thrasher, H. Watkins, AR. Webster, MR. Wilkins, C. Williamson, J. Whitworth, S. Humphray, DR. Bentley, N. Kingston, N. Walker, JR. Bradley, S. Ashford, CJ. Penkett, K. Freson, KE. Stirrups, FL. Raymond, and WH. Ouwehand. Whole-genome sequencing of patients with rare diseases in a national health system. Nature, 583(7814):96–9102, 2020. PMCID: PMC7610553, PMID: 32581362.

[45] J. Zhang, K. Wang, J. Zhang, SS. Liu, L. Dai, and JY. Zhang. Using proteomic approach to identify tumor-associated proteins as biomarkers in human esophageal squamous cell

carcinoma. J Proteome Res, 10 (6):2863–2872, 2011. PMCID: PMC3119842, PMID: 21517111.

[46] N. Loukili, N. Rosenblatt-Velin, J. Li, S. Clerc, P. Pacher, F. Feihl, B. Waeber, and L. Liaudet. Peroxynitrite induces hmgb1 release by cardiac cells in vitro and hmgb1 upregulation in the infarcted myocardium in vivo. Cardiovasc Res, 89(3):586–594, 2011. PMCID: PMC3028979, PMID: 21113057.

[47] SJ. Schomisch, DG. Murdock, N. Hedayati, JL. Carino, EJ. Lesnefsky, and BL. Cmolik. Cardioplegia prevents ischemia-induced transcriptional alterations of cytoprotective genes in rat hearts: a dna microarray study. J Thorac Cardiovasc Surg, 130(4):1151, 2005. PMID: 16214533.

[48] T. Nishida, M. Tsubota, Y. Kawaishi, H. Yamanishi, N. Kamitani, F. Sekiguchi, H. Ishikura, K. Liu, M. Nishibori, and A. Kawabata. Involvement of high mobility group box 1 in the development and maintenance of chemotherapy-induced peripheral neuropathy in rats. Toxicology, 365:48–58, 2016. PMID: 27474498.

[49] SY. Lin, YY. Wang, WY. Chen, SL. Liao, ST. Chou, CP. Yang, and CJ. Chen. Hepatoprotective activities of rosmarinic acid against extrahepatic cholestasis in rats. Food Chem Toxicol, 108(Pt A):214–223, 2017. PMID: 28789951.

[50] D. Tang, R. Kang, W. Xiao, H. Zhang, MT. Lotze, H. Wang, and X. Xiao. Quercetin prevents lps-induced high-mobility group box 1 release and proinflammatory function. Am J Respir Cell Mol Biol, 41(6):651–660, 2009. PMCID: PMC2784404, PMID: 19265175.

[51] ML. Xu, XJ. Yu, JQ. Zhao, Y. Du, WJ. Xia, Q. Su, MM. Du, Q. Yang, J. Qi, Y. Li, SW. Zhou, GQ. Zhu, HB. Li, and YM. Kang. Calcitriol ameliorated autonomic dysfunction and hypertension by down-regulating inflammation and oxidative stress in the paraventricular nucleus of shr. Toxicol Appl Pharmacol, 394:114950, 2020. PMID: 32147540.

[52] M. Gao, Z. Hu, Y. Zheng, Y. Zeng, X. Shen, D. Zhong, and F. He. Peroxisome proliferator-activated receptor γ agonist troglitazone inhibits high mobility group box 1 expression in endothelial cells via suppressing transcriptional activity of nuclear factor κb and activator protein 1. Shock, 36(3):228–234, 2011. PMID: 21617575.

[53] TH. Kim, SK. Ku, and JS. Bae. Inhibitory effects of kaempferol-3-o-sophoroside on hmgb1-mediated proinflammatory responses. Food Chem Toxicol, 50(3-4):1118–1123, 2012. PMID: 22178603.

[54] EJ. Yang, W. Lee, SK. Ku, KS. Song, and JS. Bae. Anti-inflammatory activities of oleanolic acid on hmgb1 activated huvecs. Food Chem Toxicol, 50(5):1288–1294, 2012. PMID: 22386814.

[55] W. Lee, SK. Ku, JW. Bae, and JS. Bae. Inhibitory effects of lycopene on hmgb1-mediated pro-inflammatory responses in both cellular and animal models. Food Chem Toxicol, 50(6):1826–1833, 2012. PMID: 22429818.

[56] W. Lee, SK. Ku, TH. Kim, and JS. Bae. Emodin-6-o-β-d-glucoside inhibits hmgb1-induced inflammatory responses in vitro and in vivo. Food Chem Toxicol, 52:97–9104, 2013.

PMID: 23146691.

[57] M. Zhao, Y. Feng, J. Xiao, J. Liang, Y. Yin, and D. Chen. Sodium tanshinone iia sulfonate prevents hypoxic trophoblast-induced endothelial cell dysfunction via targeting hmgb1 release. J Biochem Mol Toxicol, 31(7), 2017. PMID: 28294475.

[58] DJ. Antoine, DP. Williams, A. Kipar, RE. Jenkins, SL. Regan, JG. Sathish, NR. Kitteringham, and BK. Park. High-mobility group box-1 protein and keratin-18, circulating serum proteins informative of acetaminophen-induced necrosis and apoptosis in vivo. Toxicol Sci, 112(2):521–531, 2009. PMID: 19783637.

[59] W. Zhang, M. Zhang, Z. Wang, Y. Cheng, H. Liu, Z. Zhou, B. Han, B. Chen, H. Yao, and J. Chao. Neogambogic acid prevents silica-induced fibrosis via inhibition of high-mobility group box 1 and mcp-1-induced protein 1. Toxicol Appl Pharmacol, 309:129–140, 2016. PMID: 27616297.

[60] YM. Allette, MR. Due, SM. Wilson, P. Feldman, MS. Ripsch, R. Khanna, and FA. White. Identification of a functional interaction of hmgb1 with receptor for advanced glycation end-products in a model of neuropathic pain. Brain Behav Immun, 42:169–177, 2014. PMCID: PMC4560334, PMID: 25014009.

[61] I. Kushima, B. Aleksic, M. Nakatochi, T. Shimamura, T. Okada, Y. Uno, M. Morikawa, K. Ishizuka, T. Shiino, H. Kimura, Y. Arioka, A. Yoshimi, Y. Takasaki, Y. Yu, Y. Nakamura, M. Yamamoto, T. Iidaka, S. Iritani, T. Inada, N. Ogawa, E. Shishido, Y. Torii, N. Kawano, Y. Omura, T. Yoshikawa, T. Uchiyama, T. Yamamoto, M. Ikeda, R. Hashimoto, H. Yamamori, Y. Yasuda, T. Someya, Y. Watanabe, J. Egawa, A. Nunokawa, M. Itokawa, M. Arai, M. Miyashita, A. Kobori, M. Suzuki, T. Takahashi, M. Usami, M. Kodaira, K. Watanabe, T. Sasaki, H. Kuwabara, M. Tochigi, F. Nishimura, H. Yamasue, Y. Eriguchi, S. Benner, M. Kojima, W. Yassin, T. Munesue, S. Yokoyama, R. Kimura, Y. Funabiki, H. Kosaka, M. Ishitobi, T. Ohmori, S. Numata, T. Yoshikawa, T. Toyota, K. Yamakawa, T. Suzuki, Y. Inoue, K. Nakaoka, YI. Goto, M. Inagaki, N. Hashimoto, I. Kusumi, S. Son, T. Murai, T. Ikegame, N. Okada, K. Kasai, S. Kunimoto, D. Mori, N. Iwata, and N. Ozaki. Comparative analyses of copy-number variation in autism spectrum disorder and schizophrenia reveal etiological overlap and biological insights. Cell Rep, 24(11):2838–2856, 2018. PMID: 30208311.

[62] IM. Campbell, B. Yuan, C. Robberecht, R. Pfundt, P. Szafranski, ME. McEntagart, SC. Nagamani, A. Erez, M. Bartnik, B. Wiśniowiecka-Kowalnik, KS. Plunkett, AN. Pursley, SH. Kang, W. Bi, SR. Lalani, CA. Bacino, M. Vast, K. Marks, M. Patton, P. Olofsson, A. Patel, JA. Veltman, SW. Cheung, CA. Shaw, LE. Vissers, JR. Vermeesch, JR. Lupski, and P. Stankiewicz. Parental somatic mosaicism is underrecognized and influences recurrence risk of genomic disorders. Am J Hum Genet, 95(2):173–182, 2014. PMCID: PMC4129404, PMID: 25087610.

[63] EA. Otto, TW. Hurd, R. Airik, M. Chaki, W. Zhou, C. Stoetzel, SB. Patil, S. Levy, AK. Ghosh, CA. Murga-Zamalloa, J. van Reeuwijk, SJ. Letteboer, L. Sang, RH. Giles, Q. Liu, KL. Coene, A. Estrada-Cuzcano, RW. Collin, HM. McLaughlin, S. Held, JM. Kasanuki, G. Ramaswami, J. Conte, I. Lopez, J. Washburn, J. Macdonald, J. Hu, Y. Yamashita, ER. Maher, LM. Guay-Woodford, HP. Neumann, N. Obermüller, RK. Koenekoop, C. Bergmann, X. Bei, RA. Lewis, N. Katsanis, V. Lopes, DS. Williams, RH.

Lyons, CV. Dang, DA. Brito, MB. Dias, X. Zhang, JD. Cavalcoli, G. Nürnberg, P. Nürnberg, EA. Pierce, PK. Jackson, C. Antignac, S. Saunier, R. Roepman, H. Dollfus, H. Khanna, and F. Hildebrandt. Candidate exome capture identifies mutation of sdccag8 as the cause of a retinal-renal ciliopathy. Nat Genet, 42(10):840–850, 2010. PMCID: PMC2947620, PMID: 20835237.

[64] MA. Enoch, Z. Zhou, M. Kimura, DC. Mash, Q. Yuan, and D. Goldman. Gabaergic gene expression in postmortem hippocampus from alcoholics and cocaine addicts; corresponding findings in alcohol-naïve p and np rats. PLoS One, 7(1):e29369, 2012. PMCID: PMC3258238, PMID: 22253714.

[65] CI. Dixon, TW. Rosahl, and DN. Stephens. Targeted deletion of the gabra2 gene encoding alpha2-subunits of gaba(a) receptors facilitates performance of a conditioned emotional response, and abolishes anxiolytic effects of benzodiazepines and barbiturates. Pharmacol Biochem Behav, 90(1):1–8, 2008. PMID: 18313124.

[66] SH. Fatemi, TJ. Reutiman, TD. Folsom, and PD. Thuras. Gaba(a) receptor downregulation in brains of subjects with autism. J Autism Dev Disord, 39(2):223–230, 2009. PMCID: PMC2697059, PMID: 18821008.

[67] MA. Enoch, CA. Hodgkinson, Q. Yuan, PH. Shen, D. Goldman, and A. Roy. The influence of gabra2, childhood trauma, and their interaction on alcohol, heroin, and cocaine dependence. Biol Psychiatry, 67(1):20–27, 2010. PMCID: PMC2964936, PMID: 19833324.

[68] CI. Dixon, HV. Morris, G. Breen, S. Desrivieres, S. Jugurnauth, RC. Steiner, H. Vallada, C. Guindalini, R. Laranjeira, G. Messas, TW. Rosahl, JR. Atack, DR. Peden, D. Belelli, JJ. Lambert, SL. King, G. Schumann, and DN. Stephens. Cocaine effects on mouse incentive-learning and human addiction are linked to alpha2 subunit-containing gabaa receptors. Proc Natl Acad Sci U S A, 107(5):2289–2294, 2010. PMCID: PMC2836671, PMID: 20133874.

[69] N. Orenstein, H. Goldberg-Stern, R. Straussberg, L. Bazak, M. Weisz Hubshman, N. Kropach, O. Gilad, O. Scheuerman, Y. Dory, D. Kraus, S. Tzur, N. Magal, Y. Kilim, V. Shkalim Zemer, and L. Basel-Salmon. A de novo gabra2 missense mutation in severe early-onset epileptic encephalopathy with a choreiform movement disorder. Eur J Paediatr Neurol, 22(3):516–524, 2018. PMID: 29422393.

[70] M. Argos, MG. Kibriya, F. Parvez, F. Jasmine, M. Rakibuz-Zaman, and H. Ahsan. Gene expression profiles in peripheral lymphocytes by arsenic exposure and skin lesion status in a bangladeshi population. Cancer Epidemiol Biomarkers Prev, 15(7):1367–1375, 2006. PMID: 16835338.

[71] BJ. Andersen, BA. Rosa, J. Kupritz, A. Meite, T. Serge, MI. Hertz, K. Curtis, CL. King, M. Mitreva, PU. Fischer, and GJ. Weil. Systems analysis-based assessment of post-treatment adverse events in lymphatic filariasis. PLoS Negl Trop Dis, 13(9):e0007697, 2019. PMCID: PMC6762072, PMID: 31557154.

[72] W. D'Souza, S. Pradhan, and D. Saranath. Multiple single nucleotide polymorphism analysis and association of specific genotypes in fhit, samd4a, and ankrd17 in indian

patients with oral cancer. Head Neck, 39(8): 1586–1595, 2017. PMID: 28580594.

[73] RA. Spanjaard, KM. Whren, C. Graves, and J. Bhawan. Tumor necrosis factor receptor superfamily member troy is a novel melanoma biomarker and potential therapeutic target. Int J Cancer, 120(6):1304–1310, 2007. PMID: 17187358.

[74] JX. Bei, Y. Li, WH. Jia, BJ. Feng, G. Zhou, LZ. Chen, QS. Feng, HQ. Low, H. Zhang, F. He, ES. Tai, T. Kang, ET. Liu, J. Liu, and YX. Zeng. A genome-wide association study of nasopharyngeal carcinoma identifies three new susceptibility loci. Nat Genet, 42(7):599–603, 2010. PMID: 20512145.

[75] M. Iyoda, KL. Hudkins, TA. Wietecha, MC. Banas, S. Guo, G. Liu, L. Wang, J. Kowalewska, and CE. Alpers. All-trans-retinoic acid aggravates cryoglobulin-associated membranoproliferative glomerulonephritis in mice. Nephrol Dial Transplant, 22(12):3451–3461, 2007. PMID: 17686814.

[76] DG. Torgerson, EJ. Ampleford, GY. Chiu, WJ. Gauderman, CR. Gignoux, PE. Graves, BE. Himes, AM. Levin, RA. Mathias, DB. Hancock, JW. Baurley, C. Eng, DA. Stern, JC. Celedón, N. Rafaels, D. Capurso, DV. Conti, LA. Roth, M. Soto-Quiros, A. Togias, X. Li, RA. Myers, I. Romieu, DJ. Van Den Berg, D. Hu, NN. Hansel, RD. Hernandez, E. Israel, MT. Salam, J. Galanter, PC. Avila, L. Avila, JR. Rodriquez-Santana, R. Chapela, W. Rodriguez-Cintron, GB. Diette, NF. Adkinson, RA. Abel, KD. Ross, M. Shi, MU. Faruque, GM. Dunston, HR. Watson, VJ. Mantese, SC. Ezurum, L. Liang, I. Ruczinski, JG. Ford, S. Huntsman, KF. Chung, H. Vora, X. Li, WJ. Calhoun, M. Castro, JJ. Sienra-Monge, B. del Rio-Navarro, KA. Deichmann, A. Heinzmann, SE. Wenzel, WW. Busse, JE. Gern, RF. Lemanske, TH. Beaty, ER. Bleecker, BA. Raby, DA. Meyers, SJ. London, FD. Gilliland, EG. Burchard, FD. Martinez, ST. Weiss, LK. Williams, KC. Barnes, C. Ober, and DL. Nicolae. Meta-analysis of genome-wide association studies of asthma in ethnically diverse north american populations. Nat Genet, 43(9):887–892, 2011. PMCID: PMC3445408, PMID: 21804549.

[77] M. Li, P. Hener, Z. Zhang, S. Kato, D. Metzger, and P. Chambon. Topical vitamin d3 and low-calcemic analogs induce thymic stromal lymphopoietin in mouse keratinocytes and trigger an atopic dermatitis. Proc Natl Acad Sci U S A, 103(31):11736–11741, 2006. PMCID: PMC1544239, PMID: 16880407.

[78] Z. Zhu, MH. Oh, J. Yu, YJ. Liu, and T. Zheng. The role of tslp in il-13-induced atopic march. Sci Rep, 1:23, 2011. PMCID: PMC3251897, PMID: 22355542.

[79] CS. Hau, N. Kanda, and S. Watanabe. Suppressive effects of antimycotics on thymic stromal lymphopoietin production in human keratinocytes. J Dermatol Sci, 71(3):174–183, 2013. PMID: 23688403.

[80] T. Hidaka, E. Ogawa, EH. Kobayashi, T. Suzuki, R. Funayama, T. Nagashima, T. Fujimura, S. Aiba, K. Nakayama, R. Okuyama, and M. Yamamoto. The aryl hydrocarbon receptor ahr links atopic dermatitis and air pollution via induction of the neurotrophic factor artemin. Nat Immunol, 18(1):64–73, 2017. PMID: 27869817.

[81] B. Chen, W. Wei, L. Ma, B. Yang, RM. Gill, MS. Chua, AJ. Butte, and S. So. Computational discovery of niclosamide ethanolamine, a repurposed drug candidate that reduces

growth of hepatocellular carcinoma cells in vitro and in mice by inhibiting cell division cycle 37 signaling. Gastroenterology, 152(8):2022–2036, 2017. PMCID: PMC5447464, PMID: 28284560.

[82] ME. Rothenberg, JM. Spergel, JD. Sherrill, K. Annaiah, LJ. Martin, A. Cianferoni, L. Gober, C. Kim, J. Glessner, E. Frackelton, K. Thomas, C. Blanchard, C. Liacouras, R. Verma, S. Aceves, MH. Collins, T. Brown-Whitehorn, PE. Putnam, JP. Franciosi, RM. Chiavacci, SF. Grant, JP. Abonia, PM. Sleiman, and H. Hakonarson. Common variants at 5q22 associate with pediatric eosinophilic esophagitis. Nat Genet, 42(4):289–291, 2010. PMCID: PMC3740732, PMID: 20208534.

[83] Y. Wang, Y. Le, W. Zhao, Y. Lin, Y. Wu, C. Yu, J. Xiong, F. Zou, H. Dong, S. Cai, and H. Zhao. Short thymic stromal lymphopoietin attenuates toluene diisocyanate-induced airway inflammation and inhibits high mobility group box 1-receptor for advanced glycation end products and long thymic stromal lymphopoietin expression. Toxicol Sci, 157(2):276–290, 2017. PMID: 28329851.

[84] DF. Gudbjartsson, US. Bjornsdottir, E. Halapi, A. Helgadottir, P. Sulem, GM. Jonsdottir, G. Thorleifsson, H. Helgadottir, V. Steinthorsdottir, H. Stefansson, C. Williams, J. Hui, J. Beilby, NM. Warrington, A. James, LJ. Palmer, GH. Koppelman, A. Heinzmann, M. Krueger, HM. Boezen, A. Wheatley, J. Altmuller, HD. Shin, ST. Uh, HS. Cheong, B. Jonsdottir, D. Gislason, CS. Park, LM. Rasmussen, C. Porsbjerg, JW. Hansen, V. Backer, T. Werge, C. Janson, UB. Jönsson, MC. Ng, J. Chan, WY. So, R. Ma, SH. Shah, CB. Granger, AA. Quyyumi, AI. Levey, V. Vaccarino, MP. Reilly, DJ. Rader, MJ. Williams, AM. van Rij, GT. Jones, E. Trabetti, G. Malerba, PF. Pignatti, A. Boner, L. Pescollderungg, D. Girelli, O. Olivieri, N. Martinelli, BR. Ludviksson, D. Ludviksdottir, GI. Eyjolfsson, D. Arnar, G. Thorgeirsson, K. Deichmann, PJ. Thompson, M. Wjst, IP. Hall, DS. Postma, T. Gislason, J. Gulcher, A. Kong, I. Jonsdottir, U. Thorsteinsdottir, and K. Stefansson. Sequence variants affecting eosinophil numbers associate with asthma and myocardial infarction. Nat Genet, 41(3):342–347, 2009. PMID: 19198610.

[85] X. Shen, H. Ying, Y. Qiu, JS. Park, R. Shyam, ZL. Chi, T. Iwata, and BY. Yue. Processing of optineurin in neuronal cells. J Biol Chem, 286(5):3618–3629, 2011. PMCID: PMC3030366, PMID: 21059646.

[86] S. Monemi, G. Spaeth, A. DaSilva, S. Popinchalk, E. Ilitchev, J. Liebmann, R. Ritch, E. Héon, RP. Crick, A. Child, and M. Sarfarazi. Identification of a novel adult-onset primary open-angle glaucoma (poag) gene on 5q22.1. Hum Mol Genet, 14(6):725–733, 2005. PMID: 15677485.

[87] F. Pasutto, CY. Mardin, K. Michels-Rautenstrauss, BH. Weber, H. Sticht, G. Chavarria-Soley, B. Rautenstrauss, F. Kruse, and A. Reis. Profiling of wdr36 missense variants in german patients with glaucoma. Invest Ophthalmol Vis Sci, 49(1):270–274, 2008. PMID: 18172102.

[88] TK. Footz, JL. Johnson, S. Dubois, N. Boivin, V. Raymond, and MA. Walter. Glaucoma-associated wdr36 variants encode functional defects in a yeast model system. Hum Mol Genet, 18(7):1276–1287, 2009. PMID: 19150991.

[89] JS. Berg, M. Adams, N. Nassar, C. Bizon, K. Lee, CP. Schmitt, KC. Wilhelmsen, and JP.

Evans. An informatics approach to analyzing the incidentalome. Genet Med, 15(1):36–44, 2013. PMCID: PMC3538953, PMID: 22995991.

[90] Y. Freudenberg-Hua, J. Freudenberg, V. Vacic, A. Abhyankar, AK. Emde, D. Ben-Avraham, N. Barzilai, D. Oschwald, E. Christen, J. Koppel, B. Greenwald, RB. Darnell, S. Germer, G. Atzmon, and P. Davies. Disease variants in genomes of 44 centenarians. Mol Genet Genomic Med, 2(5):438–450, 2014. PMCID: PMC4190879, PMID: 25333069.

[91] J. Meletiadis, S. Chanock, and TJ. Walsh. Defining targets for investigating the pharmacogenomics of adverse drug reactions to antifungal agents. Pharmacogenomics, 9(5):561–584, 2008. PMID: 18466103.

[92] MD. AbdulHameed, GJ. Tawa, K. Kumar, DL. Ippolito, JA. Lewis, JD. Stallings, and A. Wallqvist. Systems level analysis and identification of pathways and networks associated with liver fibrosis. PLoS One, 9(11):e112193, 2014. PMCID: PMC4224449, PMID: 25380136.

[93] J. Rae, D. Noack, PG. Heyworth, BA. Ellis, JT. Curnutte, and AR. Cross. Molecular analysis of 9 new families with chronic granulomatous disease caused by mutations in cyba, the gene encoding p22(phox). Blood, 96(3): 1106–1112, 2000. PMID: 10910929.

[94] D. Roos, DB. Kuhns, A. Maddalena, J. Bustamante, C. Kannengiesser, M. de Boer, K. van Leeuwen, MY. Köker, B. Wolach, J. Roesler, HL. Malech, SM. Holland, JI. Gallin, and MJ. Stasia. Hematologically important mutations: the autosomal recessive forms of chronic granulomatous disease (second update). Blood Cells Mol Dis, 44(4):291–299, 2010. PMCID: PMC4568122, PMID: 20167518.

[95] MP. Adam, J. Feldman, GM. Mirzaa, RA. Pagon, SE. Wallace, LJH. Bean, KW. Gripp, A. Amemiya, JW. Leiding, and SM. Holland. 1993. PMCID: PMC2846862, PMID: 22876374.

[96] CF. Yang, FJ. Tsai, SP. Lin, CC. Lee, and JY. Wu. A novel in-frame deletion mutation (c106-111del) identified in a taiwan chinese patient with type iva mucopolysaccharidosis. Hum Mutat, 18(3):254, 2001. PMID: 11524742.

[97] S. Tomatsu, AM. Montaño, T. Nishioka, MA. Gutierrez, OM. Peña, GG. Tranda Firescu, P. Lopez, S. Yamaguchi, A. Noguchi, and T. Orii. Mutation and polymorphism spectrum of the galns gene in mucopolysaccharidosis iva (morquio a). Hum Mutat, 26(6):500–512, 2005. PMID: 16287098.

[98] Z. Wang, W. Zhang, Y. Wang, Y. Meng, L. Su, H. Shi, and S. Huang. Mucopolysaccharidosis iva mutations in chinese patients: 16 novel mutations. J Hum Genet, 55(8):534–540, 2010. PMID: 20574428.

[99] LM. Pollard, JR. Jones, and TC. Wood. Molecular characterization of 355 mucopolysaccharidosis patients reveals 104 novel mutations. J Inherit Metab Dis, 36(2):179–187, 2013. PMID: 22976768.

[100] VC. Dũng, S. Tomatsu, AM. Montaño, G. Gottesman, MB. Bober, W. Mackenzie, M. Maeda, GA. Mitchell, Y. Suzuki, and T. Orii. Mucopolysaccharidosis iva: correlation between genotype, phenotype and keratan sulfate levels. Mol Genet Metab, 110(1-

2):129–138, 2013. PMCID: PMC3779837, PMID: 23876334.

[101] D. He, Y. Huang, Z. Ou, H. Sheng, S. Li, X. Zhao, R. Li, J. Zheng, and L. Liu. Molecular genetic assay of mucopolysaccharidosis iva in south china. Gene, 532(1):46–52, 2013. PMID: 24035930.

[102] J. Ye, HL. Lei, HW. Zhang, WJ. Qiu, LS. Han, Y. Wang, XY. Li, and XF. Gu. [analysis of galns gene mutation in thirty-eight chinese patients with mucopolysaccharidosis type iva]. Zhonghua Er Ke Za Zhi, 51(6):414–419, 2013. PMID: 24120057.

[103] A. Morrone, KL. Tylee, M. Al-Sayed, AC. Brusius-Facchin, A. Caciotti, HJ. Church, MJ. Coll, K. Davidson, MJ. Fietz, L. Gort, M. Hegde, F. Kubaski, L. Lacerda, F. Laranjeira, S. Leistner-Segal, S. Mooney, S. Pajares, L. Pollard, I. Ribeiro, RY. Wang, and N. Miller. Molecular testing of 163 patients with morquio a (mucopolysaccharidosis iva) identifies 39 novel galns mutations. Mol Genet Metab, 112(2):160–170, 2014. PMCID: PMC4203673, PMID: 24726177.

[104] A. Morrone, A. Caciotti, R. Atwood, K. Davidson, C. Du, P. Francis-Lyon, P. Harmatz, M. Mealiffe, S. Mooney, TR. Oron, A. Ryles, KA. Zawadzki, and N. Miller. Morquio a syndrome-associated mutations: a review of alterations in the galns gene and a new locus-specific database. Hum Mutat, 35(11):1271–1279, 2014. PMCID: PMC4238747, PMID: 25137622.

[105] AM. Bidchol, A. Dalal, H. Shah, S. S, S. Nampoothiri, M. Kabra, N. Gupta, S. Danda, K. Gowrishankar, SR. Phadke, S. Kapoor, M. Kamate, IC. Verma, RD. Puri, VH. Sankar, AR. Devi, SJ. Patil, P. Ranganath, SJ. Jain, M. Agarwal, A. Singh, P. Mishra, PM. Tamhankar, PM. Gopinath, HA. Nagarajaram, K. Satyamoorthy, and KM. Girisha. Galns mutations in indian patients with mucopolysaccharidosis iva. Am J Med Genet A, (11): 2793–2801, 2014. PMID: 25252036.

[106] A. Caciotti, R. Tonin, M. Rigoldi, L. Ferri, S. Catarzi, C. Cavicchi, E. Procopio, MA. Donati, A. Ficcadenti, A. Fiumara, R. Barone, L. Garavelli, MD. Rocco, M. Filocamo, D. Antuzzi, M. Scarpa, SD. Mooney, B. Li, A. Skouma, S. Bianca, D. Concolino, R. Casalone, E. Monti, M. Pantaleo, S. Giglio, R. Guerrini, R. Parini, and A. Morrone. Optimizing the molecular diagnosis of galns: novel methods to define and characterize morquio-a syndrome-associated mutations. Hum Mutat, 36(3):357–368, 2015. PMID: 25545067.

[107] J. Xie, J. Pan, D. Guo, W. Pan, R. Li, C. Guo, M. Du, W. Jiang, and Y. Guo. Mutation analysis and pathogenicity identification of mucopolysaccharidosis type iva in 8 south china families. Gene, 686:261–269, 2019. PMID: 30458289.

[108] HY. Leong, NA. Abdul Azize, HB. Chew, WT. Keng, MK. Thong, MKN. Mohd Khalid, LC. Hung, N. Mohamed Zainudin, A. Ramlee, MA. Md Haniffa, Y. Yakob, and LH. Ngu. Clinical, biochemical and genetic profiles of patients with mucopolysaccharidosis type iva (morquio a syndrome) in malaysia: the first national natural history cohort study. Orphanet J Rare Dis, 14(1):143, 2019. PMCID: PMC6570902, PMID: 31200731.

[109] YH. Chien, NC. Lee, PW. Chen, HY. Yeh, MH. Gelb, PC. Chiu, SY. Chu, CH. Lee, AR. Lee, and WL. Hwu. Newborn screening for morquio disease and other lysosomal storage diseases: results from the 8-plex assay for 70,000 newborns. Orphanet J Rare

Dis, 15(1):38, 2020. PMCID: PMC6998831, PMID: 32014045.

[110] A. Zanetti, F. D'Avanzo, M. AlSayed, AC. Brusius-Facchin, YH. Chien, R. Giugliani, E. Izzo, DC. Kasper, HY. Lin, SP. Lin, L. Pollard, A. Singh, R. Tonin, T. Wood, A. Morrone, and R. Tomanin. Molecular basis of mucopolysaccharidosis iva (morquio a syndrome): A review and classification of galns gene variants and reporting of 68 novel variants. Hum Mutat, 42(11):1384–1398, 2021. PMCID: PMC9291100, PMID: 34387910.

[111] T. Ogawa, S. Tomatsu, S. Fukuda, A. Yamagishi, GM. Rezvi, K. Sukegawa, N. Kondo, Y. Suzuki, N. Shimozawa, and T. Orü. Mucopolysaccharidosis iva: screening and identification of mutations of the n-acetylgalactosamine-6-sulfate sulfatase gene. Hum Mol Genet, 4(3):341–349, 1995. PMID: 7795586.

[112] J. Mateu, M. Alzamora, M. Franco, and MJ. Buisán. Ifosfamide extravasation. Ann Pharmacother, 28(11): 1243–1244, 1994. PMID: 7849337.

[113] JD. Smith, AV. Hing, CM. Clarke, NM. Johnson, FA. Perez, SS. Park, JA. Horst, B. Mecham, L. Maves, DA. Nickerson, and ML. Cunningham. Exome sequencing identifies a recurrent de novo zswim6 mutation associated with acromelic frontonasal dysostosis. Am J Hum Genet, 95(2):235–240, 2014. PMCID: PMC4129399, PMID: 25105228.

[114] SR. Twigg, LB. Ousager, KA. Miller, Y. Zhou, SC. Elalaoui, A. Sefiani, GS. Bak, H. Hove, LK. Hansen, CR. Fagerberg, M. Tajir, and AO. Wilkie. Acromelic frontonasal dysostosis and zswim6 mutation: phenotypic spectrum and mosaicism. Clin Genet, 90(3):270–275, 2016. PMCID: PMC5025718, PMID: 26706854.

[115] J. Zhang, FE. Frerman, and JJ. Kim. Structure of electron transfer flavoprotein-ubiquinone oxidoreductase and electron transfer to the mitochondrial ubiquinone pool. Proc Natl Acad Sci U S A, 103(44):16212–16217, 2006. PMCID: PMC1637562, PMID: 17050691.

[116] SI. Goodman, RJ. Binard, MR. Woontner, and FE. Frerman. Glutaric acidemia type ii: gene structure and mutations of the electron transfer flavoprotein:ubiquinone oxidoreductase (etf:qo) gene. Mol Genet Metab, 77 (1-2):86–90, 2002. PMID: 12359134.

[117] RK. Olsen, M. Pourfarzam, AA. Morris, RC. Dias, I. Knudsen, BS. Andresen, N. Gregersen, and SE. Olpin. Lipid-storage myopathy and respiratory insufficiency due to etfqo mutations in a patient with late-onset multiple acyl-coa dehydrogenation deficiency. J Inherit Metab Dis, 27(5):671–678, 2004. PMID: 15669683.

[118] N. Cornelius, FE. Frerman, TJ. Corydon, J. Palmfeldt, P. Bross, N. Gregersen, and RK. Olsen. Molecular mechanisms of riboflavin responsiveness in patients with etf-qo variations and multiple acyl-coa dehydrogenation deficiency. Hum Mol Genet, 21(15):3435–3448, 2012. PMID: 22611163.

[119] MP. Marrades, P. González-Muniesa, D. Arteta, JA. Martínez, and MJ. Moreno-Aliaga. Orchestrated downregulation of genes involved in oxidative metabolic pathways in obese vs. lean high-fat young male consumers. J Physiol Biochem, 67(1):15–26, 2011. PMID: 20882379.

[120] EB. Kaminsky, V. Kaul, J. Paschall, DM. Church, B. Bunke, D. Kunig, D. Moreno-De-Luca, A. Moreno-De-Luca, JG. Mulle, ST. Warren, G. Richard, JG. Compton, AE. Fuller, TJ.

Gliem, S. Huang, MN. Collinson, SJ. Beal, T. Ackley, DL. Pickering, DM. Golden, E. Aston, H. Whitby, S. Shetty, MR. Rossi, MK. Rudd, ST. South, AR. Brothman, WG. Sanger, RK. Iyer, JA. Crolla, EC. Thorland, S. Aradhya, DH. Ledbetter, and CL. Martin. An evidence-based approach to establish the functional and clinical significance of copy number variants in intellectual and developmental disabilities. Genet Med, 13(9):777–784, 2011. PMCID: PMC3661946, PMID: 21844811.

[121] D. Moreno-De-Luca, SJ. Sanders, AJ. Willsey, JG. Mulle, JK. Lowe, DH. Geschwind, MW. State, CL. Martin, and DH. Ledbetter. Using large clinical data sets to infer pathogenicity for rare copy number variants in autism cohorts. Mol Psychiatry, 18(10):1090–1095, 2013. PMCID: PMC3720840, PMID: 23044707.

[122] VM. Leppa, SN. Kravitz, CL. Martin, J. Andrieux, C. Le Caignec, D. Martin-Coignard, C. DyBuncio, SJ. Sanders, JK. Lowe, RM. Cantor, and DH. Geschwind. Rare inherited and de novo cnvs reveal complex contributions to asd risk in multiplex families. Am J Hum Genet, 99(3):540–554, 2016. PMCID: PMC5011063, PMID: 27569545.

[123] MR. Bekheirnia, N. Bekheirnia, MN. Bainbridge, S. Gu, ZH. Coban Akdemir, T. Gambin, NK. Janzen, SN. Jhangiani, DM. Muzny, M. Michael, ED. Brewer, E. Elenberg, AS. Kale, AA. Riley, SJ. Swartz, DA. Scott, Y. Yang, PR. Srivaths, SE. Wenderfer, J. Bodurtha, CD. Applegate, M. Velinov, A. Myers, L. Borovik, WJ. Craigen, NA. Hanchard, JA. Rosenfeld, RA. Lewis, ET. Gonzales, RA. Gibbs, JW. Belmont, DR. Roth, C. Eng, MC. Braun, JR. Lupski, and DJ. Lamb. Whole-exome sequencing in the molecular diagnosis of individuals with congenital anomalies of the kidney and urinary tract and identification of a new causative gene. Genet Med, 19 (4):412–420, 2017. PMCID: PMC5362362, PMID: 27657687.

[124] CR. Marshall, A. Noor, JB. Vincent, AC. Lionel, L. Feuk, J. Skaug, M. Shago, R. Moessner, D. Pinto, Y. Ren, B. Thiruvahindrapduram, A. Fiebig, S. Schreiber, J. Friedman, CE. Ketelaars, YJ. Vos, C. Ficicioglu, S. Kirkpatrick, R. Nicolson, L. Sloman, A. Summers, CA. Gibbons, A. Teebi, D. Chitayat, R. Weksberg, A. Thompson, C. Vardy, V. Crosbie, S. Luscombe, R. Baatjes, L. Zwaigenbaum, W. Roberts, B. Fernandez, P. Szatmari, and SW. Scherer. Structural variation of chromosomes in autism spectrum disorder. Am J Hum Genet, 82(2):477–488, 2008. PMCID: PMC2426913, PMID: 18252227.

[125] A. Noor, A. Whibley, CR. Marshall, PJ. Gianakopoulos, A. Piton, AR. Carson, M. Orlic-Milacic, AC. Lionel, D. Sato, D. Pinto, I. Drmic, C. Noakes, L. Senman, X. Zhang, R. Mo, J. Gauthier, J. Crosbie, AT. Pagnamenta, J. Munson, AM. Estes, A. Fiebig, A. Franke, S. Schreiber, AF. Stewart, R. Roberts, R. McPherson, SJ. Guter, EH. Cook, G. Dawson, GD. Schellenberg, A. Battaglia, E. Maestrini, L. Jeng, T. Hutchison, E. Rajcan-Separovic, AE. Chudley, SM. Lewis, X. Liu, JJ. Holden, B. Fernandez, L. Zwaigenbaum, SE. Bryson, W. Roberts, P. Szatmari, L. Gallagher, MR. Stratton, J. Gecz, AF. Brady, CE. Schwartz, RJ. Schachar, AP. Monaco, GA. Rouleau, CC. Hui, F. Lucy Raymond, SW. Scherer, and JB. Vincent. Disruption at the ptchd1 locus on xp22.11 in autism spectrum disorder and intellectual disability. Sci Transl Med, 2(49):49ra68, 2010. PMCID: PMC2987731, PMID: 20844286.

[126] MT. Carter, SM. Nikkel, BA. Fernandez, CR. Marshall, A. Noor, AC. Lionel, A. Prasad, D. Pinto, AM. Joseph-George, C. Noakes, C. Fairbrother-Davies, W. Roberts, J. Vincent,

R. Weksberg, and SW. Scherer. Hemizygous deletions on chromosome 1p21.3 involving the dpyd gene in individuals with autism spectrum disorder. Clin Genet, 80(5):435–443, 2011. PMID: 21114665.

[127] AB. Van Kuilenburg, P. Vreken, NG. Abeling, HD. Bakker, R. Meinsma, H. Van Lenthe, RA. De Abreu, JA. Smeitink, H. Kayserili, MY. Apak, E. Christensen, I. Holopainen, K. Pulkki, D. Riva, G. Botteon, E. Holme, M. Tulinius, WJ. Kleijer, FA. Beemer, M. Duran, KE. Niezen-Koning, GP. Smit, C. Jakobs, LM. Smit, and AH. Van Gennip. Genotype and phenotype in patients with dihydropyrimidine dehydrogenase deficiency. Hum Genet, 104(1):1–9, 1999. PMID: 10071185.

[128] P. Jézéquel, MP. Joalland, G. Milano, D. Lanoë, G. Ricolleau, E. Marie-Christine, and R. Deporte-Fety. Common dpyd mutation associated with 5-fluorouracil toxicity detected by pcr-mediated site-directed mutagenesis. Clin Chem, 46(2):309–310, 2000. PMID: 10657402.

[129] ES. Collie-Duguid, MC. Etienne, G. Milano, and HL. McLeod. Known variant dpyd alleles do not explain dpd deficiency in cancer patients. Pharmacogenetics, 10(3):217–223, 2000. PMID: 10803677.

[130] AB. van Kuilenburg, J. Haasjes, DJ. Richel, L. Zoetekouw, H. Van Lenthe, RA. De Abreu, JG. Maring, P. Vreken, and AH. van Gennip. Clinical implications of dihydropyrimidine dehydrogenase (dpd) deficiency in patients with severe 5-fluorouracil-associated toxicity: identification of new mutations in the dpd gene. Clin Cancer Res, 6 (12):4705–4712, 2000. PMID: 11156223.

[131] AB. van Kuilenburg, EW. Muller, J. Haasjes, R. Meinsma, L. Zoetekouw, HR. Waterham, F. Baas, DJ. Richel, and AH. van Gennip. Lethal outcome of a patient with a complete dihydropyrimidine dehydrogenase (dpd) deficiency after administration of 5-fluorouracil: frequency of the common ivs14+1g>a mutation causing dpd deficiency. Clin Cancer Res, 7(5):1149–1153, 2001. PMID: 11350878.

[132] M. Raida, W. Schwabe, P. Häusler, AB. Van Kuilenburg, AH. Van Gennip, D. Behnke, and K. Höffken. Prevalence of a common point mutation in the dihydropyrimidine dehydrogenase (dpd) gene within the 5'-splice donor site of intron 14 in patients with severe 5-fluorouracil (5-fu)- related toxicity compared with controls. Clin Cancer Res, 7(9):2832–2839, 2001. PMID: 11555601.

[133] MR. Johnson, K. Wang, and RB. Diasio. Profound dihydropyrimidine dehydrogenase deficiency resulting from a novel compound heterozygote genotype. Clin Cancer Res, 8(3):768–774, 2002. PMID: 11895907.

[134] JG. Maring, AB. van Kuilenburg, J. Haasjes, H. Piersma, HJ. Groen, DR. Uges, AH. Van Gennip, and EG. De Vries. Reduced 5-fu clearance in a patient with low dpd activity due to heterozygosity for a mutant allele of the dpyd gene. Br J Cancer, 86(7):1028–1033, 2002. PMCID: PMC2364178, PMID: 11953843.

[135] AB. van Kuilenburg, D. Dobritzsch, R. Meinsma, J. Haasjes, HR. Waterham, MJ. Nowaczyk, GD. Maropoulos, G. Hein, H. Kalhoff, JM. Kirk, H. Baaske, A. Aukett, JA. Duley, KP. Ward, Y. Lindqvist, and AH. van Gennip. Novel disease-causing mutations in

the dihydropyrimidine dehydrogenase gene interpreted by analysis of the three-dimensional protein structure. Biochem J, 364(Pt 1):157–163, 2002. PMCID: PMC1222557, PMID: 11988088.

[136] AB. Van Kuilenburg, R. Meinsma, L. Zoetekouw, and AH. Van Gennip. Increased risk of grade iv neutropenia after administration of 5-fluorouracil due to a dihydropyrimidine dehydrogenase deficiency: high prevalence of the ivs14+1g>a mutation. Int J Cancer, 101(3):253–258, 2002. PMID: 12209976.

[137] AB. Van Kuilenburg, R. Meinsma, L. Zoetekouw, and AH. Van Gennip. High prevalence of the ivs14 + 1g>a mutation in the dihydropyrimidine dehydrogenase gene of patients with severe 5-fluorouracil-associated toxicity. Pharmacogenetics, 12(7):555–558, 2002. PMID: 12360106.

[138] H. Ezzeldin, MR. Johnson, Y. Okamoto, and R. Diasio. Denaturing high performance liquid chromatography analysis of the dpyd gene in patients with lethal 5-fluorouracil toxicity. Clin Cancer Res, 9(8):3021–3028, 2003. PMID: 12912951.

[139] N. Salgueiro, I. Veiga, M. Fragoso, O. Sousa, N. Costa, ML. Pellon, E. Sanches, JG. dos Santos, MR. Teixeira, and S. Castedo. Mutations in exon 14 of dihydropyrimidine dehydrogenase and 5-fluorouracil toxicity in portuguese colorectal cancer patients. Genet Med, 6(2):102–107, 2004. PMID: 15017333.

[140] M. Steiner, M. Seule, B. Steiner, I. Bauer, M. Freund, CH. Köhne, and P. Schuff-Werner. 5-fluorouracil/irinotecan induced lethal toxicity as a result of a combined pharmacogenetic syndrome: report of a case. J Clin Pathol, 58 (5):553–555, 2005. PMCID: PMC1770668, PMID: 15858133.

[141] NA. Al-Sanna'a, AB. Van Kuilenburg, TM. Atrak, MA. Abdul-Jabbar, and AH. Van Gennip. Dihydropyrimidine dehydrogenase deficiency presenting at birth. J Inherit Metab Dis, 28(5):793–796, 2005. PMID: 16151913.

[142] HH. Ezzeldin, AM. Lee, LK. Mattison, and RB. Diasio. Methylation of the dpyd promoter: an alternative mechanism for dihydropyrimidine dehydrogenase deficiency in cancer patients. Clin Cancer Res, 11(24 Pt 1): 8699–8705, 2005. PMID: 16361556.

[143] R. Largillier, MC. Etienne-Grimaldi, JL. Formento, J. Ciccolini, JF. Nebbia, A. Ginot, M. Francoual, N. Renée, JM. Ferrero, C. Foa, M. Namer, B. Lacarelle, and G. Milano. Pharmacogenetics of capecitabine in advanced breast cancer patients. Clin Cancer Res, 12(18):5496–5502, 2006. PMID: 17000685.

[144] M. Boisdron-Celle, G. Remaud, S. Traore, AL. Poirier, L. Gamelin, A. Morel, and E. Gamelin. 5-fluorouracil-related severe toxicity: a comparison of different methods for the pretherapeutic detection of dihydropyrimidine dehydrogenase deficiency. Cancer Lett, 249(2):271–282, 2007. PMID: 17064846.

[145] A. Morel, M. Boisdron-Celle, L. Fey, P. Soulie, MC. Craipeau, S. Traore, and E. Gamelin. Clinical relevance of different dihydropyrimidine dehydrogenase gene single nucleotide polymorphisms on 5-fluorouracil tolerance. Mol Cancer Ther, 5(11):2895–2904, 2006. PMID: 17121937.

[146] MW. Saif, H. Ezzeldin, K. Vance, S. Sellers, and RB. Diasio. Dpyd*2a mutation: the most common mutation associated with dpd deficiency. Cancer Chemother Pharmacol, 60(4):503–507, 2007. PMID: 17165084.

[147] J. Salgado, N. Zabalegui, C. Gil, I. Monreal, J. Rodríguez, and J. García-Foncillas. Polymorphisms in the thymidylate synthase and dihydropyrimidine dehydrogenase genes predict response and toxicity to capecitabine-raltitrexed in colorectal cancer. Oncol Rep, 17(2):325–328, 2007. PMID: 17203168.

[148] N. Magné, MC. Etienne-Grimaldi, L. Cals, N. Renée, JL. Formento, M. Francoual, and G. Milano. Dihydropyrimidine dehydrogenase activity and the ivs14+1g>a mutation in patients developing 5fu-related toxicity. Br J Clin Pharmacol, 64(2):237–240, 2007. PMCID: PMC2000632, PMID: 17335544.

[149] O. Capitain, M. Boisdron-Celle, AL. Poirier, S. Abadie-Lacourtoisie, A. Morel, and E. Gamelin. The influence of fluorouracil outcome parameters on tolerance and efficacy in patients with advanced colorectal cancer. Pharmacogenomics J, 8(4):256–267, 2008. PMID: 17700593.

[150] M. Schwab, UM. Zanger, C. Marx, E. Schaeffeler, K. Klein, J. Dippon, R. Kerb, J. Blievernicht, J. Fischer, U. Hofmann, C. Bokemeyer, and M. Eichelbaum. Role of genetic and nongenetic factors for fluorouracil treatment-related severe toxicity: a prospective clinical trial by the german 5-fu toxicity study group. J Clin Oncol, 26(13):2131–2138, 2008. PMID: 18299612.

[151] V. Sulzyc-Bielicka, A. Bińczak-Kuleta, W. Pioch, J. Kładny, K. Gziut, D. Bielicki, and A. Ciechanowicz. 5-fluorouracil toxicity-attributable ivs14 + 1g > a mutation of the dihydropyrimidine dehydrogenase gene in polish colorectal cancer patients. Pharmacol Rep, 60(2):238–242, 2008. PMID: 18443386.

[152] AB. van Kuilenburg, JG. Maring, A. Schalhorn, C. Terborg, H. Schmalenberg, D. Behnke, W. Schwabe, K. Jabschinsky, and P. Hausler. Pharmacokinetics of 5-fluorouracil in patients heterozygous for the ivs14+1g > a mutation in the dihydropyrimidine dehydrogenase gene. Nucleosides Nucleotides Nucleic Acids, 27(6):692–698, 2008. PMID: 18600527.

[153] E. Gross, B. Busse, M. Riemenschneider, S. Neubauer, K. Seck, HG. Klein, M. Kiechle, F. Lordick, and A. Meindl. Strong association of a common dihydropyrimidine dehydrogenase gene polymorphism with fluoropyrimidine-related toxicity in cancer patients. PLoS One, 3(12):e4003, 2008. PMCID: PMC2602733, PMID: 19104657.

[154] Z. Kleibl, J. Fidlerova, P. Kleiblova, S. Kormunda, M. Bilek, K. Bouskova, J. Sevcik, and J. Novotny. Influence of dihydropyrimidine dehydrogenase gene (dpyd) coding sequence variants on the development of fluoropyrimidine-related toxicity in patients with high-grade toxicity and patients with excellent tolerance of fluoropyrimidine-based chemotherapy. Neoplasma, 56(4):303–316, 2009. PMID: 19473056.

[155] A. Loganayagam, M. Arenas-Hernandez, L. Fairbanks, P. Ross, JD. Sanderson, and AM. Marinaki. The contribution of deleterious dpyd gene sequence variants to fluoropyrimidine toxicity in british cancer patients. Cancer Chemother Pharmacol,

65(2):403–406, 2010. PMID: 19795123.

[156] MS. Braun, SD. Richman, L. Thompson, CL. Daly, AM. Meade, JW. Adlard, JM. Allan, MK. Parmar, P. Quirke, and MT. Seymour. Association of molecular markers with toxicity outcomes in a randomized trial of chemotherapy for advanced colorectal cancer: the focus trial. J Clin Oncol, 27(33):5519–5528, 2009. PMID: 19858398.

[157] V. Boige, J. Mendiboure, JP. Pignon, MA. Loriot, M. Castaing, M. Barrois, D. Malka, DA. Trégouët, O. Bouché, D. Le Corre, I. Miran, C. Mulot, M. Ducreux, P. Beaune, and P. Laurent-Puig. Pharmacogenetic assessment of toxicity and outcome in patients with metastatic colorectal cancer treated with lv5fu2, folfox, and folfiri: Ffcd 2000-05. J Clin Oncol, 28(15):2556–2564, 2010. PMID: 20385995.

[158] T. Cerić, N. Obralić, L. Kapur-Pojskić, D. Macić, S. Beslija, A. Pasić, and S. Cerić. Investigation of ivs14 + 1g > a polymorphism of dpyd gene in a group of bosnian patients treated with 5-fluorouracil and capecitabine. Bosn J Basic Med Sci, 10(2):133–139, 2010. PMCID: PMC5509399, PMID: 20507294.

[159] HL. McLeod, DJ. Sargent, S. Marsh, EM. Green, CR. King, CS. Fuchs, RK. Ramanathan, SK. Williamson, BP. Findlay, SN. Thibodeau, A. Grothey, RF. Morton, and RM. Goldberg. Pharmacogenetic predictors of adverse events and response to chemotherapy in metastatic colorectal cancer: results from north american gastrointestinal intergroup trial n9741. J Clin Oncol, 28(20):3227–3233, 2010. PMCID: PMC2903324, PMID: 20530282.

[160] AB. van Kuilenburg, J. Meijer, AN. Mul, R. Meinsma, V. Schmid, D. Dobritzsch, RC. Hennekam, MM. Mannens, M. Kiechle, MC. Etienne-Grimaldi, HJ. Klümpen, JG. Maring, VA. Derleyn, E. Maartense, G. Milano, R. Vijzelaar, and E. Gross. Intragenic deletions and a deep intronic mutation affecting pre-mrna splicing in the dihydropyrimidine dehydrogenase gene as novel mechanisms causing 5-fluorouracil toxicity. Hum Genet, 128 (5):529–538, 2010. PMCID: PMC2955237, PMID: 20803296.

[161] J. Savva-Bordalo, J. Ramalho-Carvalho, M. Pinheiro, VL. Costa, A. Rodrigues, PC. Dias, I. Veiga, M. Machado, MR. Teixeira, R. Henrique, and C. Jerónimo. Promoter methylation and large intragenic rearrangements of dpyd are not implicated in severe toxicity to 5-fluorouracil-based chemotherapy in gastrointestinal cancer patients. BMC Cancer, 10:470, 2010. PMCID: PMC2940808, PMID: 20809970.

[162] MH. Kristensen, PL. Pedersen, GV. Melsen, J. Ellehauge, and J. Mejer. Variants in the dihydropyrimidine dehydrogenase, methylenetetrahydrofolate reductase and thymidylate synthase genes predict early toxicity of 5-fluorouracil in colorectal cancer patients. J Int Med Res, 38(3):870–883, 2010. PMID: 20819423.

[163] P. Cellier, B. Leduc, L. Martin, B. Vié, C. Chevelle, V. Vendrely, A. Salemkour, C. Carrie, G. Calais, P. Burtin, L. Campion, M. Boisdron-Celle, A. Morel, V. Berger, and E. Gamelin. Phase ii study of preoperative radiation plus concurrent daily tegafur-uracil (uft) with leucovorin for locally advanced rectal cancer. BMC Cancer, 11:98, 2011. PMCID: PMC3070684, PMID: 21410976.

[164] MJ. Deenen, J. Tol, AM. Burylo, VD. Doodeman, A. de Boer, A. Vincent, HJ. Guchelaar,

PH. Smits, JH. Beijnen, CJ. Punt, JH. Schellens, and A. Cats. Relationship between single nucleotide polymorphisms and haplotypes in dpyd and toxicity and efficacy of capecitabine in advanced colorectal cancer. Clin Cancer Res, 17(10): 3455–3468, 2011. PMID: 21498394.

[165] CA. Galván, OC. Elbarcha, EJ. Fernández, DM. Beltramo, and NW. Soria. Genetic profiling of gstp1, dpyd, fcgr2a, fcgr3a and ccnd1 genes in an argentinian population. Clin Biochem, 44(13):1058–1061, 2011. PMID: 21723269.

[166] U. Amstutz, TK. Froehlich, and CR. Largiadèr. Dihydropyrimidine dehydrogenase gene as a major predictor of severe 5-fluorouracil toxicity. Pharmacogenomics, 12(9):1321–1336, 2011. PMID: 21919607.

[167] AB. van Kuilenburg, P. Häusler, A. Schalhorn, MW. Tanck, JH. Proost, C. Terborg, D. Behnke, W. Schwabe, K. Jabschinsky, and JG. Maring. Evaluation of 5-fluorouracil pharmacokinetics in cancer patients with a c.1905+1g>a mutation in dpyd by means of a bayesian limited sampling strategy. Clin Pharmacokinet, 51(3): 163–174, 2012. PMID: 22339448.

[168] M. Whirl-Carrillo, EM. McDonagh, JM. Hebert, L. Gong, K. Sangkuhl, CF. Thorn, RB. Altman, and TE. Klein. Pharmacogenomics knowledge for personalized medicine. Clin Pharmacol Ther, 92(4):414–417, 2012. PMCID: PMC3660037, PMID: 22992668.

[169] SM. Offer, NJ. Wegner, C. Fossum, K. Wang, and RB. Diasio. Phenotypic profiling of dpyd variations relevant to 5-fluorouracil sensitivity using real-time cellular analysis and in vitro measurement of enzyme activity. Cancer Res, 73(6):1958–1968, 2013. PMCID: PMC3602211, PMID: 23328581.

[170] E. Borràs, E. Dotor, A. Arcusa, MJ. Gamundi, I. Hernan, M. de Sousa Dias, B. Mañé, JA. Agúndez, M. Blanca, and M. Carballo. High-resolution melting analysis of the common c.1905+1g>a mutation causing dihydropyrimidine dehydrogenase deficiency and lethal 5-fluorouracil toxicity. Front Genet, 3:312, 2012. PMCID: PMC3547229, PMID: 23335937.

[171] D. Dhawan, H. Panchal, S. Shukla, and H. Padh. Genetic variability & chemotoxicity of 5-fluorouracil & cisplatin in head & neck cancer patients: a preliminary study. Indian J Med Res, 137(1):125–129, 2013. PMCID: PMC3657875, PMID: 23481061.

[172] E. Magnani, E. Farnetti, D. Nicoli, B. Casali, L. Savoldi, C. Focaccetti, C. Boni, A. Albini, and M. Banzi. Fluoropyrimidine toxicity in patients with dihydropyrimidine dehydrogenase splice site variant: the need for further revision of dose and schedule. Intern Emerg Med, 8(5):417–423, 2013. PMID: 23585145.

[173] MW. Saif. Dihydropyrimidine dehydrogenase gene (dpyd) polymorphism among caucasian and non-caucasian patients with 5-fu- and capecitabine-related toxicity using full sequencing of dpyd. Cancer Genomics Proteomics, 10(2):89–92, 2013. PMID: 23603345.

[174] A. Loganayagam, M. Arenas Hernandez, A. Corrigan, L. Fairbanks, CM. Lewis, P. Harper, N. Maisey, P. Ross, JD. Sanderson, and AM. Marinaki. Pharmacogenetic variants in the dpyd, tyms, cda and mthfr genes are clinically significant predictors of fluoropyrimidine

toxicity. Br J Cancer, 108(12):2505–2515, 2013. PMCID: PMC3694243, PMID: 23736036.

[175] S. Terrazzino, S. Cargnin, M. Del Re, R. Danesi, PL. Canonico, and AA. Genazzani. Dpyd ivs14+1g>a and 2846a>t genotyping for the prediction of severe fluoropyrimidine-related toxicity: a meta-analysis. Pharmacogenomics, 14(11):1255–1272, 2013. PMID: 23930673.

[176] BA. Jennings, YK. Loke, J. Skinner, M. Keane, GS. Chu, R. Turner, D. Epurescu, A. Barrett, and G. Willis. Evaluating predictive pharmacogenetic signatures of adverse events in colorectal cancer patients treated with fluoropyrimidines. PLoS One, 8(10):e78053, 2013. PMCID: PMC3805522, PMID: 24167597.

[177] D. Rosmarin, C. Palles, D. Church, E. Domingo, A. Jones, E. Johnstone, H. Wang, S. Love, P. Julier, C. Scudder, G. Nicholson, A. Gonzalez-Neira, M. Martin, D. Sargent, E. Green, H. McLeod, UM. Zanger, M. Schwab, M. Braun, M. Seymour, L. Thompson, B. Lacas, V. Boige, N. Ribelles, S. Afzal, H. Enghusen, SA. Jensen, MC. Etienne-Grimaldi, G. Milano, M. Wadelius, B. Glimelius, H. Garmo, M. Gusella, T. Lecomte, P. Laurent-Puig, E. Martinez-Balibrea, R. Sharma, J. Garcia-Foncillas, Z. Kleibl, A. Morel, JP. Pignon, R. Midgley, D. Kerr, and I. Tomlinson. Genetic markers of toxicity from capecitabine and other fluorouracil-based regimens: investigation in the quasar2 study, systematic review, and meta-analysis. J Clin Oncol, 32(10):1031–1039, 2014. PMCID: PMC4879695, PMID: 24590654.

[178] D. Rosmarin, C. Palles, A. Pagnamenta, K. Kaur, G. Pita, M. Martin, E. Domingo, A. Jones, K. Howarth, L. Freeman-Mills, E. Johnstone, H. Wang, S. Love, C. Scudder, P. Julier, C. Fernández-Rozadilla, C. Ruiz-Ponte, A. Carracedo, S. Castellvi-Bel, A. Castells, A. Gonzalez-Neira, J. Taylor, R. Kerr, D. Kerr, and I. Tomlinson. A candidate gene study of capecitabine-related toxicity in colorectal cancer identifies new toxicity variants at dpyd and a putative role for enosf1 rather than tyms. Gut, 64(1):111–120, 2015. PMCID: PMC4283622, PMID: 24647007.

[179] SM. Offer, CC. Fossum, NJ. Wegner, AJ. Stuflesser, GL. Butterfield, and RB. Diasio. Comparative functional analysis of dpyd variants of potential clinical relevance to dihydropyrimidine dehydrogenase activity. Cancer Res, 74(9):2545–2554, 2014. PMCID: PMC4012613, PMID: 24648345.

[180] B. Suarez Martinez-Falero and R. Gillmore. A rare cause of susceptibility to neutropenic sepsis in a patient with metastatic pancreas cancer. BMJ Case Rep, 2014, 2014. PMCID: PMC3987224, PMID: 24700034.

[181] X. Cai, JM. Fang, P. Xue, WF. Song, J. Hu, HL. Gu, HY. Yang, and LW. Wang. The role of ivs14+1 g > a genotype detection in the dihydropyrimidine dehydrogenase gene and pharmacokinetic monitoring of 5-fluorouracil in the individualized adjustment of 5-fluorouracil for patients with local advanced and metastatic colorectal cancer: a preliminary report. Eur Rev Med Pharmacol Sci, 18(8):1247–1258, 2014. PMID: 24817302.

[182] TK. Froehlich, U. Amstutz, S. Aebi, M. Joerger, and CR. Largiadèr. Clinical importance of risk variants in the dihydropyrimidine dehydrogenase gene for the prediction of early-

onset fluoropyrimidine toxicity. Int J Cancer, 136(3):730–739, 2015. PMID: 24923815.

[183] AM. Lee, Q. Shi, E. Pavey, SR. Alberts, DJ. Sargent, FA. Sinicrope, JL. Berenberg, RM. Goldberg, and RB. Diasio. Dpyd variants as predictors of 5-fluorouracil toxicity in adjuvant colon cancer treatment (ncctg n0147). J Natl Cancer Inst, 106(12), 2014. PMCID: PMC4271081, PMID: 25381393.

[184] J. Sistonen, B. Büchel, TK. Froehlich, D. Kummer, S. Fontana, M. Joerger, AB. van Kuilenburg, and CR. Largiadèr. Predicting 5-fluorouracil toxicity: Dpd genotype and 5,6-dihydrouracil:uracil ratio. Pharmacogenomics, 15(13):1653–1666, 2014. PMID: 25410891.

[185] X. Zhu, S. Petrovski, P. Xie, EK. Ruzzo, YF. Lu, KM. McSweeney, B. Ben-Zeev, A. Nissenkorn, Y. Anikster, D. Oz-Levi, RS. Dhindsa, Y. Hitomi, K. Schoch, RC. Spillmann, G. Heimer, D. Marek-Yagel, M. Tzadok, Y. Han, G. Worley, J. Goldstein, YH. Jiang, D. Lancet, E. Pras, V. Shashi, D. McHale, AC. Need, and DB. Goldstein. Whole-exome sequencing in undiagnosed genetic diseases: interpreting 119 trios. Genet Med, 17(10): 774–781, 2015. PMCID: PMC4791490, PMID: 25590979.

[186] M. Joerger, AD. Huitema, H. Boot, A. Cats, VD. Doodeman, PH. Smits, L. Vainchtein, H. Rosing, I. Meijerman, M. Zueger, D. Meulendijks, TD. Cerny, JH. Beijnen, and JH. Schellens. Germline tyms genotype is highly predictive in patients with metastatic gastrointestinal malignancies receiving capecitabine-based chemotherapy. Cancer Chemother Pharmacol, 75(4):763–772, 2015. PMID: 25677447.

[187] G. Toffoli, L. Giodini, A. Buonadonna, M. Berretta, A. De Paoli, S. Scalone, G. Miolo, E. Mini, S. Nobili, S. Lonardi, N. Pella, G. Lo Re, M. Montico, R. Roncato, E. Dreussi, S. Gagno, and E. Cecchin. Clinical validity of a dpyd-based pharmacogenetic test to predict severe toxicity to fluoropyrimidines. Int J Cancer, 137(12): 2971–2980, 2015. PMID: 26099996.

[188] G. Gentile, A. Botticelli, L. Lionetto, F. Mazzuca, M. Simmaco, P. Marchetti, and M. Borro. Genotype-phenotype correlations in 5-fluorouracil metabolism: a candidate dpyd haplotype to improve toxicity prediction. Pharmacogenomics J, 16(4):320–325, 2016. PMID: 26216193.

[189] F. Thomas, I. Hennebelle, C. Delmas, I. Lochon, C. Dhelens, C. Garnier Tixidre, A. Bonadona, N. Penel, A. Goncalves, JP. Delord, C. Toulas, and E. Chatelut. Genotyping of a family with a novel deleterious dpyd mutation supports the pretherapeutic screening of dpd deficiency with dihydrouracil/uracil ratio. Clin Pharmacol Ther, 99(2):235–242, 2016. PMID: 26265035.

[190] I. Karbassi, GA. Maston, A. Love, C. DiVincenzo, CD. Braastad, CD. Elzinga, AR. Bright, D. Previte, K. Zhang, CM. Rowland, M. McCarthy, JL. Lapierre, F. Dubois, KA. Medeiros, SD. Batish, J. Jones, K. Liaquat, CA. Hoffman, M. Jaremko, Z. Wang, W. Sun, A. Buller-Burckle, CM. Strom, SB. Keiles, and JJ. Higgins. A standardized dna variant scoring system for pathogenicity assessments in mendelian disorders. Hum Mutat, 37 (1):127–134, 2016. PMCID: PMC4737317, PMID: 26467025.

[191] D. Meulendijks, LM. Henricks, GS. Sonke, MJ. Deenen, TK. Froehlich, U. Amstutz, CR.

Largiadèr, BA. Jennings, AM. Marinaki, JD. Sanderson, Z. Kleibl, P. Kleiblova, M. Schwab, UM. Zanger, C. Palles, I. Tomlinson, E. Gross, AB. van Kuilenburg, CJ. Punt, M. Koopman, JH. Beijnen, A. Cats, and JH. Schellens. Clinical relevance of dpyd variants c.1679t>g, c.1236g>a/hapb3, and c.1601g>a as predictors of severe fluoropyrimidine-associated toxicity: a systematic review and meta-analysis of individual patient data. Lancet Oncol, 16(16):1639–1650, 2015. PMID: 26603945.

[192] V. Boige, M. Vincent, P. Alexandre, S. Tejpar, S. Landolfi, K. Le Malicot, R. Greil, PJ. Cuyle, M. Yilmaz, R. Faroux, A. Matzdorff, R. Salazar, C. Lepage, J. Taieb, and P. Laurent-Puig. Dpyd genotyping to predict adverse events following treatment with fluorouracil-based adjuvant chemotherapy in patients with stage iii colon cancer: A secondary analysis of the petacc-8 randomized clinical trial. JAMA Oncol, 2(5):655–662, 2016. PMID: 26794347.

[193] ABPV. Kuilenburg, J. Meijer, MWT. Tanck, D. Dobritzsch, L. Zoetekouw, LL. Dekkers, J. Roelofsen, R. Meinsma, M. Wymenga, W. Kulik, B. Büchel, RCM. Hennekam, and CR. Largiadèr. Phenotypic and clinical implications of variants in the dihydropyrimidine dehydrogenase gene. Biochim Biophys Acta, 1862(4):754–762, 2016. PMID: 26804652.

[194] XQ. Zhao, WJ. Cao, HP. Yang, XW. Yang, P. Tang, L. Sun, and X. Gao. Dpyd gene polymorphisms are associated with risk and chemotherapy prognosis in pediatric patients with acute lymphoblastic leukemia. Tumour Biol, 37(8):10393–10402, 2016. PMID: 26846104.

[195] F. Mazzuca, M. Borro, A. Botticelli, E. Mazzotti, L. Marchetti, G. Gentile, M. La Torre, L. Lionetto, M. Simmaco, and P. Marchetti. Pre-treatment evaluation of 5-fluorouracil degradation rate: association of poor and ultra-rapid metabolism with severe toxicity in a colorectal cancer patients cohort. Oncotarget, 7(15):20612–20620, 2016. PMCID: PMC4991479, PMID: 26967565.

[196] G. Milano. Highlight on dpyd gene polymorphisms and treatment by capecitabine (.). Scand J Clin Lab Invest Suppl, 245:30–33, 2016. PMID: 27454530.

[197] M. Borro, A. Botticelli, F. Mazzuca, EC. Onesti, G. Gentile, A. Romiti, B. Cerbelli, E. Mazzotti, L. Marchetti, L. Lionetto, M. Simmaco, and P. Marchetti. Pre-treatment assay of 5-fluorouracil degradation rate (5-fudr) to improve prediction of 5-fluorouracil toxicity in gastro-esophageal cancer. Oncotarget, 8(8):14050–14057, 2017. PMCID: PMC5355161, PMID: 27738344.

[198] M. Roberto, A. Romiti, A. Botticelli, F. Mazzuca, L. Lionetto, G. Gentile, I. Paris, R. Falcone, M. Bassanelli, FR. Di Pietro, CE. Onesti, E. Anselmi, S. Macrini, M. Simmaco, and P. Marchetti. Evaluation of 5-fluorouracil degradation rate and pharmacogenetic profiling to predict toxicity following adjuvant capecitabine. Eur J Clin Pharmacol, 73(2):157–164, 2017. PMID: 27864592.

[199] Q. Nie, S. Shrestha, EE. Tapper, CS. Trogstad-Isaacson, KJ. Bouchonville, AM. Lee, R. Wu, CR. Jerde, Z. Wang, PA. Kubica, SM. Offer, and RB. Diasio. Quantitative contribution of rs75017182 to dihydropyrimidine dehydrogenase mrna splicing and enzyme activity. Clin Pharmacol Ther, 102(4):662–670, 2017. PMCID: PMC6138243, PMID: 28295243.

[200] MC. Etienne-Grimaldi, JC. Boyer, C. Beroud, L. Mbatchi, A. van Kuilenburg, C. Bobin-Dubigeon, F. Thomas, E. Chatelut, JL. Merlin, F. Pinguet, C. Ferrand, J. Meijer, A. Evrard, L. Llorca, G. Romieu, P. Follana, T. Bachelot, L. Chaigneau, X. Pivot, V. Dieras, R. Largillier, M. Mousseau, A. Goncalves, H. Roché, J. Bonneterre, V. Servent, N. Dohollou, Y. Château, E. Chamorey, JP. Desvignes, D. Salgado, JM. Ferrero, and G. Milano. New advances in dpyd genotype and risk of severe toxicity under capecitabine. PLoS One, 12(5):e0175998, 2017. PMCID: PMC5421769, PMID: 28481884.

[201] LM. Henricks, EJM. Siemerink, H. Rosing, J. Meijer, SMI. Goorden, AM. Polstra, L. Zoetekouw, A. Cats, JHM. Schellens, and ABP. van Kuilenburg. Capecitabine-based treatment of a patient with a novel dpyd genotype and complete dihydropyrimidine dehydrogenase deficiency. Int J Cancer, 142(2):424–430, 2018. PMID: 28929491.

[202] A. Ruzzo, F. Graziano, F. Galli, F. Galli, E. Rulli, S. Lonardi, M. Ronzoni, B. Massidda, V. Zagonel, N. Pella, C. Mucciarini, R. Labianca, MT. Ionta, I. Bagaloni, E. Veltri, P. Sozzi, S. Barni, V. Ricci, L. Foltran, M. Nicolini, E. Biondi, A. Bramati, D. Turci, S. Lazzarelli, C. Verusio, F. Bergamo, A. Sobrero, L. Frontini, M. Menghi, and M. Magnani. Dihydropyrimidine dehydrogenase pharmacogenetics for predicting fluoropyrimidine-related toxicity in the randomised, phase iii adjuvant tosca trial in high-risk colon cancer patients. Br J Cancer, 117(9): 1269–1277, 2017. PMCID: PMC5709672, PMID: 29065426.

[203] NA. Nahid, MNH. Apu, MR. Islam, S. Shabnaz, SM. Chowdhury, MU. Ahmed, Z. Nahar, MS. Islam, MS. Islam, and A. Hasnat. Dpyd*2a and mthfr c677t predict toxicity and efficacy, respectively, in patients on chemotherapy with 5-fluorouracil for colorectal cancer. Cancer Chemother Pharmacol, 81(1):119–129, 2018. PMID: 29134491.

[204] U. Amstutz, LM. Henricks, SM. Offer, J. Barbarino, JHM. Schellens, JJ. Swen, TE. Klein, HL. McLeod, KE. Caudle, RB. Diasio, and M. Schwab. Clinical pharmacogenetics implementation consortium (cpic) guideline for dihydropyrimidine dehydrogenase genotype and fluoropyrimidine dosing: 2017 update. Clin Pharmacol Ther, 103(2):210–216, 2018. PMCID: PMC5760397, PMID: 29152729.

[205] LM. Henricks, CATC. Lunenburg, FM. de Man, D. Meulendijks, GWJ. Frederix, E. Kienhuis, GJ. Creemers, A. Baars, VO. Dezentjé, ALT. Imholz, FJF. Jeurissen, JEA. Portielje, RLH. Jansen, P. Hamberg, AJ. Ten Tije, HJ. Droogendijk, M. Koopman, P. Nieboer, MHW. van de Poel, CMPW. Mandigers, H. Rosing, JH. Beijnen, EV. Werkhoven, ABP. van Kuilenburg, RHN. van Schaik, RHJ. Mathijssen, JJ. Swen, H. Gelderblom, A. Cats, HJ. Guchelaar, and JHM. Schellens. Dpyd genotype-guided dose individualisation of fluoropyrimidine therapy in patients with cancer: a prospective safety analysis. Lancet Oncol, 19(11):1459–1467, 2018. PMID: 30348537.

[206] R. Meinsma, P. Fernandez-Salguero, AB. Van Kuilenburg, AH. Van Gennip, and FJ. Gonzalez. Human polymorphism in drug metabolism: mutation in the dihydropyrimidine dehydrogenase gene results in exon skipping and thymine uracilurea. DNA Cell Biol, 14(1):1–6, 1995. PMID: 7832988.

[207] AH. van Gennip, NG. Abeling, AE. Stroomer, H. van Lenthe, and HD. Bakker. Clinical and biochemical findings in six patients with pyrimidine degradation defects. J Inherit Metab Dis, 17(1):130–132, 1994. PMID: 8051923.

[208] X. Wei, HL. McLeod, J. McMurrough, FJ. Gonzalez, and P. Fernandez-Salguero. Molecular basis of the human dihydropyrimidine dehydrogenase deficiency and 5-fluorouracil toxicity. J Clin Invest, 98(3):610–615, 1996. PMCID: PMC507468, PMID: 8698850.

[209] P. Vreken, AB. Van Kuilenburg, R. Meinsma, GP. Smit, HD. Bakker, RA. De Abreu, and AH. van Gennip. A point mutation in an invariant splice donor site leads to exon skipping in two unrelated dutch patients with dihydropyrimidine dehydrogenase deficiency. J Inherit Metab Dis, 19(5):645–654, 1996. PMID: 8892022.

[210] I. Holopainen, K. Pulkki, OJ. Heinonen, K. Näntö-Salonen, L. Haataja, J. Greter, E. Holme, AB. van Kuilenburg, P. Vreken, and AH. van Gennip. Partial epilepsy in a girl with a symptom-free sister: first two finnish patients with dihydropyrimidine dehydrogenase deficiency. J Inherit Metab Dis, 20(5):719–720, 1997. PMID: 9323575.

[211] P. Vreken, AB. Van Kuilenburg, R. Meinsma, and AH. van Gennip. Dihydropyrimidine dehydrogenase (dpd) deficiency: identification and expression of missense mutations c29r, r886h and r235w. Hum Genet, 101(3): 333–338, 1997. PMID: 9439663.

[212] AB. Van Kuilenburg, P. Vreken, LV. Beex, R. Meinsma, H. Van Lenthe, RA. De Abreu, and AH. van Gennip. Heterozygosity for a point mutation in an invariant splice donor site of dihydropyrimidine dehydrogenase and severe 5-fluorouracil related toxicity. Eur J Cancer, 33(13):2258–2264, 1997. PMID: 9470816.

[213] P. Vreken, AB. van Kuilenburg, R. Meinsma, FA. Beemer, M. Duran, and AH. van Gennip. Dihydropyrimidine dehydrogenase deficiency: a novel mutation and expression of missense mutations in e. coli. J Inherit Metab Dis, 21(3):276–279, 1998. PMID: 9686374.

[214] J. Lindberg, IG. Mills, D. Klevebring, W. Liu, M. Neiman, J. Xu, P. Wikström, P. Wiklund, F. Wiklund, L. Egevad, and H. Grönberg. The mitochondrial and autosomal mutation landscapes of prostate cancer. Eur Urol, 63(4): 702–708, 2013. PMID: 23265383.

[215] K. Miyake, S. Imura, T. Yoshizumi, T. Ikemoto, Y. Morine, and M. Shimada. Role of thymidine phosphorylase and orotate phosphoribosyltransferase mrna expression and its ratio to dihydropyrimidine dehydrogenase in the prognosis and clinicopathological features of patients with pancreatic cancer. Int J Clin Oncol, 12(2):111–119, 2007. PMID: 17443278.

[216] Y. Fukui, T. Oka, S. Nagayama, PV. Danenberg, KD. Danenberg, and M. Fukushima. Thymidylate synthase, dihydropyrimidine dehydrogenase, orotate phosphoribosyltransferase mrna and protein expression levels in solid tumors in large scale population analysis. Int J Mol Med, 22(6):709–716, 2008. PMID: 19020767.

[217] T. Takahashi, H. Yoshida, Y. Mamada, N. Taniai, Y. Mizuguchi, T. Shimizu, D. Kakinuma, Y. Ishikawa, K. Akimaru, Y. Sugisaki, and T. Tajiri. Profiling of fluorouracil-related genes by microdissection technique in hepatocellular carcinoma. Hepatogastroenterology, 54(78):1612–1616, 2007. PMID: 18019677.

[218] R. Napieralski, K. Ott, M. Kremer, K. Specht, H. Vogelsang, K. Becker, M. Müller, F. Lordick, U. Fink, J. Rüdiger Siewert, H. Höfler, and G. Keller. Combined gadd45a and

thymidine phosphorylase expression levels predict response and survival of neoadjuvant-treated gastric cancer patients. Clin Cancer Res, 11(8): 3025–3031, 2005. PMID: 15837757.

[219] M. Oeda, K. Yoshida, Y. Sanada, Y. Wada, T. Suzuki, H. Mizuiri, K. Konishi, H. Shigematsu, K. Tanabe, and M. Fukushima. The expression profiles of orotate phosphoribosyltransferase and dihydropyrimidine dehydrogenase in gastric cancer and their clinical significance. Oncol Rep, 16(6):1165–1172, 2006. PMID: 17089033.

[220] H. Makino, H. Uetake, K. Danenberg, PV. Danenberg, and K. Sugihara. Efficacy of laser capture microdissection plus rt-pcr technique in analyzing gene expression levels in human gastric cancer and colon cancer. BMC Cancer, 8:210, 2008. PMCID: PMC2533342, PMID: 18652704.

[221] D. Vallböhmer, DY. Yang, H. Kuramochi, D. Shimizu, KD. Danenberg, J. Lindebjerg, JN. Nielsen, A. Jakobsen, and PV. Danenberg. Dpd is a molecular determinant of capecitabine efficacy in colorectal cancer. Int J Oncol, 31(2):413–418, 2007. PMID: 17611699.

[222] H. Nagano, W. Ichikawa, M. Simizu, Y. Shirota, and Z. Nihei. [thymidylate synthase and dihydropyrimidine dehydrogenase gene expressions in colorectal cancer using the danenberg tumor profile method]. Gan To Kagaku Ryoho, 31(6):889–892, 2004. PMID: 15222106.

[223] EA. Kidd, J. Yu, X. Li, WD. Shannon, MA. Watson, and HL. McLeod. Variance in the expression of 5-fluorouracil pathway genes in colorectal cancer. Clin Cancer Res, 11(7):2612–2619, 2005. PMID: 15814641.

[224] F. Amatori, A. Di Paolo, M. Del Tacca, G. Fontanini, F. Vannozzi, L. Boldrini, G. Bocci, M. Lastella, and R. Danesi. Thymidylate synthase, dihydropyrimidine dehydrogenase and thymidine phosphorylase expression in colorectal cancer and normal mucosa in patients. Pharmacogenet Genomics, 16(11):809–816, 2006. PMID: 17047489.

[225] DA. Franco and HS. Greenberg. 5-fu multifocal inflammatory leukoencephalopathy and dihydropyrimidine dehydrogenase deficiency. Neurology, 56(1):110–112, 2001. PMID: 11148247.

[226] C. Schmidt, U. Hofmann, D. Kohlmüller, T. Mürdter, UM. Zanger, M. Schwab, and GF. Hoffmann. Comprehensive analysis of pyrimidine metabolism in 450 children with unspecific neurological symptoms using high-pressure liquid chromatography-electrospray ionization tandem mass spectrometry. J Inherit Metab Dis, 28 (6):1109–1122, 2005. PMID: 16435204.

[227] AB. van Kuilenburg, JW. Baars, R. Meinsma, and AH. van Gennip. Lethal 5-fluorouracil toxicity associated with a novel mutation in the dihydropyrimidine dehydrogenase gene. Ann Oncol, 14(2):341–342, 2003. PMID: 12562666.

[228] A. Broyl, SL. Corthals, JL. Jongen, B. van der Holt, R. Kuiper, Y. de Knegt, M. van Duin, L. el Jarari, U. Bertsch, HM. Lokhorst, BG. Durie, H. Goldschmidt, and P. Sonneveld. Mechanisms of peripheral neuropathy associated with bortezomib and vincristine in patients with newly diagnosed multiple myeloma: a prospective analysis of data from the

hovon-65/gmmg-hd4 trial. Lancet Oncol, 11(11):1057–1065, 2010. PMID: 20864405.

[229] B. Xu, JL. Roos, P. Dexheimer, B. Boone, B. Plummer, S. Levy, JA. Gogos, and M. Karayiorgou. Exome sequencing supports a de novo mutational paradigm for schizophrenia. Nat Genet, 43(9):864–868, 2011. PMCID: PMC3196550, PMID: 21822266.

[230] B. Xu, I. Ionita-Laza, JL. Roos, B. Boone, S. Woodrick, Y. Sun, S. Levy, JA. Gogos, and M. Karayiorgou. De novo gene mutations highlight patterns of genetic and neural complexity in schizophrenia. Nat Genet, 44(12): 1365–1369, 2012. PMCID: PMC3556813, PMID: 23042115.

[231] RB. Diasio, TL. Beavers, and JT. Carpenter. Familial deficiency of dihydropyrimidine dehydrogenase. biochemical basis for familial pyrimidinemia and severe 5-fluorouracil-induced toxicity. J Clin Invest, 81(1): 47–51, 1988. PMCID: PMC442471, PMID: 3335642.

[232] AB. van Kuilenburg, R. Meinsma, and AH. van Gennip. Pyrimidine degradation defects and severe 5-fluorouracil toxicity. Nucleosides Nucleotides Nucleic Acids, 23(8-9):1371–1375, 2004. PMID: 15571261.

[233] S. Podliesna, J. Delanne, L. Miller, DJ. Tester, M. Uzunyan, S. Yano, M. Klerk, BC. Cannon, A. Khongphatthanayothin, G. Laurent, G. Bertaux, S. Falcon-Eicher, S. Wu, HY. Yen, H. Gao, AAM. Wilde, L. Faivre, MJ. Ackerman, EM. Lodder, and CR. Bezzina. Supraventricular tachycardias, conduction disease, and cardiomyopathy in 3 families with the same rare variant in tnni3k (p.glu768lys). Heart Rhythm, 16(1): 98–9105, 2019. PMID: 30010057.

[234] S. Hoshimoto, CT. Kuo, KK. Chong, TL. Takeshima, Y. Takei, MW. Li, SK. Huang, MS. Sim, DL. Morton, and DS. Hoon. Aim1 and line-1 epigenetic aberrations in tumor and serum relate to melanoma progression and disease outcome. J Invest Dermatol, 132(6):1689–1697, 2012. PMCID: PMC3352986, PMID: 22402438.

[235] ME. Talkowski, SV. Mullegama, JA. Rosenfeld, BW. van Bon, Y. Shen, EA. Repnikova, J. Gastier-Foster, DL. Thrush, S. Kathiresan, DM. Ruderfer, C. Chiang, C. Hanscom, C. Ernst, AM. Lindgren, CC. Morton, Y. An, C. Astbury, LA. Brueton, KD. Lichtenbelt, LC. Ades, M. Fichera, C. Romano, JW. Innis, CA. Williams, D. Bartholomew, MI. Van Allen, A. Parikh, L. Zhang, BL. Wu, RE. Pyatt, S. Schwartz, LG. Shaffer, BB. de Vries, JF. Gusella, and SH. Elsea. Assessment of 2q23.1 microdeletion syndrome implicates mbd5 as a single causal locus of intellectual disability, epilepsy, and autism spectrum disorder. Am J Hum Genet, 89(4):551–563, 2011. PMCID: PMC3188839, PMID: 21981781.

[236] SV. Mullegama, JA. Rosenfeld, C. Orellana, BW. van Bon, S. Halbach, EA. Repnikova, L. Brick, C. Li, L. Dupuis, M. Rosello, S. Aradhya, DJ. Stavropoulos, K. Manickam, E. Mitchell, JC. Hodge, ME. Talkowski, JF. Gusella, K. Keller, J. Zonana, S. Schwartz, RE. Pyatt, DJ. Waggoner, LG. Shaffer, AE. Lin, BB. de Vries, R. Mendoza-Londono, and SH. Elsea. Reciprocal deletion and duplication at 2q23.1 indicates a role for mbd5 in autism spectrum disorder. Eur J Hum Genet, 22(1):57–63, 2014. PMCID: PMC3865402, PMID: 23632792.

[237] CS. Richards, S. Bale, DB. Bellissimo, S. Das, WW. Grody, MR. Hegde, E. Lyon, and BE. Ward. Acmg recommendations for standards for interpretation and reporting of sequence variations: Revisions 2007. Genet Med, 10(4):294–300, 2008. PMID: 18414213.

[238] JMM. Howson, W. Zhao, DR. Barnes, WK. Ho, R. Young, DS. Paul, LL. Waite, DF. Freitag, EB. Fauman, EL. Salfati, BB. Sun, JD. Eicher, AD. Johnson, WHH. Sheu, SF. Nielsen, WY. Lin, P. Surendran, A. Malarstig, JB. Wilk, A. Tybjærg-Hansen, KL. Rasmussen, PR. Kamstrup, P. Deloukas, J. Erdmann, S. Kathiresan, NJ. Samani, H. Schunkert, H. Watkins, R. Do, DJ. Rader, JA. Johnson, SL. Hazen, AA. Quyyumi, JA. Spertus, CJ. Pepine, N. Franceschini, A. Justice, AP. Reiner, S. Buyske, LA. Hindorff, CL. Carty, KE. North, C. Kooperberg, E. Boerwinkle, K. Young, M. Graff, U. Peters, D. Absher, CA. Hsiung, WJ. Lee, KD. Taylor, YH. Chen, IT. Lee, X. Guo, RH. Chung, YJ. Hung, JI. Rotter, JJ. Juang, T. Quertermous, TD. Wang, A. Rasheed, P. Frossard, DS. Alam, AAS. Majumder, E. Di Angelantonio, R. Chowdhury, YI. Chen, BG. Nordestgaard, TL. Assimes, J. Danesh, AS. Butterworth, and D. Saleheen. Fifteen new risk loci for coronary artery disease highlight arterial-wall-specific mechanisms. Nat Genet, 49(7):1113–1119, 2017. PMCID: PMC5555387, PMID: 28530674.

[239] SK. Ku, EJ. Yang, KS. Song, and JS. Bae. Rosmarinic acid down-regulates endothelial protein c receptor shedding in vitro and in vivo. Food Chem Toxicol, 59:311–315, 2013. PMID: 23774263.

[240] L. Shan, M. Yu, and EG. Snyderwine. Global gene expression profiling of chemically induced rat mammary gland carcinomas and adenomas. Toxicol Pathol, 33(7):768–775, 2005. PMID: 16316942.

[241] SJ. Hsiao, MA. Karajannis, D. Diolaiti, MM. Mansukhani, JG. Bender, AL. Kung, and JH. Garvin. A novel, potentially targetable tmem106b-braf fusion in pleomorphic xanthoastrocytoma. Cold Spring Harb Mol Case Stud, 3(2):a001396, 2017. PMCID: PMC5334470, PMID: 28299358.

[242] D. Pehlivan, CR. Beck, Y. Okamoto, T. Harel, ZH. Akdemir, SN. Jhangiani, MA. Withers, MT. Goksungur, CM. Carvalho, D. Czesnik, C. Gonzaga-Jauregui, W. Wiszniewski, DM. Muzny, RA. Gibbs, B. Rautenstrauss, MW. Sereda, and JR. Lupski. The role of combined snv and cnv burden in patients with distal symmetric polyneuropathy. Genet Med, 18(5):443–451, 2016. PMCID: PMC5322766, PMID: 26378787.

[243] R. Altara, FA. Zouein, RD. Brandão, SN. Bajestani, A. Cataliotti, and GW. Booz. In silico analysis of differential gene expression in three common rat models of diastolic dysfunction. Front Cardiovasc Med, 5:11, 2018. PMCID: PMC5850854, PMID: 29556499.

[244] J. Wang, PR. Ahimaz, S. Hashemifar, J. Khlevner, JA. Picoraro, W. Middlesworth, MM. Elfiky, J. Que, Y. Shen, and WK. Chung. Novel candidate genes in esophageal atresia/tracheoesophageal fistula identified by exome sequencing. Eur J Hum Genet, 29(1):122–130, 2021. PMCID: PMC7852873, PMID: 32641753.

[245] Y. Yu, K. Schleich, B. Yue, S. Ji, P. Lohneis, K. Kemper, MR. Silvis, N. Qutob, E. van Rooijen, M. Werner-Klein, L. Li, D. Dhawan, S. Meierjohann, M. Reimann, A. Elkahloun,

S. Treitschke, B. Dörken, C. Speck, FA. Mallette, LI. Zon, SL. Holmen, DS. Peeper, Y. Samuels, CA. Schmitt, and S. Lee. Targeting the senescence-overriding cooperative activity of structurally unrelated h3k9 demethylases in melanoma. Cancer Cell, 33(2):322–336, 2018. PMCID: PMC5977991, PMID: 29438700.

[246] AP. Trifa, C. Bănescu, AS. Bojan, CM. Voina, S;. Popa, S. Vis, an, AD. Ciubean, F. Tripon, D. Dima, VM. Popov, SC. Vesa, M. Andreescu, T. Török-Vistai, RG. Mihăilă, N. Berbec, I. Macarie, A. Coliţă, M. Iordache, AC. Cătană, MF. Farcas, , C. Tomuleasa, K. Vasile, C. Truică, A. Todincă, L. Pop-Muntean, R. Manolache, H. Bumbea, AM. Vlădăreanu, M. Gaman, CM. Ciufu, and RA. Popp. Mecom, hbs1l-myb, thrb-rarb, jak2, and tert polymorphisms defining the genetic predisposition to myeloproliferative neoplasms: A study on 939 patients. Am J Hematol, 93(1):100–106, 2018. PMID: 29047144.

[247] D. Pietra, A. Brisci, E. Rumi, S. Boggi, C. Elena, A. Pietrelli, R. Bordoni, M. Ferrari, F. Passamonti, G. De Bellis, L. Cremonesi, and M. Cazzola. Deep sequencing reveals double mutations in cis of mpl exon 10 in myeloproliferative neoplasms. Haematologica, 96(4):607–611, 2011. PMCID: PMC3069239, PMID: 21228032.

[248] Y. Pikman, BH. Lee, T. Mercher, E. McDowell, BL. Ebert, M. Gozo, A. Cuker, G. Wernig, S. Moore, I. Galinsky, DJ. DeAngelo, JJ. Clark, SJ. Lee, TR. Golub, M. Wadleigh, DG. Gilliland, and RL. Levine. Mplw515l is a novel somatic activating mutation in myelofibrosis with myeloid metaplasia. PLoS Med, 3(7):e270, 2006. PMCID: PMC1502153, PMID: 16834459.

[249] AD. Pardanani, RL. Levine, T. Lasho, Y. Pikman, RA. Mesa, M. Wadleigh, DP. Steensma, MA. Elliott, AP. Wolanskyj, WJ. Hogan, RF. McClure, MR. Litzow, DG. Gilliland, and A. Tefferi. Mpl515 mutations in myeloproliferative and other myeloid disorders: a study of 1182 patients. Blood, 108(10):3472–3476, 2006. PMID: 16868251.

[250] J. Kota, N. Caceres, and SN. Constantinescu. Aberrant signal transduction pathways in myeloproliferative neoplasms. Leukemia, 22(10):1828–1840, 2008. PMID: 18769448.

[251] AC. Glembotsky, L. Korin, PR. Lev, CD. Chazarreta, RF. Marta, FC. Molinas, and PG. Heller. Screening for mpl mutations in essential thrombocythemia and primary myelofibrosis: normal mpl expression and absence of constitutive stat3 and stat5 activation in mplw515l-positive platelets. Eur J Haematol, 84(5):398–405, 2010. PMID: 20113333.

[252] W. Ma, X. Zhang, X. Wang, Z. Zhang, CH. Yeh, J. Uyeji, and M. Albitar. Mpl mutation profile in jak2 mutation-negative patients with myeloproliferative disorders. Diagn Mol Pathol, 20(1):34–39, 2011. PMID: 21326037.

[253] Z. Wu, X. Zhang, X. Xu, Y. Chen, T. Hu, Z. Kang, S. Li, H. Wang, W. Liu, X. Ma, and M. Guan. The mutation profile of jak2 and calr in chinese han patients with philadelphia chromosome-negative myeloproliferative neoplasms. J Hematol Oncol, 7:48, 2014. PMCID: PMC4223390, PMID: 25023898.

[254] J. Yu, Z. Huang, and JY. Fan. [akt is a therapeutic target in myeloproliferative neoplasms]. Zhongguo Shi Yan Xue Ye Xue Za Zhi, 25(4):1105–1112, 2017. PMID:

28823277.

[255] J. Xie, X. Chen, F. Gao, R. Hou, T. Tian, Y. Zhang, L. Fan, J. Hu, G. Zhu, W. Yang, and H. Wang. Two activating mutations of mpl in triple-negative myeloproliferative neoplasms. Cancer Med, 8(11):5254–5263, 2019. PMCID: PMC6718619, PMID: 31294534.

[256] X. Qin, Y. Guo, H. Du, Y. Zhong, J. Zhang, X. Li, H. Yu, Z. Zhang, Z. Jia, and Z. Li. Comparative analysis for glycopatterns and complex-type n-glycans of glycoprotein in sera from chronic hepatitis b- and c-infected patients. Front Physiol, 8:596, 2017. PMCID: PMC5566988, PMID: 28871230.

[257] AR. Moliterno, DM. Williams, LI. Gutierrez-Alamillo, R. Salvatori, RG. Ingersoll, and JL. Spivak. Mpl baltimore: a thrombopoietin receptor polymorphism associated with thrombocytosis. Proc Natl Acad Sci U S A, 101(31): 11444–11447, 2004. PMCID: PMC509220, PMID: 15269348.

[258] DL. Bodian, JN. McCutcheon, P. Kothiyal, KC. Huddleston, RK. Iyer, JG. Vockley, and JE. Niederhuber. Germline variation in cancer-susceptibility genes in a healthy, ancestrally diverse cohort: implications for individual genome sequencing. PLoS One, 9(4):e94554, 2014. PMCID: PMC3984285, PMID: 24728327.

[259] DS. Kim, AA. Burt, JE. Ranchalis, B. Wilmot, JD. Smith, KE. Patterson, BP. Coe, YK. Li, MJ. Bamshad, M. Nikolas, EE. Eichler, JM. Swanson, JT. Nigg, DA. Nickerson, and GP. Jarvik. Sequencing of sporadic attention-deficit hyperactivity disorder (adhd) identifies novel and potentially pathogenic de novo variants and excludes overlap with genes associated with autism spectrum disorder. Am J Med Genet B Neuropsychiatr Genet, 174(4):381–389, 2017. PMCID: PMC5467442, PMID: 28332277.

[260] MS. Leduc, Z. Niu, W. Bi, W. Zhu, I. Miloslavskaya, T. Chiang, H. Streff, JR. Seavitt, SA. Murray, C. Eng, A. Chan, Y. Yang, and SR. Lalani. Cript exonic deletion and a novel missense mutation in a female with short stature, dysmorphic features, microcephaly, and pigmentary abnormalities. Am J Med Genet A, 170(8): 2206–2211, 2016. PMCID: PMC5725961, PMID: 27250922.

[261] Y. Ciribilli, P. Singh, A. Inga, and J. Borlak. c-myc targeted regulators of cell metabolism in a transgenic mouse model of papillary lung adenocarcinoma. Oncotarget, 7(40):65514–65539, 2016. PMCID: PMC5323172, PMID: 27602772.

[262] K. Warnatz, U. Salzer, M. Rizzi, B. Fischer, S. Gutenberger, J. Böhm, AK. Kienzler, Q. Pan-Hammarström, L. Hammarström, M. Rakhmanov, M. Schlesier, B. Grimbacher, HH. Peter, and H. Eibel. B-cell activating factor receptor deficiency is associated with an adult-onset antibody deficiency syndrome in humans. Proc Natl Acad Sci U S A, 106(33):13945–13950, 2009. PMCID: PMC2722504, PMID: 19666484.

[263] F. Vetrini, S. McKee, JA. Rosenfeld, M. Suri, AM. Lewis, KM. Nugent, E. Roeder, RO. Littlejohn, S. Holder, W. Zhu, JT. Alaimo, B. Graham, JM. Harris, JB. Gibson, M. Pastore, KL. McBride, M. Komara, L. Al-Gazali, A. Al Shamsi, EA. Fanning, KJ. Wierenga, DA. Scott, Z. Ben-Neriah, V. Meiner, H. Cassuto, O. Elpeleg, JL. Holder, LC. Burrage, LH. Seaver, L. Van Maldergem, S. Mahida, JS. Soul, M. Marlatt, L. Matyakhina, J. Vogt, JA. Gold, SM. Park, V. Varghese, AK. Lampe, A. Kumar, M. Lees, M. Holder-

Espinasse, V. McConnell, B. Bernhard, E. Blair, V. Harrison, DM. Muzny, RA. Gibbs, SH. Elsea, JE. Posey, W. Bi, S. Lalani, F. Xia, Y. Yang, CM. Eng, JR. Lupski, and P. Liu. De novo and inherited tcf20 pathogenic variants are associated with intellectual disability, dysmorphic features, hypotonia, and neurological impairments with similarities to smith-magenis syndrome. Genome Med, 11(1):12, 2019. PMCID: PMC6393995, PMID: 30819258.

[264] Y. Huang, L. Yang, J. Wang, F. Yang, Y. Xiao, R. Xia, X. Yuan, and M. Yan. Twelve novel atm mutations identified in chinese ataxia telangiectasia patients. Neuromolecular Med, 15(3):536–540, 2013. PMCID: PMC3732755, PMID: 23807571.

[265] MJ. Podralska, A. Stembalska, R. Ślezak, ˛ A. Lewandowicz-Uszyńska, B. Pietrucha, S. Kołtan, J. Wigowska-Sowińska, J. Pilch, M. Mosor, I. Ziółkowska-Suchanek, A. Dzikiewicz-Krawczyk, and R. Słomski. Ten new atm alterations in polish patients with ataxia-telangiectasia. Mol Genet Genomic Med, 2(6):504–511, 2014. PMCID: PMC4303220, PMID: 25614872.

[266] SR. McWhinney, RT. Pilarski, SR. Forrester, MC. Schneider, MM. Sarquis, EP. Dias, and C. Eng. Large germline deletions of mitochondrial complex ii subunits sdhb and sdhd in hereditary paraganglioma. J Clin Endocrinol Metab, 89(11):5694–5699, 2004. PMID: 15531530.

[267] JA. Wambach, GM. Stettner, TB. Haack, K. Writzl, A. Škofljanec, A. Maver, F. Munell, S. Ossowski, M. Bosio, DJ. Wegner, M. Shinawi, D. Baldridge, B. Alhaddad, TM. Strom, DK. Grange, E. Wilichowski, R. Troxell, J. Collins, BB. Warner, RE. Schmidt, A. Pestronk, FS. Cole, and R. Steinfeld. Survival among children with "lethal" congenital contracture syndrome 11 caused by novel mutations in the gliomedin gene (gldn). Hum Mutat, 38(11):1477–1484, 2017. PMCID: PMC5638693, PMID: 28726266.

[268] M. Pergande, S. Motameny, O. Özdemir, M. Kreutzer, H. Wang, HS. Daimagüler, K. Becker, M. Karakaya, H. Ehrhardt, N. Elcioglu, S. Ostojic, CM. Chao, A. Kawalia, O. Duman, A. Koy, A. Hahn, J. Reimann, K. Schoner, A. Schänzer, JH. Westhoff, EMC. Schwaibold, M. Cossee, M. Imbert-Bouteille, H. von Pein, G. Haliloglu, H. Topaloglu, J. Altmüller, P. Nürnberg, H. Thiele, R. Heller, and S. Cirak. The genomic and clinical landscape of fetal akinesia. Genet Med, 22(3):511–523, 2020. PMID: 31680123.

[269] AI. den Hollander, RK. Koenekoop, MD. Mohamed, HH. Arts, K. Boldt, KV. Towns, T. Sedmak, M. Beer, K. Nagel-Wolfrum, M. McKibbin, S. Dharmaraj, I. Lopez, L. Ivings, GA. Williams, K. Springell, CG. Woods, H. Jafri, Y. Rashid, TM. Strom, B. van der Zwaag, I. Gosens, FF. Kersten, E. van Wijk, JA. Veltman, MN. Zonneveld, SE. van Beersum, IH. Maumenee, U. Wolfrum, ME. Cheetham, M. Ueffing, FP. Cremers, CF. Inglehearn, and R. Roepman. Mutations in lca5, encoding the ciliary protein lebercilin, cause leber congenital amaurosis. Nat Genet, 39(7):889–895, 2007. PMID: 17546029.

[270] DS. Mackay, AD. Borman, R. Sui, LI. van den Born, EL. Berson, LA. Ocaka, AE. Davidson, JR. Heckenlively, K. Branham, H. Ren, I. Lopez, M. Maria, M. Azam, A. Henkes, E. Blokland, R. Qamar, AR. Webster, FPM. Cremers, AT. Moore, RK. Koenekoop, S. Andreasson, E. de Baere, J. Bennett, GJ. Chader, W. Berger, I. Golovleva, J. Greenberg, AI. den Hollander, CCW. Klaver, BJ. Klevering, B. Lorenz, MN. Preising, R. Ramsear, L. Roberts, R. Roepman, K. Rohrschneider, and B. Wissinger.

Screening of a large cohort of leber congenital amaurosis and retinitis pigmentosa patients identifies novel lca5 mutations and new genotype-phenotype correlations. Hum Mutat, 34(11):1537–1546, 2013. PMCID: PMC4337959, PMID: 23946133.

[271] KJ. Carss, G. Arno, M. Erwood, J. Stephens, A. Sanchis-Juan, S. Hull, K. Megy, D. Grozeva, E. Dewhurst, S. Malka, V. Plagnol, C. Penkett, K. Stirrups, R. Rizzo, G. Wright, D. Josifova, M. Bitner-Glindzicz, RH. Scott, E. Clement, L. Allen, R. Armstrong, AF. Brady, J. Carmichael, M. Chitre, RHH. Henderson, J. Hurst, RE. MacLaren, E. Murphy, J. Paterson, E. Rosser, DA. Thompson, E. Wakeling, WH. Ouwehand, M. Michaelides, AT. Moore, AR. Webster, and FL. Raymond. Comprehensive rare variant analysis via whole-genome sequencing to determine the molecular pathology of inherited retinal disease. Am J Hum Genet, 100(1):75–90, 2017. PMCID: PMC5223092, PMID: 28041643.

[272] SG. Jacobson, TS. Aleman, AV. Cideciyan, A. Sumaroka, SB. Schwartz, EA. Windsor, M. Swider, W. Herrera, and EM. Stone. Leber congenital amaurosis caused by lebercilin (lca5) mutation: retained photoreceptors adjacent to retinal disorganization. Mol Vis, 15:1098–1106, 2009. PMCID: PMC2690955, PMID: 19503738.

[273] MP. Adam, J. Feldman, GM. Mirzaa, RA. Pagon, SE. Wallace, LJH. Bean, KW. Gripp, A. Amemiya, RG. Weleber, PJ. Francis, KM. Trzupek, and C. Beattie. 1993. PMCID: PMC3647373, PMID: 20301475.

[274] K. Boldt, DA. Mans, J. Won, J. van Reeuwijk, A. Vogt, N. Kinkl, SJ. Letteboer, WL. Hicks, RE. Hurd, JK. Naggert, Y. Texier, AI. den Hollander, RK. Koenekoop, J. Bennett, FP. Cremers, CJ. Gloeckner, PM. Nishina, R. Roepman, and M. Ueffing. Disruption of intraflagellar protein transport in photoreceptor cilia causes leber congenital amaurosis in humans and mice. J Clin Invest, 121(6):2169–2180, 2011. PMCID: PMC3104757, PMID: 21606596.

[275] KD. Farwell, L. Shahmirzadi, D. El-Khechen, Z. Powis, EC. Chao, B. Tippin Davis, RM. Baxter, W. Zeng, C. Mroske, MC. Parra, SK. Gandomi, I. Lu, X. Li, H. Lu, HM. Lu, D. Salvador, D. Ruble, M. Lao, S. Fischbach, J. Wen, S. Lee, A. Elliott, CL. Dunlop, and S. Tang. Enhanced utility of family-centered diagnostic exome sequencing with inheritance model-based analysis: results from 500 unselected families with undiagnosed genetic conditions. Genet Med, 17(7):578–586, 2015. PMID: 25356970.

[276] N. Akizu, V. Cantagrel, MS. Zaki, L. Al-Gazali, X. Wang, RO. Rosti, E. Dikoglu, AB. Gelot, B. Rosti, KK. Vaux, EM. Scott, JL. Silhavy, J. Schroth, B. Copeland, AE. Schaffer, PL. Gordts, JD. Esko, MD. Buschman, SJ. Field, G. Napolitano, GM. Abdel-Salam, RK. Ozgul, MS. Sagıroglu, M. Azam, S. Ismail, M. Aglan, L. Selim, IG. Mahmoud, S. Abdel-Hadi, AE. Badawy, AA. Sadek, F. Mojahedi, H. Kayserili, A. Masri, L. Bastaki, S. Temtamy, U. Müller, I. Desguerre, JL. Casanova, A. Dursun, M. Gunel, SB. Gabriel, P. de Lonlay, and JG. Gleeson. Biallelic mutations in snx14 cause a syndromic form of cerebellar atrophy and lysosome-autophagosome dysfunction. Nat Genet, 47(5):528–534, 2015. PMCID: PMC4414867, PMID: 25848753.

[277] AS. Ho, K. Kannan, DM. Roy, LG. Morris, I. Ganly, N. Katabi, D. Ramaswami, LA. Walsh, S. Eng, JT. Huse, J. Zhang, I. Dolgalev, K. Huberman, A. Heguy, A. Viale, M. Drobnjak, MA. Leversha, CE. Rice, B. Singh, NG. Iyer, CR. Leemans, E. Bloemena, RL. Ferris,

RR. Seethala, BE. Gross, Y. Liang, R. Sinha, L. Peng, BJ. Raphael, S. Turcan, Y. Gong, N. Schultz, S. Kim, S. Chiosea, JP. Shah, C. Sander, W. Lee, and TA. Chan. The mutational landscape of adenoid cystic carcinoma. Nat Genet, 45(7):791–798, 2013. PMCID: PMC3708595, PMID: 23685749.

[278] K. Kataoka, Y. Nagata, A. Kitanaka, Y. Shiraishi, T. Shimamura, J. Yasunaga, Y. Totoki, K. Chiba, A. Sato-Otsubo, G. Nagae, R. Ishii, S. Muto, S. Kotani, Y. Watatani, J. Takeda, M. Sanada, H. Tanaka, H. Suzuki, Y. Sato, Y. Shiozawa, T. Yoshizato, K. Yoshida, H. Makishima, M. Iwanaga, G. Ma, K. Nosaka, M. Hishizawa, H. Itonaga, Y. Imaizumi, W. Munakata, H. Ogasawara, T. Sato, K. Sasai, K. Muramoto, M. Penova, T. Kawaguchi, H. Nakamura, N. Hama, K. Shide, Y. Kubuki, T. Hidaka, T. Kameda, T. Nakamaki, K. Ishiyama, S. Miyawaki, SS. Yoon, K. Tobinai, Y. Miyazaki, A. Takaori-Kondo, F. Matsuda, K. Takeuchi, O. Nureki, H. Aburatani, T. Watanabe, T. Shibata, M. Matsuoka, S. Miyano, K. Shimoda, and S. Ogawa. Integrated molecular analysis of adult t cell leukemia/lymphoma. Nat Genet, 47(11):1304–1315, 2015. PMID: 26437031.

[279] JD. Campbell, A. Alexandrov, J. Kim, J. Wala, AH. Berger, CS. Pedamallu, SA. Shukla, G. Guo, AN. Brooks, BA. Murray, M. Imielinski, X. Hu, S. Ling, R. Akbani, M. Rosenberg, C. Cibulskis, A. Ramachandran, EA. Collisson, DJ. Kwiatkowski, MS. Lawrence, JN. Weinstein, RG. Verhaak, CJ. Wu, PS. Hammerman, AD. Cherniack, G. Getz, MN. Artyomov, R. Schreiber, R. Govindan, and M. Meyerson. Distinct patterns of somatic genome alterations in lung adenocarcinomas and squamous cell carcinomas. Nat Genet, 48(6):607–616, 2016. PMCID: PMC4884143, PMID: 27158780.

[280] RN. Doan, ET. Lim, S. De Rubeis, C. Betancur, DJ. Cutler, AG. Chiocchetti, LM. Overman, A. Soucy, S. Goetze, CM. Freitag, MJ. Daly, CA. Walsh, JD. Buxbaum, and TW. Yu. Recessive gene disruptions in autism spectrum disorder. Nat Genet, 51(7):1092–1098, 2019. PMCID: PMC6629034, PMID: 31209396.

[281] K. Niizuma, H. Endo, C. Nito, DJ. Myer, and PH. Chan. Potential role of puma in delayed death of hippocampal ca1 neurons after transient global cerebral ischemia. Stroke, 40(2):618–625, 2009. PMCID: PMC2631621, PMID: 19095966.

[282] SP. Garrison, JR. Jeffers, C. Yang, JA. Nilsson, MA. Hall, JE. Rehg, W. Yue, J. Yu, L. Zhang, M. Onciu, JT. Sample, JL. Cleveland, and GP. Zambetti. Selection against puma gene expression in myc-driven b-cell lymphomagenesis. Mol Cell Biol, 28(17):5391–5402, 2008. PMCID: PMC2519737, PMID: 18573879.

[283] H. Wang, H. Qian, J. Yu, X. Zhang, L. Zhang, M. Fu, X. Liang, Q. Zhan, and C. Lin. Administration of puma adenovirus increases the sensitivity of esophageal cancer cells to anticancer drugs. Cancer Biol Ther, 5(4): 380–385, 2006. PMID: 16481741.

[284] SY. Shi, CT. Luk, SA. Schroer, MJ. Kim, DW. Dodington, T. Sivasubramaniyam, L. Lin, EP. Cai, SY. Lu, KU. Wagner, RP. Bazinet, and M. Woo. Janus kinase 2 (jak2) dissociates hepatosteatosis from hepatocellular carcinoma in mice. J Biol Chem, 292(9):3789–3799, 2017. PMCID: PMC5339761, PMID: 28100771.

[285] A. Driss, S. Noguchi, R. Amouri, M. Kefi, T. Sasaki, K. Sugie, S. Souilem, YK. Hayashi, N. Shimizu, S. Minoshima, J. Kudoh, F. Hentati, and I. Nishino. Fukutin-related protein gene mutated in the original kindred limb-girdle md 2i. Neurology, 60(8):1341–1344,

2003. PMID: 12707439.

[286] S. Quijano-Roy, I. Martí-Carrera, S. Makri, M. Mayer, S. Maugenre, P. Richard, C. Berard, L. Viollet, B. Leheup, P. Guicheney, JM. Pinard, B. Estournet, and RY. Carlier. Brain mri abnormalities in muscular dystrophy due to fkrp mutations. Brain Dev, 28(4):232–242, 2006. PMID: 16368217.

[287] XQ. Rosales, SJ. Moser, T. Tran, B. McCarthy, N. Dunn, P. Habib, OP. Simonetti, JR. Mendell, and SV. Raman. Cardiovascular magnetic resonance of cardiomyopathy in limb girdle muscular dystrophy 2b and 2i. J Cardiovasc Magn Reson, 13(1):39, 2011. PMCID: PMC3170213, PMID: 21816046.

[288] P. Hafner, U. Bonati, A. Fischmann, J. Schneider, S. Frank, DJ. Morris-Rosendahl, A. Dumea, K. Heinimann, and D. Fischer. Skeletal muscle mri of the lower limbs in congenital muscular dystrophy patients with novel pomt1 and pomt2 mutations. Neuromuscul Disord, 24(4):321–324, 2014. PMID: 24556424.

[289] A. Abulí, M. Boada, B. Rodríguez-Santiago, B. Coroleu, A. Veiga, L. Armengol, PN. Barri, LA. Pérez-Jurado, and X. Estivill. Ngs-based assay for the identification of individuals carrying recessive genetic mutations in reproductive medicine. Hum Mutat, 37(6):516–523, 2016. PMID: 26990548.

[290] M. Brockington, Y. Yuva, P. Prandini, SC. Brown, S. Torelli, MA. Benson, R. Herrmann, LV. Anderson, R. Bashir, JM. Burgunder, S. Fallet, N. Romero, M. Fardeau, V. Straub, G. Storey, C. Pollitt, I. Richard, CA. Sewry, K. Bushby, T. Voit, DJ. Blake, and F. Muntoni. Mutations in the fukutin-related protein gene (fkrp) identify limb girdle muscular dystrophy 2i as a milder allelic variant of congenital muscular dystrophy mdc1c. Hum Mol Genet, 10(25):2851–2859, 2001. PMID: 11741828.

[291] E. Mercuri, M. Brockington, V. Straub, S. Quijano-Roy, Y. Yuva, R. Herrmann, SC. Brown, S. Torelli, V. Dubowitz, DJ. Blake, NB. Romero, B. Estournet, CA. Sewry, P. Guicheney, T. Voit, and F. Muntoni. Phenotypic spectrum associated with mutations in the fukutin-related protein gene. Ann Neurol, 53(4):537–542, 2003. PMID: 12666124.

[292] F. de Paula, N. Vieira, A. Starling, LU. Yamamoto, B. Lima, R. de Cássia Pavanello, M. Vainzof, V. Nigro, and M. Zatz. Asymptomatic carriers for homozygous novel mutations in the fkrp gene: the other end of the spectrum. Eur J Hum Genet, 11(12):923–930, 2003. PMID: 14647208.

[293] MC. Walter, JA. Petersen, R. Stucka, D. Fischer, R. Schröder, M. Vorgerd, A. Schroers, H. Schreiber, CO. Hanemann, U. Knirsch, A. Rosenbohm, A. Huebner, N. Barisic, R. Horvath, S. Komoly, P. Reilich, W. Müller-Felber, D. Pongratz, JS. Müller, EA. Auerswald, and H. Lochmüller. Fkrp (826c>a) frequently causes limb-girdle muscular dystrophy in german patients. J Med Genet, 41(4):e50, 2004. PMCID: PMC1735747, PMID: 15060126.

[294] CT. Esapa, RA. McIlhinney, and DJ. Blake. Fukutin-related protein mutations that cause congenital muscular dystrophy result in er-retention of the mutant protein in cultured cells. Hum Mol Genet, 14(2):295–305, 2005. PMID: 15574464.

[295] P. Frosk, CR. Greenberg, AA. Tennese, R. Lamont, E. Nylen, C. Hirst, D. Frappier, NM.

Roslin, M. Zaik, K. Bushby, V. Straub, M. Zatz, F. de Paula, K. Morgan, TM. Fujiwara, and K. Wrogemann. The most common mutation in fkrp causing limb girdle muscular dystrophy type 2i (lgmd2i) may have occurred only once and is present in hutterites and other populations. Hum Mutat, 25(1):38–44, 2005. PMID: 15580560.

[296] T. Müller, M. Krasnianski, R. Witthaut, M. Deschauer, and S. Zierz. Dilated cardiomyopathy may be an early sign of the c826a fukutin-related protein mutation. Neuromuscul Disord, 15(5):372–376, 2005. PMID: 15833432.

[297] M. Schwartz, JM. Hertz, ML. Sveen, and J. Vissing. Lgmd2i presenting with a characteristic duchenne or becker muscular dystrophy phenotype. Neurology, 64(9):1635–1637, 2005. PMID: 15883334.

[298] P. Frosk, MR. Del Bigio, K. Wrogemann, and CR. Greenberg. Hutterite brothers both affected with two forms of limb girdle muscular dystrophy: Lgmd2h and lgmd2i. Eur J Hum Genet, 13(8):978–982, 2005. PMID: 15886712.

[299] ML. Sveen, M. Schwartz, and J. Vissing. High prevalence and phenotype-genotype correlations of limb girdle muscular dystrophy type 2i in denmark. Ann Neurol, 59(5):808–815, 2006. PMID: 16634037.

[300] C. Gaul, M. Deschauer, C. Tempelmann, S. Vielhaber, HU. Klein, HJ. Heinze, S. Zierz, and F. Grothues. Cardiac involvement in limb-girdle muscular dystrophy 2i : conventional cardiac diagnostic and cardiovascular magnetic resonance. J Neurol, 253(10):1317–1322, 2006. PMID: 16786213.

[301] E. Keramaris-Vrantsis, PJ. Lu, T. Doran, A. Zillmer, J. Ashar, CT. Esapa, MA. Benson, DJ. Blake, J. Rosenfeld, and QL. Lu. Fukutin-related protein localizes to the golgi apparatus and mutations lead to mislocalization in muscle in vivo. Muscle Nerve, 36(4):455–465, 2007. PMID: 17554798.

[302] A. D'Amico, S. Petrini, F. Parisi, A. Tessa, P. Francalanci, G. Grutter, FM. Santorelli, and E. Bertini. Heart transplantation in a child with lgmd2i presenting as isolated dilated cardiomyopathy. Neuromuscul Disord, 18(2): 153–155, 2008. PMID: 18060779.

[303] P. Reilich, JA. Petersen, S. Vielhaber, C. Mawrin, C. Schneider-Gold, C. Sommer, K. Reiners, M. Deschauer, D. Pongratz, H. Lochmüller, and MC. Walter. Lgmd 2i due to the common mutation 826c>a in the fkrp gene presenting as myopathy with vacuoles and paired-helical filaments. Acta Myol, 25(2):73–76, 2006. PMID: 18593008.

[304] K. Wahbi, C. Meune, el H. Hamouda, T. Stojkovic, P. Laforêt, HM. Bécane, B. Eymard, and D. Duboc. Cardiac assessment of limb-girdle muscular dystrophy 2i patients: an echography, holter ecg and magnetic resonance imaging study. Neuromuscul Disord, 18(8):650–655, 2008. PMID: 18639457.

[305] F. Hanisch, D. Grimm, S. Zierz, and M. Deschauer. Frequency of the fkrp mutation c.826c>a in isolated hyperckemia and in limb girdle muscular dystrophy type 2 in german patients. J Neurol, 257(2):300–301, 2010. PMID: 19820980.

[306] RR. Bennett, HE. Schneider, E. Estrella, S. Burgess, AS. Cheng, C. Barrett, V. Lip, PS. Lai, Y. Shen, BL. Wu, BT. Darras, AH. Beggs, and LM. Kunkel. Automated dna mutation

detection using universal conditions direct sequencing: application to ten muscular dystrophy genes. BMC Genet, 10:66, 2009. PMCID: PMC2781300, PMID: 19835634.

[307] PJ. Lu, A. Zillmer, X. Wu, H. Lochmuller, J. Vachris, D. Blake, YM. Chan, and QL. Lu. Mutations alter secretion of fukutin-related protein. Biochim Biophys Acta, 1802(2):253–258, 2010. PMID: 19900540.

[308] E. Stensland, S. Lindal, C. Jonsrud, T. Torbergsen, LA. Bindoff, M. Rasmussen, A. Dahl, F. Thyssen, and O. Nilssen. Prevalence, mutation spectrum and phenotypic variability in norwegian patients with limb girdle muscular dystrophy 2i. Neuromuscul Disord, 21(1):41–46, 2011. PMID: 20961759.

[309] KD. Mathews, CM. Stephan, K. Laubenthal, TL. Winder, DE. Michele, SA. Moore, and KP. Campbell. Myoglobinuria and muscle pain are common in patients with limb-girdle muscular dystrophy 2i. Neurology, 76 (2):194–195, 2011. PMCID: PMC3030231, PMID: 21220724.

[310] CJ. Bell, DL. Dinwiddie, NA. Miller, SL. Hateley, EE. Ganusova, J. Mudge, RJ. Langley, L. Zhang, CC. Lee, FD. Schilkey, V. Sheth, JE. Woodward, HE. Peckham, GP. Schroth, RW. Kim, and SF. Kingsmore. Carrier testing for severe childhood recessive diseases by next-generation sequencing. Sci Transl Med, 3(65):65ra4, 2011. PMCID: PMC3740116, PMID: 21228398.

[311] D. Renard, C. Fernandez, C. Bouchet-Seraphin, and P. Labauge. Cortical heterotopia in lgmd2i. Neuromuscul Disord, 22(5):443–444, 2012. PMID: 22264518.

[312] JX. Chong, R. Ouwenga, RL. Anderson, DJ. Waggoner, and C. Ober. A population-based study of autosomal-recessive disease-causing mutations in a founder population. Am J Hum Genet, 91(4):608–620, 2012. PMCID: PMC3484657, PMID: 22981120.

[313] KG. Hollingsworth, TA. Willis, MG. Bates, BJ. Dixon, H. Lochmüller, K. Bushby, J. Bourke, GA. MacGowan, and V. Straub. Subepicardial dysfunction leads to global left ventricular systolic impairment in patients with limb girdle muscular dystrophy 2i. Eur J Heart Fail, 15(9):986–994, 2013. PMID: 23576288.

[314] A. Blaeser, E. Keramaris, YM. Chan, S. Sparks, D. Cowley, X. Xiao, and QL. Lu. Mouse models of fukutin-related protein mutations show a wide range of disease phenotypes. Hum Genet, 132(8):923–934, 2013. PMID: 23591631.

[315] H. Duzkale, J. Shen, H. McLaughlin, A. Alfares, MA. Kelly, TJ. Pugh, BH. Funke, HL. Rehm, and MS. Lebo. A systematic approach to assessing the clinical significance of genetic variants. Clin Genet, 84(5):453–463, 2013. PMCID: PMC3995020, PMID: 24033266.

[316] M. Rasmussen, D. Scheie, N. Breivik, M. Mork, and S. Lindal. Clinical and muscle biopsy findings in norwegian paediatric patients with limb girdle muscular dystrophy 2i. Acta Paediatr, 103(5):553–558, 2014. PMID: 24447024.

[317] C. Qiao, CH. Wang, C. Zhao, P. Lu, H. Awano, B. Xiao, J. Li, Z. Yuan, Y. Dai, CB. Martin, J. Li, Q. Lu, and X. Xiao. Muscle and heart function restoration in a limb girdle muscular dystrophy 2i (lgmd2i) mouse model by systemic fkrp gene delivery. Mol Ther,

22(11):1890–1899, 2014. PMCID: PMC4429733, PMID: 25048216.

[318] LV. Schottlaender, A. Petzold, N. Wood, and H. Houlden. Diagnostic clues and manifesting carriers in fukutin-related protein (fkrp) limb-girdle muscular dystrophy. J Neurol Sci, 348(1-2):266–268, 2015. PMID: 25560911.

[319] J. Svahn, N. Streichenberger, O. Benveniste, R. Menassa, L. Michel, H. Fayolle, and P. Petiot. Significant response to immune therapies in a case of subacute necrotizing myopathy and fkrp mutations. Neuromuscul Disord, 25(11):865–868, 2015. PMID: 26363967.

[320] TO. Krag and J. Vissing. A new mouse model of limb-girdle muscular dystrophy type 2i homozygous for the common l276i mutation mimicking the mild phenotype in humans. J Neuropathol Exp Neurol, 74(12): 1137–1146, 2015. PMID: 26574668.

[321] SM. Yap, M. Farrell, J. Cryan, and S. Smyth. An irish case of limb-girdle muscular dystrophy 2i with structural eye involvement. Muscle Nerve, 54(3):509–510, 2016. PMID: 26833294.

[322] L. Ten Dam, WS. Frankhuizen, WHJP. Linssen, CS. Straathof, EH. Niks, K. Faber, A. Fock, JB. Kuks, E. Brusse, R. de Coo, N. Voermans, A. Verrips, JE. Hoogendijk, L. van der Pol, D. Westra, M. de Visser, AJ. van der Kooi, and I. Ginjaar. Autosomal recessive limb-girdle and miyoshi muscular dystrophies in the netherlands: The clinical and molecular spectrum of 244 patients. Clin Genet, 96(2):126–133, 2019. PMID: 30919934.

[323] M. Brockington, DJ. Blake, P. Prandini, SC. Brown, S. Torelli, MA. Benson, CP. Ponting, B. Estournet, NB. Romero, E. Mercuri, T. Voit, CA. Sewry, P. Guicheney, and F. Muntoni. Mutations in the fukutin-related protein gene (fkrp) cause a form of congenital muscular dystrophy with secondary laminin alpha2 deficiency and abnormal glycosylation of alpha-dystroglycan. Am J Hum Genet, 69(6):1198–1209, 2001. PMCID: PMC1235559, PMID: 11592034.

[324] CT. Esapa, MA. Benson, JE. Schröder, E. Martin-Rendon, M. Brockington, SC. Brown, F. Muntoni, S. Kröger, and DJ. Blake. Functional requirements for fukutin-related protein in the golgi apparatus. Hum Mol Genet, 11 (26):3319–3331, 2002. PMID: 12471058.

[325] D. Atac, S. Koller, JVM. Hanson, S. Feil, A. Tiwari, A. Bahr, L. Baehr, I. Magyar, R. Kottke, C. Gerth-Kahlert, and W. Berger. Atonal homolog 7 (atoh7) loss-of-function mutations in predominant bilateral optic nerve hypoplasia. Hum Mol Genet, 29(1):132–148, 2020. PMID: 31696227.

[326] C. Chen, X. Fu, D. Zhang, Y. Li, Y. Xie, Y. Li, and Y. Huang. Varied pathways of stage ia lung adenocarcinomas discovered by integrated gene expression analysis. Int J Biol Sci, 7(5):551–566, 2011. PMCID: PMC3088877, PMID: 21552421.

[327] NA. Bersinger, DM. Wunder, MH. Birkhäuser, and MD. Mueller. Gene expression in cultured endometrium from women with different outcomes following ivf. Mol Hum Reprod, 14(8):475–484, 2008. PMID: 18539642.

[328] LR. Shiow, DW. Roadcap, K. Paris, SR. Watson, IL. Grigorova, T. Lebet, J. An, Y. Xu, CN. Jenne, N. Föger, RU. Sorensen, CC. Goodnow, JE. Bear, JM. Puck, and JG. Cyster.

The actin regulator coronin 1a is mutant in a thymic egress-deficient mouse strain and in a patient with severe combined immunodeficiency. Nat Immunol, 9 (11):1307–1315, 2008. PMCID: PMC2672406, PMID: 18836449.

[329] A. Stray-Pedersen, E. Jouanguy, A. Crequer, AA. Bertuch, BS. Brown, SN. Jhangiani, DM. Muzny, T. Gambin, H. Sorte, G. Sasa, D. Metry, J. Campbell, MM. Sockrider, MK. Dishop, DM. Scollard, RA. Gibbs, EM. Mace, JS. Orange, JR. Lupski, JL. Casanova, and LM. Noroski. Compound heterozygous coro1a mutations in siblings with a mucocutaneous-immunodeficiency syndrome of epidermodysplasia verruciformis-hpv, molluscum contagiosum and granulomatous tuberculoid leprosy. J Clin Immunol, 34(7):871–890, 2014. PMCID: PMC4386834, PMID: 25073507.

[330] LA. Weiss, Y. Shen, JM. Korn, DE. Arking, DT. Miller, R. Fossdal, E. Saemundsen, H. Stefansson, MA. Ferreira, T. Green, OS. Platt, DM. Ruderfer, CA. Walsh, D. Altshuler, A. Chakravarti, RE. Tanzi, K. Stefansson, SL. Santangelo, JF. Gusella, P. Sklar, BL. Wu, and MJ. Daly. Association between microdeletion and microduplication at 16p11.2 and autism. N Engl J Med, 358(7):667–675, 2008. PMID: 18184952.

[331] DP. Moreira, K. Griesi-Oliveira, AL. Bossolani-Martins, NC. Lourenço, VN. Takahashi, KM. da Rocha, ES. Moreira, E. Vadasz, JG. Meira, D. Bertola, E. O’Halloran, TR. Magalhães, AC. Fett-Conte, and MR. Passos-Bueno. Investigation of 15q11-q13, 16p11.2 and 22q13 cnvs in autism spectrum disorder brazilian individuals with and without epilepsy. PLoS One, 9(9):e107705, 2014. PMCID: PMC4177849, PMID: 25255310.

[332] V. Krishnan and SL. Zeichner. Host cell gene expression during human immunodeficiency virus type 1 latency and reactivation and effects of targeting genes that are differentially expressed in viral latency. J Virol, 78(17): 9458–9473, 2004. PMCID: PMC506933, PMID: 15308739.

[333] DB. Sparrow, A. McInerney-Leo, ZS. Gucev, B. Gardiner, M. Marshall, PJ. Leo, DL. Chapman, V. Tasic, A. Shishko, MA. Brown, EL. Duncan, and SL. Dunwoodie. Autosomal dominant spondylocostal dysostosis is caused by mutation in tbx6. Hum Mol Genet, 22(8):1625–1631, 2013. PMID: 23335591.

[334] JR. Lupski, C. Gonzaga-Jauregui, Y. Yang, MN. Bainbridge, S. Jhangiani, CJ. Buhay, CL. Kovar, M. Wang, AC. Hawes, JG. Reid, C. Eng, DM. Muzny, and RA. Gibbs. Exome sequencing resolves apparent incidental findings and reveals further complexity of sh3tc2 variant alleles causing charcot-marie-tooth neuropathy. Genome Med, 5(6):57, 2013. PMCID: PMC3706849, PMID: 23806086.

[335] Y. Yang, DM. Muzny, JG. Reid, MN. Bainbridge, A. Willis, PA. Ward, A. Braxton, J. Beuten, F. Xia, Z. Niu, M. Hardison, R. Person, MR. Bekheirnia, MS. Leduc, A. Kirby, P. Pham, J. Scull, M. Wang, Y. Ding, SE. Plon, JR. Lupski, AL. Beaudet, RA. Gibbs, and CM. Eng. Clinical whole-exome sequencing for the diagnosis of mendelian disorders. N Engl J Med, 369(16):1502–1511, 2013. PMCID: PMC4211433, PMID: 24088041.

[336] N. Wu, X. Ming, J. Xiao, Z. Wu, X. Chen, M. Shinawi, Y. Shen, G. Yu, J. Liu, H. Xie, ZS. Gucev, S. Liu, N. Yang, H. Al-Kateb, J. Chen, J. Zhang, N. Hauser, T. Zhang, V. Tasic, P. Liu, X. Su, X. Pan, C. Liu, L. Wang, J. Shen, J. Shen, Y. Chen, T. Zhang, J. Zhang,

KW. Choy, J. Wang, Q. Wang, S. Li, W. Zhou, J. Guo, Y. Wang, C. Zhang, H. Zhao, Y. An, Y. Zhao, J. Wang, Z. Liu, Y. Zuo, Y. Tian, X. Weng, VR. Sutton, H. Wang, Y. Ming, S. Kulkarni, TP. Zhong, PF. Giampietro, SL. Dunwoodie, SW. Cheung, X. Zhang, L. Jin, JR. Lupski, G. Qiu, and F. Zhang. Tbx6 null variants and a common hypomorphic allele in congenital scoliosis. N Engl J Med, 372(4):341–350, 2015. PMCID: PMC4326244, PMID: 25564734.

[337] K. Wang, ST. Yuen, J. Xu, SP. Lee, HH. Yan, ST. Shi, HC. Siu, S. Deng, KM. Chu, S. Law, KH. Chan, AS. Chan, WY. Tsui, SL. Ho, AK. Chan, JL. Man, V. Foglizzo, MK. Ng, AS. Chan, YP. Ching, GH. Cheng, T. Xie, J. Fernandez, VS. Li, H. Clevers, PA. Rejto, M. Mao, and SY. Leung. Whole-genome sequencing and comprehensive molecular profiling identify new driver mutations in gastric cancer. Nat Genet, 46(6):573–582, 2014. PMID: 24816253.

[338] SL. Van Driest, O. Gakh, SR. Ommen, G. Isaya, and MJ. Ackerman. Molecular and functional characterization of a human frataxin mutation found in hypertrophic cardiomyopathy. Mol Genet Metab, 85(4):280–285, 2005. PMID: 15936968.

[339] ML. Jauslin, T. Meier, RA. Smith, and MP. Murphy. Mitochondria-targeted antioxidants protect friedreich ataxia fibroblasts from endogenous oxidative stress more effectively than untargeted antioxidants. FASEB J, 17(13): 1972–1974, 2003. PMID: 12923074.

[340] M. Pandolfo. Frataxin deficiency and mitochondrial dysfunction. Mitochondrion, 2(1-2):87–93, 2002. PMID: 16120311.

[341] RA. Schoenfeld, E. Napoli, A. Wong, S. Zhan, L. Reutenauer, D. Morin, AR. Buckpitt, F. Taroni, B. Lonnerdal, M. Ristow, H. Puccio, and GA. Cortopassi. Frataxin deficiency alters heme pathway transcripts and decreases mitochondrial heme metabolites in mammalian cells. Hum Mol Genet, 14(24):3787–3799, 2005. PMID: 16239244.

[342] KZ. Bencze, KC. Kondapalli, JD. Cook, S. McMahon, C. Millán-Pacheco, N. Pastor, and TL. Stemmler. The structure and function of frataxin. Crit Rev Biochem Mol Biol, 41(5):269–291, 2006. PMCID: PMC2859089, PMID: 16911956.

[343] RB. Wilson. Iron dysregulation in friedreich ataxia. Semin Pediatr Neurol, 13(3):166–175, 2006. PMID: 17101455.

[344] M. Rai, E. Soragni, CJ. Chou, G. Barnes, S. Jones, JR. Rusche, JM. Gottesfeld, and M. Pandolfo. Two new pimelic diphenylamide hdac inhibitors induce sustained frataxin upregulation in cells from friedreich's ataxia patients and in a mouse model. PLoS One, 5(1):e8825, 2010. PMCID: PMC2809102, PMID: 20098685.

[345] K. Kemp, E. Mallam, K. Hares, J. Witherick, N. Scolding, and A. Wilkins. Mesenchymal stem cells restore frataxin expression and increase hydrogen peroxide scavenging enzymes in friedreich ataxia fibroblasts. PLoS One, 6(10):e26098, 2011. PMCID: PMC3189234, PMID: 22016819.

[346] L. Li, L. Voullaire, C. Sandi, MA. Pook, PA. Ioannou, MB. Delatycki, and JP. Sarsero. Pharmacological screening using an fxn-egfp cellular genomic reporter assay for the therapy of friedreich ataxia. PLoS One, 8(2):e55940, 2013. PMCID: PMC3572186, PMID: 23418481.

[347] Y. Shen, MZ. McMackin, Y. Shan, A. Raetz, S. David, and G. Cortopassi. Frataxin deficiency promotes excess microglial dna damage and inflammation that is rescued by pj34. PLoS One, 11(3):e0151026, 2016. PMCID: PMC4783034, PMID: 26954031.

[348] L. Guo, Q. Wang, L. Weng, LA. Hauser, CJ. Strawser, AG. Rocha, A. Dancis, C. Mesaros, DR. Lynch, and IA. Blair. Liquid chromatography-high resolution mass spectrometry analysis of platelet frataxin as a protein biomarker for the rare disease friedreich's ataxia. Anal Chem, 90(3):2216–2223, 2018. PMCID: PMC5817373, PMID: 29272104.

[349] L. Guo, Q. Wang, L. Weng, LA. Hauser, CJ. Strawser, C. Mesaros, DR. Lynch, and IA. Blair. Characterization of a new n-terminally acetylated extra-mitochondrial isoform of frataxin in human erythrocytes. Sci Rep, 8(1): 17043, 2018. PMCID: PMC6242848, PMID: 30451920.

[350] Y. Gui, G. Guo, Y. Huang, X. Hu, A. Tang, S. Gao, R. Wu, C. Chen, X. Li, L. Zhou, M. He, Z. Li, X. Sun, W. Jia, J. Chen, S. Yang, F. Zhou, X. Zhao, S. Wan, R. Ye, C. Liang, Z. Liu, P. Huang, C. Liu, H. Jiang, Y. Wang, H. Zheng, L. Sun, X. Liu, Z. Jiang, D. Feng, J. Chen, S. Wu, J. Zou, Z. Zhang, R. Yang, J. Zhao, C. Xu, W. Yin, Z. Guan, J. Ye, H. Zhang, J. Li, K. Kristiansen, ML. Nickerson, D. Theodorescu, Y. Li, X. Zhang, S. Li, J. Wang, H. Yang, J. Wang, and Z. Cai. Frequent mutations of chromatin remodeling genes in transitional cell carcinoma of the bladder. Nat Genet, 43(9):875–878, 2011. PMCID: PMC5373841, PMID: 21822268.

[351] Z. Zelenko, L. Aghajanova, JC. Irwin, and LC. Giudice. Nuclear receptor, coregulator signaling, and chromatin remodeling pathways suggest involvement of the epigenome in the steroid hormone response of endometrium and abnormalities in endometriosis. Reprod Sci, 19(2):152–162, 2012. PMCID: PMC3343132, PMID: 22138541.

[352] DM. Park, J. Li, H. Okamoto, O. Akeju, SH. Kim, I. Lubensky, A. Vortmeyer, J. Dambrosia, RJ. Weil, EH. Oldfield, JK. Park, and Z. Zhuang. N-cor pathway targeting induces glioblastoma derived cancer stem cell differentiation. Cell Cycle, 6(4):467–470, 2007. PMID: 17312396.

[353] J. Armenia, SAM. Wankowicz, D. Liu, J. Gao, R. Kundra, E. Reznik, WK. Chatila, D. Chakravarty, GC. Han, I. Coleman, B. Montgomery, C. Pritchard, C. Morrissey, CE. Barbieri, H. Beltran, A. Sboner, Z. Zafeiriou, S. Miranda, CM. Bielski, AV. Penson, C. Tolonen, FW. Huang, D. Robinson, YM. Wu, R. Lonigro, LA. Garraway, F. Demichelis, PW. Kantoff, ME. Taplin, W. Abida, BS. Taylor, HI. Scher, PS. Nelson, JS. de Bono, MA. Rubin, CL. Sawyers, AM. Chinnaiyan, N. Schultz, and EM. Van Allen. The long tail of oncogenic drivers in prostate cancer. Nat Genet, 50(5):645–651, 2018. PMCID: PMC6107367, PMID: 29610475.

[354] S. Chen, W. Lu, MF. Yueh, E. Rettenmeier, M. Liu, M. Paszek, J. Auwerx, RT. Yu, RM. Evans, K. Wang, M. Karin, and RH. Tukey. Intestinal ncor1, a regulator of epithelial cell maturation, controls neonatal hyperbilirubinemia. Proc Natl Acad Sci U S A, 114(8):E1432–E1440, 2017. PMCID: PMC5338369, PMID: 28167773.

[355] A. Fujimoto, M. Furuta, Y. Totoki, T. Tsunoda, M. Kato, Y. Shiraishi, H. Tanaka, H. Taniguchi, Y. Kawakami, M. Ueno, K. Gotoh, S. Ariizumi, CP. Wardell, S. Hayami, T. Nakamura, H. Aikata, K. Arihiro, KA. Boroevich, T. Abe, K. Nakano, K. Maejima, A.

Sasaki-Oku, A. Ohsawa, T. Shibuya, H. Nakamura, N. Hama, F. Hosoda, Y. Arai, S. Ohashi, T. Urushidate, G. Nagae, S. Yamamoto, H. Ueda, K. Tatsuno, H. Ojima, N. Hiraoka, T. Okusaka, M. Kubo, S. Marubashi, T. Yamada, S. Hirano, M. Yamamoto, H. Ohdan, K. Shimada, O. Ishikawa, H. Yamaue, K. Chayama, S. Miyano, H. Aburatani, T. Shibata, and H. Nakagawa. Whole-genome mutational landscape and characterization of noncoding and structural mutations in liver cancer. Nat Genet, 48(5): 500–509, 2016. PMID: 27064257.

[356] ZH. Zhang, H. Yamashita, T. Toyama, Y. Yamamoto, T. Kawasoe, M. Ibusuki, S. Tomita, H. Sugiura, S. Kobayashi, Y. Fujii, and H. Iwase. Nuclear corepressor 1 expression predicts response to first-line endocrine therapy for breast cancer patients on relapse. Chin Med J (Engl), 122(15):1764–1768, 2009. PMID: 19781322.

[357] P. Szafranski, GK. Von Allmen, BH. Graham, AA. Wilfong, SH. Kang, JA. Ferreira, SJ. Upton, JB. Moeschler, W. Bi, JA. Rosenfeld, LG. Shaffer, S. Wai Cheung, P. Stankiewicz, and SR. Lalani. 6q22.1 microdeletion and susceptibility to pediatric epilepsy. Eur J Hum Genet, 23(2):173–179, 2015. PMCID: PMC4297903, PMID: 24824130.

[358] S. Hanein, I. Perrault, S. Gerber, G. Tanguy, F. Barbet, D. Ducroq, P. Calvas, H. Dollfus, C. Hamel, T. Lopponen, F. Munier, L. Santos, S. Shalev, D. Zafeiriou, JL. Dufier, A. Munnich, JM. Rozet, and J. Kaplan. Leber congenital amaurosis: comprehensive survey of the genetic heterogeneity, refinement of the clinical definition, and genotype-phenotype correlations as a strategy for molecular diagnosis. Hum Mutat, 23(4):306–317, 2004. PMID: 15024725.

[359] JC. Booij, RJ. Florijn, JB. ten Brink, W. Loves, F. Meire, MJ. van Schooneveld, PT. de Jong, and AA. Bergen. Identification of mutations in the aipl1, crb1, gucy2d, rpe65, and rpgrip1 genes in patients with juvenile retinitis pigmentosa. J Med Genet, 42(11):e67, 2005. PMCID: PMC1735944, PMID: 16272259.

[360] BH. Anderson, PR. Kasher, J. Mayer, M. Szynkiewicz, EM. Jenkinson, SS. Bhaskar, JE. Urquhart, SB. Daly, JE. Dickerson, J. O'Sullivan, EO. Leibundgut, J. Muter, GM. Abdel-Salem, R. Babul-Hirji, P. Baxter, A. Berger, L. Bonafé, JE. Brunstom-Hernandez, JA. Buckard, D. Chitayat, WK. Chong, DM. Cordelli, P. Ferreira, J. Fluss, EH. Forrest, E. Franzoni, C. Garone, SR. Hammans, G. Houge, I. Hughes, S. Jacquemont, PY. Jeannet, RJ. Jefferson, R. Kumar, G. Kutschke, S. Lundberg, CM. Lourenço, R. Mehta, S. Naidu, KK. Nischal, L. Nunes, K. Ounap, M. Philippart, P. Prabhakar, SR. Risen, R. Schiffmann, C. Soh, JB. Stephenson, H. Stewart, J. Stone, JL. Tolmie, MS. van der Knaap, JP. Vieira, CN. Vilain, EL. Wakeling, V. Wermenbol, A. Whitney, SC. Lovell, S. Meyer, JH. Livingston, GM. Baerlocher, GC. Black, GI. Rice, and YJ. Crow. Mutations in ctc1, encoding conserved telomere maintenance component 1, cause coats plus. Nat Genet, 44(3):338–342, 2012. PMID: 22267198.

[361] A. Polvi, T. Linnankivi, T. Kivelä, R. Herva, JP. Keating, O. Mäkitie, D. Pareyson, L. Vainionpää, J. Lahtinen, I. Hovatta, H. Pihko, and AE. Lehesjoki. Mutations in ctc1, encoding the cts telomere maintenance complex component 1, cause cerebroretinal microangiopathy with calcifications and cysts. Am J Hum Genet, 90(3): 540–549, 2012. PMCID: PMC3309194, PMID: 22387016.

[362] MW. Ruijs, S. Verhoef, MA. Rookus, R. Pruntel, AH. van der Hout, FB. Hogervorst, I. Kluijt, RH. Sijmons, CM. Aalfs, A. Wagner, MG. Ausems, N. Hoogerbrugge, CJ. van Asperen, EB. Gomez Garcia, H. Meijers-Heijboer, LP. Ten Kate, FH. Menko, and LJ. van 't Veer. Tp53 germline mutation testing in 180 families suspected of li-fraumeni syndrome: mutation detection rate and relative frequency of cancers in different familial phenotypes. J Med Genet, 47(6):421–428, 2010. PMID: 20522432.

[363] A. Shlien, B. Baskin, MI. Achatz, DJ. Stavropoulos, KE. Nichols, L. Hudgins, CF. Morel, MP. Adam, N. Zhukova, L. Rotin, A. Novokmet, H. Druker, M. Shago, PN. Ray, P. Hainaut, and D. Malkin. A common molecular mechanism underlies two phenotypically distinct 17p13.1 microdeletion syndromes. Am J Hum Genet, 87(5): 631–642, 2010. PMCID: PMC2978979, PMID: 21056402.

[364] Y. Zerdoumi, J. Aury-Landas, C. Bonaïti-Pellié, C. Derambure, R. Sesboüé, M. Renaux-Petel, T. Frebourg, G. Bougeard, and JM. Flaman. Drastic effect of germline tp53 missense mutations in li-fraumeni patients. Hum Mutat, 34(3):453–461, 2013. PMID: 23172776.

[365] LR. Susswein, ML. Marshall, R. Nusbaum, KJ. Vogel Postula, SM. Weissman, L. Yackowski, EM. Vaccari, J. Bissonnette, JK. Booker, ML. Cremona, F. Gibellini, PD. Murphy, DE. Pineda-Alvarez, GD. Pollevick, Z. Xu, G. Richard, S. Bale, RT. Klein, KS. Hruska, and WK. Chung. Pathogenic and likely pathogenic variant prevalence among the first 10,000 patients referred for next-generation cancer panel testing. Genet Med, 18(8): 823–832, 2016. PMCID: PMC4985612, PMID: 26681312.

[366] M. Weigell-Weber, S. Fokstuen, B. Török, G. Niemeyer, A. Schinzel, and M. Hergersberg. Codons 837 and 838 in the retinal guanylate cyclase gene on chromosome 17p: hot spots for mutations in autosomal dominant cone-rod dystrophy? Arch Ophthalmol, 118(2):300, 2000. PMID: 10676808.

[367] SE. Wilkie, RJ. Newbold, E. Deery, CE. Walker, I. Stinton, V. Ramamurthy, JB. Hurley, SS. Bhattacharya, MJ. Warren, and DM. Hunt. Functional characterization of missense mutations at codon 838 in retinal guanylate cyclase correlates with disease severity in patients with autosomal dominant cone-rod dystrophy. Hum Mol Genet, 9(20):3065–3073, 2000. PMID: 11115851.

[368] AM. Payne, AG. Morris, SM. Downes, S. Johnson, AC. Bird, AT. Moore, SS. Bhattacharya, and DM. Hunt. Clustering and frequency of mutations in the retinal guanylate cyclase (gucy2d) gene in patients with dominant cone-rod dystrophies. J Med Genet, 38(9):611–614, 2001. PMCID: PMC1734946, PMID: 11565546.

[369] N. Udar, S. Yelchits, M. Chalukya, V. Yellore, S. Nusinowitz, R. Silva-Garcia, T. Vrabec, I. Hussles Maumenee, L. Donoso, and KW. Small. Identification of gucy2d gene mutations in cord5 families and evidence of incomplete penetrance. Hum Mutat, 21(2):170–171, 2003. PMID: 12552567.

[370] D. Zobor, E. Zrenner, B. Wissinger, S. Kohl, and H. Jägle. Gucy2d- or guca1a-related autosomal dominant cone-rod dystrophy: is there a phenotypic difference? Retina, 34(8):1576–1587, 2014. PMID: 24875811.

[371] F. Jiang, K. Xu, X. Zhang, Y. Xie, F. Bai, and Y. Li. Gucy2d mutations in a chinese cohort with autosomal dominant cone or cone-rod dystrophies. Doc Ophthalmol, 131(2):105–114, 2015. PMID: 26298565.

[372] C. Jespersgaard, M. Fang, M. Bertelsen, X. Dang, H. Jensen, Y. Chen, N. Bech, L. Dai, T. Rosenberg, J. Zhang, LB. Møller, Z. Tümer, K. Brøndum-Nielsen, and K. Grønskov. Molecular genetic analysis using targeted ngs analysis of 677 individuals with retinal dystrophy. Sci Rep, 9(1):1219, 2019. PMCID: PMC6362094, PMID: 30718709.

[373] J. Maggi, S. Koller, L. Bähr, S. Feil, F. Kivrak Pfiffner, JVM. Hanson, A. Maspoli, C. Gerth-Kahlert, and W. Berger. Long-range pcr-based ngs applications to diagnose mendelian retinal diseases. Int J Mol Sci, 22(4), 2021. PMCID: PMC7913364, PMID: 33546218.

[374] G. Dodt, DG. Kim, SA. Reimann, BE. Reuber, K. McCabe, SJ. Gould, and SJ. Mihalik. L-pipecolic acid oxidase, a human enzyme essential for the degradation of l-pipecolic acid, is most similar to the monomeric sarcosine oxidases. Biochem J, (Pt 3):487–494, 2000. PMCID: PMC1220782, PMID: 10642506.

[375] JE. Posey, JA. Rosenfeld, RA. James, M. Bainbridge, Z. Niu, X. Wang, S. Dhar, W. Wiszniewski, ZH. Akdemir, T. Gambin, F. Xia, RE. Person, M. Walkiewicz, CA. Shaw, VR. Sutton, AL. Beaudet, D. Muzny, CM. Eng, Y. Yang, RA. Gibbs, JR. Lupski, E. Boerwinkle, and SE. Plon. Molecular diagnostic experience of whole-exome sequencing in adult patients. Genet Med, 18(7):678–685, 2016. PMCID: PMC4892996, PMID: 26633545.

[376] XD. Hao, P. Chen, YY. Zhang, SX. Li, WY. Shi, and H. Gao. De novo mutations of tuba3d are associated with keratoconus. Sci Rep, 7(1):13570, 2017. PMCID: PMC5648796, PMID: 29051577.

[377] CL. Galligan, E. Baig, V. Bykerk, EC. Keystone, and EN. Fish. Distinctive gene expression signatures in rheumatoid arthritis synovial tissue fibroblast cells: correlates with disease activity. Genes Immun, 8(6): 480–491, 2007. PMID: 17568789.

[378] W. Zhang, K. Murao, X. Zhang, K. Matsumoto, S. Diah, M. Okada, K. Miyake, N. Kawai, Z. Fei, and T. Tamiya. Resveratrol represses ykl-40 expression in human glioma u87 cells. BMC Cancer, 10:593, 2010. PMCID: PMC2988030, PMID: 21029458.

[379] BM. Ku, YK. Lee, J. Ryu, JY. Jeong, J. Choi, KM. Eun, HY. Shin, DG. Kim, EM. Hwang, JC. Yoo, JY. Park, GS. Roh, HJ. Kim, GJ. Cho, WS. Choi, SH. Paek, and SS. Kang. Chi3l1 (ykl-40) is expressed in human gliomas and regulates the invasion, growth and survival of glioma cells. Int J Cancer, 128(6):1316–1326, 2011. PMID: 20506295.

[380] C. Ruiz-Romero, V. Calamia, J. Mateos, V. Carreira, M. Martínez-Gomariz, M. Fernández, and FJ. Blanco. Mitochondrial dysregulation of osteoarthritic human articular chondrocytes analyzed by proteomics: a decrease in mitochondrial superoxide dismutase points to a redox imbalance. Mol Cell Proteomics, 8(1):172–189, 2009. PMCID: PMC2713027, PMID: 18784066.

[381] RR. Reams, D. Agrawal, MB. Davis, S. Yoder, FT. Odedina, N. Kumar, JM. Higginbotham, T. Akinremi, S. Suther, and KF. Soliman. Microarray comparison of prostate tumor gene expression in african-american and caucasian american males: a pilot project study.

Infect Agent Cancer, (Suppl 1):S3, 2009. PMCID: PMC2638462, PMID: 19208208.

[382] H. Tummala, AD. Dokal, A. Walne, A. Ellison, S. Cardoso, S. Amirthasigamanipillai, M. Kirwan, I. Browne, JK. Sidhu, V. Rajeeve, A. Rio-Machin, AA. Seraihi, AS. Duncombe, M. Jenner, OP. Smith, H. Enright, A. Norton, T. Aksu, NY. Özbek, N. Pontikos, P. Cutillas, I. Dokal, and T. Vulliamy. Genome instability is a consequence of transcription deficiency in patients with bone marrow failure harboring biallelic ercc6l2 variants. Proc Natl Acad Sci U S A, 115(30):7777–7782, 2018. PMCID: PMC6064997, PMID: 29987015.

[383] E. Dardiotis, E. Karampinis, V. Siokas, AM. Aloizou, D. Rikos, S. Ralli, D. Papadimitriou, DP. Bogdanos, and GM. Hadjigeorgiou. Ercc6l2 rs591486 polymorphism and risk for amyotrophic lateral sclerosis in greek population. Neurol Sci, 40(6):1237–1244, 2019. PMID: 30879219.

[384] CC. Sun, Q. Zhou, W. Hu, SJ. Li, F. Zhang, ZL. Chen, G. Li, ZY. Bi, YY. Bi, FY. Gong, T. Bo, ZP. Yuan, WD. Hu, BT. Zhan, Q. Zhang, QZ. Tang, and DJ. Li. Transcriptional e2f1/2/5/8 as potential targets and transcriptional e2f3/6/7 as new biomarkers for the prognosis of human lung carcinoma. Aging (Albany NY), 10(5):973–987, 2018. PMCID: PMC5990399, PMID: 29754146.

[385] W. Cai, DS. Casey, and JC. Dekkers. Selection response and genetic parameters for residual feed intake in yorkshire swine. J Anim Sci, 86(2):287–298, 2008. PMID: 17998435.

[386] AG. Ozuna, RR. Rowland, JC. Nietfeld, MA. Kerrigan, JC. Dekkers, and CR. Wyatt. Preliminary findings of a previously unrecognized porcine primary immunodeficiency disorder. Vet Pathol, 50(1):144–146, 2013. PMID: 22903400.

[387] MT. Basel, S. Balivada, AP. Beck, MA. Kerrigan, MM. Pyle, JC. Dekkers, CR. Wyatt, RR. Rowland, DE. Anderson, SH. Bossmann, and DL. Troyer. Human xenografts are not rejected in a naturally occurring immunodeficient porcine line: a human tumor model in pigs. Biores Open Access, 1(2):63–68, 2012. PMCID: PMC3559234, PMID: 23514746.

[388] CL. Ewen, AG. Cino-Ozuna, H. He, MA. Kerrigan, JC. Dekkers, CK. Tuggle, RR. Rowland, and CR. Wyatt. Analysis of blood leukocytes in a naturally occurring immunodeficiency of pigs shows the defect is localized to b and t cells. Vet Immunol Immunopathol, 162(3-4):174–179, 2014. PMID: 25454085.

[389] EH. Waide, JC. Dekkers, JW. Ross, RR. Rowland, CR. Wyatt, CL. Ewen, AB. Evans, DM. Thekkoot, NJ. Boddicker, NV. Serão, NM. Ellinwood, and CK. Tuggle. Not all scid pigs are created equally: Two independent mutations in the artemis gene cause scid in pigs. J Immunol, 195(7):3171–3179, 2015. PMCID: PMC5621739, PMID: 26320255.

[390] F. Connell, K. Kalidas, P. Ostergaard, G. Brice, T. Homfray, L. Roberts, DJ. Bunyan, S. Mitton, S. Mansour, P. Mortimer, and S. Jeffery. Linkage and sequence analysis indicate that ccbe1 is mutated in recessively inherited generalised lymphatic dysplasia. Hum Genet, 127(2):231–241, 2010. PMID: 19911200.

[391] M. Alders, BM. Hogan, E. Gjini, F. Salehi, L. Al-Gazali, EA. Hennekam, EE. Holmberg, MM. Mannens, MF. Mulder, GJ. Offerhaus, TE. Prescott, EJ. Schroor, JB. Verheij, M.

Witte, PJ. Zwijnenburg, M. Vikkula, S. Schulte-Merker, and RC. Hennekam. Mutations in ccbe1 cause generalized lymph vessel dysplasia in humans. Nat Genet, 41(12):1272–1274, 2009. PMID: 19935664.

[392] S. Yadav, S. Mukhopadhyay, M. Anbalagan, and N. Makridakis. Somatic mutations in catalytic core of polk reported in prostate cancer alter translesion dna synthesis. Hum Mutat, 36(9):873–880, 2015. PMCID: PMC4537374, PMID: 26046662.

[393] AC. Bostian, L. Maddukuri, MR. Reed, T. Savenka, JH. Hartman, L. Davis, DL. Pouncey, GP. Miller, and RL. Eoff. Kynurenine signaling increases dna polymerase kappa expression and promotes genomic instability in glioblastoma cells. Chem Res Toxicol, 29(1):101–108, 2016. PMCID: PMC4718841, PMID: 26651356.

[394] M. Cai, S. Dai, W. Chen, C. Xia, L. Lu, S. Dai, J. Qi, M. Wang, M. Wang, L. Zhou, F. Lei, T. Zuo, H. Zeng, and X. Zhao. Environmental factors, seven gwas-identified susceptibility loci, and risk of gastric cancer and its precursors in a chinese population. Cancer Med, 6(3):708–720, 2017. PMCID: PMC5345626, PMID: 28220687.

[395] Y. Kinoshita, K. Takasu, T. Yuri, K. Yoshizawa, N. Uehara, A. Kimura, H. Miki, A. Tsubura, and N. Shikata. Two cases of malignant peritoneal mesothelioma without asbestos exposure: cytologic and immunohistochemical features. Ann Diagn Pathol, 17(1):99–9103, 2013. PMID: 22784439.

[396] H. Helgason, T. Rafnar, HS. Olafsdottir, JG. Jonasson, A. Sigurdsson, SN. Stacey, A. Jonasdottir, L. Tryggvadottir, K. Alexiusdottir, A. Haraldsson, L. le Roux, J. Gudmundsson, H. Johannsdottir, A. Oddsson, A. Gylfason, OT. Magnusson, G. Masson, T. Jonsson, H. Skuladottir, DF. Gudbjartsson, U. Thorsteinsdottir, P. Sulem, and K. Stefansson. Loss-of-function variants in atm confer risk of gastric cancer. Nat Genet, 47(8): 906–910, 2015. PMID: 26098866.

[397] A. Kirby, A. Gnirke, DB. Jaffe, V. Barešová, N. Pochet, B. Blumenstiel, C. Ye, D. Aird, C. Stevens, JT. Robinson, MN. Cabili, I. Gat-Viks, E. Kelliher, R. Daza, M. DeFelice, H. Hůlková, J. Sovová, P. Vylet'al, C. Antignac, M. Guttman, RE. Handsaker, D. Perrin, S. Steelman, S. Sigurdsson, SJ. Scheinman, C. Sougnez, K. Cibulskis, M. Parkin, T. Green, E. Rossin, MC. Zody, RJ. Xavier, MR. Pollak, SL. Alper, K. Lindblad-Toh, S. Gabriel, PS. Hart, A. Regev, C. Nusbaum, S. Kmoch, AJ. Bleyer, ES. Lander, and MJ. Daly. Mutations causing medullary cystic kidney disease type 1 lie in a large vntr in muc1 missed by massively parallel sequencing. Nat Genet, 45 (3):299–303, 2013. PMCID: PMC3901305, PMID: 23396133.

[398] J. Creaney, A. Segal, G. Sterrett, MA. Platten, E. Baker, AR. Murch, AK. Nowak, BW. Robinson, and MJ. Millward. Overexpression and altered glycosylation of muc1 in malignant mesothelioma. Br J Cancer, 98(9): 1562–1569, 2008. PMCID: PMC2391110, PMID: 18454162.

[399] X. Xu, L. Bai, W. Chen, MT. Padilla, Y. Liu, KC. Kim, SA. Belinsky, and Y. Lin. Muc1 contributes to bpde-induced human bronchial epithelial cell transformation through facilitating egfr activation. PLoS One, 7(3):e33846, 2012. PMCID: PMC3310874, PMID: 22457794.

[400] SP. Treon, JA. Mollick, M. Urashima, G. Teoh, D. Chauhan, A. Ogata, N. Raje, JH. Hilgers, L. Nadler, AR. Belch, LM. Pilarski, and KC. Anderson. Muc-1 core protein is expressed on multiple myeloma cells and is induced by dexamethasone. Blood, 93(4):1287–1298, 1999. PMID: 9949172.

[401] CH. Takimoto, ZH. Lu, R. Zhang, MD. Liang, LV. Larson, LR. Cantilena, JL. Grem, CJ. Allegra, RB. Diasio, and E. Chu. Severe neurotoxicity following 5-fluorouracil-based chemotherapy in a patient with dihydropyrimidine dehydrogenase deficiency. Clin Cancer Res, 2(3):477–481, 1996. PMID: 9816193.

[402] CW. Woods, MT. McClain, M. Chen, AK. Zaas, BP. Nicholson, J. Varkey, T. Veldman, SF. Kingsmore, Y. Huang, R. Lambkin-Williams, AG. Gilbert, AO. Hero, E. Ramsburg, S. Glickman, JE. Lucas, L. Carin, and GS. Ginsburg. A host transcriptional signature for presymptomatic detection of infection in humans exposed to influenza h1n1 or h3n2. PLoS One, 8(1):e52198, 2013. PMCID: PMC3541408, PMID: 23326326.

[403] KA. Aldinger, AE. Timms, Z. Thomson, GM. Mirzaa, JT. Bennett, AB. Rosenberg, CM. Roco, M. Hirano, F. Abidi, P. Haldipur, CV. Cheng, S. Collins, K. Park, J. Zeiger, LM. Overmann, FS. Alkuraya, LG. Biesecker, SR. Braddock, S. Cathey, MT. Cho, BHY. Chung, DB. Everman, YA. Zarate, JR. Jones, CE. Schwartz, A. Goldstein, RJ. Hopkin, ID. Krantz, RL. Ladda, KA. Leppig, BC. McGillivray, S. Sell, K. Wusik, JG. Gleeson, DA. Nickerson, MJ. Bamshad, D. Gerrelli, SN. Lisgo, G. Seelig, GE. Ishak, AJ. Barkovich, CJ. Curry, IA. Glass, KJ. Millen, D. Doherty, and WB. Dobyns. Redefining the etiologic landscape of cerebellar malformations. Am J Hum Genet, 105(3):606–615, 2019. PMCID: PMC6731369, PMID: 31474318.

[404] C. Nava, F. Lamari, D. Héron, C. Mignot, A. Rastetter, B. Keren, D. Cohen, A. Faudet, D. Bouteiller, M. Gilleron, A. Jacquette, S. Whalen, A. Afenjar, D. Périsse, C. Laurent, C. Dupuits, C. Gautier, M. Gérard, G. Huguet, S. Caillet, B. Leheup, M. Leboyer, C. Gillberg, R. Delorme, T. Bourgeron, A. Brice, and C. Depienne. Analysis of the chromosome x exome in patients with autism spectrum disorders identified novel candidate genes, including tmlhe. Transl Psychiatry, 2(10):e179, 2012. PMCID: PMC3565810, PMID: 23092983.

[405] YJ. Yang, L. Hu, YP. Xia, CY. Jiang, C. Miao, CQ. Yang, M. Yuan, and L. Wang. Resveratrol suppresses glial activation and alleviates trigeminal neuralgia via activation of ampk. J Neuroinflammation, 13(1):84, 2016. PMCID: PMC4837542, PMID: 27093858.

[406] M. Tong, M. Dong, and SM. de la Monte. Brain insulin-like growth factor and neurotrophin resistance in parkinson's disease and dementia with lewy bodies: potential role of manganese neurotoxicity. J Alzheimers Dis, 16(3):585–599, 2009. PMCID: PMC2852260, PMID: 19276553.

[407] T. Chen, W. Wang, JR. Li, HZ. Xu, YC. Peng, LF. Fan, F. Yan, C. Gu, L. Wang, and G. Chen. Parp inhibition attenuates early brain injury through nf-κb/mmp-9 pathway in a rat model of subarachnoid hemorrhage. Brain Res, 1644:32–38, 2016. PMID: 27157545.

[408] SS. Wang, Y. Lu, N. Rothman, AM. Abdou, JR. Cerhan, A. De Roos, S. Davis, RK. Severson, W. Cozen, SJ. Chanock, L. Bernstein, LM. Morton, and P. Hartge. Variation

in effects of non-hodgkin lymphoma risk factors according to the human leukocyte antigen (hla)-drb1*01:01 allele and ancestral haplotype 8.1. PLoS One, 6 (11):e26949, 2011. PMCID: PMC3212525, PMID: 22096508.

[409] SH. Kim, YM. Ye, NS. Palikhe, JE. Kim, and HS. Park. Genetic and ethnic risk factors associated with drug hypersensitivity. Curr Opin Allergy Clin Immunol, 10(4):280–290, 2010. PMID: 20485159.

[410] M. Marchini, R. Antonioli, A. Lleò, M. Barili, M. Caronni, L. Origgi, M. Vanoli, and R. Scorza. Hla class ii antigens associated with lupus nephritis in italian sle patients. Hum Immunol, 64(4):462–468, 2003. PMID: 12651073.

[411] W. Wen, W. Su, H. Tang, W. Le, X. Zhang, Y. Zheng, X. Liu, L. Xie, J. Li, J. Ye, L. Dong, X. Cui, Y. Miao, D. Wang, J. Dong, C. Xiao, W. Chen, and H. Wang. Immune cell profiling of covid-19 patients in the recovery stage by single-cell sequencing. Cell Discov, 6:31, 2020. PMCID: PMC7197635, PMID: 32377375.

[412] JR. Bill, DG. Mack, MT. Falta, LA. Maier, AK. Sullivan, FG. Joslin, AK. Martin, BM. Freed, BL. Kotzin, and AP. Fontenot. Beryllium presentation to cd4+ t cells is dependent on a single amino acid residue of the mhc class ii beta-chain. J Immunol, 175(10):7029–7037, 2005. PMID: 16272364.

[413] H. Sato, P. Spagnolo, L. Silveira, KI. Welsh, RM. du Bois, LS. Newman, and LA. Maier. Btnl2 allele associations with chronic beryllium disease in hla-dpb1*glu69-negative individuals. Tissue Antigens, 70(6):480–486, 2007. PMID: 17927685.

[414] I. Gunnarsson, B. Ringertz, J. Bratt, and B. Sundelin. Hla-drb1*0301 and dqa1*0501 in ra. Ann Rheum Dis, 60 (7):727, 2001. PMCID: PMC1753742, PMID: 11436868.

[415] N. Zhu, F. Luo, Q. Chen, N. Li, H. Xiong, Y. Feng, Z. Yang, and W. Hou. Influence of hla-drb alleles on haemorrhagic fever with renal syndrome in a chinese han population in hubei province, china. Eur J Clin Microbiol Infect Dis, 34(1):187–195, 2015. PMID: 25169964.

[416] MD. Rossman, B. Thompson, M. Frederick, M. Maliarik, MC. Iannuzzi, BA. Rybicki, JP. Pandey, LS. Newman, E. Magira, B. Beznik-Cizman, and D. Monos. Hla-drb1*1101: a significant risk factor for sarcoidosis in blacks and whites. Am J Hum Genet, 73(4):720–735, 2003. PMCID: PMC1180597, PMID: 14508706.

[417] F. Speerstra, P. Reekers, LB. van de Putte, JP. Vandenbroucke, JJ. Rasker, and DJ. de Rooij. Hla-dr antigens and proteinuria induced by aurothioglucose and d-penicillamine in patients with rheumatoid arthritis. J Rheumatol, 10(6):948–953, 1983. PMID: 6420562.

[418] AA. Al-Eisa, MZ. Haider, and BS. Srivasta. Hla-drb1 alleles in kuwaiti children with idiopathic nephrotic syndrome. Pediatr Nephrol, 15(1-2):79–81, 2000. PMID: 11095018.

[419] K. Saito. [analysis of a genetic factor of metal allergy–polymorphism of hla-dr, -dq gene]. Kokubyo Gakkai Zasshi, 63(1):53–69, 1996. PMID: 8725357.

[420] RP. Warren, JD. Odell, WL. Warren, RA. Burger, A. Maciulis, WW. Daniels, and AR. Torres. Strong association of the third hypervariable region of hla-dr beta 1 with autism.

J Neuroimmunol, 67(2):97–9102, 1996. PMID: 8765331.

[421] SJ. Dunstan, NT. Hue, B. Han, Z. Li, TT. Tram, KS. Sim, CM. Parry, NT. Chinh, H. Vinh, NP. Lan, NT. Thieu, PV. Vinh, S. Koirala, S. Dongol, A. Arjyal, A. Karkey, O. Shilpakar, C. Dolecek, JN. Foo, le T. Phuong, MN. Lanh, T. Do, T. Aung, DN. Hon, YY. Teo, ML. Hibberd, KL. Anders, Y. Okada, S. Raychaudhuri, CP. Simmons, S. Baker, PI. de Bakker, B. Basnyat, TT. Hien, JJ. Farrar, and CC. Khor. Variation at hla-drb1 is associated with resistance to enteric fever. Nat Genet, 46(12):1333–1336, 2014. PMCID: PMC5099079, PMID: 25383971.

[422] M. Grosser, T. Luther, M. Fuessel, J. Bickhardt, V. Magdolen, and G. Baretton. Clinical course of sarcoidosis in dependence on hla-drb1 allele frequencies, inflammatory markers, and the presence of m. tuberculosis dna fragments. Sarcoidosis Vasc Diffuse Lung Dis, 22(1):66–74, 2005. PMID: 15881283.

[423] SH. Kim, JH. Choi, KW. Lee, SH. Kim, ES. Shin, HB. Oh, CH. Suh, DH. Nahm, and HS. Park. The human leucocyte antigen-drb1*1302-dqb1*0609-dpb1*0201 haplotype may be a strong genetic marker for aspirin-induced urticaria. Clin Exp Allergy, 35(3):339–344, 2005. PMID: 15784113.

[424] LOF. Cangussu, R. Teixeira, EF. Campos, GF. Rampim, SA. Mingoti, OA. Martins-Filho, and M. Gerbase-DeLima. Hla class ii alleles and chronic hepatitis c virus infection. Scand J Immunol, 74(3): 282–287, 2011. PMID: 21535077.

[425] N. Kaushansky, M. Eisenstein, S. Boura-Halfon, BE. Hansen, CH. Nielsen, R. Milo, G. Zeilig, H. Lassmann, DM. Altmann, and A. Ben-Nun. Role of a novel human leukocyte antigen-dqa1*01:02;drb1*15:01 mixed isotype heterodimer in the pathogenesis of "humanized" multiple sclerosis-like disease. J Biol Chem, 290(24): 15260–15278, 2015. PMCID: PMC4463466, PMID: 25911099.

[426] SH. Kim, HB. Oh, KW. Lee, ES. Shin, CW. Kim, CS. Hong, DH. Nahm, and HS. Park. Hla drb1*15-dpb1*05 haplotype: a susceptible gene marker for isocyanate-induced occupational asthma? Allergy, 61(7):891–894, 2006. PMID: 16792590.

[427] N. Keicho, S. Itoyama, K. Kashiwase, NC. Phi, HT. Long, LD. Ha, VV. Ban, BK. Hoa, NT. Hang, M. Hijikata, S. Sakurada, M. Satake, K. Tokunaga, T. Sasazuki, and T. Quy. Association of human leukocyte antigen class ii alleles with severe acute respiratory syndrome in the vietnamese population. Hum Immunol, 70(7):527–531, 2009. PMCID: PMC7132661, PMID: 19445991.

[428] GA. Heap, MN. Weedon, CM. Bewshea, A. Singh, M. Chen, JB. Satchwell, JP. Vivian, K. So, PC. Dubois, JM. Andrews, V. Annese, P. Bampton, M. Barnardo, S. Bell, A. Cole, SJ. Connor, T. Creed, FR. Cummings, M. D’Amato, TK. Daneshmend, RN. Fedorak, TH. Florin, DR. Gaya, E. Greig, J. Halfvarson, A. Hart, PM. Irving, G. Jones, A. Karban, IC. Lawrance, JC. Lee, C. Lees, R. Lev-Tzion, JO. Lindsay, J. Mansfield, J. Mawdsley, Z. Mazhar, M. Parkes, K. Parnell, TR. Orchard, G. Radford-Smith, RK. Russell, D. Reffitt, J. Satsangi, MS. Silverberg, GC. Sturniolo, M. Tremelling, EV. Tsianos, DA. van Heel, A. Walsh, G. Watermeyer, RK. Weersma, S. Zeissig, J. Rossjohn, AL. Holden, and T. Ahmad. Hla-dqa1-hla-drb1 variants confer susceptibility to pancreatitis induced by thiopurine immunosuppressants. Nat Genet, 46(10):1131–1134, 2014. PMCID:

PMC6379053, PMID: 25217962.

[429] SJ. Glatt, IP. Everall, WS. Kremen, J. Corbeil, R. Sásik, N. Khanlou, M. Han, CC. Liew, and MT. Tsuang. Comparative gene expression analysis of blood and brain provides concurrent validation of selenbp1 up-regulation in schizophrenia. Proc Natl Acad Sci U S A, 102(43):15533–15538, 2005. PMCID: PMC1266138, PMID: 16223876.

[430] H. Mahdi, BA. Fisher, H. Källberg, D. Plant, V. Malmström, J. Rönnelid, P. Charles, B. Ding, L. Alfredsson, L. Padyukov, DP. Symmons, PJ. Venables, L. Klareskog, and K. Lundberg. Specific interaction between genotype, smoking and autoimmunity to citrullinated alpha-enolase in the etiology of rheumatoid arthritis. Nat Genet, 41(12):1319–1324, 2009. PMID: 19898480.

[431] EA. Stahl, S. Raychaudhuri, EF. Remmers, G. Xie, S. Eyre, BP. Thomson, Y. Li, FA. Kurreeman, A. Zhernakova, A. Hinks, C. Guiducci, R. Chen, L. Alfredsson, CI. Amos, KG. Ardlie, A. Barton, J. Bowes, E. Brouwer, NP. Burtt, JJ. Catanese, J. Coblyn, MJ. Coenen, KH. Costenbader, LA. Criswell, JB. Crusius, J. Cui, PI. de Bakker, PL. De Jager, B. Ding, P. Emery, E. Flynn, P. Harrison, LJ. Hocking, TW. Huizinga, DL. Kastner, X. Ke, AT. Lee, X. Liu, P. Martin, AW. Morgan, L. Padyukov, MD. Posthumus, TR. Radstake, DM. Reid, M. Seielstad, MF. Seldin, NA. Shadick, S. Steer, PP. Tak, W. Thomson, AH. van der Helm-van Mil, IE. van der Horst-Bruinsma, CE. van der Schoot, PL. van Riel, ME. Weinblatt, AG. Wilson, GJ. Wolbink, BP. Wordsworth, C. Wijmenga, EW. Karlson, RE. Toes, N. de Vries, AB. Begovich, J. Worthington, KA. Siminovitch, PK. Gregersen, L. Klareskog, and RM. Plenge. Genome-wide association study meta-analysis identifies seven new rheumatoid arthritis risk loci. Nat Genet, 42(6):508–514, 2010. PMCID: PMC4243840, PMID: 20453842.

[432] S. Raychaudhuri, C. Sandor, EA. Stahl, J. Freudenberg, HS. Lee, X. Jia, L. Alfredsson, L. Padyukov, L. Klareskog, J. Worthington, KA. Siminovitch, SC. Bae, RM. Plenge, PK. Gregersen, and PI. de Bakker. Five amino acids in three hla proteins explain most of the association between mhc and seropositive rheumatoid arthritis. Nat Genet, 44(3):291–296, 2012. PMCID: PMC3288335, PMID: 22286218.

[433] SV. Ramagopalan, UC. Meier, M. Conacher, GC. Ebers, G. Giovannoni, DH. Crawford, and KA. McAulay. Role of the hla system in the association between multiple sclerosis and infectious mononucleosis. Arch Neurol, 68 (4):469–472, 2011. PMID: 21482926.

[434] P. Goyette, G. Boucher, D. Mallon, E. Ellinghaus, L. Jostins, H. Huang, S. Ripke, ES. Gusareva, V. Annese, SL. Hauser, JR. Oksenberg, I. Thomsen, S. Leslie, MJ. Daly, K. Van Steen, RH. Duerr, JC. Barrett, DP. McGovern, LP. Schumm, JA. Traherne, MN. Carrington, V. Kosmoliaptsis, TH. Karlsen, A. Franke, and JD. Rioux. High-density mapping of the mhc identifies a shared role for hla-drb1*01:03 in inflammatory bowel diseases and heterozygous advantage in ulcerative colitis. Nat Genet, 47(2):172–179, 2015. PMCID: PMC4310771, PMID: 25559196.

[435] H. Hor, Z. Kutalik, Y. Dauvilliers, A. Valsesia, GJ. Lammers, CE. Donjacour, A. Iranzo, J. Santamaria, R. Peraita Adrados, JL. Vicario, S. Overeem, I. Arnulf, I. Theodorou, P. Jennum, S. Knudsen, C. Bassetti, J. Mathis, M. Lecendreux, G. Mayer, P. Geisler, A. Benetó, B. Petit, C. Pfister, JV. Bürki, G. Didelot, M. Billiard, G. Ercilla, W. Verduijn, FH. Claas, P. Vollenweider, G. Waeber, DM. Waterworth, V. Mooser, R. Heinzer, JS.

Beckmann, S. Bergmann, and M. Tafti. Genome-wide association study identifies new hla class ii haplotypes strongly protective against narcolepsy. Nat Genet, 42(9):786–789, 2010. PMID: 20711174.

[436] C. Lesseur, B. Diergaarde, AF. Olshan, V. Wünsch-Filho, AR. Ness, G. Liu, M. Lacko, J. Eluf-Neto, S. Franceschi, P. Lagiou, GJ. Macfarlane, L. Richiardi, S. Boccia, J. Polesel, K. Kjaerheim, D. Zaridze, M. Johansson, AM. Menezes, MP. Curado, M. Robinson, W. Ahrens, C. Canova, A. Znaor, X. Castellsagué, DI. Conway, I. Holcátová, D. Mates, M. Vilensky, CM. Healy, N. Szeszenia-Dabrowska, ˛ E. Fabiánová, J. Lissowska, JR. Grandis, MC. Weissler, EH. Tajara, FD. Nunes, MB. de Carvalho, S. Thomas, RJ. Hung, WH. Peters, R. Herrero, G. Cadoni, HB. Bueno-de Mesquita, A. Steffen, A. Agudo, O. Shangina, X. Xiao, V. Gaborieau, A. Chabrier, D. Anantharaman, P. Boffetta, CI. Amos, JD. McKay, and P. Brennan. Genome-wide association analyses identify new susceptibility loci for oral cavity and pharyngeal cancer. Nat Genet, 48(12):1544–1550, 2016. PMCID: PMC5131845, PMID: 27749845.

[437] MI. Lucena, M. Molokhia, Y. Shen, TJ. Urban, GP. Aithal, RJ. Andrade, CP. Day, F. Ruiz-Cabello, PT. Donaldson, C. Stephens, M. Pirmohamed, M. Romero-Gomez, JM. Navarro, RJ. Fontana, M. Miller, M. Groome, E. Bondon-Guitton, A. Conforti, BH. Stricker, A. Carvajal, L. Ibanez, QY. Yue, M. Eichelbaum, A. Floratos, I. Pe'er, MJ. Daly, DB. Goldstein, JF. Dillon, MR. Nelson, PB. Watkins, and AK. Daly. Susceptibility to amoxicillin-clavulanate-induced liver injury is influenced by multiple hla class i and ii alleles. Gastroenterology, 141(1):338–347, 2011. PMCID: PMC3129430, PMID: 21570397.

[438] M. Fakiola, A. Strange, HJ. Cordell, EN. Miller, M. Pirinen, Z. Su, A. Mishra, S. Mehrotra, GR. Monteiro, G. Band, C. Bellenguez, S. Dronov, S. Edkins, C. Freeman, E. Giannoulatou, E. Gray, SE. Hunt, HG. Lacerda, C. Langford, R. Pearson, NN. Pontes, M. Rai, SP. Singh, L. Smith, O. Sousa, D. Vukcevic, E. Bramon, MA. Brown, JP. Casas, A. Corvin, A. Duncanson, J. Jankowski, HS. Markus, CG. Mathew, CN. Palmer, R. Plomin, A. Rautanen, SJ. Sawcer, RC. Trembath, AC. Viswanathan, NW. Wood, ME. Wilson, P. Deloukas, L. Peltonen, F. Christiansen, C. Witt, SM. Jeronimo, S. Sundar, CC. Spencer, JM. Blackwell, and P. Donnelly. Common variants in the hla-drb1-hla-dqa1 hla class ii region are associated with susceptibility to visceral leishmaniasis. Nat Genet, 45(2):208–213, 2013. PMCID: PMC3664012, PMID: 23291585.

[439] X. Hu, AJ. Deutsch, TL. Lenz, S. Onengut-Gumuscu, B. Han, WM. Chen, JM. Howson, JA. Todd, PI. de Bakker, SS. Rich, and S. Raychaudhuri. Additive and interaction effects at three amino acid positions in hla-dq and hla-dr molecules drive type 1 diabetes risk. Nat Genet, 47(8):898–905, 2015. PMCID: PMC4930791, PMID: 26168013.

[440] S. Okada, ML. Kamb, JP. Pandey, RM. Philen, LA. Love, and FW. Miller. Immunogenetic risk and protective factors for the development of l-tryptophan-associated eosinophilia-myalgia syndrome and associated symptoms. Arthritis Rheum, 61(10):1305–1311, 2009. PMCID: PMC2761987, PMID: 19790128.

[441] SH. Kim and HS. Park. Genetic markers for differentiating aspirin-hypersensitivity. Yonsei Med J, 47(1):15–21, 2006. PMCID: PMC2687575, PMID: 16502481.

[442] CE. Mapp, B. Beghè, A. Balboni, G. Zamorani, M. Padoan, L. Jovine, OR. Baricordi, and

LM. Fabbri. Association between hla genes and susceptibility to toluene diisocyanate-induced asthma. Clin Exp Allergy, 30 (5):651–656, 2000. PMID: 10792356.

[443] K. Kiryluk, Y. Li, F. Scolari, S. Sanna-Cherchi, M. Choi, M. Verbitsky, D. Fasel, S. Lata, S. Prakash, S. Shapiro, C. Fischman, HJ. Snyder, G. Appel, C. Izzi, BF. Viola, N. Dallera, L. Del Vecchio, C. Barlassina, E. Salvi, FE. Bertinetto, A. Amoroso, S. Savoldi, M. Rocchietti, A. Amore, L. Peruzzi, R. Coppo, M. Salvadori, P. Ravani, R. Magistroni, GM. Ghiggeri, G. Caridi, M. Bodria, F. Lugani, L. Allegri, M. Delsante, M. Maiorana, A. Magnano, G. Frasca, E. Boer, G. Boscutti, C. Ponticelli, R. Mignani, C. Marcantoni, D. Di Landro, D. Santoro, A. Pani, R. Polci, S. Feriozzi, S. Chicca, M. Galliani, M. Gigante, L. Gesualdo, P. Zamboli, GG. Battaglia, M. Garozzo, D. Maixnerová, V. Tesar, F. Eitner, T. Rauen, J. Floege, T. Kovacs, J. Nagy, K. Mucha, L. Paczek, ˛ M. Zaniew, M. Mizerska-Wasiak, M. Roszkowska-Blaim, K. Pawlaczyk, D. Gale, J. Barratt, L. Thibaudin, F. Berthoux, G. Canaud, A. Boland, M. Metzger, U. Panzer, H. Suzuki, S. Goto, I. Narita, Y. Caliskan, J. Xie, P. Hou, N. Chen, H. Zhang, RJ. Wyatt, J. Novak, BA. Julian, J. Feehally, B. Stengel, D. Cusi, RP. Lifton, and AG. Gharavi. Discovery of new risk loci for iga nephropathy implicates genes involved in immunity against intestinal pathogens. Nat Genet, 46(11):1187–1196, 2014. PMCID: PMC4213311, PMID: 25305756.

[444] TR. Radstake, O. Gorlova, B. Rueda, JE. Martin, BZ. Alizadeh, R. Palomino-Morales, MJ. Coenen, MC. Vonk, AE. Voskuyl, AJ. Schuerwegh, JC. Broen, PL. van Riel, R. van 't Slot, A. Italiaander, RA. Ophoff, G. Riemekasten, N. Hunzelmann, CP. Simeon, N. Ortego-Centeno, MA. González-Gay, MF. González-Escribano, P. Airo, J. van Laar, A. Herrick, J. Worthington, R. Hesselstrand, V. Smith, F. de Keyser, F. Houssiau, MM. Chee, R. Madhok, P. Shiels, R. Westhovens, A. Kreuter, H. Kiener, E. de Baere, T. Witte, L. Padykov, L. Klareskog, L. Beretta, R. Scorza, BA. Lie, AM. Hoffmann-Vold, P. Carreira, J. Varga, M. Hinchcliff, PK. Gregersen, AT. Lee, J. Ying, Y. Han, SF. Weng, CI. Amos, FM. Wigley, L. Hummers, JL. Nelson, SK. Agarwal, S. Assassi, P. Gourh, FK. Tan, BP. Koeleman, FC. Arnett, J. Martin, and MD. Mayes. Genome-wide association study of systemic sclerosis identifies cd247 as a new susceptibility locus. Nat Genet, 42(5): 426–429, 2010. PMCID: PMC2861917, PMID: 20383147.

[445] I. Gockel, J. Becker, MM. Wouters, S. Niebisch, HR. Gockel, T. Hess, D. Ramonet, J. Zimmermann, AG. Vigo, G. Trynka, AR. de León, JP. de la Serna, E. Urcelay, V. Kumar, L. Franke, HJ. Westra, D. Drescher, W. Kneist, JU. Marquardt, PR. Galle, M. Mattheisen, V. Annese, A. Latiano, U. Fumagalli, L. Laghi, R. Cuomo, G. Sarnelli, M. Müller, AJ. Eckardt, J. Tack, P. Hoffmann, S. Herms, E. Mangold, S. Heilmann, R. Kiesslich, BH. von Rahden, HD. Allescher, HG. Schulz, C. Wijmenga, MT. Heneka, H. Lang, KP. Hopfner, MM. Nöthen, GE. Boeckxstaens, PI. de Bakker, M. Knapp, and J. Schumacher. Common variants in the hla-dq region confer susceptibility to idiopathic achalasia. Nat Genet, 46(8):901–904, 2014. PMID: 24997987.

[446] M. Hussong, ST. Börno, M. Kerick, A. Wunderlich, A. Franz, H. Sültmann, B. Timmermann, H. Lehrach, M. Hirsch-Kauffmann, and MR. Schweiger. The bromodomain protein brd4 regulates the keap1/nrf2-dependent oxidative stress response. Cell Death Dis, 5(4):e1195, 2014. PMCID: PMC4001311, PMID: 24763052.

[447] F. Marbach, CF. Rustad, A. Riess, D. Đukić, TC. Hsieh, I. Jobani, T. Prescott, A. Bevot, F. Erger, G. Houge, M. Redfors, J. Altmueller, T. Stokowy, C. Gilissen, C. Kubisch, E. Scarano, L. Mazzanti, T. Fiskerstrand, PM. Krawitz, D. Lessel, and C. Netzer. The

discovery of a lemd2-associated nuclear envelopathy with early progeroid appearance suggests advanced applications for ai-driven facial phenotyping. Am J Hum Genet, 104 (4):749–757, 2019. PMCID: PMC6451726, PMID: 30905398.

[448] M. Christensen, M. Duno, AM. Lund, F. Skovby, and E. Christensen. Xanthurenic aciduria due to a mutation in kynu encoding kynureninase. J Inherit Metab Dis, 30(2):248–255, 2007. PMID: 17334708.

[449] DG. Walker, J. Link, LF. Lue, JE. Dalsing-Hernandez, and BE. Boyes. Gene expression changes by amyloid beta peptide-stimulated human postmortem brain microglia identify activation of multiple inflammatory processes. J Leukoc Biol, 79(3):596–610, 2006. PMID: 16365156.

[450] N. Ehmke, K. Cusmano-Ozog, R. Koenig, M. Holtgrewe, B. Nur, E. Mihci, H. Babcock, C. Gonzaga-Jauregui, JD. Overton, J. Xiao, AF. Martinez, M. Muenke, A. Balzer, J. Jochim, N. El Choubassi, B. Fischer-Zirnsak, C. Huber, U. Kornak, SH. Elsea, V. Cormier-Daire, and CR. Ferreira. Biallelic variants in kynu cause a multisystemic syndrome with hand hyperphalangism. Bone, 133:115219, 2020. PMCID: PMC10521254, PMID: 31923704.

[451] RW. Collin, K. Nikopoulos, M. Dona, C. Gilissen, A. Hoischen, FN. Boonstra, JA. Poulter, H. Kondo, W. Berger, C. Toomes, T. Tahira, LR. Mohn, EA. Blokland, L. Hetterschijt, M. Ali, JM. Groothuismink, L. Duijkers, CF. Inglehearn, L. Sollfrank, TM. Strom, E. Uchio, CE. van Nouhuys, H. Kremer, JA. Veltman, E. van Wijk, and FP. Cremers. Znf408 is mutated in familial exudative vitreoretinopathy and is crucial for the development of zebrafish retinal vasculature. Proc Natl Acad Sci U S A, 110(24):9856–9861, 2013. PMCID: PMC3683717, PMID: 23716654.

[452] I. Habibi, A. Chebil, F. Kort, DF. Schorderet, and L. El Matri. Exome sequencing confirms znf408 mutations as a cause of familial retinitis pigmentosa. Ophthalmic Genet, 38(5):494–497, 2017. PMID: 28095122.

[453] A. Avila-Fernandez, R. Perez-Carro, M. Corton, MI. Lopez-Molina, L. Campello, A. Garanto, L. Fernandez-Sanchez, L. Duijkers, MA. Lopez-Martinez, R. Riveiro-Alvarez, LR. Da Silva, R. Sanchez-Alcudia, E. Martin-Garrido, N. Reyes, F. Garcia-Garcia, J. Dopazo, B. Garcia-Sandoval, RW. Collin, N. Cuenca, and C. Ayuso. Whole-exome sequencing reveals znf408 as a new gene associated with autosomal recessive retinitis pigmentosa with vitreal alterations. Hum Mol Genet, 24(14):4037–4048, 2015. PMID: 25882705.

[454] H. Najmabadi, H. Hu, M. Garshasbi, T. Zemojtel, SS. Abedini, W. Chen, M. Hosseini, F. Behjati, S. Haas, P. Jamali, A. Zecha, M. Mohseni, L. Püttmann, LN. Vahid, C. Jensen, LA. Moheb, M. Bienek, F. Larti, I. Mueller, R. Weissmann, H. Darvish, K. Wrogemann, V. Hadavi, B. Lipkowitz, S. Esmaeeli-Nieh, D. Wieczorek, R. Kariminejad, SG. Firouzabadi, M. Cohen, Z. Fattahi, I. Rost, F. Mojahedi, C. Hertzberg, A. Dehghan, A. Rajab, MJ. Banavandi, J. Hoffer, M. Falah, L. Musante, V. Kalscheuer, R. Ullmann, AW. Kuss, A. Tzschach, K. Kahrizi, and HH. Ropers. Deep sequencing reveals 50 novel genes for recessive cognitive disorders. Nature, 478(7367):57–63, 2011. PMID: 21937992.

[455] RS. Thomas, TM. O'Connell, L. Pluta, RD. Wolfinger, L. Yang, and TJ. Page. A

comparison of transcriptomic and metabonomic technologies for identifying biomarkers predictive of two-year rodent cancer bioassays. Toxicol Sci, 96(1):40–46, 2007. PMID: 17114358.

[456] S. Nicole, A. Chaouch, T. Torbergsen, S. Bauché, E. de Bruyckere, MJ. Fontenille, MA. Horn, M. van Ghelue, S. Løseth, Y. Issop, D. Cox, JS. Müller, T. Evangelista, E. Stålberg, C. Ioos, A. Barois, G. Brochier, D. Sternberg, E. Fournier, D. Hantaï, A. Abicht, M. Dusl, SH. Laval, H. Griffin, B. Eymard, and H. Lochmüller. Agrin mutations lead to a congenital myasthenic syndrome with distal muscle weakness and atrophy. Brain, 137(Pt 9): 2429–2443, 2014. PMID: 24951643.

[457] JC. Zenteno, B. Buentello-Volante, MA. Quiroz-González, and MA. Quiroz-Reyes. Compound heterozygosity for a novel and a recurrent mfrp gene mutation in a family with the nanophthalmos-retinitis pigmentosa complex. Mol Vis, 15:1794–1798, 2009. PMCID: PMC2742641, PMID: 19753314.

[458] MB. Consugar, D. Navarro-Gomez, EM. Place, KM. Bujakowska, ME. Sousa, ZD. Fonseca-Kelly, DG. Taub, M. Janessian, DY. Wang, ED. Au, KB. Sims, DA. Sweetser, AB. Fulton, Q. Liu, JL. Wiggs, X. Gai, and EA. Pierce. Panel-based genetic diagnostic testing for inherited eye diseases is highly accurate and reproducible, and more sensitive for variant detection, than exome sequencing. Genet Med, 17(4):253–261, 2015. PMCID: PMC4572572, PMID: 25412400.

[459] Y. Xu, L. Guan, X. Xiao, J. Zhang, S. Li, H. Jiang, X. Jia, Y. Yin, X. Guo, Z. Yang, and Q. Zhang. Identification of mfrp mutations in chinese families with high hyperopia. Optom Vis Sci, 93(1):19–26, 2016. PMID: 26583794.

[460] G. Velez, SH. Tsang, YT. Tsai, CW. Hsu, A. Gore, AH. Abdelhakim, M. Mahajan, RH. Silverman, JR. Sparrow, AG. Bassuk, and VB. Mahajan. Gene therapy restores mfrp and corrects axial eye length. Sci Rep, 7(1):16151, 2017. PMCID: PMC5701072, PMID: 29170418.

[461] D. Matías-Pérez, LA. García-Montaño, M. Cruz-Aguilar, IA. García-Montalvo, J. Nava-Valdéz, T. Barragán-Arevalo, C. Villanueva-Mendoza, CE. Villarroel, C. Guadarrama-Vallejo, RV. la Cruz, O. Chacón-Camacho, and JC. Zenteno. Identification of novel pathogenic variants and novel gene-phenotype correlations in mexican subjects with microphthalmia and/or anophthalmia by next-generation sequencing. J Hum Genet, 63(11):1169–1180, 2018. PMID: 30181649.

[462] R. Ayyagari, MN. Mandal, AJ. Karoukis, L. Chen, NC. McLaren, M. Lichter, DT. Wong, PF. Hitchcock, RC. Caruso, SE. Moroi, IH. Maumenee, and PA. Sieving. Late-onset macular degeneration and long anterior lens zonules result from a ctrp5 gene mutation. Invest Ophthalmol Vis Sci, 46(9):3363–3371, 2005. PMID: 16123441.

[463] OH. Sundin, GS. Leppert, ED. Silva, JM. Yang, S. Dharmaraj, IH. Maumenee, LC. Santos, CF. Parsa, EI. Traboulsi, KW. Broman, C. Dibernardo, JS. Sunness, J. Toy, and EM. Weinberg. Extreme hyperopia is the result of null mutations in mfrp, which encodes a frizzled-related protein. Proc Natl Acad Sci U S A, 102(27): 9553–9558, 2005. PMCID: PMC1172243, PMID: 15976030.

[464] T. Yamamoto, MA. Mencarelli, C. Di Marco, M. Mucciolo, M. Vascotto, P. Balestri, M. Gérard, M. Mathieu-Dramard, J. Andrieux, M. Breuning, MJ. Hoffer, CA. Ruivenkamp, S. Shimada, N. Sangu, K. Shimojima, R. Umezu, H. Kawame, M. Matsuo, K. Saito, A. Renieri, and F. Mari. Overlapping microdeletions involving 15q22.2 narrow the critical region for intellectual disability to narg2 and rora. Eur J Med Genet, 57(4): 163–168, 2014. PMID: 24525055.

[465] T. Yamauchi, K. Hara, S. Maeda, K. Yasuda, A. Takahashi, M. Horikoshi, M. Nakamura, H. Fujita, N. Grarup, S. Cauchi, DP. Ng, RC. Ma, T. Tsunoda, M. Kubo, H. Watada, H. Maegawa, M. Okada-Iwabu, M. Iwabu, N. Shojima, HD. Shin, G. Andersen, DR. Witte, T. Jørgensen, T. Lauritzen, A. Sandbæk, T. Hansen, T. Ohshige, S. Omori, I. Saito, K. Kaku, H. Hirose, WY. So, D. Beury, JC. Chan, KS. Park, ES. Tai, C. Ito, Y. Tanaka, A. Kashiwagi, R. Kawamori, M. Kasuga, P. Froguel, O. Pedersen, N. Kamatani, Y. Nakamura, and T. Kadowaki. A genome-wide association study in the japanese population identifies susceptibility loci for type 2 diabetes at ube2e2 and c2cd4a-c2cd4b. Nat Genet, 42(10):864–868, 2010. PMID: 20818381.

[466] RN. Bamford, E. Roessler, RD. Burdine, U. Saplakoğlu, J. dela Cruz, M. Splitt, JA. Goodship, J. Towbin, P. Bowers, GB. Ferrero, B. Marino, AF. Schier, MM. Shen, M. Muenke, and B. Casey. Loss-of-function mutations in the egf-cfc gene cfc1 are associated with human left-right laterality defects. Nat Genet, 26(3):365–369, 2000. PMID: 11062482.

[467] JM. de la Cruz, RN. Bamford, RD. Burdine, E. Roessler, AJ. Barkovich, D. Donnai, AF. Schier, and M. Muenke. A loss-of-function mutation in the cfc domain of tdgf1 is associated with human forebrain defects. Hum Genet, 110(5):422–428, 2002. PMID: 12073012.

[468] F. Cottrez, E. Boitel, C. Auriault, P. Aeby, and H. Groux. Genes specifically modulated in sensitized skins allow the detection of sensitizers in a reconstructed human skin model. development of the sens-is assay. Toxicol In Vitro, 29(4):787–802, 2015. PMID: 25724174.
